# Supplementary material for: Sufficient conditions for rapid range expansion of a boreal conifer
Source: Nature. 2022 Aug 10;608(7923):546–51. doi: 10.1038/s41586-022-05093-2 (PMC9385489; doi:10.1038/s41586-022-05093-2)
Supplement: Supplementary file 1 — This file contains eight sections with calculations and equations that are annotated in support of the Methods and main text of the paper. [file 41586_2022_5093_MOESM1_ESM.pdf]

nature portfolio

https://doi.org/10.1038/s41586-022-05093-2

Supplementary information

Sufficient conditions for rapid range expansion of a boreal conifer

In the format provided by the authors and unedited

Supplementary Information Calculations for

Sufficient conditions for rapid range expansion of a boreal conifer

R. J. Dial, C. T. Maher, R. E. Hewitt, and P. F. Sullivan

Contents

1 Movement and Timing of White Spruce Range Expansion **4**
1.1 How big is the study area? 4
1.2 Digitizing 4
1.3 Field Validation 5
1.4 How many colonists are documented (minimum)? 6
1.5 How many kilometers did ground-transects cover in the study area? 6
1.6 Cone bearing trees and their heights 7
1.7 When did the 20 oldest aged trees establish? 10
1.8 Distance calculations 12
1.9 Northward movement 12
1.10 How far north in degrees and distance have adults moved from Kobuk treelines? 13
1.11 Convert from degrees to kilometers north 14
1.12 What is the rate of movement for the furthest forward adult? 14
1.13 How many juvenile records are we reporting? 14
1.14 How far north are the furthest forward juveniles from the nearest adults? 15
1.15 How far north are these furthest forward juveniles from the nearest established treelines? . . 17
1.16 How far are juveniles generally from adults? 18
1.17 How many juveniles are more than 300 m away from the nearest adult? 19
1.18 How many are more than 800 m away? 19
1.19 What are the velocities (as northward component of speed) and speed, given the year of establishment and distances of adults and juveniles from established treelines? 19
1.20 Northward migration velocity in km N/decade: 20
1.21 Migration speed in km/decade: 21

2 Calculating Age to Increment Core Height **22**
2.1 Using juvenile colonist growth rates to estimate age at core height 22

3 Calculating Relative Growth Rates (RGR) for Juveniles **27**
3.1 Growth Model and Assumptions 27
3.2 Data Wrangling 27
3.3 Calculating Age at 2 cm for Colonists using RGR 29
3.4 Statistics of coefficient of determination 31
3.5 Individual juvenile growth curves 31

4 Reconstructing Population Growth **57**
4.1 Modeling Height by Shadow Length 59
4.2 What's the probability of not casting a shadow given height? 66
4.3 Establishment-year density distributions by height class 72
4.4 1,000 simulations 77
4.5 Population simulations displayed 81

4.6 Sensitivity Analysis . . . 82

5 Foliar Sample Comparisons **90**
5.1 Linear mixed effects model comparing CAG . . . 90
5.2 Linear mixed effects model comparing foliar nutrients . . . 92

6 Relative Growth Rate (RGR) Comparison Between Colonist and Established Treeline Populations **95**
6.1 Linear mixed effects models comparing RGR . . . 95
6.2 Interaction model fixed effects coefficients with REML = T . . . 97

7 Tree Ring Correlation with July Air Temperatures **100**
7.1 Relationship between mean daily July temperatures in the AOI and Kotzebue . . . 100
7.2 Lapse rates in the Baird Mountains of the Brooks Range . . . 102
7.3 Tree ring indices . . . 108
7.4 Ages corrected by core height . . . 111
7.5 Data frame of ring indices and Kotzebue July air temperatures . . . 113
7.6 Growth trends by cambial age . . . 114
7.7 Cambial age growth across thirty year cohorts . . . 115
7.8 Correlations of tree ring metrics with July air temperature . . . 117
7.9 Juveniles and temperature . . . 118
7.10 Adults and temperature . . . 119
7.11 Plotting distibutions of correlations of BAI with July Kotzebue temperature . . . 121

8 Climate **123**
8.1 PRISM . . . 123
8.2 Constructing climate envelopes . . . 126
8.3 Kotzebue and the Chukchi Sea . . . 126
8.4 1937-2020 Kotzebue July Air Temperatures . . . 127
8.5 Chukchi Sea Ice . . . 129

```r
suppressMessages(library(tidyverse))
suppressMessages(library(dplyr))
suppressMessages(library(bookdown))
suppressMessages(library(MASS))
suppressMessages(library(broom))
suppressMessages(library(emmeans))
suppressMessages(library(raster))
suppressMessages(library(rgeos))
suppressMessages(library(sp))
suppressMessages(library(rgdal))
suppressMessages(library(sf))
suppressMessages(library(terra))
suppressMessages(library(stars))
suppressMessages(library(fields))
suppressMessages(library(ggridges))
suppressMessages(library(data.table))
suppressMessages(library(rcartocolor))
suppressMessages(library(lme4))
suppressMessages(library(dplR))
suppressMessages(library(janitor))
suppressMessages(library(knitr))
suppressMessages(library(cowplot))


#install.packages("remotes")
#remotes::install_github("clauswilke/ungeviz")
#remotes::install_github("zeehio/facetscales")
suppressMessages(library(ungeviz))
suppressMessages(library(facetscales))

geog <- "+proj=longlat +ellps=WGS84 +datum=WGS84 +no_defs"
utm4 <- "+proj=utm +zone=4 +datum=WGS84 +units=m +no_defs" #"+init=epsg:32604"
# Colorblind palette
cbPalette <- c("#999999",#gray 1
               "#E69F00",
               "#56B4E9",
               "#009E73", ## green 4
               "#F0E442", ## yellow 5
               "#0072B2",
               "#D55E00",
               "#CC79A7")
```

1 Movement and Timing of White Spruce Range Expansion

1.1 How big is the study area?

The main area of interest (AOI) consists of eight USGS HUC 10 watersheds. These watersheds include four watersheds of the Noatak basin (Kaluich, Cutler, Amakomanak, and Imelyak) that support a large population of white spruce (*Picea glauca*) colonists separated by the Baird Mountains from established treelines (**SI Fig. 1**) in four watersheds of the Kobuk basin (Redstone, Akillik, Hunt, and Miluet).

```
## Polygon digitized in Google Earth Pro
study.area <- st_read("1000sqkm.kml",quiet=T) %>%
        st_zm()
st_area(study.area) %>%
    format(big.mark   = ",")
```

```
## [1] "1,014,915,911 [m^2]"
```

1.2 Digitizing

The study used WorldView satellite (WV) images, both WorldView-1 and WorldView-2, to manually count white spruce (*Picea glacua*) trees by their shadows, visible across the deep snow of March and April 2018 when the abundant tall willows (*Salix spp.*) of the area of interest (AOI; Fig. 1a, ED Fig. 1a) were buried. Here we describe the approach, its validity, and applicability.

Satellite data were mainly used to scale field observations to larger areas by manually digitizing shadows in Google Earth Pro (GEP). The digitizing was accomplished by a single individual (ST) under the quality assurance and quality control (QAQC) supervision of the first author (RJD), who also digitized <100 shadows missed by the technician but subsequently included with the dataset. There was no systematic or quantitative estimate of congruence between ST and RJD, but in general the QAQC comparisons of shadows lengths with digitized lines during spot checks suggested that shadows digitized by ST and RJD were within ± 2m in length, equivalent to about four pixels and when multiplied by the tangent of the sun elevation angle in the region where we reconstructed historic populations is equivalent to 0.7 m in tree height.

In order to digitize the WV imagery in GEP, we first converted the imagery to "super-overlays" (https://developers.google.com/kml/documentation/kml_21tutorial?csw=1#superoverlays). This process degraded the images such that shadows from very small trees and the tops of tall trees were either lost or became ambiguous. We have not quantified the drop-out rate, but in the visual comparison of the scene shown in main text Fig. 1b (constructed using the *raster* package in R and shown also as **SI Fig. 5**) with that using the super-overlay from GEP (**SI Fig. 6**), it is apparent that the digitizing technician missed the smaller trees.

We are quite certain the the spruce tree shadows digitized are white and not black spruce (*P. mariana*). Black spruce are uncommon at treeline in the Brooks Range and we found none during field surveys in the Noatak watersheds of the AOI. Similarly, we observed few cottonwoods (*Populus balsamifera*) upstream of the confluence of the Imelyak and Cutler Rivers. The most abundant woody vegetation is shrub cover from dwarf to very tall.

Shrubs in the AOI are mostly less than 2.5 m tall, although some reach 5 m, especially in riparian areas where felt-leaf willow (*Salix alaxensis*) thrives. When digitizing, it was generally possible to distinguish spruce shadows from those of very tall willow. Spruce cast blacker, more triangular or linear shadows than the grayer, more diffuse and broom-topped shadows of willow (**SI Figs. 5-10**, especially **SI Figs. 7-8**). The most confusion was between short spruce and tall shrubs. Short spruce were not visible on summer scenes, which if visible in summer could have aided in distinguishing from tall shrubs. Also some spruce cast longer, more linear shadows in winter scenes, obscuring spruce individuals that were collinear with shadows. Overall, we documented errors of commission of 7.0% and of omission of 5.8% (see **Field Validation** below).

1.3 Field Validation

The digitizing technician used the ruler tool in GEP to measure from the base of the shadow to its tip. We made three field expeditions to the AOI to gather information on individual trees for matching with shadows identified by the technician using WV imagery. The ease of matching depended on the spatial distribution of trees (the trees were generally widely spaced–see main text Fig. 1 and **SI Figs. 5-10**). The georeferencing accuracy of the imagery, as reported by Maxar with the image metadata, was RMSE = 3.9 m. The built-in GNSS on late-model iPhones we used for field surveys was probably around 3 m, as the landscape is open and trees are sparse. The WV images were acquired at 5-23° off-nadir giving confidence in the orthorectification accuracy. When off-nadir angle is $\geq 25°$, positional accuracy in orthorectified imagery tends to be poorer than imagery captured more directly overhead.

As a measure of our ability to correctly identify trees among the 1,971 digitized shadows within the region of population reconstruction (small central rectangle in ED Fig. 1a and red rectangles in **SI Fig. 1-4**), we visited 157 shadow locations (8% of the 1,971 shadow locations) to collect heights and other data. Of these 157 shadow locations, 11 shadows were cast by very tall willows (7%); nine of these willows were within a 9 ha area of riparian flood plain and two were on hillsides, the latter being where most spruce grow.

Of the 146 shadows confirmed as trees, two were dead (1%), and another suffered most likely wind damage, as we found a recently broken $\approx$ 1.5 m green crown laying on the ground. We added its length to the standing height measured with a laser range-finder.

Finally, as a measure of omission, we found three sets of trees that were collinear, such that the single digitized shadow length overestimated the height of the tree standing to the SW (toward the sun) and missed the other tree(s) to the NE that cast their overlapping shadows in line with the first. Six trees among the 157 field-checked shadows fell into this category of omission and an additional three trees were missed during digitizing but observed in the field near shadow locations.

In summary, 155 trees were identified in the field where we expected 157. Applying this fraction overall suggest $(155/157) * 1971 = 0.987 * 1,971 = 1,945$ trees represent the reconstructed population. Overall in the AOI, the total adult count of 5,988 individuals based on shadows may be closer to 5,910 trees. Moreover, in so far as our estimates of age based on tree height are usefully predictive, perhaps 2% of the “trees” in our reconstruction are not a single tree casting long shadows, but 2-3 younger, collinear trees. Thus our estimate of past populations may be slightly biased to older trees, implying that the population growth rate may be slightly higher than estimated.

```
mat <- data.frame(Field_tree=c(146,9,155),
                  Field_non_tree=c(11,NA,NA),
                  Image_total_trees=c(157,NA,NA))
rownames(mat)=c("Image_tree","Image_non_tree","Field_total_trees")
kable(mat, caption = "Classification error matrix.")
```

Classification error matrix.

	Field_tree	Field_non_tree	Image_total_trees
Image_tree	146	11	157
Image_non_tree	9	NA	NA
Field_total_trees	155	NA	NA

Upon return from the field, RJD plotted the digitized shadow points and the field points on a 27-inch monitor and visually matched the two. Usually the nearest shadow was matched to the field point, but shadows were also matched to field points based on shadow length as well as proximity.

For example, in the upper left corner of the super-overlay image **SI Fig. 9** there is a cluster of five shadows (marked at base with yellow bullets) and five field points located with GNSS (marked as green tree icons) with the field-measured heights of the trees (m) associated with the field points as green numbers.

In **SI Fig. 9** the field point marked "5.3" is farther from the longest shadow base than is the field point marked "3.3" that is located to the right. Similarly, a second field point also marked "3.3" and to the left is closer to the second longest shadow in the cluster and located just below the number. Because the two shadows to either side (left and right) of the longest shadow are approximately equal and each is relatively near a field point labeled "3.3", they were assigned the nearby heights 3.3 m, and the longest shadow was assigned a height of 5.3 m. Similarly, the two mid-length shadows below the three shadows near the top edge are approximately equal and so matched with the field heights of 4.1 m and 4 m.

We made field expeditions to six study areas: three inside the red rectangles in **SI Figs. 2** and **4** ("GaiaHill," "BobWoods," and "Buffalo Drifts") and three other areas to the east and shown as clusters of small, green tree icons in **SI Fig. 2** ("Cutler," "AmakW," and "AmakE"). Among-area variability was apparent in the snow depth, terrain slope angle relative to the sun azimuth at the time of image capture, and in the sun elevation angle itself due to the timing of image capture. The variability was identified from the height of trees casting shadows and from the slope and intercept of linear regressions of field-measured height on digitized shadow length.These consideraations are discussed below in **SI Calc. 4.1-4.2.**

1.4 How many colonists are documented (minimum)?

The AOI is located between 156.5° and 159° W, although the file "LatLongMaxEntAll.csv" contains juvenile colonists outside the AOI. The file "BestShadowData.csv" contains the digitized shadows from the AOI.

```
shadows <- read.csv("BestShadowData.csv")
```

A total of 5,988 shadows were digitized using super-overlays of very-high resolution imagery imported into Google Earth Pro by one technician with QAQC overseen by RJD (SI Fig. 1). All observed shadows that appeared cast by spruce (*i.e.,* black pixels in a triangular or thin linear arrangement) were measured for length from base to the pixels judged as most likely to be the tip of the shadow. Shadows were digitized "wall-to-wall" across the available imagery shown in **SI Fig. 1** and within the Noatak watersheds of the AOI.

```
t19 <- read.csv("./2019_09_TreesWithHeights.csv")
t20 <- read.csv("./2020_09_TreesWithHeights.csv")
t21 <- read.csv("./2021_07_TreesWithHeights.csv")
```

A subset of the 5,988 shadow locations were visited in the field where 353 individuals were measured (**SI Fig. 2**). Details of the correspondence between shadows and ground-truthed trees are given below in **SI Calc. 4, Reconstructing Population Growth.**

```
juveniles <- read.csv("LatLongMaxEntAll.csv") %>%
    filter(lon.W > 156.5 & lon.W < 159) %>%
    filter(AgeClass !="adult")
```

The total 770 juveniles located in the field over the course of four field expeditions to the AOI are shown in **SI Fig. 3.** The sum of these two counts is 6,758 geo-located individuals.

1.5 How many kilometers did ground-transects cover in the study area?

```
fils <- dir()
    fils <- fils[str_sub(fils,-3,-1)=="kml"]
    fils <- fils[fils != "1000sqkm.kml"]
    bucket <- rep(NA,length(fils))

    for(i in 1:length(fils)){
        bucket[i] <- st_read(fils[i],quiet=T) %>% st_zm() %>% st_length()}
```

Using GNSS ground tracks from field campaigns and summing their lengths gives 403 km.

1.6 Cone bearing trees and their heights

We measured heights and cone-bearing conditions of individuals during, after and prior to the Brooks Range-wide mast seeding event of 2020. We classified trees based on cone count classes. In the year previous to the mast, we searched the ground below trees as well crowns for evidence of cones.

```
Cones.2021 <- read.csv("HeightsPlus_summer2021.csv") %>%
    dplyr::mutate(Year="post-mast",
                  Cones=Cones,
                  Height.m=Height.m,
                  Area=Area,
                  .keep="none") %>%
    na.omit()

Cones.2020 <- read.csv("CutlerSpruce2020.csv") %>%
    dplyr::mutate(Year="mast-event",
                  Cones=Cones,
                  Height.m=Height.m,
                  Area=Area,
                  .keep="none")

Cones.2019 <- read.csv("CutlerSpruce2019Cones.csv") %>%
    dplyr::mutate(Year="pre-mast",
                  Cones=Cones,
                  Height.m=Height.m,
                  Area=Area,
                  .keep="none")

Cone.data <- rbind(Cones.2019,Cones.2020, Cones.2021) %>%
    dplyr::mutate(Year=factor(Year,
             levels=c("pre-mast","mast-event","post-mast"))) %>%
    subset(Height.m > 1) %>%
    subset(Cones !="Broken dead") %>%
    na.omit()

Cone.data$Cones <- factor(Cone.data$Cones,
    levels=c("0",
        "1 to 10",
        "10 to 50",
        "50 to 100",
        "100 to 200",
        "200 to 400",
        "400 to 1000"))
```

1.6.1 How many trees over 1 m tall were recorded for signs of cones?

```
paste("Sample size n =",nrow(Cone.data), "trees")
```

```
## [1] "Sample size n = 336 trees"
```

```
Cone.data %>% tabyl(Year) %>% adorn_pct_formatting(digits=0) %>%
    kable(caption="Distribution of all cone-bearing trees by year.")
```

1.6.1.1 The frequency of sample spruce with cones by year, as "pre-mast", "mast", and "post-mass."

Distribution of all cone-bearing trees by year.

Year	n	percent
pre-mast	76	23%
mast-event	149	44%
post-mast	111	33%

1.6.2 For each cone count-class, what was the fraction of trees sampled by year?

Overall cone counts for all three years among the 336 trees recorded for cone counts:

```
Cone.data %>% tabyl(Cones) %>% adorn_pct_formatting(digits=0) %>%
    kable(caption="Cone count distribution.")
```

Cone count distribution.

Cones	n	percent
0	110	33%
1 to 10	53	16%
10 to 50	105	31%
50 to 100	43	13%
100 to 200	16	5%
200 to 400	6	2%
400 to 1000	3	1%

Here are the percentages of total trees observed each year by cone-count class (columns sum to one within rounding errors).

```
Cone.data %>% tabyl(Cones,Year) %>%
    adorn_percentages("col") %>%
    adorn_pct_formatting(digits=0) %>% kable(caption="Cone count distribution by year.")
```

Cone count distribution by year.

Cones	pre-mast	mast-event	post-mast
0	76%	20%	20%
1 to 10	14%	18%	14%
10 to 50	8%	48%	24%
50 to 100	0%	12%	23%
100 to 200	1%	1%	12%
200 to 400	0%	0%	5%
400 to 1000	0%	0%	3%

1.6.3 What is the relationship between tree height and cone count during the mast and post mast years?

```
cones.ag <- aggregate(Height.m~Cones,
            FUN=min,
            data=Cone.data,
            subset=Year != "pre-mast")

cones.ag$count <- aggregate(Height.m~Cones,
            FUN=function(x) sum(!is.na(x)),
            data=Cone.data,
            subset=Year != "pre-mast")$Height.m

cones.ag$MAx.height.m <- aggregate(Height.m~Cones,
            FUN=function(x) round(max(x),1),
            data=Cone.data,
            subset=Year != "pre-mast")$Height.m

names(cones.ag) <- c("Cones",
        "Min.height.m",
        "Total.tree.count",
        "Max.height.m")

cones.ag %>%
    kable(caption="Cone count by tree height.")
```

Cone count by tree height.

Cones	Min.height.m	Total.tree.count	Max.height.m
0	1.2	52	6.5
1 to 10	1.8	42	5.6
10 to 50	2.0	99	6.7
50 to 100	3.8	43	9.5
100 to 200	3.9	15	7.9
200 to 400	6.1	6	10.7
400 to 1000	5.8	3	8.8

1.6.4 How many trees observed had cones?

Total number of cone bearing trees:

```
paste(sum(Cone.data$Cones != "0"), "trees")
```

```
## [1] "226 trees"
```

1.6.5 And how many of those trees with cones were 2.5 m or taller?

```
cone.trees <- Cone.data %>%
    subset(Cones != "0")
```

Number of cone-bearing trees greater than 2.5 m tall:

```
n.over2.5m <- cone.trees %>%
    subset(Height.m >= 2.5) %>%
    nrow()

paste(n.over2.5m, "trees")
```

```
## [1] "223 trees"
```

Trees under 2.5 m tall with cones by their height and area:

```
row.names(cone.trees) <- NULL
cone.trees %>%
    filter(Height.m < 2.5) %>%
    arrange(Height.m)  %>%
    kable(caption="Height of three juvenile-sized trees with cones.")
```

Height of three juvenile-sized trees with cones.

Cones	Area	Height.m	Year
1 to 10	AmakW	1.80	post-mast
10 to 50	AmakW	2.00	post-mast
1 to 10	W. Fork Cutler	2.26	mast-event

Percentage of trees with cones over 2.5 m:

```
(100*n.over2.5m/nrow(cone.trees)) %>%
     round() %>%
    paste0("%")
```

```
## [1] "99%"
```

Shortest cone-bearing tree:

```
min(cone.trees$Height.m) %>%
    paste("meters tall")
```

```
## [1] "1.8 meters tall"
```

1.7 When did the 20 oldest aged trees establish?

This uses data that relies on results from **SI Calc. 2,** a supplement that shows calculations of establishment years using relative growth rates of juveniles (RGR), and on results from **SI Calc. 3,** a supplement that shows calculations of RGR.

```
aged.trees <- read.csv("./AgingIncrementCoreHeight V3.csv") %>%
    dplyr::mutate(est.year=Establishment_yr) %>%
    subset(est.year < 1950)  %>%
    arrange(est.year)

aged.trees %>%
    dplyr::mutate(est.year,
        year.class=cut(est.year,
            breaks=c(1900,1910,1920,1930,1940,1950)),
            .keep="none")  %>%
    aggregate(est.year~year.class,
```

```
                        FUN=length,
                        data=.) %>%
    dplyr::mutate(tree.count=est.year,
                 year.class=c("(1900,1910]",
                    "(1910,1920]",
                    "(1920,1930]",
                    "(1930,1940]",
                    "(1940,1950]"),
                 .keep="none") %>%
    kable(caption="Count of 20 oldest trees
          by establishment decade.")
```

Count of 20 oldest trees by establishment decade.

year.class	tree.count
(1900,1910]	1
(1910,1920]	3
(1920,1930]	2
(1930,1940]	5
(1940,1950]	9

The rather large increase in *tree.count* approximately 3-4 decades after the oldest individuals established (*i.e.,* identified as "(1900,1910]" and "(1910,1920])", suggests local reproduction when the earliest colonists matured[26] at about 30 years.

1.7.1 How far are the eight oldest trees from the established Kobuk treelines?

The eight oldest tree establishment years, 1901, 1914, 1917, 1919, 1928, 1930, 1932, 1933, cover 32 years and were likely all long-distance dispersal (LDD) events from seeds sourced at the Kobuk treeline or beyond, since white spruce produce seeds in quantity[26] at 30 y old.

```
BRTL.coords <- readOGR("BRTL.AOI.shp",
               verbose = FALSE) %>%
          coordinates()

oldest.trees.dist <-    rdist.earth(aged.trees[,c("lon","lat")],
        BRTL.coords,
        miles=F) %>%
    apply(1,min) %>%
    range() %>%
    round(1)

paste("Oldest colonists are",
      min(oldest.trees.dist),
      "to",max(oldest.trees.dist),
      "km from established treelines")
```

```
## [1] "Oldest colonists are 5.1 to 13.5 km from established treelines"
```

1.8 Distance calculations

1.8.1 How far are the colonists from nearest established treelines?

SI Figure 1 shows the digitized adult spruce identified from shadows (yellow dots) and the established treelines (green lines). We found the distances between each spruce shadow and each established treeline vertex and from those distances we identified the nearest vertex. We followed the same procedure for juveniles shown in **SI Fig. 3.**

From these two sets of distances we calculated the range of colonist-to-established-treeline distances in kilometers using the **rdist.earth()** function in the *fields* package in R:

```
##### distances
juveniles <- juveniles %>% mutate(Long=-lon.W,
                                  Lat=lat.N)

distances.Adults <- rdist.earth(shadows[,c("long","lat")],
        BRTL.coords,
        miles=F)

distances.Juveniles <- rdist.earth(juveniles[,c("Long","Lat")],
        BRTL.coords,
        miles=F)

## Get nearest distances
adults <- apply(distances.Adults,1,min) %>%
    range() %>%
    round(1)

juves <- apply(distances.Juveniles,1,min) %>%
    range() %>%
    round(1)
```

The range in kilometers from colonists to established Brooks Range Treeline (BRTL) is

```
paste(min(c(adults,juves)),"to",max(c(adults,juves)),"km")
```

```
## [1] "1.3 to 42.1 km"
```

1.9 Northward movement

Estimating northward movement of spruce relied on locations of nearest treelines to each adult and of nearest adult to each juvenile. The difference in latitudes was then multiplied by the distance between two parallels of latitude 1° apart.

1.9.1 How many adults are mapped as shadows?

```
shadows <- read.csv("BestShadowData.csv")
nrow(shadows) %>%
    format(big.mark=",") %>%
    paste("adults")
```

```
## [1] "5,988 adults"
```

1.9.2 How many vertices along BRTL are contained in the Kobuk watersheds adjacent to the AOI?

```
BRTL.coords <- readOGR("BRTL.AOI.shp",
    verbose = FALSE) %>%
    coordinates()    %>%
    data.frame()

nrow(BRTL.coords)  %>%
    format(big.mark=",") %>%
    paste("vertices of treelines")
```

```
## [1] "3,366 vertices of treelines"
```

1.10 How far north in degrees and distance have adults moved from Kobuk treelines?

This is a four-step process:

1. Identify the nearest vertex for each adult shadow.
2. Establish the nearest latitude coordinates of the each adult and the nearest established treeline vertex.
3. Find displacement as difference in latitude between adult and nearest vertex.
4. Multiply by distance per degree north (km/°N) to estimate northward displacement in km.

Note that three adults were located farther north in the field (**SI Fig. 2** near word "River" in "Cutler River" label). These are appended to the shadow dataset.

```
##### Step 1: Find nearest established treeline vertex to each adult
northern.field.adults <- data.frame(long=c(-158.01011,-158.01649,-158.02017),
                lat=c(67.67165,67.67113,67.63182))

shadows.lat.lon <- rbind(shadows[,c("long","lat")],
                northern.field.adults)
distances.Adults <- rdist.earth(shadows.lat.lon,
    BRTL.coords,
    miles=F) %>%
apply(1,min)

which.ones <- rdist.earth(shadows.lat.lon,
    BRTL.coords,
    miles=F) %>%
apply(1,which.min)

shadows.lat.lon <- dplyr::mutate(shadows.lat.lon,
    lon.BRTL=BRTL.coords$coords.x1[which.ones],
    lat.BRTL=BRTL.coords$coords.x2[which.ones],
    degrees.N=lat-lat.BRTL)
```

1.10.1 Maximum adult distance north of established treeline in degrees:

```
max(shadows.lat.lon$degrees.N,na.rm=T) %>% round(3)
```

```
## [1] 0.156
```

1.11 Convert from degrees to kilometers north

To determine a conversion factor from degrees latitude north to kilometers north, we applied the **rdist.earth()** function along the meridian longitude -157.6891° that passes through the AOI and between degrees 67°N and 68°N that bound the AOI.

```
deg.to.km <- matrix(c(-157.6891,67,
        -157.6891,68),
        byrow=T,ncol=2,nrow=2) %>%
    rdist.earth(miles=F) %>%
    max(na.rm=T)

paste(round(deg.to.km,2),"km/degree")
```

```
## [1] "111.32 km/degree"
```

The distance in kilometers between 67° and 68° N is 111.32 km.

Multiplying all latitudinal displacements by this conversion factor gives displacements north in kilometers:

```
shadows.lat.lon <- dplyr::mutate(shadows.lat.lon,
        km.N=degrees.N*deg.to.km)
```

The maximum distance north (in kilometers) of an adult colonist is 17.4 km:

```
summary(shadows.lat.lon$km.N)
```

```
##    Min. 1st Qu.  Median    Mean 3rd Qu.    Max.
##  -1.620   3.458   5.561   5.375   7.648  17.357
```

The negative distance reflects that the nearest treeline may be north of a colonist because the treeline itself is not a straight east-west line.

The 95th percentile of distance north in kilometers:

```
quantile(prob=0.95,shadows.lat.lon$km.N) %>%
    round(2) %>%
    paste("km N")
```

```
## [1] "9.71 km N"
```

1.12 What is the rate of movement for the furthest forward adult?

Consider the year 1901 as the year of initial colonization and 2021 as the year of observation, giving an interval of 12 decades.

The rate of adult movement is 17.4 km over 12 decades or 1.4 km decade^{-1}

1.13 How many juvenile records are we reporting?

"LatLongMaxEntAll.csv" includes juveniles found outside the AOI, so we subset it to include only those juveniles within the longitudes of the AOI (**SI Fig. 3**).

```
juveniles <- read.csv("LatLongMaxEntAll.csv") %>%
    dplyr::mutate(Long = -lon.W,
                  Lat = lat.N) %>%
    subset(Long <= -156.5 & Long > -159) %>%
    subset(AgeClass !="adult") %>%
```

```
    dplyr::mutate(Long=Long,
                  Lat=Lat,
                  .keep="none")

paste(nrow(juveniles), "juveniles located in the field.")
```

```
## [1] "770 juveniles located in the field."
```

1.14 How far north are the furthest forward juveniles from the nearest adults?

1.14.1 In degrees north?

```
shadows <- read.csv("BestShadowData.csv")

##### Get nearest distances
distances.Juves2Adults <- rdist.earth(juveniles,
    shadows.lat.lon,
    miles=F) %>%
apply(1,min)

## get nearest treeline point and its location
which.ones <- rdist.earth(juveniles,
    shadows.lat.lon,
    miles=F) %>%
apply(1,which.min)

## find latitudes of nearest adults then find differences in latitudes
juveniles <- dplyr::mutate(juveniles,
        km.Juves2Adults=distances.Juves2Adults,
        lon.adults=shadows.lat.lon$long[which.ones],
        lat.adults=shadows.lat.lon$lat[which.ones],
        degrees.N=Lat-lat.adults)

paste("Maximum juvenile-from-adult northward displacement in degrees:",
      round(max(juveniles$degrees.N),3))
```

```
## [1] "Maximum juvenile-from-adult northward displacement in degrees: 0.206"
```

The furthest north juvenile is 0.206° north of the nearest adult. It is north of the Noatak River near the mouth of the Cutler River.

1.14.2 In kilometers north?

Here are all the furthest forward juveniles that are more than 5 km north of the nearest adult:

```
juveniles <- dplyr::mutate(juveniles,
    km.N=degrees.N*deg.to.km)

juves.over10km.away <- juveniles %>%
    subset(km.N > 5)

juves.over10km.away %>%
    arrange(desc(km.N)) %>%
```

```
    mutate(km.N = round(km.N,1)) %>%
    dplyr::select(km.N) %>%
    kable(caption="Distance from nearest adult
          of 14 most distant juveniles.")
```

Distance from nearest adult of 14 most distant juveniles.

km.N
22.9
21.8
15.0
9.8
9.6
9.3
9.3
9.3
9.3
9.3
9.3
9.3
9.3
9.3

How many kilometers apart are the 14 furthest forward juveniles, given we know their five locations?

```
most.distant <- which(juveniles$km.N > 5)

## locations of 14 furthest forward
pops <- matrix(c(-158.3433,67.87696,
        -158.331100,67.805425,
        -158.2288,67.86737,
        -158.2485,67.75948,
        -158.2225,67.75480),
        nrow=5,ncol=2,byrow=T)

closest <- RdistEarth(pops,miles=F) %>%
        apply(1,FUN=function(x) min(x[x>0]))

closest     %>%
    round(1) %>%
    data.frame(distance_to_nearest_others_in_km=.) %>%
    kable(caption="Distances among most distant juvenile populations.")
```

Distances among most distant juvenile populations.

distance_to_nearest_others_in_km
4.9
6.2
4.9
1.2
1.2

1.15 How far north are these furthest forward juveniles from the nearest established treelines?

```
BRTL.coords <- readOGR("BRTL.AOI.shp",
    verbose = FALSE) %>%
    coordinates() %>%
    data.frame()

farthest <- juveniles[most.distant,]

##### Get nearest distances
farthest$Juves2Treelines <- rdist.earth(farthest,
        BRTL.coords,
        miles=F) %>%
apply(1,min)

## get nearest treeline point and its location
which.ones <- rdist.earth(farthest,
    BRTL.coords,
    miles=F) %>%
apply(1,which.min)

## find latitudes of nearest treelines then find difference in latitudes
farthest <- dplyr::mutate(farthest,
    lon.BRTL=BRTL.coords$coords.x1[which.ones],
    lat.BRTL=BRTL.coords$coords.x2[which.ones],
    degrees.N=Lat-lat.BRTL,
  km.N=deg.to.km*degrees.N)
```

Range in northness metrics from established treelines for the 14 furthest forward juveniles calculated as:

```
farthest %>% summarize(North.displacement.deg=
            round(range(degrees.N),3),
            Metric=c("Min","Max")) %>%
    kable(caption="Degrees north of treeline of
          14 furthest forward juveniles.")
```

Degrees north of treeline of 14 furthest forward juveniles.

North.displacement.deg	Metric
0.239	Min
0.361	Max

```
farthest %>% summarize(North.displacement.km=
            round(range(km.N),1),
            Metric=c("Min","Max")) %>%
    kable(caption="Range from established treelines of 14 furthest forward juveniles.")
```

Range from established treelines of 14 furthest forward juveniles.

North.displacement.km	Metric
26.6	Min

North.displacement.km	Metric
40.2	Max

1.15.1 How far north of established treelines are the 14 furthest forward juveniles?

The juveniles range from 0.239° to 0.361° north of nearest BRTL treeline.

1.15.2 How many kilometers north of treelines?

The juveniles range from 26.6 km to 40.2 km north of nearest BRTL treeline.

1.15.3 How much growth was recently added for the furthest forward juveniles with growth measurements?

```
distant.juves <- read.csv("Farthest14Juveniles.csv")
```

Of the 14 farthest forward juveniles $n = 8$ had growth measurments. The mean fraction of total height added in most recent five years was 57% with sd = 14%.

1.16 How far are juveniles generally from adults?

This dataframe has the calculated distances for all juveniles using the approach from above:

```
juvenile.stats <- read.csv("JuvenileMovementStats.csv")
```

It shows that juvenile colonists were found to range from 393 m above and -216 m below nearest adults.

1.16.1 We used relative growth rates (RGR) to estimate year of establishment of the single most distant juvenile:

SI Calc. 3 shows calculations for estimating RGR of juveniles based on using RGR to estimate juvenile age and so year of establishment.

```
year.of.most.distant <- read.csv("Farthest14Juveniles.csv") %>%
    subset(lat == max(lat)) %>%
            .$raw.est.year

year.of.most.distant %>% sort()
```

```
## [1] 1997
```

1.16.2 The summary statistics for elevations (m.asl = meters above sea level) of all juvenile samples are:

```
juvenile.stats %>%
    mutate(juvenile.elev=round(juvenile.elev)) %>%
    summarize(Min.m.asl=min(juvenile.elev),
                Percentile25.m.asl=quantile(p=0.25,juvenile.elev),
                Median.m.asl=quantile(p=0.5,juvenile.elev),
```

```
                Mean.m.asl=round(mean(juvenile.elev)),
                Percentile75.m.asl=quantile(p=0.75,juvenile.elev),
                Percentile97.5.m.asl=quantile(p=0.975,juvenile.elev),
                Max.m.asl=max(juvenile.elev)) %>%
    kable(caption="Summary statistics of juvenile elevation (meters above sea level).")
```

Summary statistics of juvenile elevation (meters above sea level).

Min.m.asl	Percentile25.m.asl	Median.m.asl	Mean.m.asl	Percentile75.m.asl	Percentile97.5.m.asl	Max.m.asl
282	442	492	507	522	752.775	846

1.16.3 The median distance to nearest adult of the 770 samples of juveniles was 59 meters

```
round(1000*median(juvenile.stats$adult.dist.km)) %>%
    paste("m")
```

```
## [1] "59 m"
```

1.17 How many juveniles are more than 300 m away from the nearest adult?

Reference 26 cites 300 meters as long-distance dispersal (LDD) for white spruce.

```
tot.300 <- sum(juvenile.stats$adult.dist.km > 0.3)
    paste(tot.300,"juveniles,")
```

```
## [1] "244 juveniles,"
```

```
perc.300 <- round(100*tot.300/nrow(juvenile.stats))
```

or 32%.

1.18 How many are more than 800 m away?

Reference 27 cites 800 meters as LDD for white spruce.

```
tot.800 <- sum(juvenile.stats$adult.dist.km > 0.8)
    paste(tot.800,"juveniles,")
```

```
## [1] "99 juveniles,"
```

```
perc.800 <- round(100*tot.800/nrow(juvenile.stats))
```

or 13%.

1.19 What are the velocities (as northward component of speed) and speed, given the year of establishment and distances of adults and juveniles from established treelines?

```
year.of.most.distant <- read.csv("Farthest14Juveniles.csv") %>%
    subset(lat == max(lat)) %>%
            .$raw.est.year
```

```
juvenile.stats <- read.csv("JuvenileMovementStats.csv")

read.csv("AgingIncrementCoreHeight V3.csv") %>%
     summarize(year.of.establishment.of.oldest.tree=
            min(Establishment_yr,na.rm=T)) %>%
  kable(caption="Year of establishment of oldest trees.")
```

Year of establishment of oldest trees.

year.of.establishment.of.oldest.tree
1901

1.19.1 Number of years between colonization of Cutler basin and arrival of established seedling to Noatak River:

```
delta.y <- year.of.most.distant - 1901

delta.y %>%
    paste("years")
```

```
## [1] "96 years"
```

1.19.2 Kilometers from furthest forward juvenile to nearest treeline:

```
distance.from.treeline <- max(juvenile.stats$BRTL.km)

distance.from.treeline %>%
    round(1) %>%
    paste("km")
```

```
## [1] "42.1 km"
```

1.19.3 Furthest forward juvenile distance in kilometers north from nearest treeline:

```
distance.from.treeline.N <- max(juvenile.stats$diff.dist.N.BRTL.km)

distance.from.treeline.N %>%
    round(1) %>%
    paste("km")
```

```
## [1] "40.2 km"
```

1.20 Northward migration velocity in km N/decade:

```
(10*distance.from.treeline.N/delta.y) %>%
    round(1) %>%
    paste("km N/decade")
```

```
## [1] "4.2 km N/decade"
```

1.21 Migration speed in km/decade:

```
(10*distance.from.treeline/delta.y) %>%
    round(1) %>%
    paste("km/decade")
```

```
## [1] "4.4 km/decade"
```

2 Calculating Age to Increment Core Height

We used a sample of the 2015-2020 relative growth rates (RGR) from 300 juvenile colonists (calculations shown below in **SI Calc. 3**) to estimate the age that a cored tree was when it reached the height at which it was cored. We then subtracted this amount from the estimated establishment year of the cross-dated increment core.

The first assumption is that the five-years of growth follow an exponential curve

$$H(t) = H_0 \exp(RGRt)$$

where t is years after 2015 and H_0 was height in 2015. **SI Calculation 3** shows the theory and code used to derive RGR for each individual juvenile.

We used the RGR for each juvenile to estimate when the individual was 2 cm tall. We selected 2 cm as an estimate of the height of a successful seedling at the end of its first season and estimated the year the juvenile was 2 cm tall as $t = \ln(h_{2015}/2)/RGR$. We saved the individual-specific RGR ("growth.rate"), year at 2 cm ("year.at.2.cm"), and height in 2015 ("Initial.cm") within the file "Juvenile.RGR.age.lat.lon.csv" used here.

Across all 300 juvenile colonists, the correlation between the natural log of height in 2015 and the year it was 2 cm tall was strong and negative. The relationship between RGR and year formed the basis for estimating how many years it took a tree to reach the increment core height.

```
dat <- read.csv("./Juvenile.RGR.age.lat.lonV2.csv")

cor.test(~year.at.2cm+log(Initial.cm),
                 data=dat,
                 subset=Source=="colonist")
```

```
##
##  Pearson's product-moment correlation
##
## data:  year.at.2cm and log(Initial.cm)
## t = -16.163, df = 298, p-value < 2.2e-16
## alternative hypothesis: true correlation is not equal to 0
## 95 percent confidence interval:
##  -0.7394798 -0.6180683
## sample estimates:
##        cor
## -0.6834725
```

2.1 Using juvenile colonist growth rates to estimate age at core height

Because all the core heights from colonist adults were under 20 cm, we used only RGR from juveniles that were under 20 cm tall in 2015.

```
new.r <- read.csv("./Juvenile.RGR.age.lat.lonV2.csv") %>%
    filter(Initial.cm <= 20) %>%
    filter(Source=="colonist")
```

There were 83 juvenile colonists $\leq$ 20 cm from four areas in the AOI.

Using $H(t) = H_0 \exp(RGRt)$ with $H_0 = 2$ and H equal to the height, C, at the increment core hole, the time T to C from $H_0 = 2$ is

$$T = \frac{\ln(C/2)}{\bar{R}} = \beta \ln C + \beta_o,$$

where $\beta_o = -\ln(C)/\bar{R}$ and $\beta = 1/\bar{R}$, where $\bar{R}$ is some measure of average RGR.

We modeled this relationship using a linear mixed model with sample area as a random factor and T estimates the years before 2015 when a juvenile was 2 cm tall, given a height C in 2015.

We numerically adjusted the height of a successful seedling to arrive at an x-intercept of 2 cm. In particular, the **lmer()** fit with an x-intercept = 2 used $0.9 \neq 2$ in the denominator of the dividend as

$$T = \frac{\ln(C/0.9)}{RGR}.$$

```
mod.mer <- new.r  %>%
    lmer((I(2015-year.at.2cm)) ~ log(Initial.cm) + (1|Field.Site),
         data=.)

summary(mod.mer)
```

```
## Linear mixed model fit by REML ['lmerMod']
## Formula: (I(2015 - year.at.2cm)) ~ log(Initial.cm) + (1 | Field.Site)
##    Data: .
##
## REML criterion at convergence: 395.8
##
## Scaled residuals:
##     Min      1Q  Median      3Q     Max
## -1.8012 -0.5553 -0.2633  0.3613  3.7316
##
## Random effects:
##  Groups     Name        Variance Std.Dev.
##  Field.Site (Intercept) 13.351   3.654
##  Residual                6.356   2.521
## Number of obs: 83, groups:  Field.Site, 4
##
## Fixed effects:
##                 Estimate Std. Error t value
## (Intercept)      -0.9367     2.8161  -0.333
## log(Initial.cm)   4.1615     0.8204   5.073
##
## Correlation of Fixed Effects:
##             (Intr)
## lg(Intl.cm) -0.741
```

```
lmer.Intercept <- fixef(mod.mer)[1]

lmer.slope <- fixef(mod.mer)[2]
```

The relationship between T, "years-from-2-cm-to-core-height-above-ground, C", is T = -0.94 + 4.16 $\ln C$ where C is core height in cm above ground.

While this result is crude, without a large degree of destructive sampling in a scientifically sensitive area, it is convenient.

Applying it to the core samples gives for the first set:

```
cores1 <- read.csv("./Core.hts.2020_cleaned.csv") %>%
    dplyr::mutate(Colin.ID,
        TreeID,
        Core.ht.cm,
        estab.year.adj=round(lmer.Intercept+lmer.slope*log(Core.ht.cm)),
```

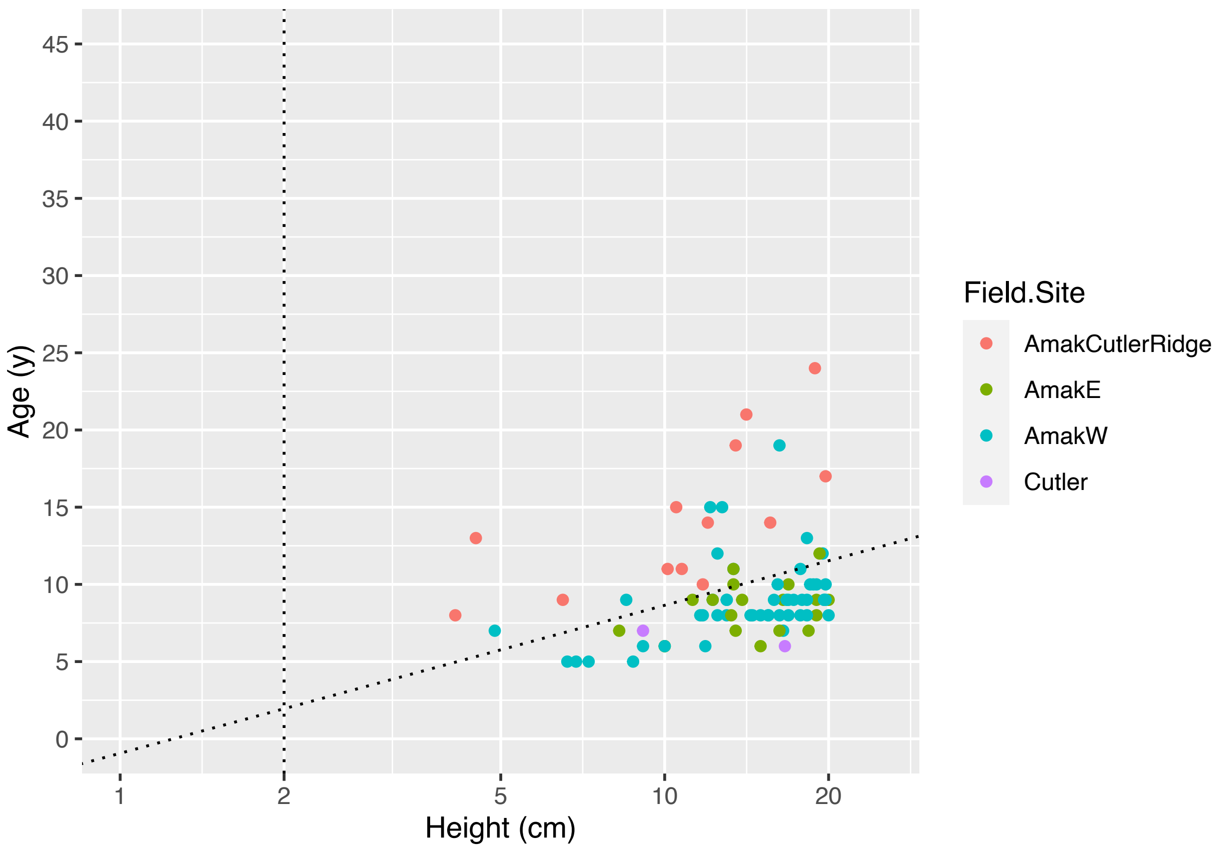

Estimated juvenile age against height. Age estimated using juvenile relative growth rate (RGR) to back-calculate number of years since 0.9 cm tall. n = 83 juvenile colonists. Dotted line has fixed effects slope and intercept from mixed effects model calculated above as T = -0.94 + 4.16 ln C.

```
        .keep="none") %>%
    merge(read.csv("./Cutler2020_tree_ages.csv"),
         by="Colin.ID") %>%
    dplyr::mutate(Colin.ID,
        TreeID,
        Core.ht.cm,
        Established.year=Est.yr-estab.year.adj,
        .keep="none")

cores1.m <- read.csv("./CutlerSpruce2020.csv") %>%
    dplyr::mutate(lat,lon,TreeID,Height.m,
        .keep="none") %>%
    merge(cores1,by="TreeID")
```

And the second set:

```
cores2 <- read.csv("./Core.hts.2019_cleaned.csv") %>%
    dplyr::mutate(Colin.ID,
        TreeID,
        Core.ht.cm,
        estab.year.adj=round(lmer.Intercept+lmer.slope*log(Core.ht.cm)),
      .keep="none") %>%
    merge(read.csv("./Cutler2019_tree_ages.csv"),
        by="Colin.ID") %>%
    dplyr::mutate(Colin.ID,
        TreeID,
        Core.ht.cm,
        Established.year=Est.yr-estab.year.adj,
        .keep="none")

cores2.m <- read.csv("./CutlerSpruce2019.csv") %>%
    dplyr::mutate(lat,
        lon=long,
        TreeID=ID,
        Height.m,
        keep="none") %>%
    merge(cores2,by="TreeID")
```

Put together:

```
aged.trees <- data.frame(Colin.ID=c(cores2.m$Colin.ID,cores1.m$Colin.ID),
        ID=c(cores2.m$TreeID,cores1.m$TreeID),
        Core.ht.cm=c(cores2.m$Core.ht.cm,cores1.m$Core.ht.cm),
        Established.year=c(cores2.m$Established.year,cores1.m$Established.year),
        lat=c(cores2.m$lat,cores1.m$lat),
        lon=c(cores2.m$lon,cores1.m$lon),
        Height.m=c(cores2.m$Height.m,cores1.m$Height.m))

paste(sum(!is.na(aged.trees$Established.year)), "trees with years of establishment")
```

```
## [1] "142 trees with years of establishment"
```

This dataset represents a total of 142 trees with years of establishment.

The heights above ground (cm) of the tree-cores are described as

```r
summary(aged.trees$Core.ht.cm)
```

```
##    Min. 1st Qu.  Median    Mean 3rd Qu.    Max.    NA's 
##   2.000   5.000   5.000   6.468  10.000  20.000       5
```

The dataset with estimated estimated years of establishment is saved as:

```r
# Not run
# write.csv(aged.trees,row.names=F,
#                    "./AgingIncrementCoreHeight V3.csv")
```

3 Calculating Relative Growth Rates (RGR) for Juveniles

We measured the height above ground of the most recent six bud scars on juvenile colonists encountered along transects through the AOI in four areas. We also did the same for juveniles within sample plots at 14 established treelines. These heights above ground provided relative growth rates (RGR) for each individual juvenile. The RGRs in turn provide a means to age juveniles and to age when an increment-cored tree first reached the height above ground of the increment bore hole (**SI Calc. 2**). We also use RGR to compare growth in colonists to established treeline juveniles (**SI Calc. 6**).

3.1 Growth Model and Assumptions

We assume that the five-years of growth follows an exponential curve

$$H(t) = H_0 \exp(RGRt)$$

where t is years after 2015 and H_0 is height in 2015. We use juveniles for which we measured the last six bud scar heights above ground, applying simple linear regression of $\log(H)$ on t, where the parameter of interest is RGR, the relative growth rate.

We chose the function **lm()** with semi-log regression rather than **nls()** with an exponential curve for two reasons. First **nls()** fails for those instances when growth is linear in time. Second, the residuals are heteroscedastic when height is not logged, violating an assumption of homoscedasticity in **nls()**.

The following code calculates the RGR for each individual and saves it as a data frame with associated metadata about that individual, including its location and sample area, as well as the calculated age at 2 cm. Section **SI Calc. 3.5** provides code and figures showing semi-log plots for the 505 individual juveniles (*i.e.*, height < 2.5 m) as the growth curve for each.

3.2 Data Wrangling

We select only the juvenile sized trees and create a "Year" variable centered at 2015.

```
suppressMessages(library(ggplot2))
suppressMessages(library(ggridges))
suppressMessages(library(tidyverse))
library(stringr)
library(lme4)

raw <- read.csv("./JuvenileGrowthRaw.csv") %>%
    filter(Height.cm < 250) %>%
    dplyr::mutate(Year=year-2015)
```

3.2.1 Upper Noatak Basin Juvenies

First we handle the juveniles from the Cutler River basin and from the Phillip Smith[55] treeline (67.997°, 161.521°). The Phillip Smith treeline marks the uppermost established treeline on the Noatak River.

The Cutler River basin and the Phillip Smith treeline are located within the Noatak basin. The Phillip Smith treeline is located about 135 km from the nearest known spruce in the AOI. Neither of these sites are part of the Brooks Range Treeline (BRTL) as defined here. BRTL juveniles are handled below and include juveniles measured at established experimental field sites at established treelines in the lower Noatak basin, the Kobuk basin, and the Koyukuk basin.

3.2.2 Summary of all bud scar heights (cm) above ground

```
summary(raw$Height.cm)
```

```
##    Min. 1st Qu.  Median    Mean 3rd Qu.    Max. 
##   4.125  27.250  46.125  61.203  80.000 248.625
```

3.2.3 Define vectors to hold the calculated values and metadata.

First setup some empty vectors:

"growth.rate" is RGR.

"Initial.cm" is the height above ground of the 2015 bud scar.

"R.sq" is the R^2 from the regression of $\ln(h)$ on t, the number of years after 2015.

"Phone" is the smartphone used in the field to record measurements.

"Field.Site" is the area sampled, generaly treated as a random factor.

"Source" is the population from which the juvenile was sampled: Colonist or Established Treeline.

"lat" and "lon" are GNSS locations of the juvenile.

"year.at.2cm" is the estimated year the juvenile established (**SI Calc. 3.3**).

```
IDs <- unique(raw$uniqueID)
how.many <- length(IDs)
    growth.rate <- rep(NA,how.many)
    Initial.cm <- rep(NA,how.many)
    R.sq <- rep(NA,how.many)
    Phone <- rep(NA,how.many)
    Field.Site <- rep(NA,how.many)
    Source <- rep(NA,how.many)
    lat <- rep(NA,how.many)
    lon <- rep(NA,how.many)
    year.at.2cm <- rep(NA,how.many)
```

Next, loop through all individuals in the Noatak basin filling the vectors defined above with values.

```
for(i in 1:how.many)
 {temp <- raw %>%
   subset(uniqueID==IDs[i])
   mod <- lm(log(Height.cm)~Year,
       data=temp)
   R.sq[i] <- summary(mod)$r.squared
   growth.rate[i] <- coef(mod)[2]
   Phone[i] <- unique(temp$Phone)
   Initial.cm[i] <- min(temp$Height.cm)
   Field.Site[i] <- unique(temp$Field.Site)
   Source[i]=unique(temp$Source)
   lon[i]=unique(temp$lon)
   lat[i]=unique(temp$lat)
   year.at.2cm[i]=
        round(2015-(log(min(temp$Height.cm)/2)/coef(mod)[2]))
}
```

Finally, save the summary statistics for all Noatak basin individuals

```
dat <- data.frame(Field.Site=Field.Site,
    ID=IDs,
    growth.rate=growth.rate,
    Phone=Phone, # field recording device
    Source=Source,
    Initial.cm=Initial.cm,
    R.sq=R.sq)

dat %>%
  tabyl(Field.Site) %>%
  adorn_pct_formatting(digits=0) %>%
  kable(caption="Frequency distribution of Noatak basin juvenile samples.")
```

Frequency distribution of Noatak basin juvenile samples.

Field.Site	n	percent
AmakCutlerRidge	19	5%
AmakE	79	22%
AmakW	195	54%
Cutler	7	2%
PhilipSmith	60	17%

3.3 Calculating Age at 2 cm for Colonists using RGR

The RGR is compared to H_0 to determine a relationship such that given the height in 2015, what would be the best estimate of how long it took to reach that height from 2 cm, the assumed height of a robust germinant at the end of its first growing season that has survived to 2021, the year of sampling. That is, given height h_{2015} find T so that:

$$h_{2015} = 2\exp(rT)$$

or

$$T = \frac{\log(h_{2015}/2)}{RGR}$$

Subtracting T from 2015 gives the year of establishment, here defined as "year.at.2cm".

```
DAT.noatak <- data.frame(lat=lat,
    lon=lon,
    Field.Site=Field.Site,
    ID=IDs,
    growth.rate=growth.rate,
    Phone=Phone,
    Source=Source,
    Initial.cm=Initial.cm,
    year.at.2cm=year.at.2cm,
    R.sq=R.sq)
```

This dataframe as used above in **SI Calc. 2 Calculating Age to Increment Core Height.**

3.3.1 Brooks Range Treeline (BRTL) juveniles

Now do something similar for all individuals from BRTL, leaving out some unused variables.

```
BRTL <- read.csv("./BRTL JuvenileGrowthRaw.csv")
```

3.3.2 Summary of all bud scar heights (cm) above ground

```
summary(BRTL$Height.cm)
```

```
##    Min. 1st Qu.  Median    Mean 3rd Qu.    Max.
##    1.20   18.52   42.00   49.91   76.45  142.00
```

```
BRTL <- BRTL %>%
    subset(Height.cm < 250) %>%
    dplyr::mutate(Year=year-2015)

# vectors defined
IDs <- unique(BRTL$uniqueID)
how.many <- length(IDs)
growth.rate <- rep(NA,how.many)
Initial.cm <- rep(NA,how.many)
R.sq <- rep(NA,how.many)
Phone <- rep(NA,how.many)
Field.Site <- rep(NA,how.many)
Source <- rep(NA,how.many)

for(i in 1:how.many)
 {temp <- BRTL %>%
    subset(uniqueID==IDs[i])
    mod <- lm(log(Height.cm)~Year,
        data=temp)
    R.sq[i] <- summary(mod)$r.squared
    growth.rate[i] <- coef(mod)[2]
    Phone[i] <- unique(temp$Phone)
    Initial.cm[i] <- min(temp$Height.cm)
    Field.Site[i] <- unique(temp$Field.Site)
    Source[i]=unique(temp$Source)
}

## Collect vectors into data frame
dat.BRTL <- data.frame(Field.Site=Field.Site,
    ID=IDs,
    growth.rate=growth.rate,
    Phone=Phone,
    Source=Source,
    Initial.cm=Initial.cm,
    R.sq=R.sq)

table(dat.BRTL$Field.Site)
```

```
##
## ALL 3  AML3  AMR1  KEL1  KER1  KUL1 KUR1   OML1  OMR1  TAL1  TAR1  TIL1  TIR1
##     5    15    14    12    12    12    13     6    11    12    12     9    12
```

3.4 Statistics of coefficient of determination

Here are summaries of the R^2 values from the semi-log regressions.

```
summary(DAT.noatak$R.sq[DAT.noatak$Source=="colonist"])
```

3.4.0.1 Colonist juvenile coefficients of determination

```
##    Min. 1st Qu.  Median    Mean 3rd Qu.    Max.
##  0.7414  0.9846  0.9913  0.9867  0.9955  0.9999
```

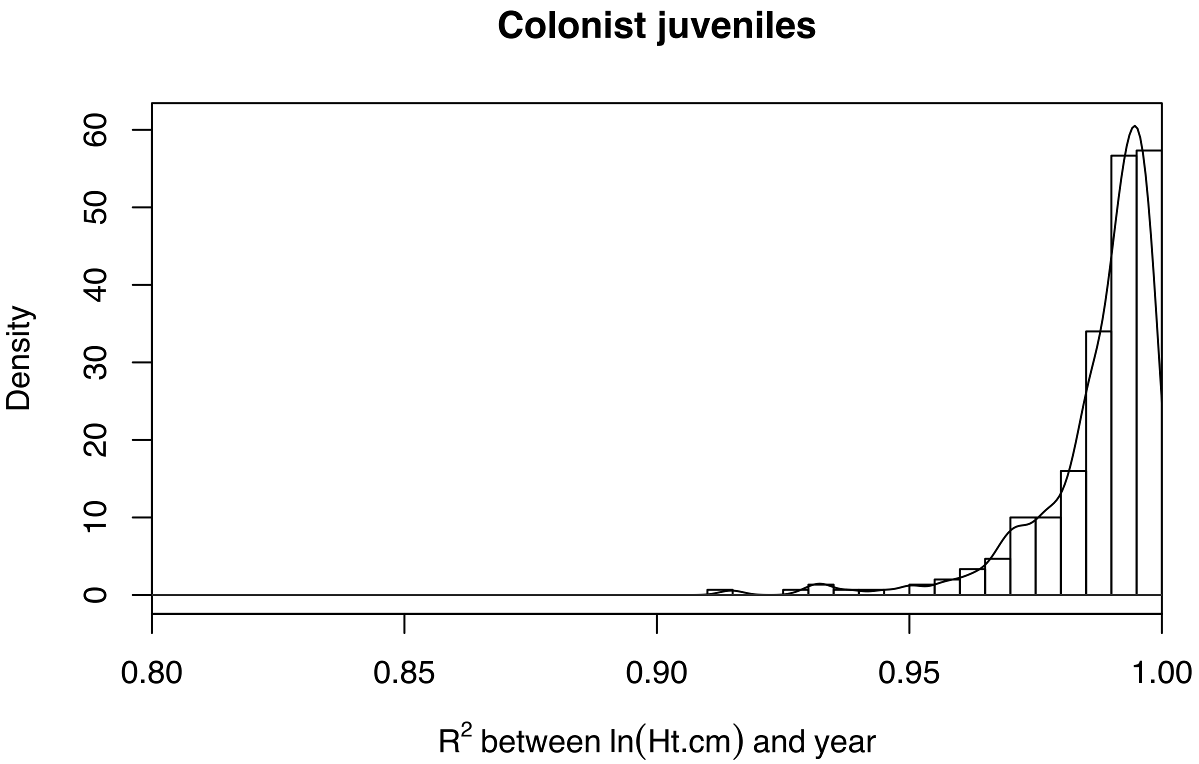

Distribution of coefficient of determination for juvenile colonist log(height) regressed on year.

```
summary(dat.BRTL$R.sq)
```

3.4.0.2 Established treeline juvenile coefficients of determination (R^2)

```
##    Min. 1st Qu.  Median    Mean 3rd Qu.    Max.
##  0.7071  0.9778  0.9892  0.9785  0.9943  0.9988
```

3.5 Individual juvenile growth curves

Below we present graphical representations of the most recent five years of growth for all juveniles measured, organized by sample area, and showing code for the first set of graphs. The remaining graphs are produced similarly, but code is hidden. In all plots, the y-axis is a log scale with heights (cm) indicated at 10, 40, and 160 cm.

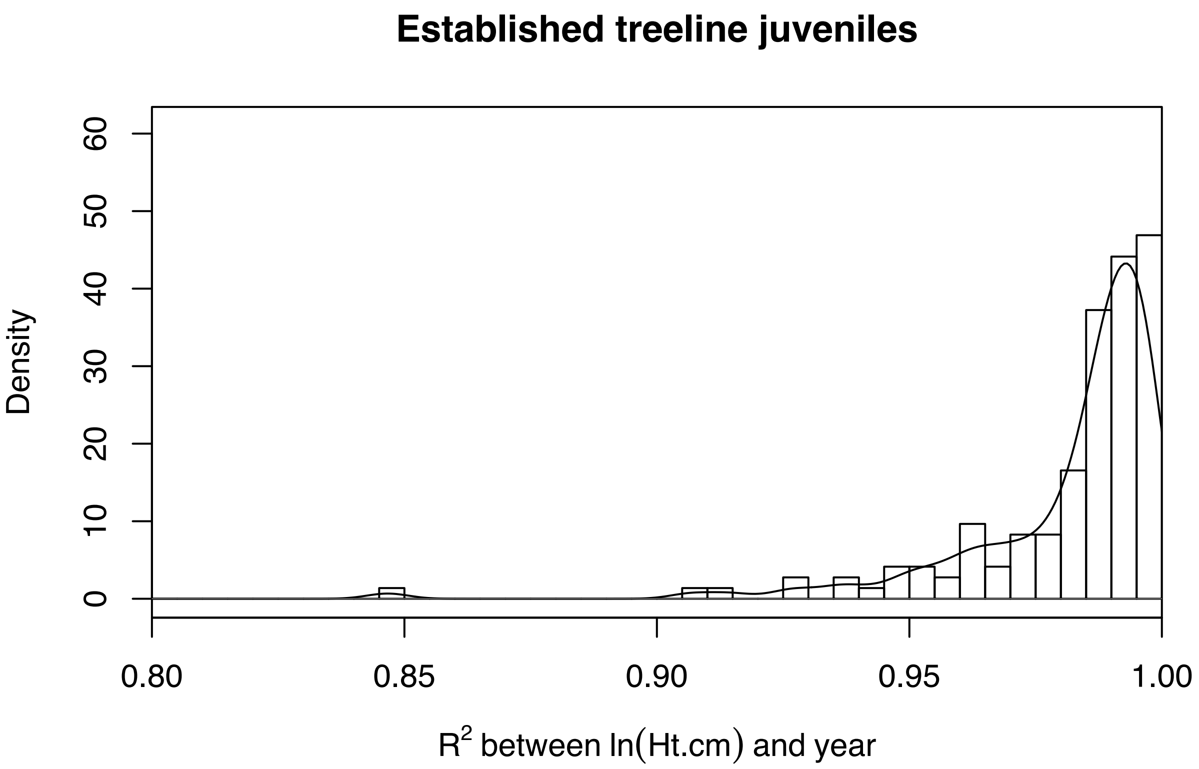

Distribution of coefficient of determination for juvenile log(height) at established treelines regressed on year.

3.5.1 300 individual Colonist growth curves used for estimating RGR

```
raw <- read.csv("./JuvenileGrowthRaw.csv")
```

There are four colonist areas with relative growth rates (RGR) calculated for each:

1. Cutler Colonist
2. AmakCutlerRidge
3. AmakE
4. AmakW

3.5.1.1 Cutler Cutler valley has 7 juvenile samples.

```
####  Cutler
raw %>% subset(Field.Site == "Cutler") %>%
    ggplot(aes(x=year-2015,y=log(Height.cm),col=uniqueID)) +
    geom_point() +
    facet_wrap(vars(uniqueID)) +
    geom_smooth(method = "lm",se=F) +
    guides(col="none") +
    scale_x_continuous(breaks=0:5,labels=2015:2020) +
        scale_y_continuous(breaks=log(c(10,40,160)),
        labels=c(10,40,160)) +
    labs(y="Height (cm)",
       x="") +
    theme(axis.text.x =
```

```
        element_text(angle = 90,
            vjust = 0.5,
            hjust=1))
```

```
## `geom_smooth()` using formula 'y ~ x'
```

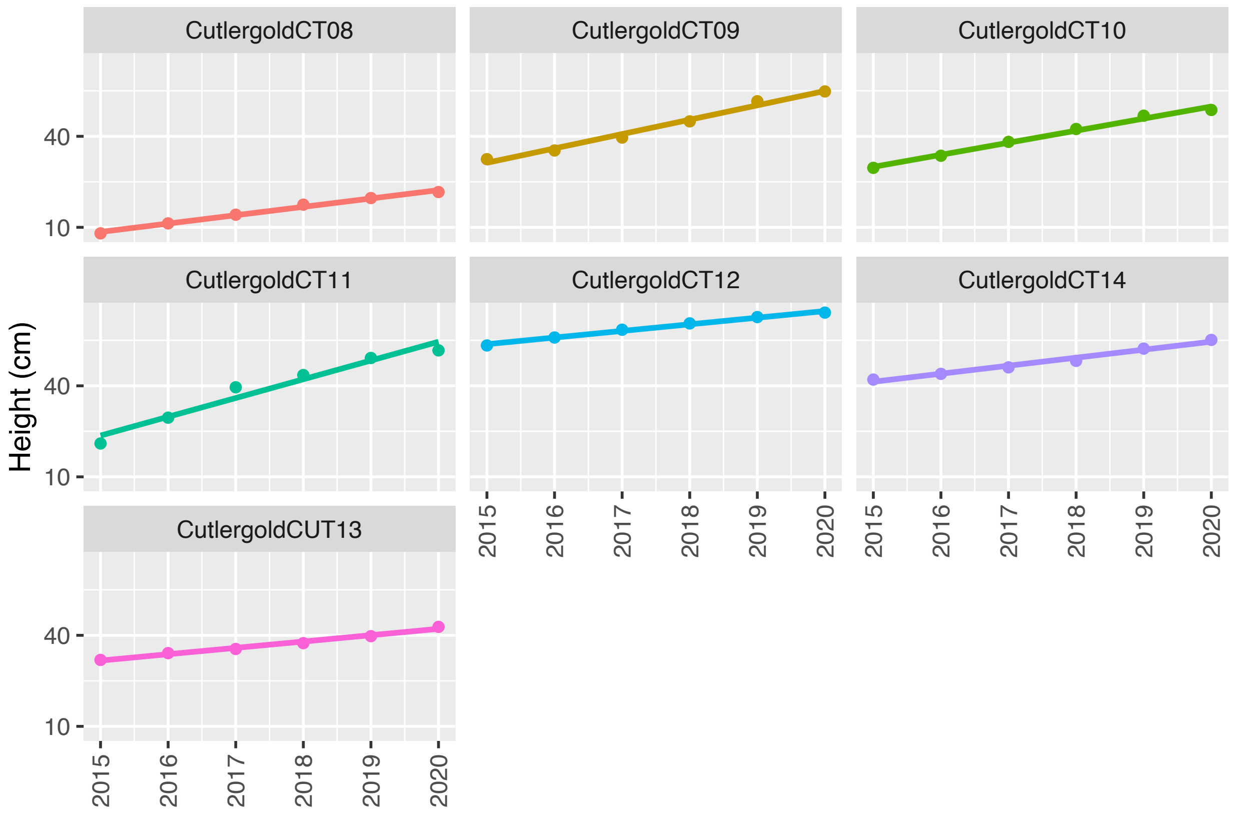

Growth curves for Cutler colonist juveniles.

3.5.1.2 AmakCutlerRidge

AmakCutlerRidge has 19 juvenile samples.

```
## `geom_smooth()` using formula 'y ~ x'
```

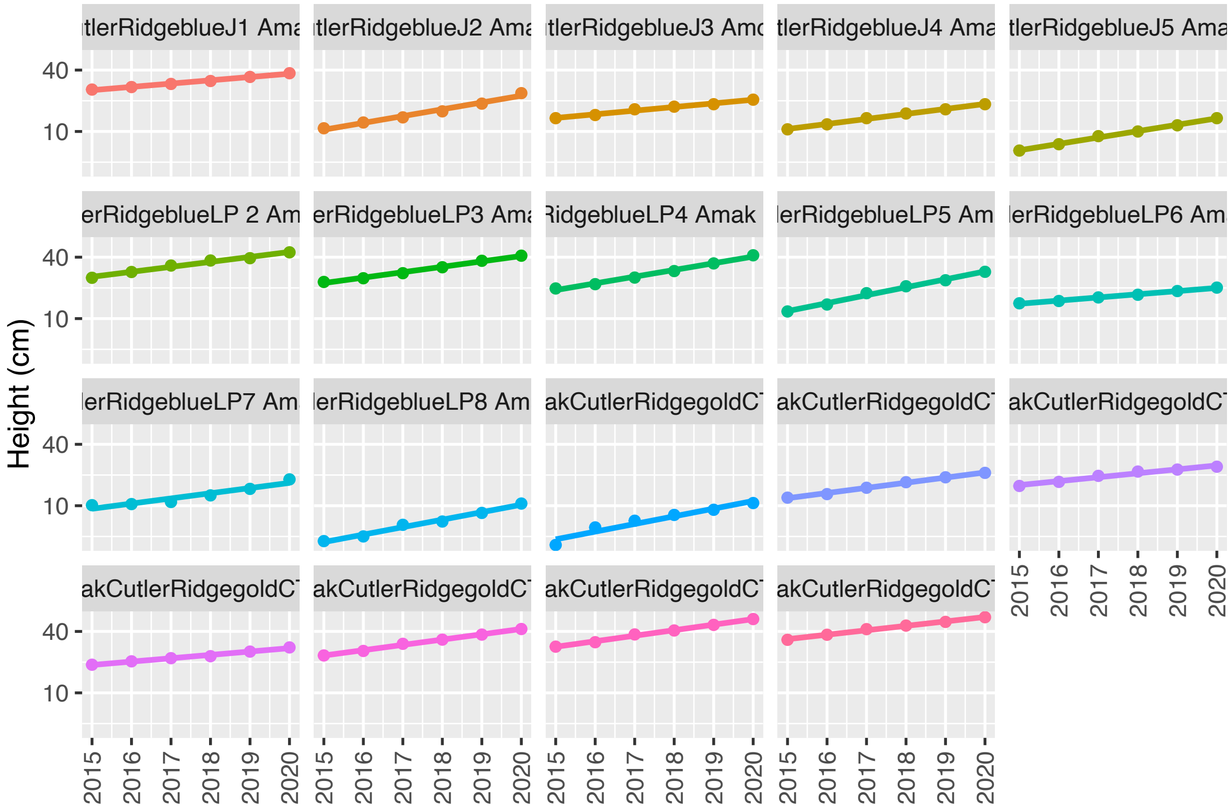

Growth curves for AmakCutlerRidge colonist juveniles.

3.5.1.3 AmakW Colonist growth curves by individual

AmakE area has 79 juvenile samples.

```
## `geom_smooth()` using formula 'y ~ x'
```

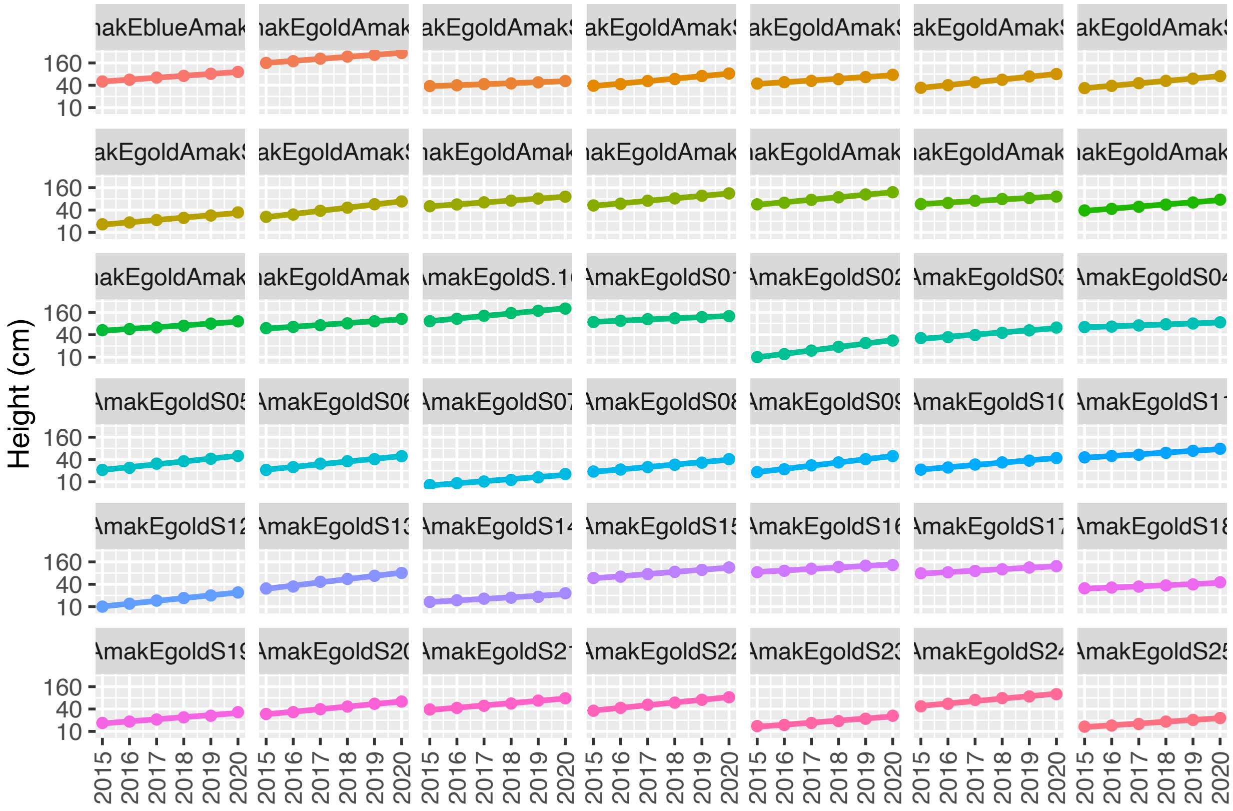

Growth curves for AmakE colonist juveniles.

```
## `geom_smooth()` using formula 'y ~ x'
```

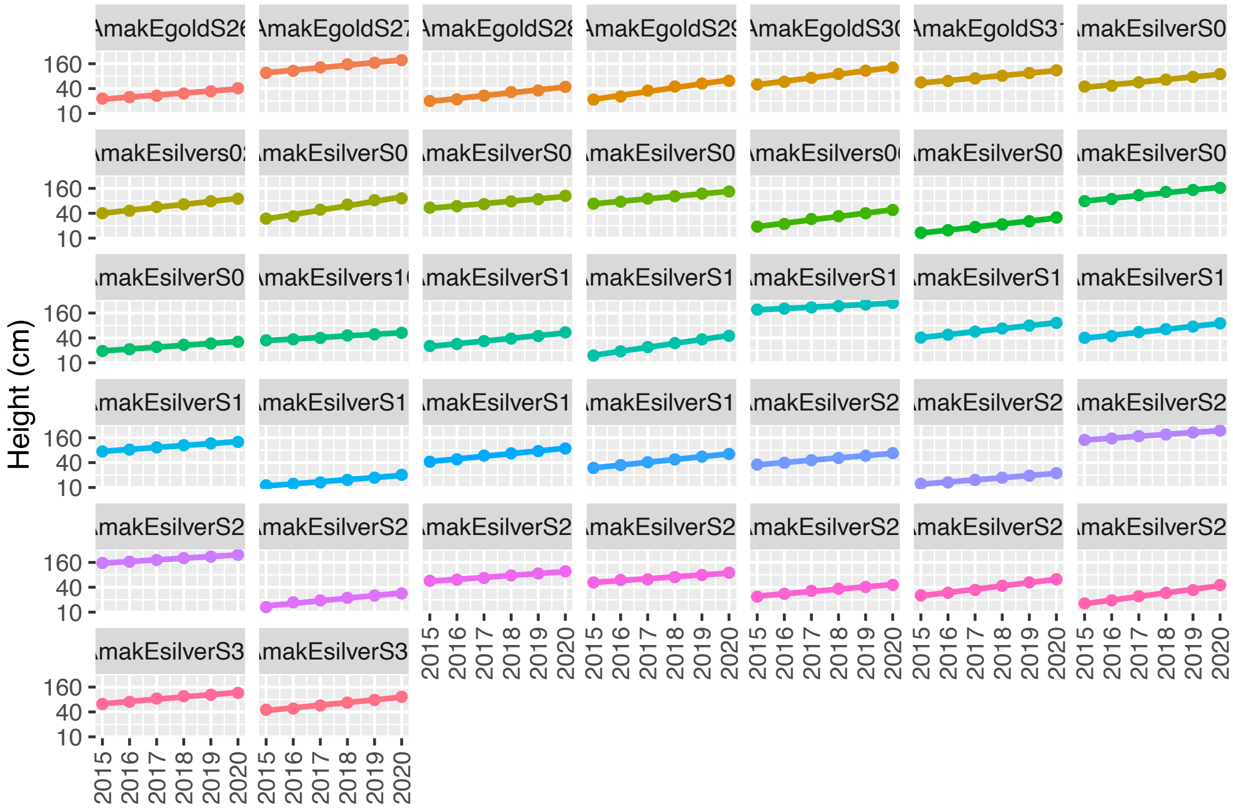

Growth curves for AmakE colonist juveniles.

3.5.1.4 AmakW Colonist growth curves by individual

AmakW area has 195 samples

```
## `geom_smooth()` using formula 'y ~ x'
```

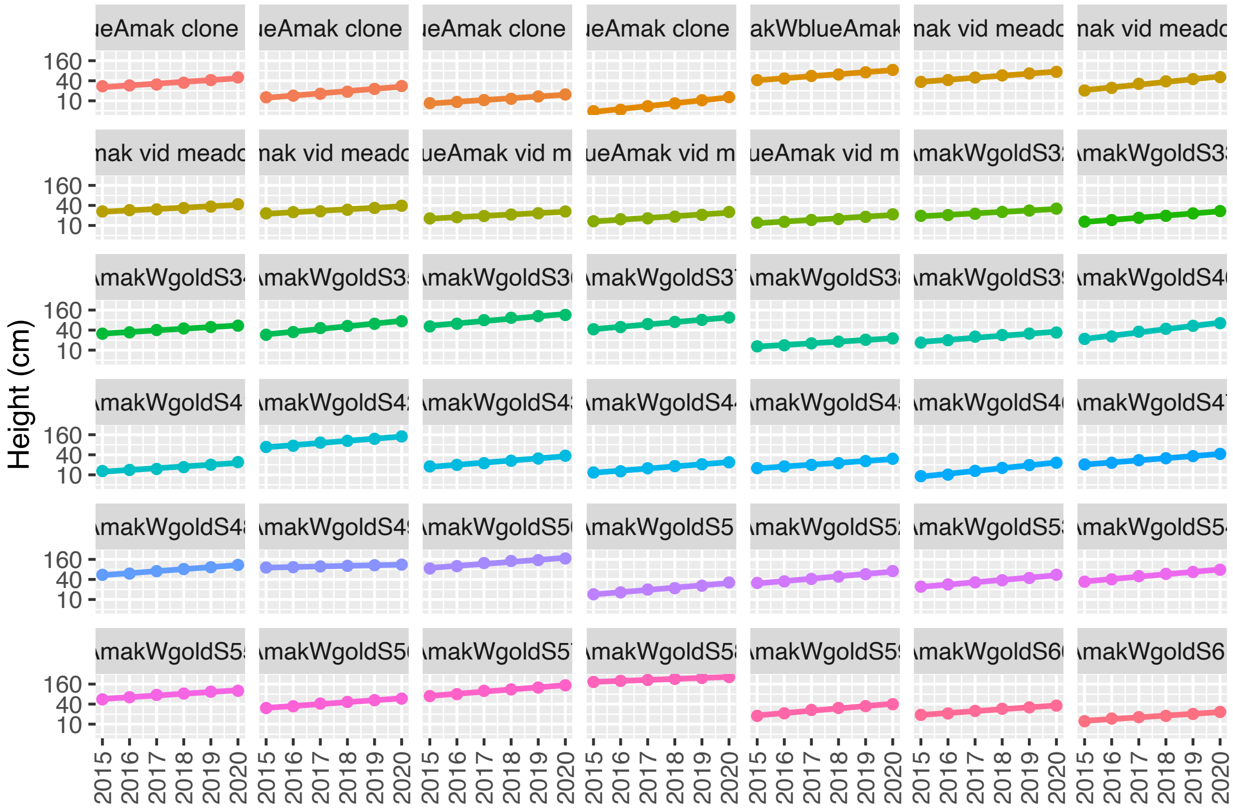

Growth curves for AmakW colonist juveniles.

```
## `geom_smooth()` using formula 'y ~ x'
```

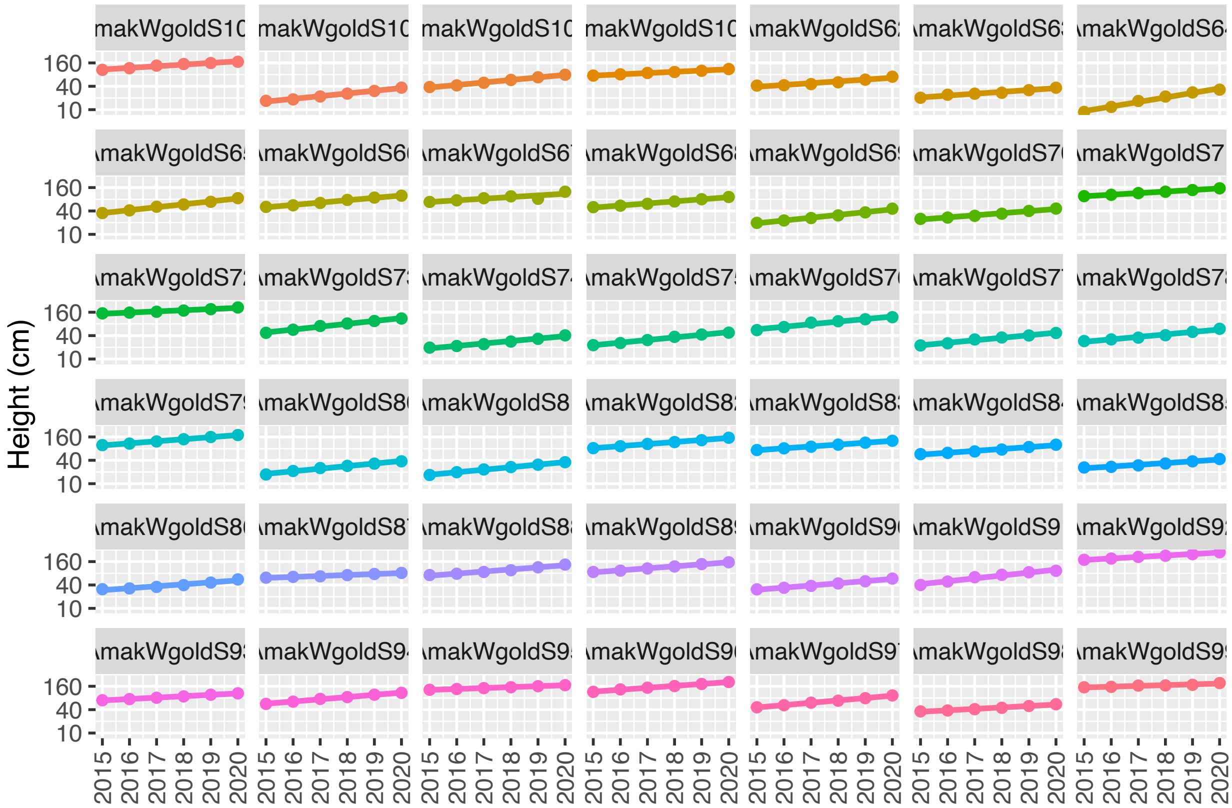

Growth curves for AmakW colonist juveniles.

```
## `geom_smooth()` using formula 'y ~ x'
```

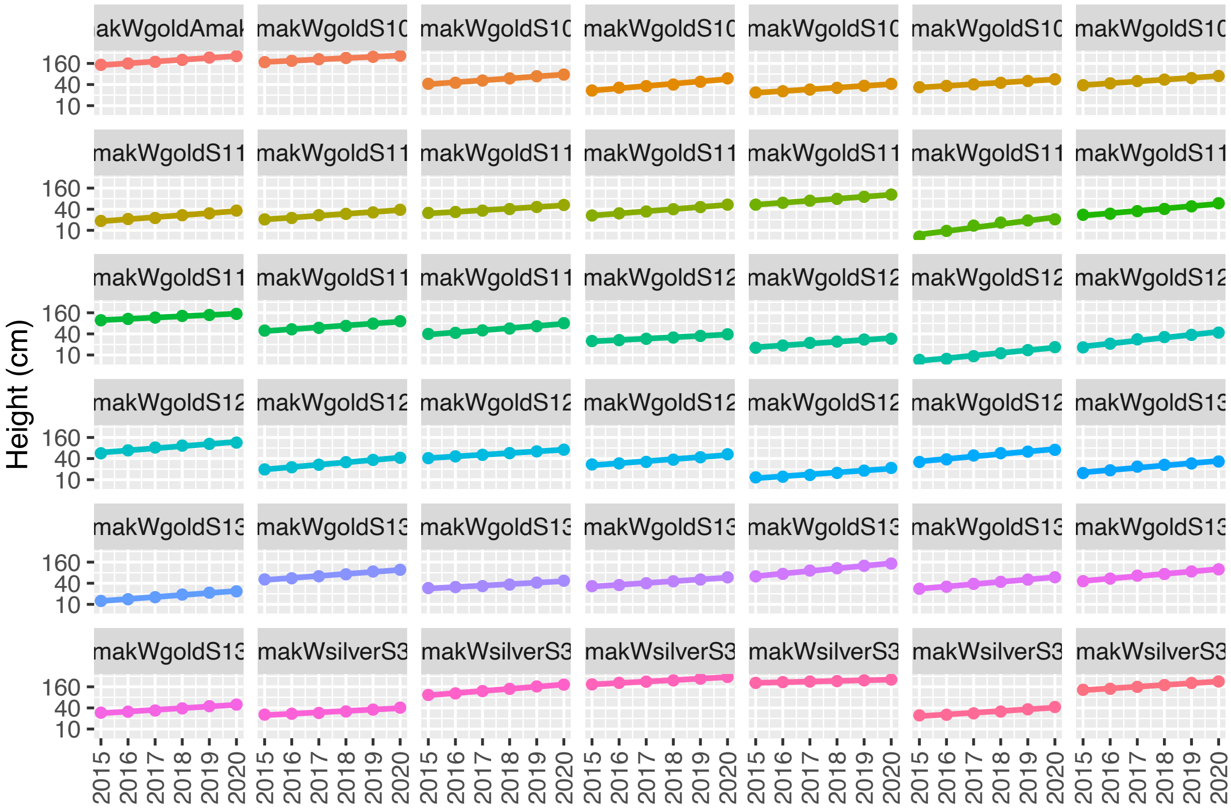

Growth curves for AmakW colonist juveniles.

```
## `geom_smooth()` using formula 'y ~ x'
```

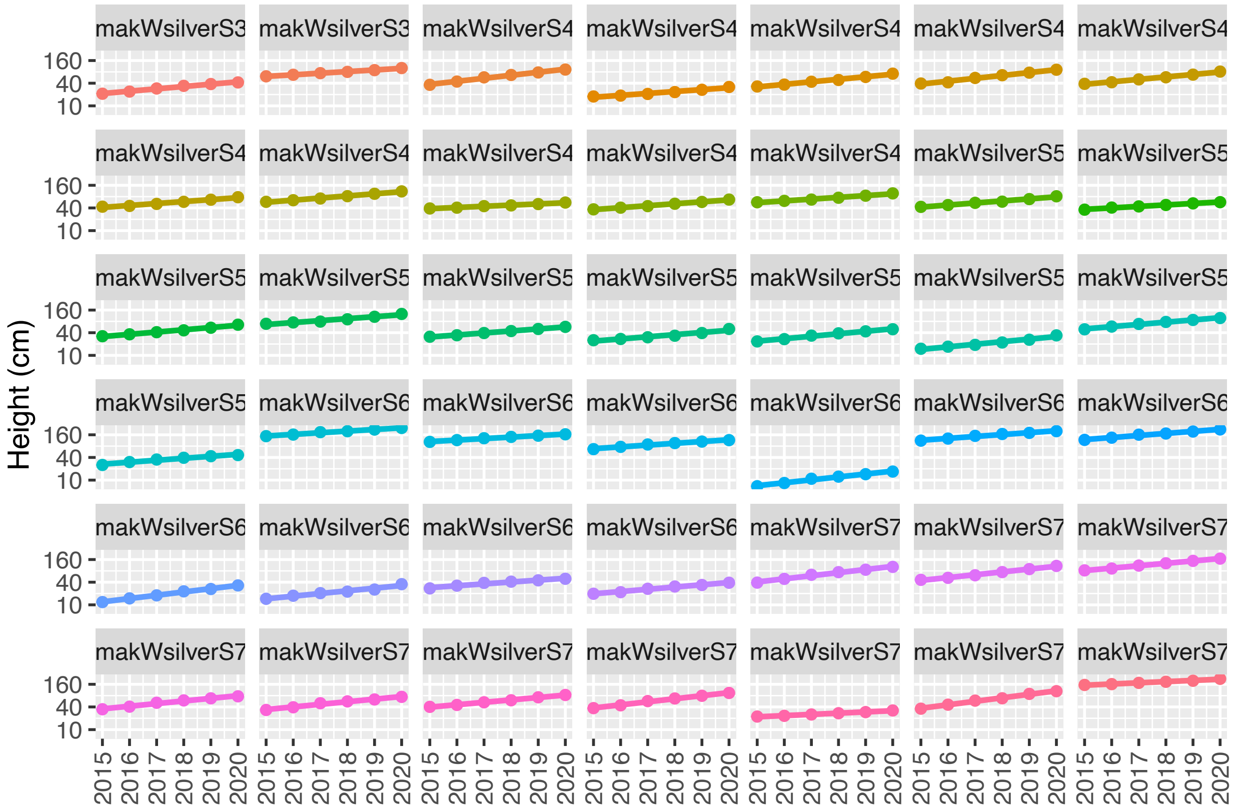

Growth curves for AmakW colonist juveniles.

```
## `geom_smooth()` using formula 'y ~ x'
```

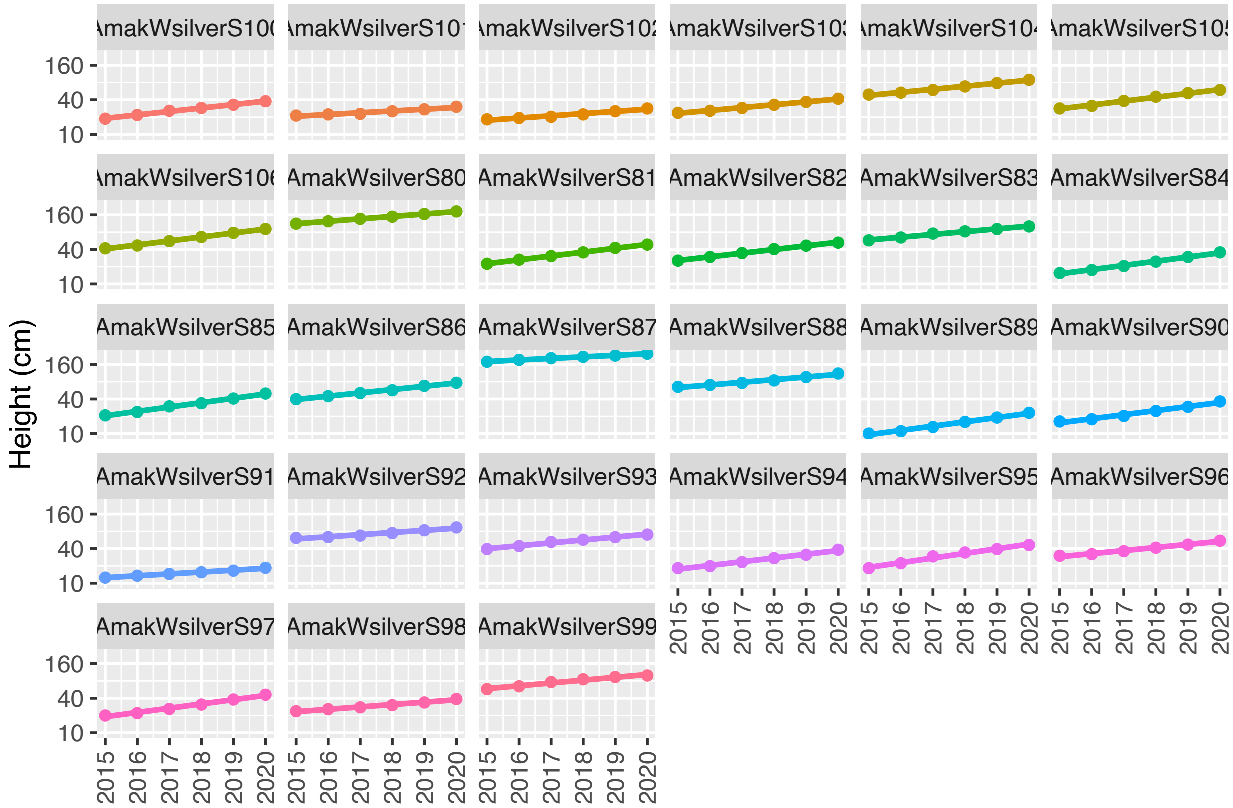

Growth curves for AmakW colonist juveniles.

3.5.2 271 individual growth curves for juveniles from established treelines and used for estimating RGR

```
BRTL <- read.csv("./BRTL JuvenileGrowthRaw.csv")
read.csv("./GrowthRates_BRTL.csv") %>%
    aggregate(data=.,
                        growth.rate~Field.Site,
                        FUN=length) %>%
    dplyr::mutate(Field.Site,
                juveniles=growth.rate,
                .keep="none") %>%
  kable(caption="Juvenile sample sizes by established treeline.")
```

Juvenile sample sizes by established treeline.

Field.Site	juveniles
AGL	6
AGR	10
ALL 3	5
AML3	15
AMR1	14
CLL	13
CLR	8
HFL	17

Field.Site	juveniles
HFR	12
KEL1	12
KER1	12
KUL1	12
KUR1	13
OML1	6
OMR1	11
TAL1	12
TAR1	12
TIL1	9
TIR1	12

The established treeline juveniles come from multiple field sites, all but one of which (Philip Smith) are BRTL sites. Seven are from the Noatak basin, eight from the Kobuk basin, and five from the Koyukuk basin. The Koyukuk was used because the Alatna basin abuts the Noatak basin and the uppermost named tributary of the Noatak, "Lucky Six Creek," has a population of colonists.

The BRTL sites come from established treelines that form upper (high elevation) and lower (low elevation) boundaries of forests on hillsides.

1. PhilipSmith
2. Kugururok high elevation "KUL1"
3. Kugururok low elevation "KUR1"
4. Kelly high elevation "KEL1"
5. Kelly low elevation "KER1"
6. Squirrel high elevation "TAL1"
7. Squirrel low elevation "TAR1"
8. Omar high elevation "OML1"
9. Omar low elevation "OMR1"
10. Timber high elevation "TIL1"
11. Timber low elevation "TIR1"
12. Ambler high elevation "AML3"
13. Ambler low elevation "AMR1"
14. Alatna high elevation "ALL 3"
15. Agashashok high elevation "AGL"
16. Agashashok low elevation "AGR"
17. John high elevation "HFL"
18. John low elevation "HFR"
19. Koyukuk high elevation "CLL"
20. Koyukuk low elevation "CLR"

PhilipSmith growth curves for 60 individuals.

```
## `geom_smooth()` using formula 'y ~ x'
```

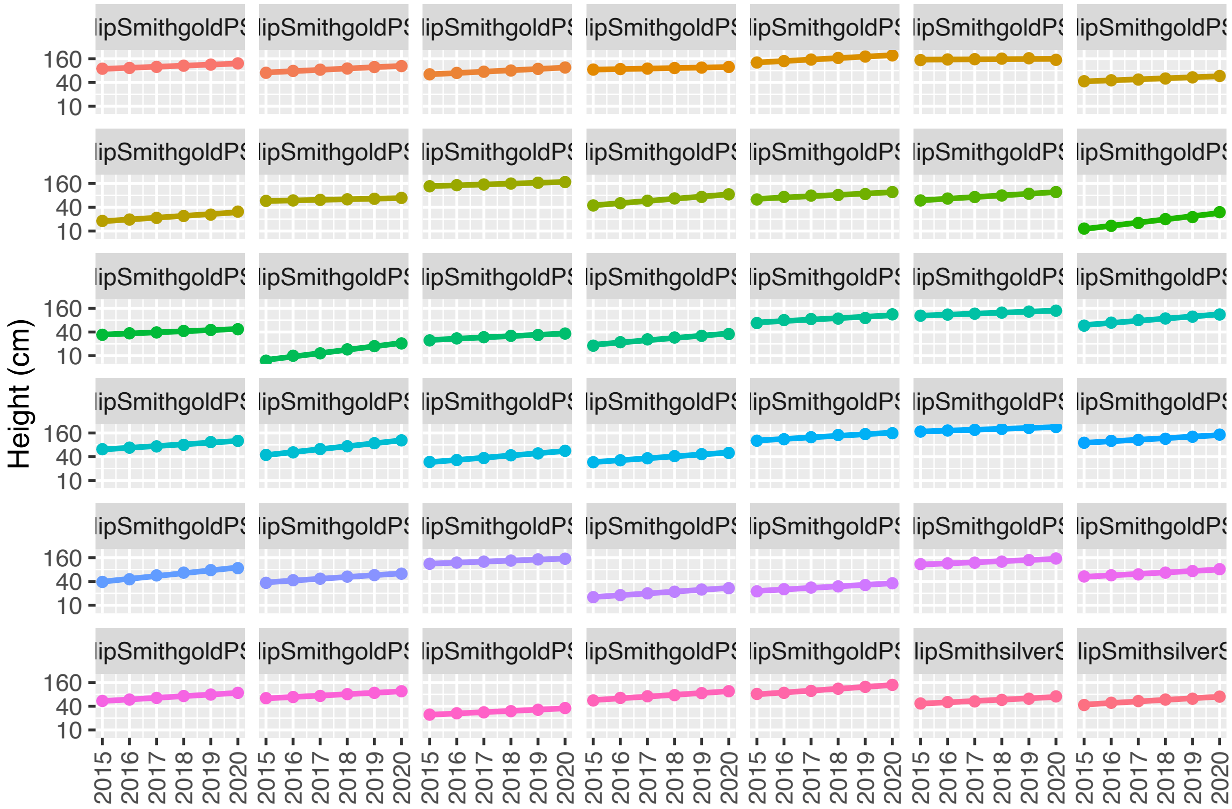

Growth curves for PhilipSmith established treeline juveniles.

```
## `geom_smooth()` using formula 'y ~ x'
```

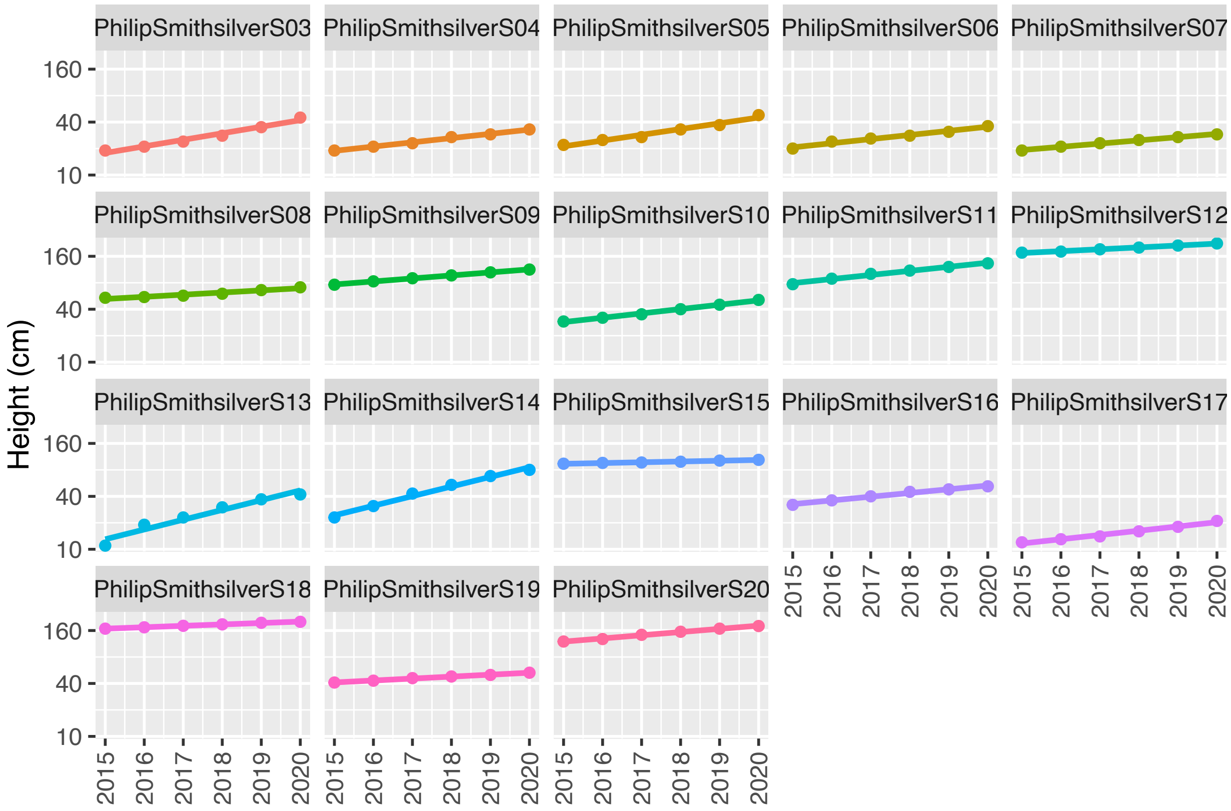

Growth curves for PhilipSmith established treeline juveniles.

Kugururok River high elevation "KUL1" 12 samples; low elevation "KUR1" has 13 samples.

```
## `geom_smooth()` using formula 'y ~ x'
```

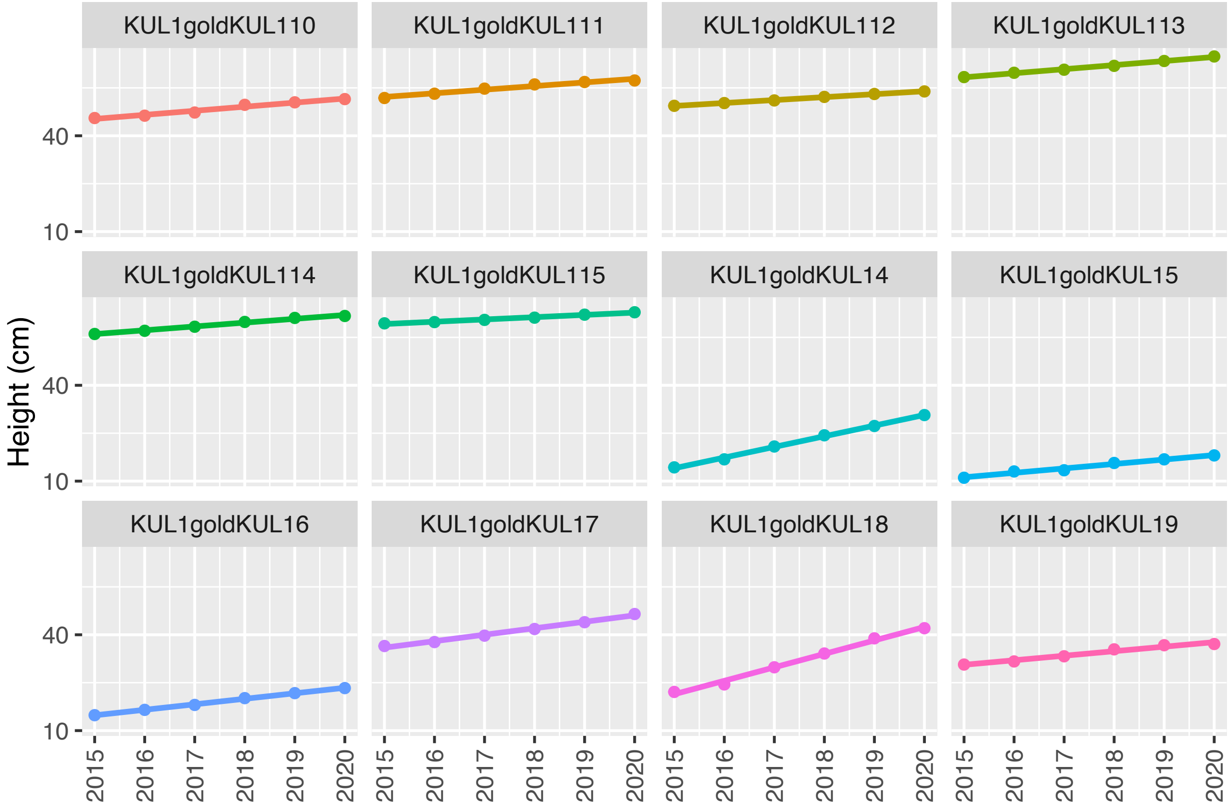

Growth curves for Kugururok established treeline juveniles.

Kelly River high elevation "KEL1" has 12 samples; low elevation "KER1" has 12 samples

```
## `geom_smooth()` using formula 'y ~ x'
```

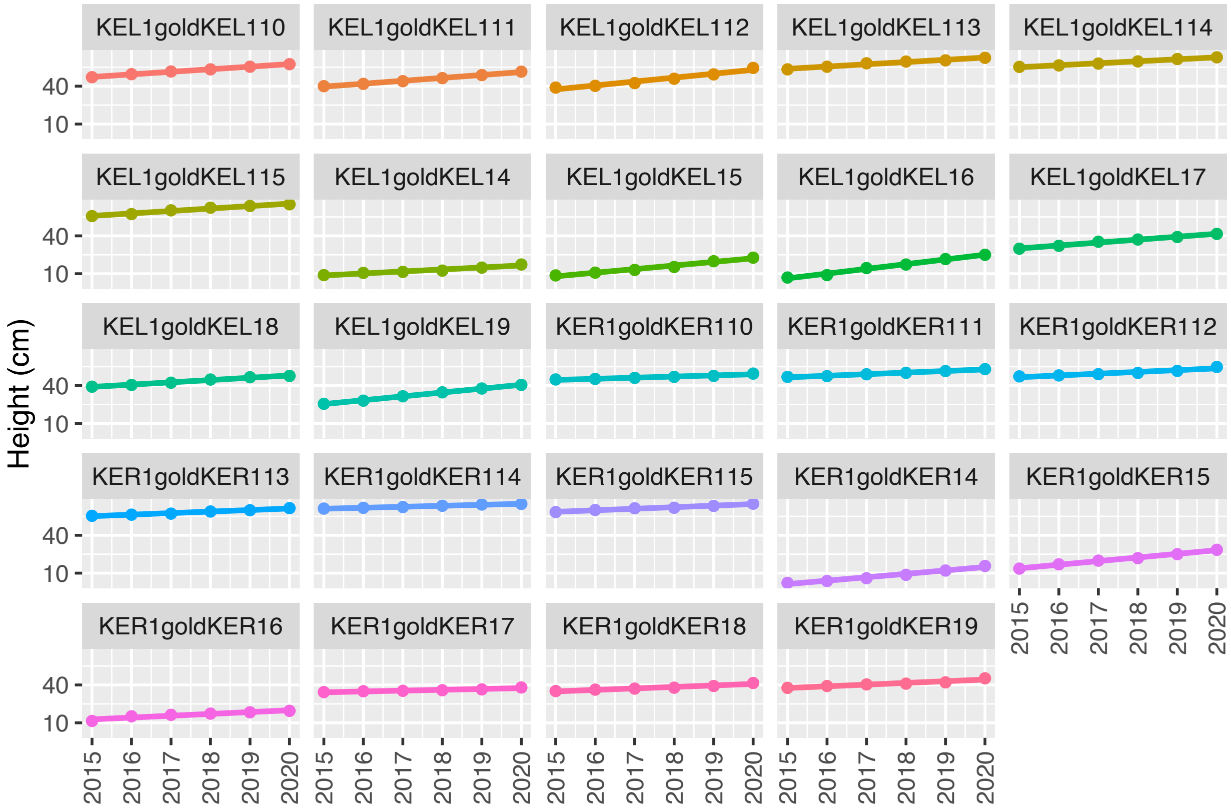

Growth curves for Kelly established treeline juveniles.

Squirrel River high elevation "TAL1" and low elevation "TAR1" have 12 samples each.

```
## `geom_smooth()` using formula 'y ~ x'
```

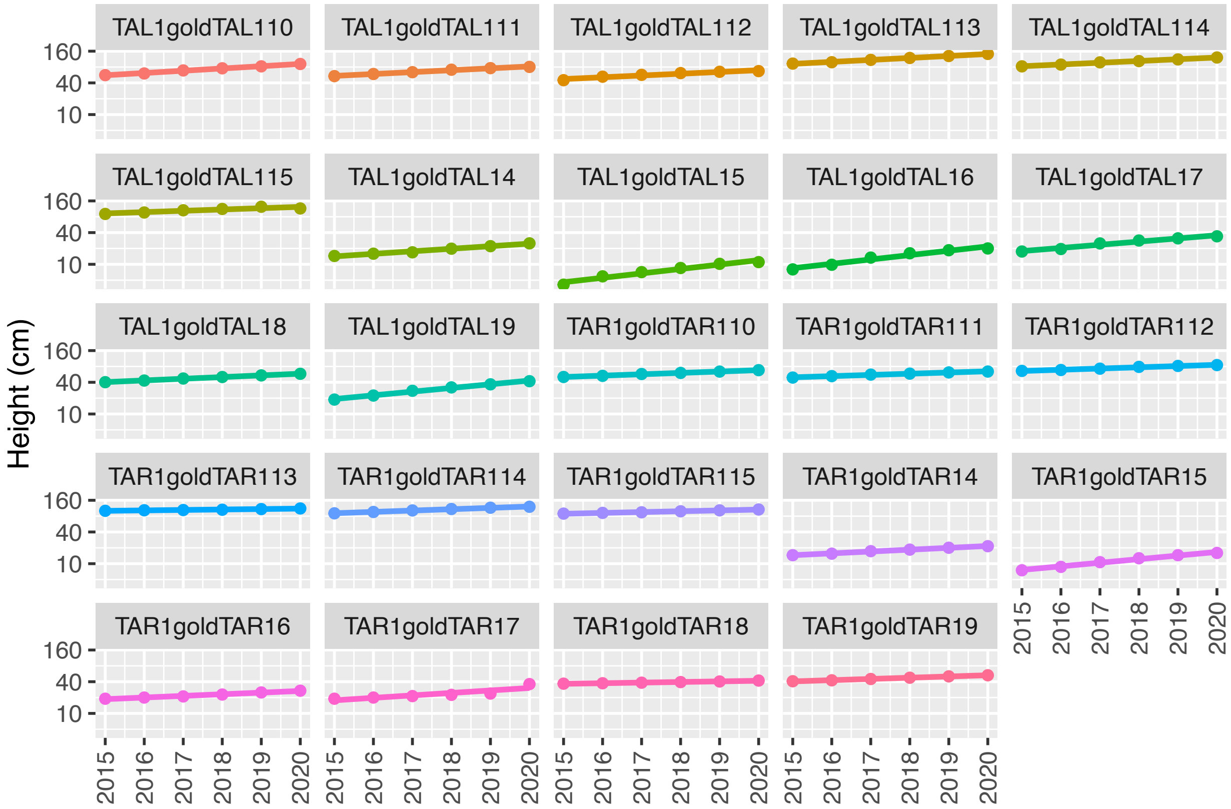

Growth curves for Squirrel established treeline juveniles.

Omar River high elevation "OML1" has 6 samples; low elevation "OMR1" has 11 samples.

```
## `geom_smooth()` using formula 'y ~ x'
```

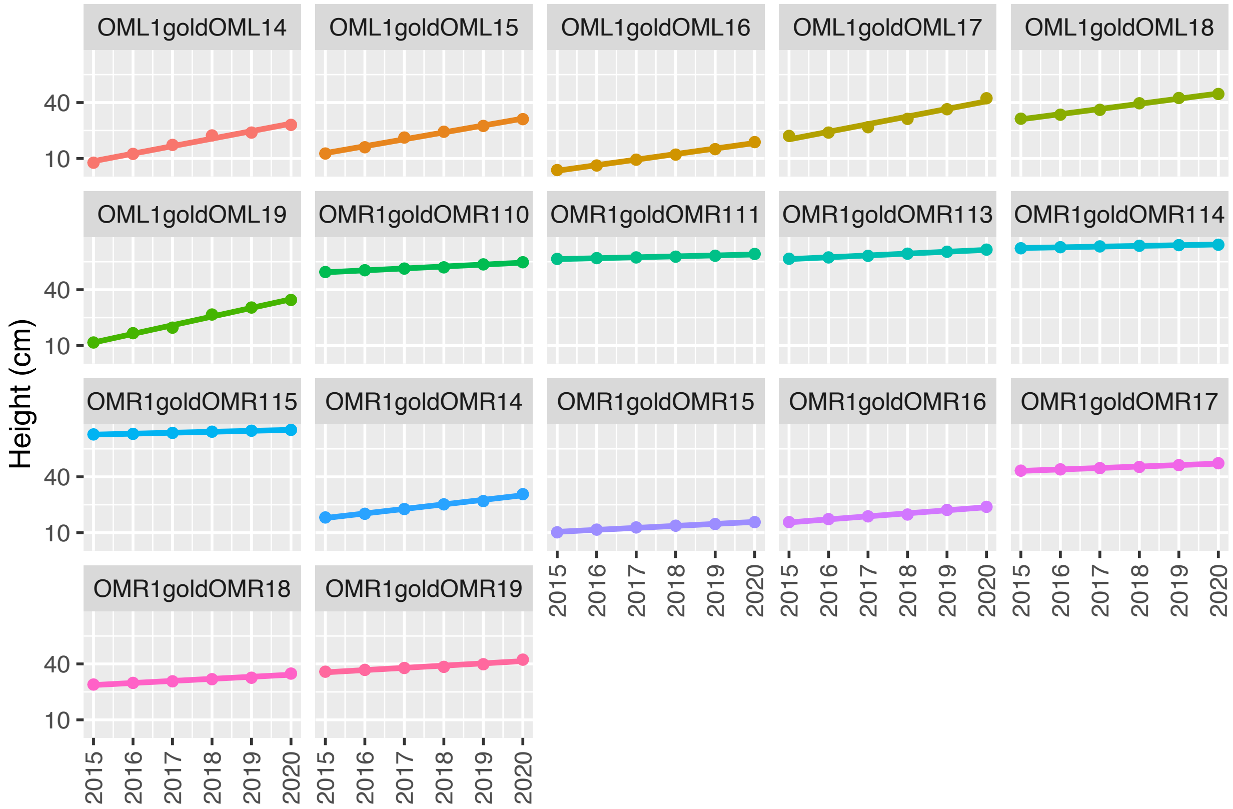

Growth curves for Omar established treeline juveniles.

Timber Creek high elevation "TIL1" has 12 samples; low elevation "TIR1" has 12 samples.

```
## `geom_smooth()` using formula 'y ~ x'
```

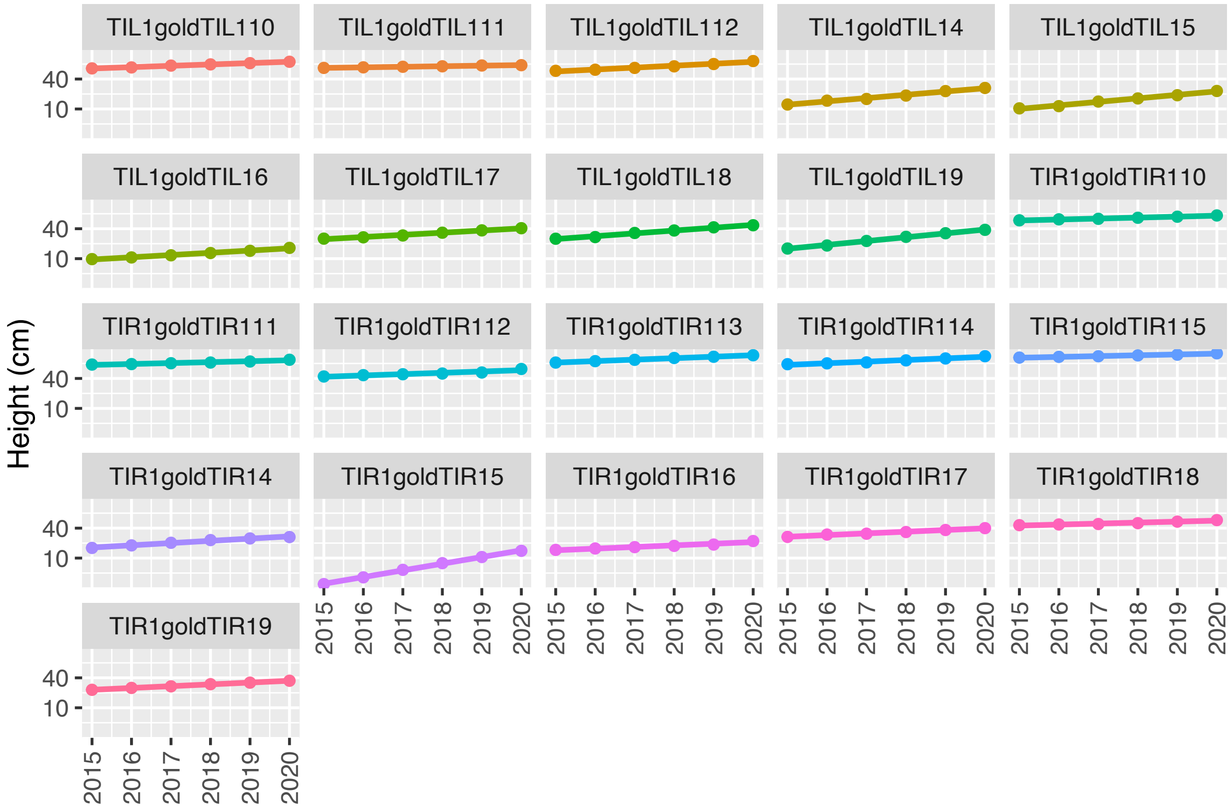

Growth curves for Timber Creek established treeline juveniles.

Ambler River high elevation “AML1” has 15 samples; low elevation “AMR1” has 14 samples.

```
## `geom_smooth()` using formula 'y ~ x'
```

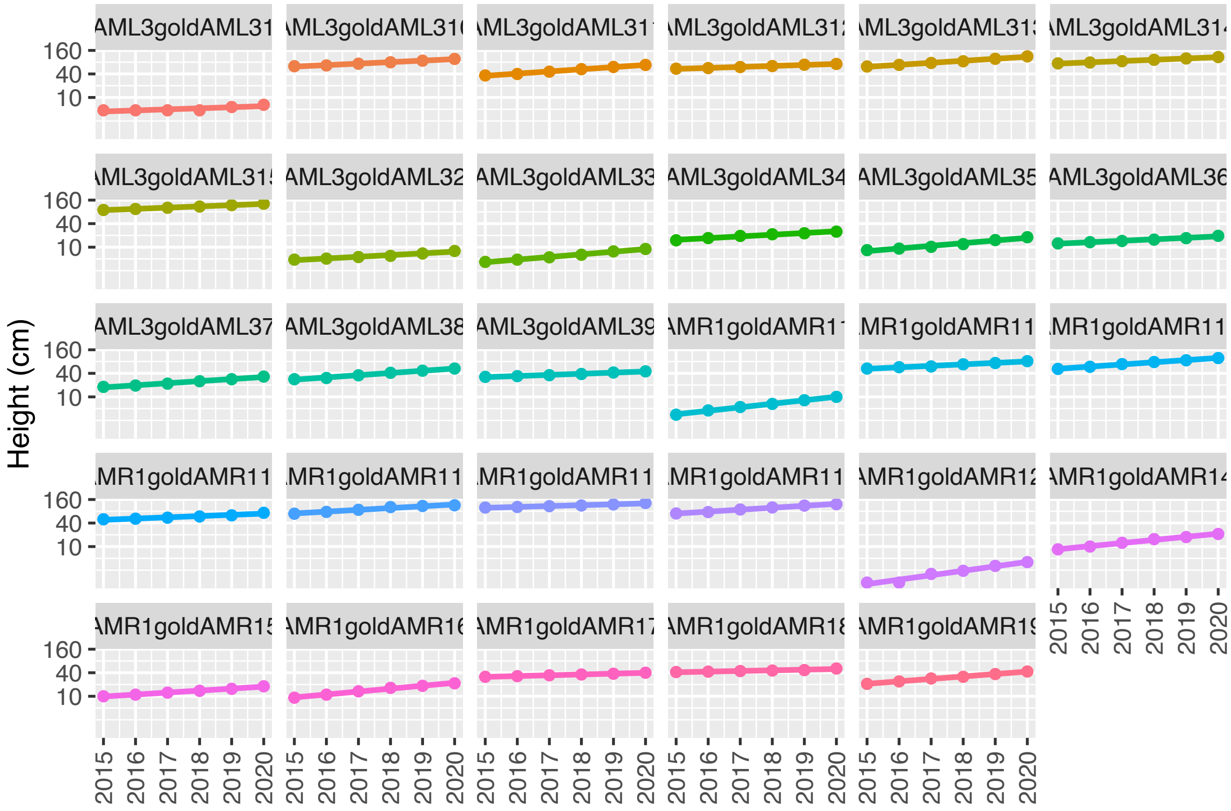

Growth curves for Timber Creek established treeline juveniles.

Alatna high elevation "ALL3" has 5 samples.

```
## `geom_smooth()` using formula 'y ~ x'
```

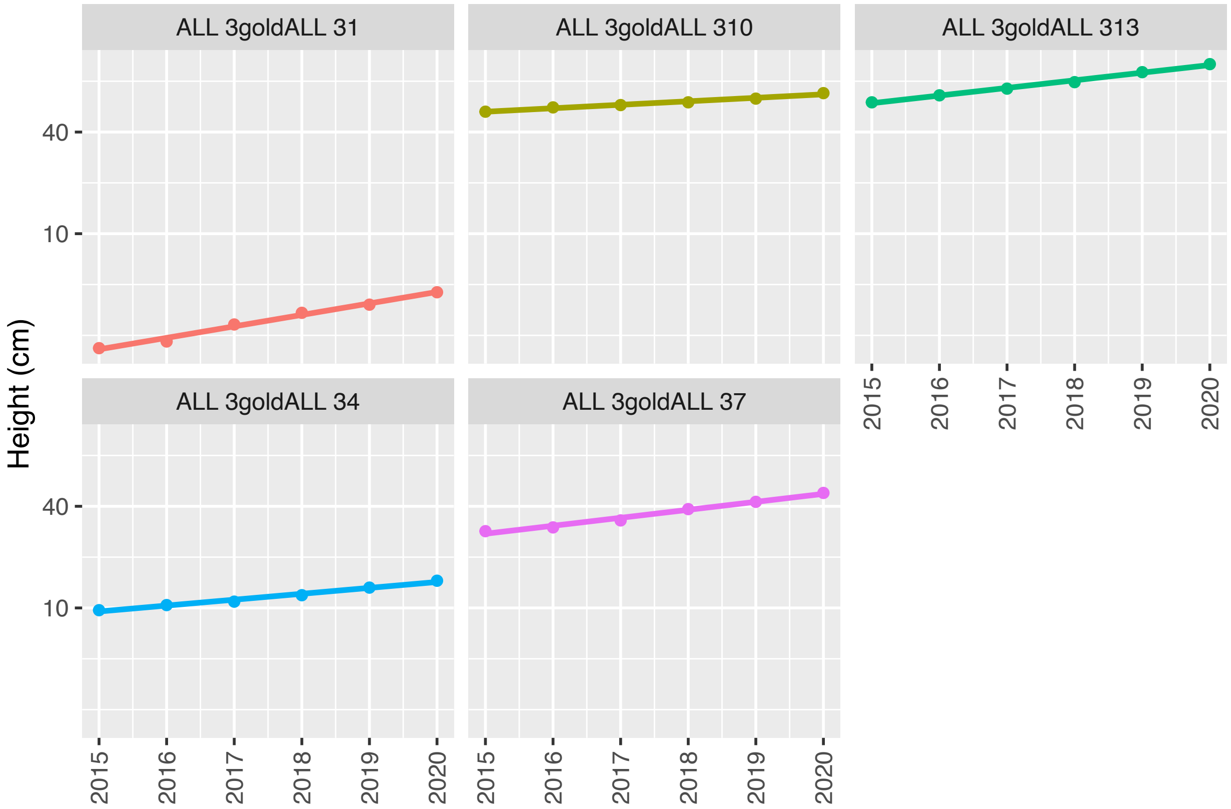

Growth curves for Alatna established treeline juveniles.

Agashashok has 6 high elevation “AGL” and 10 low elevation “AGR” samples.

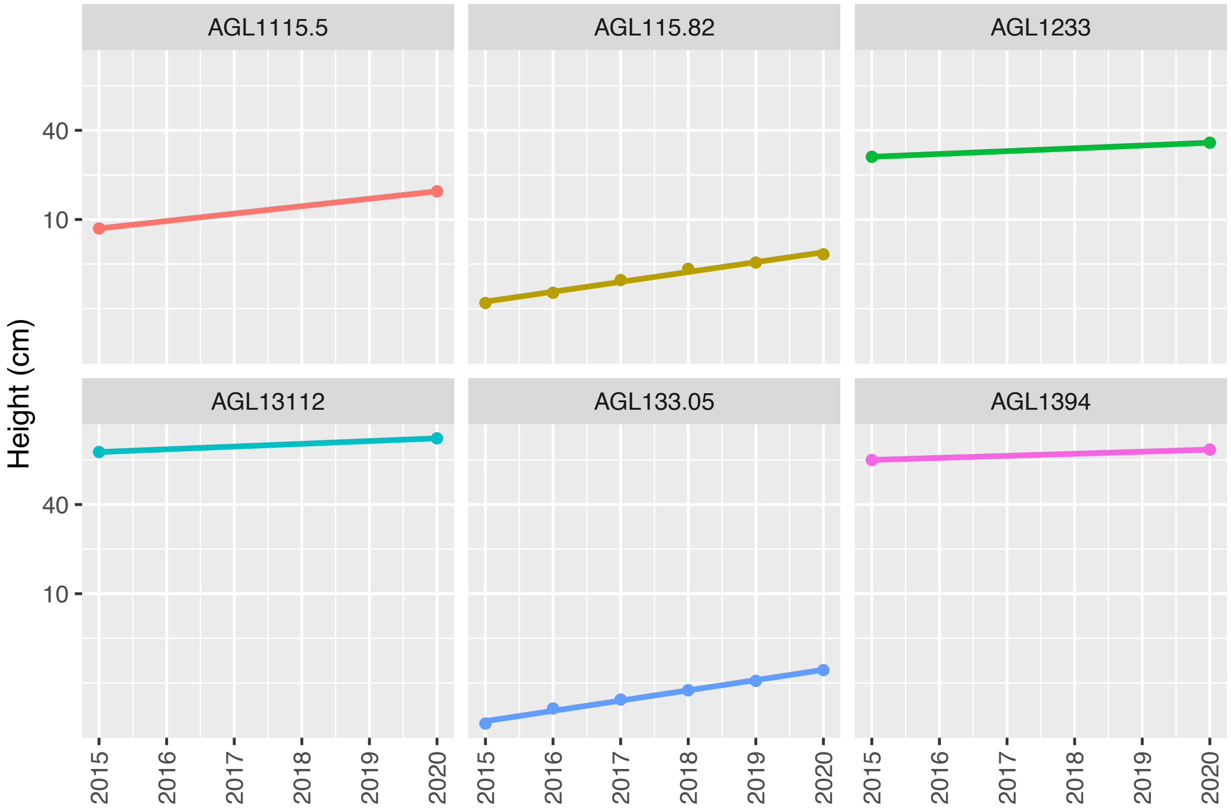

Growth curves for Agashashok established treeline juveniles.

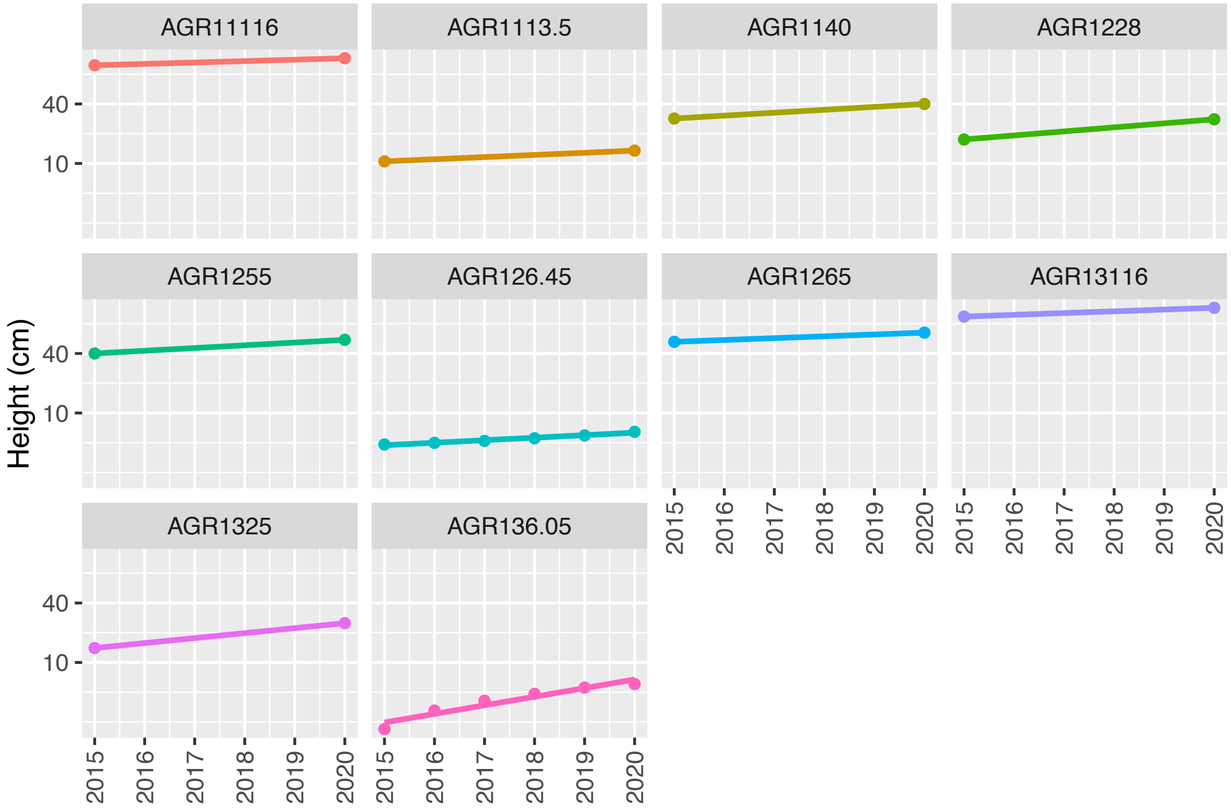

Growth curves for Agashashok established treeline juveniles.

John has 17 high elevation "HFL" and 12 low elevation "HFR" samples.

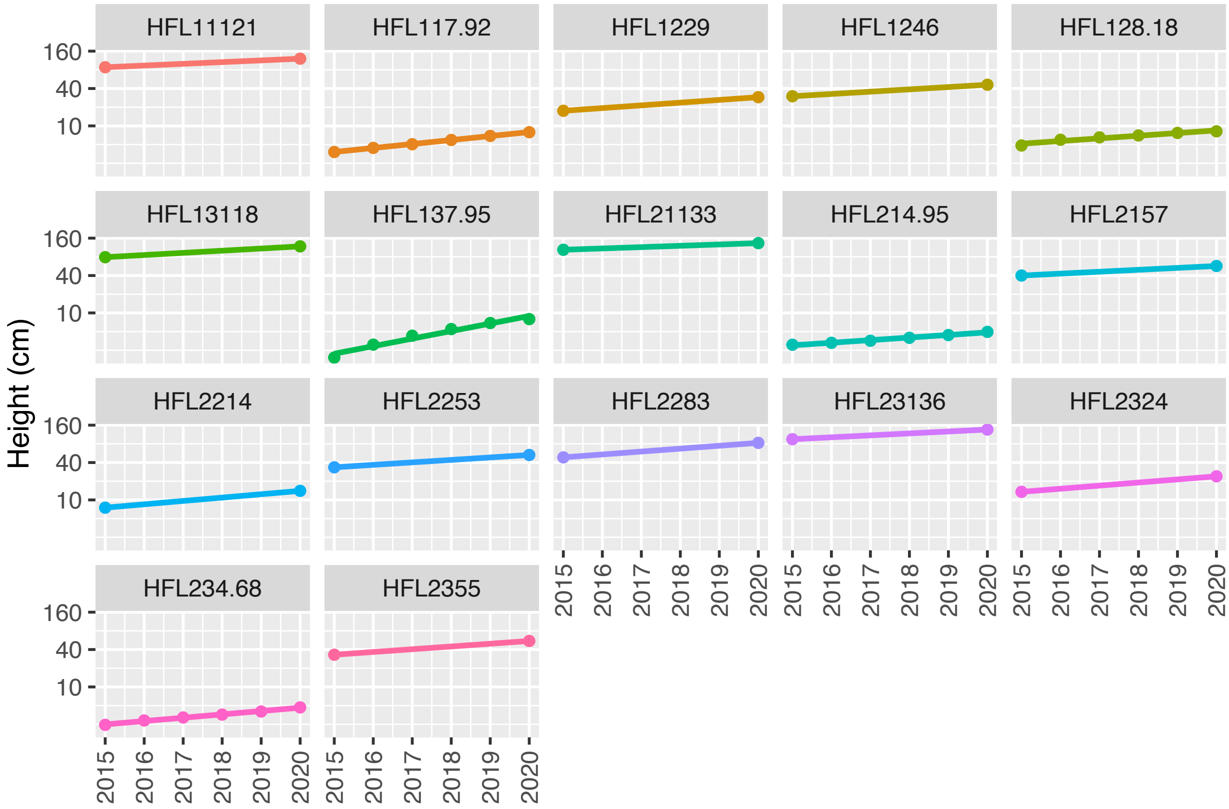

Growth curves for John established treeline juveniles.

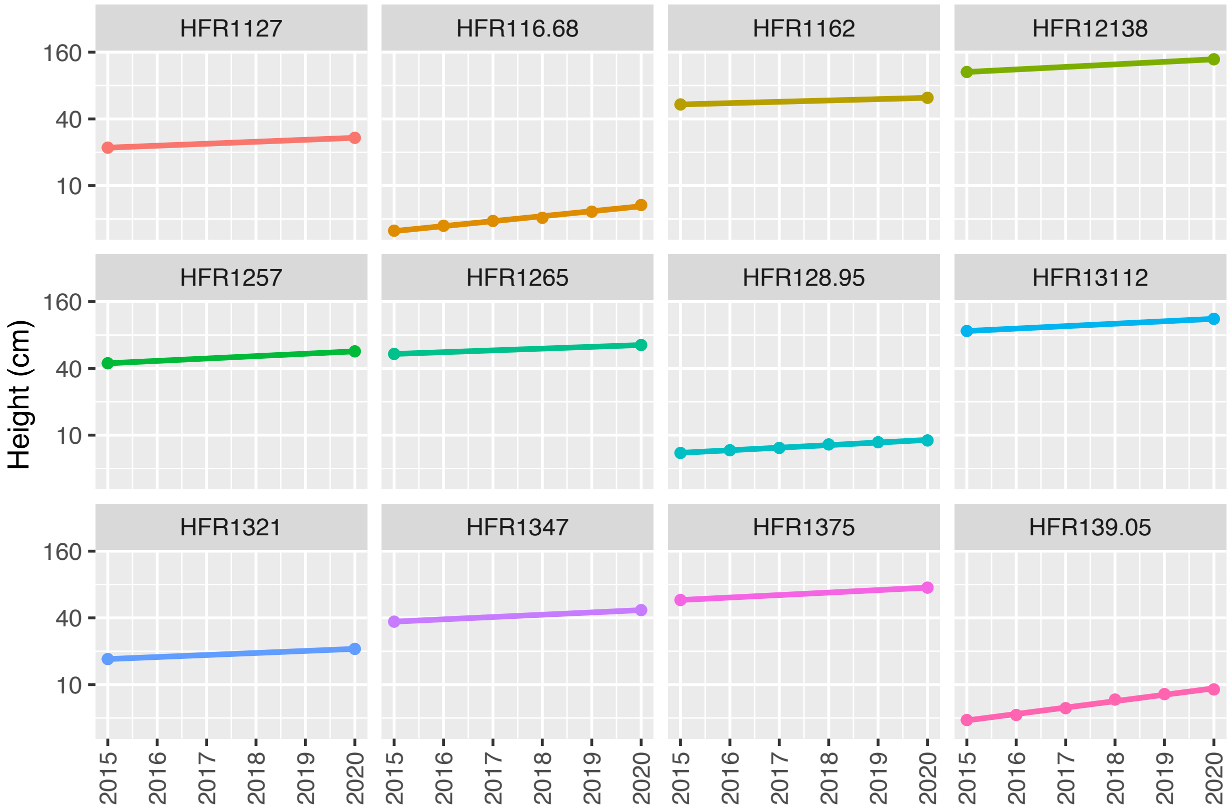

Growth curves for John established treeline juveniles.

Koyukuk has 13 high elevation "CLL" and 8 low elevation "CLR" samples.

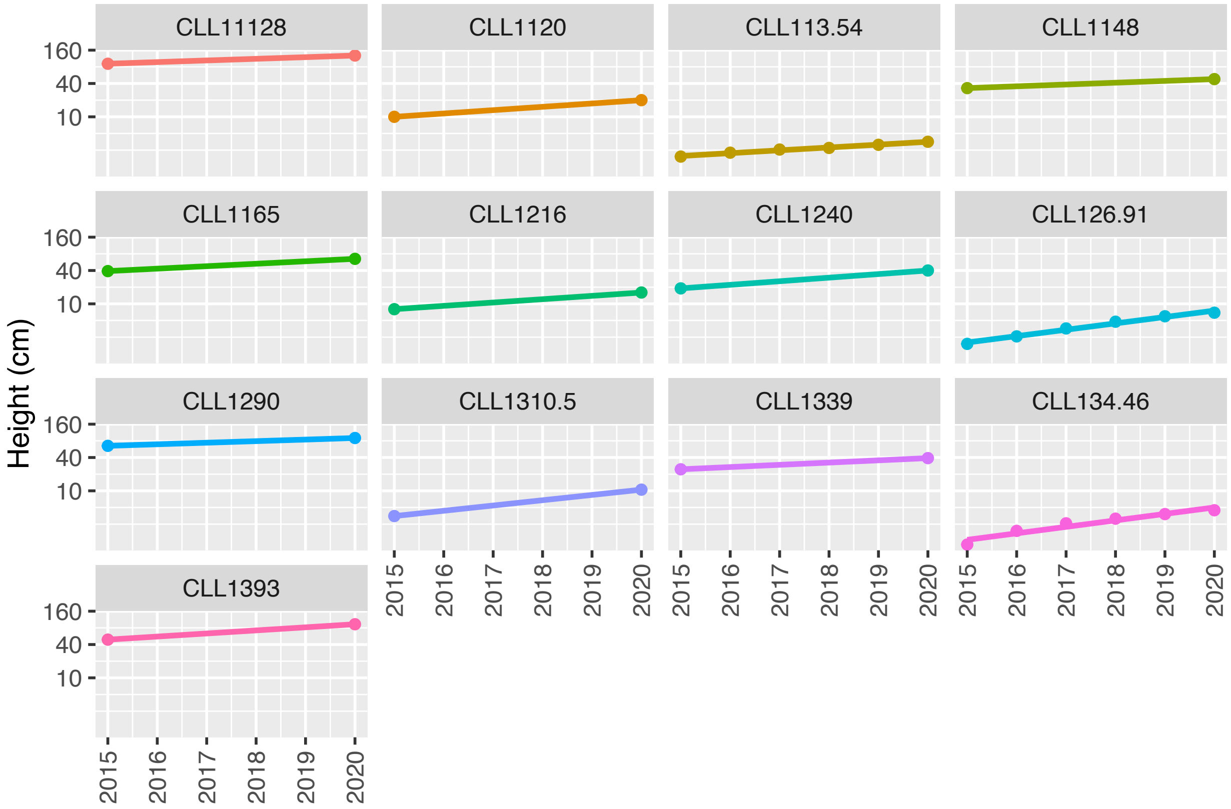

Growth curves for Koyukuk established treeline juveniles.

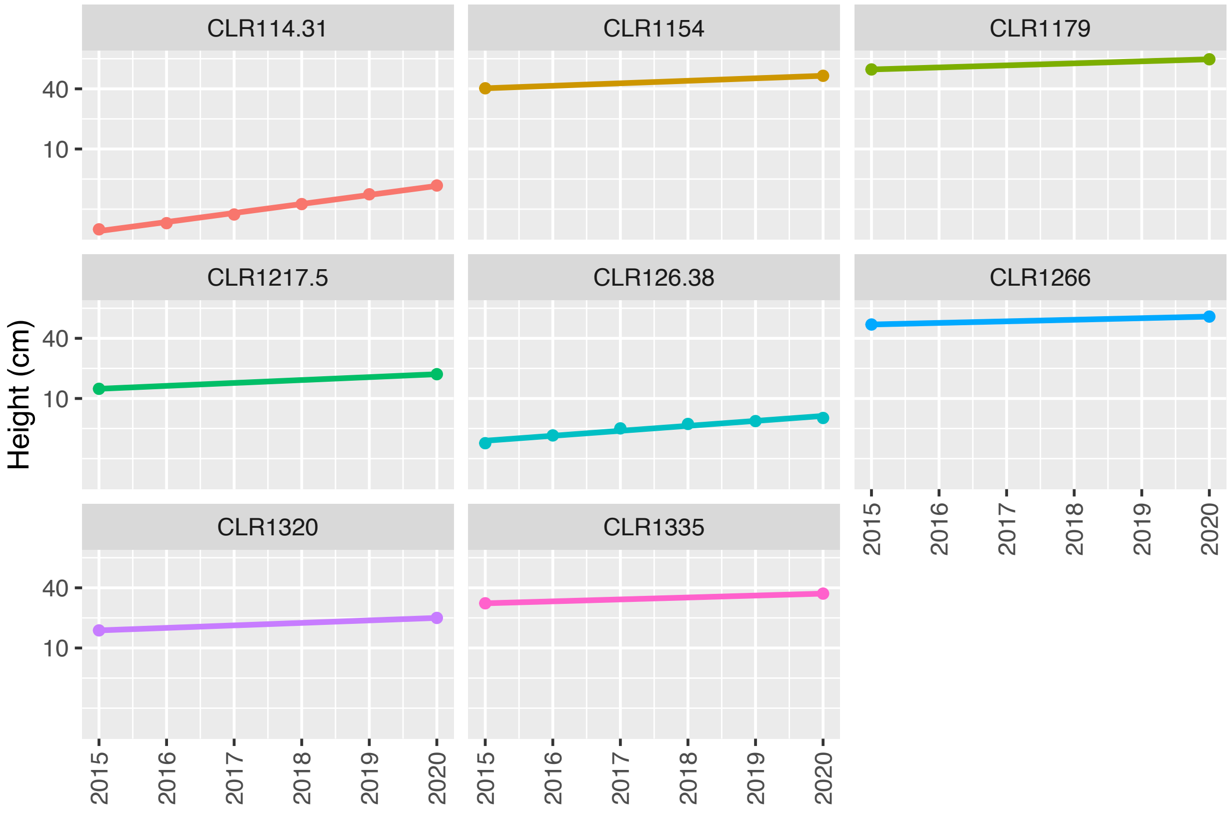

Growth curves for Koyukuk established treeline juveniles.

4 Reconstructing Population Growth

Using 1,971 shadows, we reconstructed population size from the years 1900 to 1980 in an area of 136 km^2 that includes two sub-watersheds of the Cutler River basin in the AOI. The area of reconstruction is labeled with "Simulated population area" as a central black rectangle in ED Fig. 1 andas a red rectangle in **SI Figs. 1-4**. We began with year 1900 because that was near the year estimated for establishment of oldest aged tree in the sub-watershed. We ended in 1980 because trees younger than about 40 years were not reliably visible on the super-overlay imagery: they were too short to cast detectable shadows given the deep snow of 2018 (see **SI Calc. 4.4.** below).

As a basis for simulation, we used 5,988 digitized shadows visible on snow-covered scenes (ED Fig. 1b, **SI Fig. 1**) of DigitalGlobe WV imagery (ED Table 1). We identified a sample of shadows cast by spruce and stratified by length that we located in the field with GNSS, then increment cored basally and/or measured for height.

To reconstruct the population by year, we began by constructing a linear mixed model of tree height on digitized shadow length. Then we constructed non-parametric, kernel density probability distributions of establishment-year by height-class based on aged trees and their heights measured in the field.

Given the linear model for heights on shadow length, we calculated for each of the digitized shadows a height in meters, then assigned it to one of five height-classes. We did this in a Monte Carlo framework during the simulations using the covariance matrix of random effects for the intercept and regression coefficient estimating tree height based on shadow length. Height classes deduced from the shadow measurements were in some cases only 0.5 m in width. Although, as we show below, the intercept varied by about 0.4 m across the six random study areas, the snow depth at any given tree most certainly differed by greater

than 0.4 m from elsewhere and that motivated our application of Monte Carlo sampling of the covariance matrix: to capture the among-site variability in snow depth as intercept and the relative sun elevation angle, whose tangent was the slope (regression coefficient). As a sensitivity test for the approach, we also provide results of a simulation using three height classes with interval width of 1.3 m which captured in some degree the within-site variability in snow depths and the relative sun elevation angles (*i.e.,* the direction uphill or downhill combined with absolute sun elevation angle).

Given heights estimated from the 1,971 shadows, we determined establishment year for each shadow by randomly sampling with replacement (bootstrapping) from the kernel density distribution of establishment-years associated with the height-class of the tree casting the shadow. Because these trees all survived to cast shadows in 2018, we tallied the count of trees establishing each year as recruitment for that year. We then cumulatively summed recruitment by year to calculate population size by year.

This flow of calculations, beginning with sampling from the covariance matrix to estimate each height based on shadow length, then drawing from the non-parametric distributions of ages-given-height class to estimate year of establishment, and finally cumulatively summing by year, was repeated 1,000 times. Each run of the simulation applied non-linear least squares (**nls()** function of base R) to fit an exponential growth model. The exponential growth rate from each simulation was saved and the exponential curve using the average of the 1,000 simulations is shown in main text Fig. 3a.

To recap, the population reconstructions shown below use digitized shadows, measured heights, and years of establishment of increment-cored trees. Reconstructing the population for each year relies on the following relationships:

1. Height and its relationship with shadow length by incorporating variability across the landscape.
2. Year of establishment, t, and its relationship with height as non-parametric probability distribution.
3. Total number of trees establishing in year t and its relationship with recruitment to the population visible in 2018 ignoring mortality.
4. Cumulative sum of recruits and its relationship with population size.

The relationships rely on the following three data streams:

1. the digitized lengths of $n = 1,971$ spruce shadows cast on the 26 March 2018 scene from WV-1 panchromatic imagery.

```
read.csv("./BestShadowData.csv") %>%
    subset(sun.elevation.deg < 20 &
    Shadow_m <= 14.2 &
    lat > 67.536 &
    long < -157.8) %>%
 dplyr::mutate(Shadow_m,
    .keep="none") %>%

nrow() %>% format(big.mark=",") %>% paste("shadows")
```

```
## [1] "1,971 shadows"
```

2. The establishment-year for 124 aged trees that are > 3m tall (of the 150 total). The choice of 3 m as a cut-off is because shorter trees were more difficult to see as shadows due to snow depth and their short, ambiguous, or absent shadow length.

```
dat.r <- read.csv("./AgingIncrementCoreHeight V3.csv") %>%
   subset(Height_m > 3)

nrow(dat.r) %>% paste("trees with establishment year")
```

```
## [1] "124 trees with establishment year"
```

3. The heights of $n = 260$ adult colonists matched to their shadows in six areas from three watersheds.

```r
shadows.and.heights <- read.csv("./Shadows and Heights.csv") %>%
    subset(!is.na(Shadow.m))

str(shadows.and.heights)
```

```
## 'data.frame':    260 obs. of  5 variables:
##  $ ID      : chr  "Amak 27" "S14" "Amak 43 cutler giant" "Amak 52" ...
##  $ Height.m: num  5.5 4.6 10.7 7.7 8.5 ...
##  $ Shadow.m: num  7.1 10.2 34.3 33.2 29.6 ...
##  $ Where.3 : chr  "AmakE" "AmakW" "AmakW" "AmakW" ...
##  $ Shadow  : chr  "yes" "yes" "yes" "yes" ...
```

4.1 Modeling Height by Shadow Length

Given the heights and shadow lengths, we used linear mixed effects models with study area (the six study sites) of field sampling as a random factor to capture both the variability of terrain slope-angle and of snow-depth to predict height of spruce as a function of shadow length.

On a flat landscape covered uniformly in snow, the height of each tree would equal snow depth added to the product of shadow length with the tangent of sun elevation angle. However, because both the relative sun elevation angle casting shadows on surface snow and snow depth vary with terrain, we used a linear mixed effects model of height on shadow length, interpreting the intercept as average snow depth and the inverse tangent of the regression coefficient as average sun elevation angle relative to the terrain slope. We then applied the fixed effects as coefficients in a linear model to estimate height from shadow for 1,971 shadows in the sub-watershed study area but included variability in the coefficients by drawing from the random effects covariance matrix in a Monte Carlo approach.

We measured heights from trees matched with shadows during three field campaigns:

```r
Cutler19 <- read.csv("./September2019TreesWithHeights.csv") %>%
    dplyr::mutate(ID=ID,
      lat=lat,
       lon=long,
       Height.m,
       Shadow.m,
       Status,
       Where.3,
       sample.year,
       .keep="none")

Cutler20 <- read.csv("./September2020TreesWithHeights.csv") %>%
    dplyr::mutate(ID=ID,
       lat=lat,
       lon=lon,
       Height.m,
       Shadow.m,
       Status,
       Where.3,
       sample.year=2020,
       .keep="none")

Amak21 <- read.csv("./July2021TreesWithHeights.csv") %>%
    subset(ID != "Amak 35 Massive cone Dwarf") %>%
    subset(Sunlight.presence != "absent") %>%
```

```
    dplyr::mutate(ID,
       Height.m,
       lat=lat,
       lon=long,
       Shadow.m=shadow_length,
       Status,
       Where.3=ifelse(long < -157.4,
            "AmakW", "AmakE"),
       sample.year=2021,
       .keep="none")

dat <- rbind(Amak21,Cutler20,Cutler19) %>%
   dplyr::mutate(Where.3=
        ifelse(Where.3 %in% c("CutlerHillside",
            "CutlerValley","UpperCutlerValley"),
            "Cutler Main",Where.3),
     Shadow=ifelse(is.na(Shadow.m),"no","yes"))

dat.L <- subset(dat,Status=="live") %>%
    dplyr::mutate(ID,
    lat,
    lon,
    Height.m,
    Shadow.m,
    Study.area=Where.3,
    Shadow,
    .keep="none")

    dat.L$Study.area[which(is.na(dat.L$Study.area))] <- "Cutler Main"

    dat.L$Study.area <- factor(dat.L$Study.area,
            levels=c("Cutler Main","AmakE",                                    "BuffaloD
                 "GaiaHill","BobWoods"))
```

4.1.1 What tree heights cast no shadows?

The only direct measurement of snow depth was a snow-pit "8-10 feet deep" (2.4-3 m) dug in one location in late March 2018 by pilot Eric Sieh of Kotzebue, Alaska. Here we use measured tree heights to further estimate snow depth. By portraying the heights of trees casting and not casting shadows across the six study areas in the AOI as violin plots, it is possible to visualize the variability in snow depth with a reference snow depth as 2.4-3 m (the snow pit depth) in tree height.

```
dat.L %>%
    ggplot(aes(y=Height.m,x=Study.area,fill=Shadow)) +
    geom_violin() +
    labs(x="Landscapes",
             y="Field height (m)") +
    geom_hline(yintercept=c(2.4,3)) +
    scale_y_continuous(breaks=0:10)
```

In the violin plots for study areas "Cutler Main", "AmakE", "AmakW", and "GaiaHill" there is a degree of matching between the maximum height of trees that fail to cast shadows ("Max_fail") and the minimum height of trees that do cast shadows ("Min_cast"). If Max_fail = Min_cast, then that would be evidence of

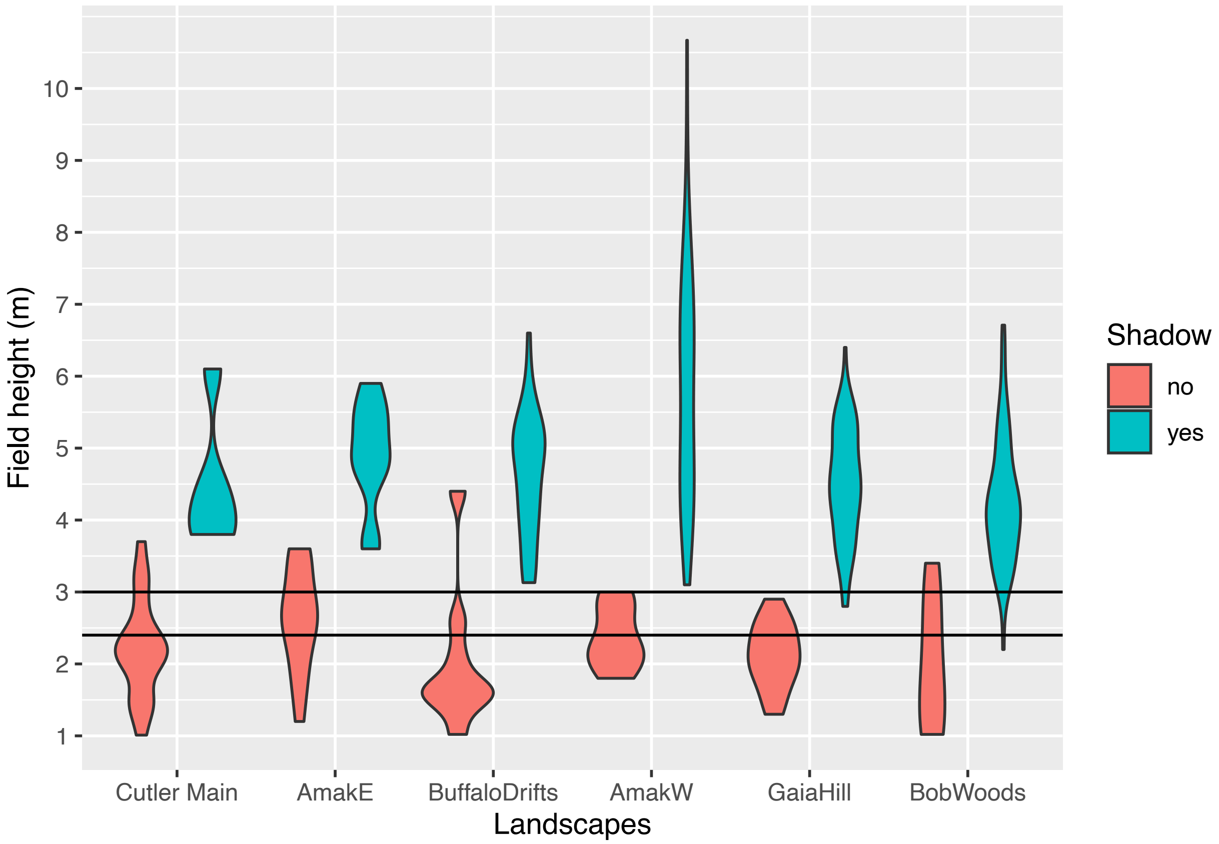

Distributions as violin plots of field measured height of trees with (blue) and without (red) shadows seen on World-View snow-covered satellite scenes. Landscapes give name of field sites. Horizontal lines give 3 m height and 2.4 m height of trees, reflecting the estimated depth of a single snow pit dug in the population reconstruction area in late March 2018.

snow depth uniformity.

The Max_fail and Min_cast values of "BuffaloDrifts" and "BobWoods" differ most. For both study areas, the overlap of violin plots of shadow casting trees with non-shadow casting suggests variability in snow depth. These two study sites have the most variable topography, marked by parallel ridgelines and other surface features perpendicular to the prevailing winter winds (**SI Fig. 11**). In contrast, "Cutler Main" (**SI Fig. 13**), "AmakE", "AmakW" (**SI Fig. 10**) and "GaiaHill" suggest more uniform snow depths, because each study site displays violin plots with Max_fail $\approx$ Min_cast.

These visual comparisons can be made more rigorous by considering the heights at each study site. First, in each area find the number of trees measured for height that cast shadows:

```
ag.count.yes <- aggregate(Height.m~Study.area,
   FUN=length,
   data=dat.L,
   subset=Shadow=="yes")
```

Next, aggregate over each study site the heights of trees casting shadows by finding the shortest height in each area casting a shadow (*i.e.*, "Min_cast" defined above). Also append the number of measured trees casting shadows.

```
shadows.yes <- aggregate(Height.m~Study.area,
       FUN=min,
       data=dat.L,
       subset=Shadow=="yes") %>%
  dplyr::mutate(Study.area,
       Min_cast=round(Height.m,1),
       n.trees=ag.count.yes$Height.m,
       .keep="none") %>%
                arrange(Min_cast)

shadows.yes %>%
  kable(caption="Sample size (n.trees) of
        trees casting shadows by study area with minimum height (Min_cast) in meters.")
```

Sample size (n.trees) of trees casting shadows by study area with minimum height (Min_cast) in meters.

Study.area	Min_cast	n.trees
BobWoods	2.2	65
GaiaHill	2.8	72
BuffaloDrifts	3.1	33
AmakW	3.1	69
AmakE	3.6	16
Cutler Main	3.8	5

Perform a similar analysis for trees measured for height but not casting shadows by identifying the maximum heights (*i.e.*, "Max_fail" defined above).

```
ag.count.no <- aggregate(Height.m~Study.area,
    FUN=length,
    data=dat.L,
    subset=Shadow=="no")
shadows.no <- aggregate(Height.m~Study.area,
     FUN=max,
     data=dat.L,
```

```
      subset=Shadow=="no") %>%
    dplyr::mutate(Study.area,
      Max_fail=Height.m,
      n.trees=ag.count.no$Height.m,
      .keep="none") %>%
                arrange(Max_fail)

shadows.no %>%
  kable(caption="Sample size (n.trees) of trees without shadows by study area
        with maximum height (Max_fail) in meters.")
```

Sample size (n.trees) of trees without shadows by study area with maximum height (Max_fail) in meters.

Study.area	Max_fail	n.trees
GaiaHill	2.9	14
AmakW	3.0	8
BobWoods	3.4	7
AmakE	3.6	14
Cutler Main	3.7	21
BuffaloDrifts	4.4	9

Finally, checking for variability between minimum height casting shadows ("Min_cast") and maximum height not casting shadows ("Max_fail") offers an indication of within-study area variability in snow depth: a small difference between Min_cast and Max_fail suggests uniform snow depth across a study site, whereas a large difference suggests snow drifting and wind scouring.

Among-study area variability is evident by comparing the differences in Min_cast and Max_fail between areas. With over a meter of difference between Min_cast and Max_fail, "BuffaloDrifts" (**Fig. 12**) and "BobWoods" (**Fig. 9**) appear to have more drifting and scouring than the other four study areas that display 10 cm or less difference between Min_cast and Max_fail.

The among-area variability varies more when comparing the Max_fail (range = 1.5 m) and Min_cast (range = 1.6 m) than within-area.

```
merge(shadows.no,shadows.yes,by="Study.area") %>%
    dplyr::mutate(diff.m= abs(Max_fail - Min_cast)) %>%
    arrange(diff.m) %>%
  kable(caption="Sample size (n.trees.x) of trees without shadows
        with maximum height (Max_fail) in meters and of trees
        with shadows (n.trees.x) with minimum height (Min_cast)
        in meters. Difference between Max_fail and Min_cast gives
        index of snow depth variability.")
```

Sample size (n.trees.x) of trees without shadows with maximum height (Max_fail) in meters and of trees with shadows (n.trees.x) with minimum height (Min_cast) in meters. Difference between Max_fail and Min_cast gives index of snow depth variability.

Study.area	Max_fail	n.trees.x	Min_cast	n.trees.y	diff.m
AmakE	3.6	14	3.6	16	0.0
Cutler Main	3.7	21	3.8	5	0.1
AmakW	3.0	8	3.1	69	0.1
GaiaHill	2.9	14	2.8	72	0.1
BobWoods	3.4	7	2.2	65	1.2

Study.area	Max_fail	n.trees.x	Min_cast	n.trees.y	diff.m
BuffaloDrifts	4.4	9	3.1	33	1.3

4.1.2 Linear mixed model of height on shadow length

The above analysis shows random variability in snow depth. Snow depth is a physical property of the landscape captured by the intercept in a linear model of height on shadow length. There is also random variability in the terrain's hill-slope relative to the sun. The relative sun-elevation angle across the landscape is captured by the regression slope. The section above shows that there is variability in snow depth both within and among sites. We address the variability using a linear mixed effects model with study-site as a random factor with six levels.

The **lmer()** function of the *lme4* R package handles unbalanced designs well. Here, "Shadow.m" is the fixed covariate of interest, "Study.site" is the random spatial factor, and the model specifies both random intercept (snow depth variability) and random regression slope (sun elevation angle variability)

```
mod.mer <- lmer(Height.m ~ Shadow.m + (Shadow.m|Study.area),
      data=dat.L, REML=T)

Intercept <- mean(coef(mod.mer)$Study.area[,1])

Slope <- mean(coef(mod.mer)$Study.area[,2])

summary(mod.mer)
```

```
## Linear mixed model fit by REML ['lmerMod']
## Formula: Height.m ~ Shadow.m + (Shadow.m | Study.area)
##    Data: dat.L
##
## REML criterion at convergence: 458
##
## Scaled residuals:
##      Min       1Q   Median       3Q      Max
## -3.04918 -0.60994 -0.02494  0.61164  2.83697
##
## Random effects:
##  Groups     Name        Variance Std.Dev. Corr
##  Study.area (Intercept) 0.045953 0.21437
##             Shadow.m    0.006669 0.08166  -0.46
##  Residual               0.303897 0.55127
## Number of obs: 260, groups:  Study.area, 6
##
## Fixed effects:
##             Estimate Std. Error t value
## (Intercept)  2.84714    0.13847  20.561
## Shadow.m     0.26997    0.03761   7.178
##
## Correlation of Fixed Effects:
##          (Intr)
## Shadow.m -0.571
```

Calculating the random effects covariance matrix using the correlation matrix and standard deviations for (Intercept) and Shadow.m follows:

```r
##these are the random effects standard deviations
S.ran <- c(0.21437,0.08166)

## these are random effects correlations
R.ran <- matrix(c(1,-0.46,-0.46,1),
                        nrow=2)

##these are the fixed effects standard deviations
S.fix <- c(0.13847,0.03761 )
## this is fixed effects correlation
R.fix <- matrix(c(1,-0.571,-0.571,1),
                        nrow=2)

## according to B. Bolker and others at
## https://stats.stackexchange.com/questions/62850/obtaining-covariance-matrix-from-correlation-matrix
# the following gives covariance matrices easily
(cov.ran <- outer(S.ran,S.ran)*R.ran)
```

```
##              [,1]         [,2]
## [1,]  0.045954497 -0.008052509
## [2,] -0.008052509  0.006668356
```

```r
(cov.fix <- outer(S.fix,S.fix)*R.fix)
```

```
##              [,1]         [,2]
## [1,]  0.019173941 -0.002973686
## [2,] -0.002973686  0.001414512
```

```r
##  fixed effects estimates
(mu <- fixef(mod.mer))
```

```
## (Intercept)    Shadow.m
##   2.8471433   0.2699687
```

Given these covariance matrices and a vector of means for use in the **mvrnorm()** function in the *MASS* package, we calculated Monte Carlo estimates of heights by shadow length.

The following table displays the estimates of snow depth by location using the mixed effects model intercepts and the tangent of the relative sun elevation angles as regression slope. Taking the inverse tangent of the regression slope we can compare the mean relative sun elevation angle to the sun elevation angle on a flat surface the day the imagery was captured.

```r
coefficents <- coef(mod.mer)$Study.area

names(coefficents) <- c("Intercept", "Shadow.m")

coefficents <- round(coefficents[order(coefficents$Intercept),],2)

dplyr::mutate(coefficents,
   rel.sun.elev.deg=round(180*atan(Shadow.m)/pi),
   imagery.sun.elev.deg=c(18,18,18,18,27,27),
   diff.angle=imagery.sun.elev.deg-rel.sun.elev.deg) %>%
    arrange(diff.angle) %>%
  kable(caption="Regression coefficients (Intercept and slope = Shadow.m), average sun elevation angle
```

Regression coefficients (Intercept and slope = Shadow.m), average sun elevation angle (degrees) relative to hill slope (rel.sun.elev.deg), imagery sun elevation angle (degrees) from metadata (imagery.sun.elev.deg), and difference between elevation angles giving estimate of mean hill slope in direction of sun azimuth.

	Intercept	Shadow.m	rel.sun.elev.deg	imagery.sun.elev.deg	diff.angle
BobWoods	2.67	0.33	18	18	0
GaiaHill	2.73	0.27	15	18	3
Cutler Main	2.87	0.25	14	18	4
AmakE	2.91	0.39	21	27	6
BuffaloDrifts	2.86	0.20	11	18	7
AmakW	3.04	0.19	11	27	16

The difference between "imagery sun elevation angle" and the "relative sun elevation angle" is the terrain slope angle measured along the sun azimuth angle in the direction away from the sun. If the difference is positive, as here for all sites but GaiaHill, then the terrain slope is such that the shadow is cast uphill.

We can plot the snow depth estimates as horizontal lines alongside tree heights measured in the field categorized by whether they cast visible shadows or not.

```
dat.L %>%
    ggplot(aes(y=Height.m,x=Study.area,fill=Shadow)) +
    geom_violin() +
    labs(x="Landscapes",
         y="Field height (m)") +
    geom_hline(yintercept=2.5) +
      geom_hline(yintercept=2.8,
        linetype="dashed") +
    geom_hline(yintercept=coef(mod.mer)$Study.area[,1],
        linetype="dotted",
        colour=1:6)
```

4.2 What's the probability of not casting a shadow given height?

We can apply a generalized linear mixed effects logistic regression model to estimate probability of not casting a shadow as a function of tree height, where study-area provides the random effects in intercept and regression slope.

```
inv.logt <- function(a,b,x){exp(a + b*x)/(1+ exp(a + b*x))}

dat.L  <- dat.L %>%
    dplyr::mutate(no.shadow= ifelse(Shadow=="no",1,0))

shad.mer <- glmer(no.shadow~Height.m+(Height.m | Study.area),
                     data=dat.L,
                     family=binomial,
                     subset=Height.m <= 6)
summary(shad.mer)
```

```
## Generalized linear mixed model fit by maximum likelihood (Laplace
##   Approximation) [glmerMod]
##  Family: binomial  ( logit )
## Formula: no.shadow ~ Height.m + (Height.m | Study.area)
##    Data: dat.L
```

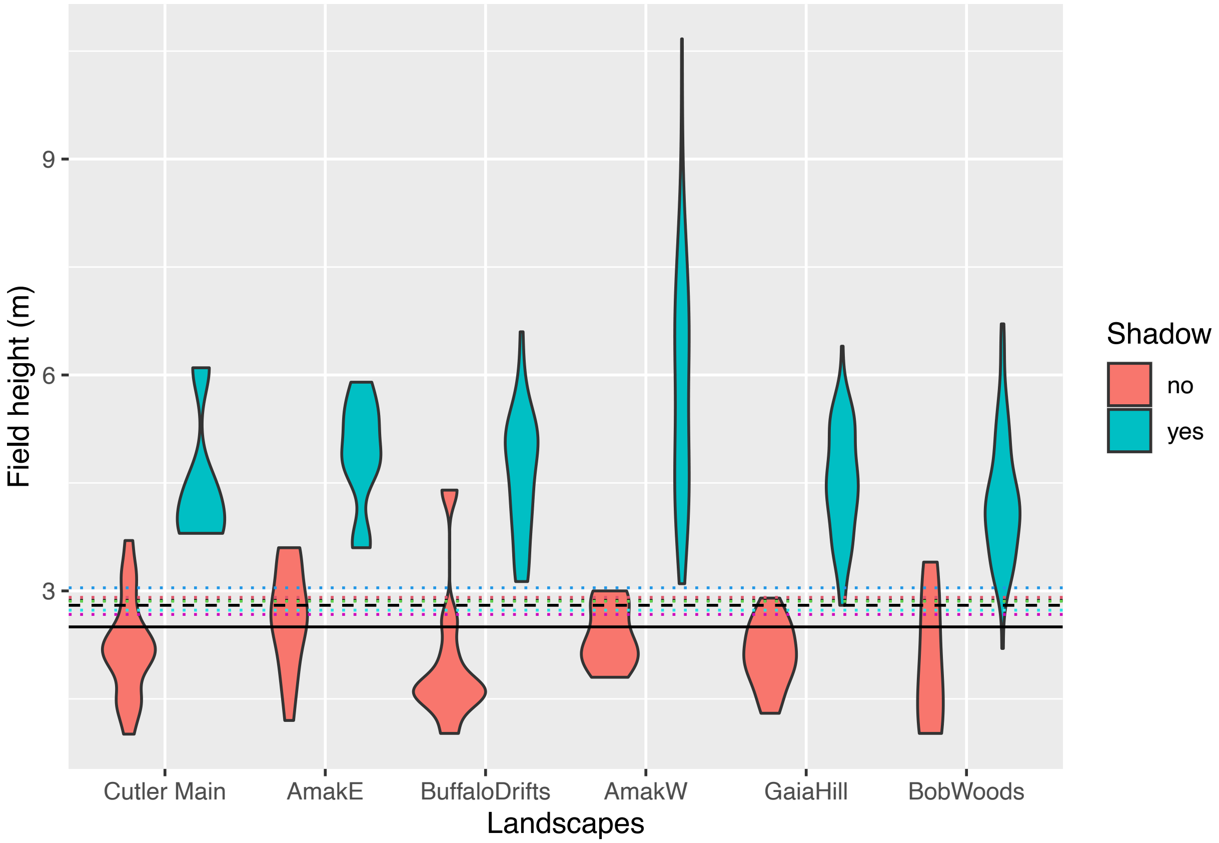

Distributions as violin plots of field measured height of trees with (blue) and without (red) shadows seen on World-View snow-covered satellite scenes. Landscapes give name of field sites. Horizontal dotted lines are random intercepts by study area. The dashed line is the mean of random intercepts at 2.8 m, and the solid line is 2.5 m, defining adult size used in the text.

```
##  Subset: Height.m <= 6
##
##      AIC      BIC   logLik deviance df.resid
##     84.2    102.6    -37.1     74.2      290
##
## Scaled residuals:
##     Min      1Q  Median      3Q     Max
## -2.4341 -0.0697 -0.0121 -0.0006 21.0377
##
## Random effects:
##  Groups     Name        Variance Std.Dev. Corr
##  Study.area (Intercept) 31.166   5.583
##             Height.m     1.537   1.240    -0.97
## Number of obs: 295, groups:  Study.area, 6
##
## Fixed effects:
##             Estimate Std. Error z value Pr(>|z|)
## (Intercept)   16.214      5.177   3.132  0.00173 **
## Height.m      -5.136      1.443  -3.560  0.00037 ***
## ---
## Signif. codes:  0 '***' 0.001 '**' 0.01 '*' 0.05 '.' 0.1 ' ' 1
##
## Correlation of Fixed Effects:
##          (Intr)
## Height.m -0.987
```

Let's compare the mixed effects model above and a fixed effects model to the data.

```
shad.lm <- glm(no.shadow~Height.m,
                     data=dat.L,
                     family=binomial,
                     subset=Height.m <= 6)

littlecuts <- seq(1,9.5,0.5)
mids <- (littlecuts[1:(length(littlecuts)-1)] +
                littlecuts[2:length(littlecuts)])/2

dat.L.ag.tot <- dat.L %>%
    dplyr::mutate(Height.class=cut(Height.m,littlecuts)) %>%
    aggregate(no.shadow~Height.class,
                        FUN=length,
                        data=.)

dat.L.ag <- dat.L %>%
    dplyr::mutate(Height.class=cut(Height.m,littlecuts)) %>%
    aggregate(no.shadow~Height.class,
                        FUN=sum,
                        data=.) %>%
    dplyr::mutate(prop.no.shad=no.shadow/dat.L.ag.tot$no.shadow,
                 Height.class=Height.class,
                 mids=mids,
                 .keep="none")

plot(prop.no.shad~mids,
                     data=dat.L.ag,
```

```r
         pch=16,cex=0.5,ann=F)

mtext(side=1,line=2,"Height (m)")
mtext(side=2,line=2,"Probability unobserved")

curve(inv.logt(fixef(shad.mer)[1],fixef(shad.mer)[2],x),
 add=T,col="blue")

curve(inv.logt(coef(shad.lm)[1],coef(shad.lm)[2],x),
 add=T)

abline(h=0.5,lty=2)

abline(v=-fixef(shad.mer)[1]/fixef(shad.mer)[2],lty=3,col="blue")
abline(v=-coef(shad.lm)[1]/coef(shad.lm)[2],lty=3)

legend("topright",
             bty="n",
             lty=1,
             col=c("black","blue"),
             legend=c("glm","glmer"))

mtext(line=0,side=3,col="blue",
            at=-fixef(shad.mer)[1]/fixef(shad.mer)[2],
            text=round(-fixef(shad.mer)[1]/fixef(shad.mer)[2],1))

mtext(line=0,side=1,col="black",
            at=-coef(shad.lm)[1]/coef(shad.lm)[2],
            text=round(-coef(shad.lm)[1]/coef(shad.lm)[2],1))
```

```r
#-fixef(shad.mer)[1]/fixef(shad.mer)[2]

# inv.logt(fixef(shad.mer)[1],fixef(shad.mer)[2],3)
```

The figure below, showing the probability of not casting a shadow as a function of height based on two models, has plots with inflection points at 3 and 3.2 m, suggesting that trees $\leq$ 3.2 m are more likely to be unobserved on the winter scene WV imagery. Thus, using the shadows to estimate the population of adults will underestimate that population and given the positive relationship between age and tree height suggests that the simulated population will miss many younger trees: hence the cut-off at about 1980.

4.2.1 Model of tree height given shadow length uses the fixed effects:

We see that the fixed effects 2.8 and 0.27 represent a mean snow depth above 2.8 m and a sun elevation angle (imagery sun elevation angles: 18°, 22°, and 27°) relative to the surface snow of 15 degrees, suggesting a mostly southerly (imagery azimuth 181°) and southwesterly sun azimuth (226°) cast on slopes facing in part away from the sun's azimuth at the time of image capture.

Height = 2.8 + 0.27 $\times$ Shadow length.

4.2.2 Estimating 1,971 tree heights using shadow lengths and binning by height

We first use the fixed effects of the mixed model (with random effects covariance structure to increase variability in intercept and slope reflecting the variability in topography) to estimate height, then bin into

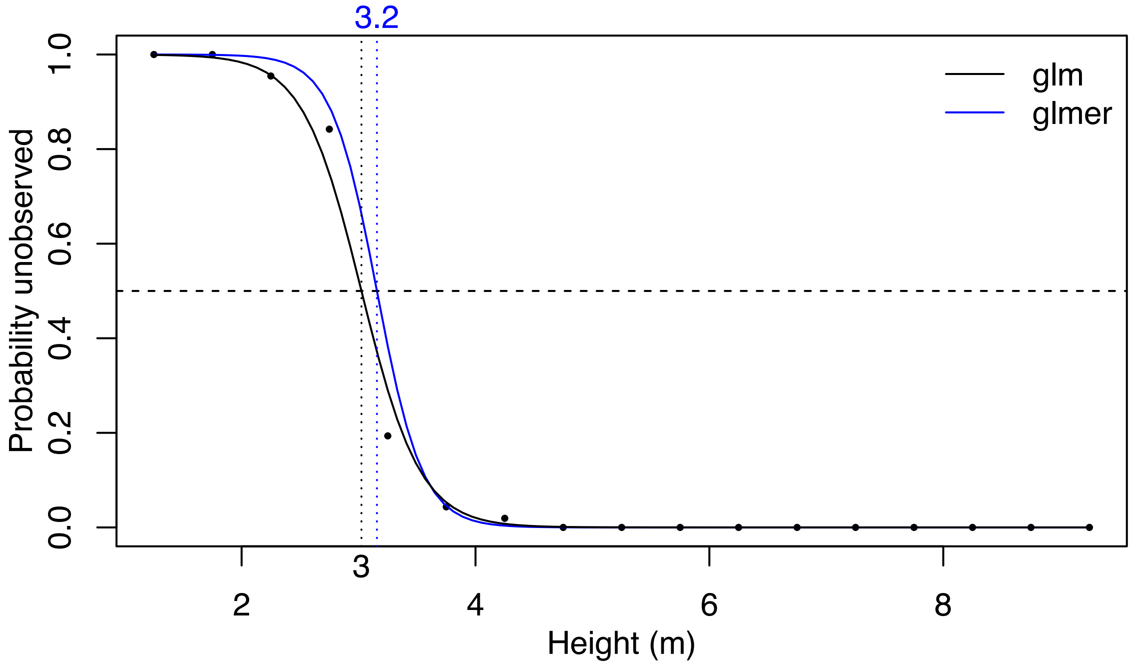

Mixed effects generalized linear model (blue) and fixed effects generalized linear model (black) of probability of not casting a shadow as a function of tree height. The hoizontal line gives 0.5 probability of not casting a shadow and the vertical lines give for each model the predicted tree height that has a 50% chance of casting a shadow.

five height classes (below in **SI Calc. 4.8** we provide a sensitivity analysis with three size classes of wider height intervals):

1. 3-4 m
2. 4-4.5 m
3. 4.5-5 m
4. 5-5.5 m
5. 5.5-7 m

4.2.2.1 Comparison of random vs. fixed effects covariance structure.

First, to see the variability using the random effects covariance structure between slope and intercept and fixed effects we will take random draws from each and display bivariate density plots showing the much greater variability in the random effects.

```
library(MASS)
n <- 2000
ran.df <- mvrnorm(n,
                Sigma=cov.ran,
                mu=mu) %>%
    data.frame() %>%
    dplyr::mutate(effects="random")

fix.df <- mvrnorm(n,
                Sigma=cov.fix,
                mu=mu) %>%
    data.frame() %>%
    dplyr::mutate(effects="Fixed")
```

```
rbind(ran.df,fix.df) %>%
    ggplot(aes(y=X.Intercept.,x=Shadow.m)) +
    geom_density_2d_filled(contour_var="ndensity",
                                                  alpha=0.75) +
    facet_grid(row=vars(effects)) +
    labs(x="Regression slope estimates",
            y="Intercept",
            title = "")
```

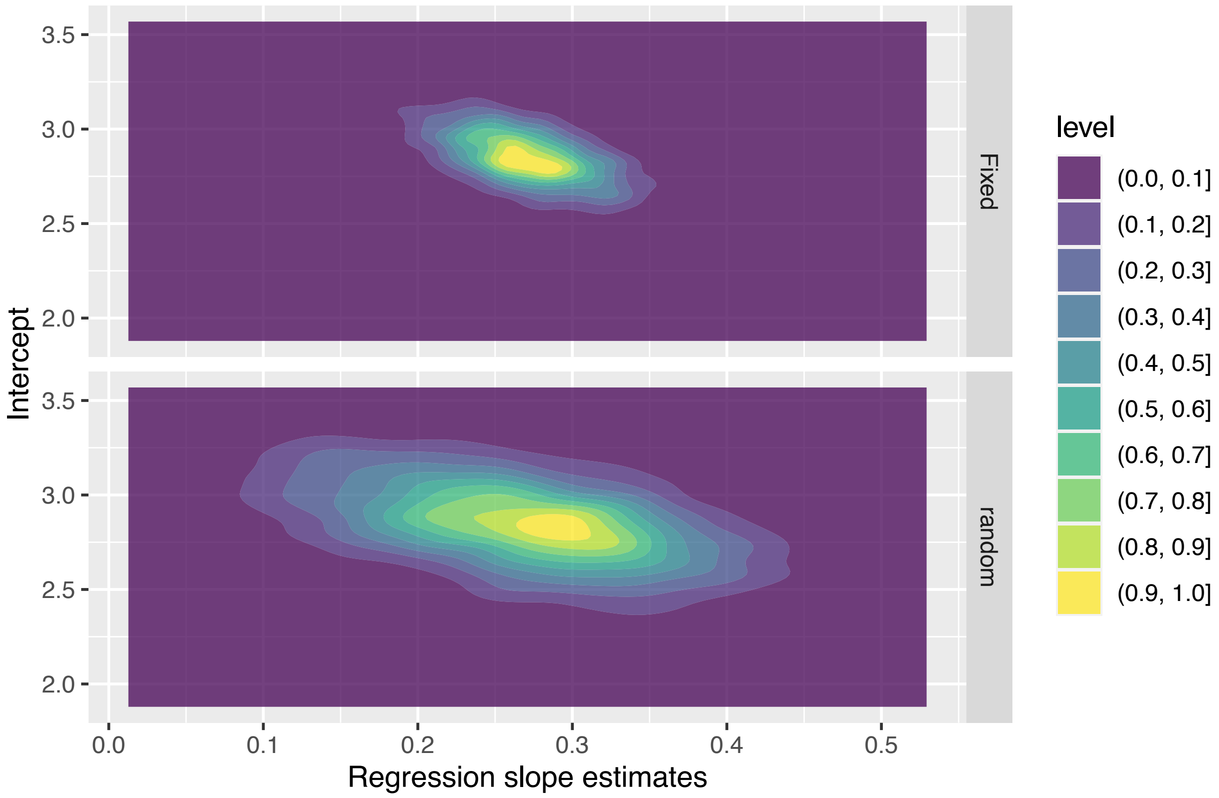

Bivariate distributions of slope and intercept for height on shadow model for fixed effects model (top) and mixed effects model (bottom).

```
cutpoints <- c(3,seq(4,5.5,0.5),7)

dat <- read.csv("./BestShadowData.csv") %>%
   subset(sun.elevation.deg < 20 &
    Shadow_m <= 14.2 &
    lat > 67.536 &
    long < -157.8) %>%
    dplyr::mutate(height.m=Slope*Shadow_m + Intercept,
        height.class=cut(height.m,cutpoints))
```

These 1,971 trees fall into the five size classes as follows:

```
dat %>% tabyl(height.class) %>%
  adorn_pct_formatting() %>%
  kable(caption="Frequency distribution of height
        estimates from 1,971 shadows in two sub-watersheds.")
```

Frequency distribution of height estimates from 1,971 shadows in two sub-watersheds.

height.class	n	percent
(3,4]	792	40.2%
(4,4.5]	763	38.7%
(4.5,5]	286	14.5%
(5,5.5]	96	4.9%
(5.5,7]	34	1.7%

We wish to estimate the establishment-year for each of these individuals by using their size class to sample from known establishment-year by size class distributions.

4.3 Establishment-year density distributions by height class

We used the trees that were aged by increment cores and the height of the increment core hole above the ground (**SI Calc. 2**). These trees were measured for their heights in the field when cored.

From data streams (2) and (3) above we will bin aged adults into height-classes and construct kernel density establishment-year distributions for each height class:

```
year.cuts <- seq(1900,2000,10)
mid.year <- (year.cuts[1:(length(year.cuts)-1)] +
                        year.cuts[2:length(year.cuts)])/2

dat.aged <- dat.r %>%
   dplyr::mutate(height.class=cut(Height_m,cutpoints),
             Age.Class=cut(Establishment_yr,year.cuts),
         Year.established=Establishment_yr,
         prob.no.shadow=inv.logt(fixef(shad.mer)[1],
                        fixef(shad.mer)[2],Height_m),
      .keep="none") %>%
    na.omit()

paste(nrow(dat.aged), "aged trees")
```

```
## [1] "123 aged trees"
```

```
dat.aged %>% tabyl(height.class) %>%
  adorn_pct_formatting() %>%
   kable(caption="Frequency distribution of 123 trees
         of known age and heights into five height-classes.")
```

Frequency distribution of 123 trees of known age and heights into five height-classes.

height.class	n	percent
(3,4]	31	25.2%
(4,4.5]	25	20.3%
(4.5,5]	21	17.1%
(5,5.5]	29	23.6%
(5.5,7]	17	13.8%

These 123 individuals with ages and heights are used to construct non-parametric, Gaussian kernel density

distributions with the following constraints:

1. Each kernel density extends as a discrete distribution across 107 years from 1897 to 2004.
2. Kernel densities are scaled so that any non-zero density value is greater than one.
3. Establishment-year distributions for each height class is constructed with a function called **sampler()** that is defined to return a discrete distribution of year of establishment.

```
sampler <- function(class){
    temp <- subset(dat.aged,height.class == class)
  dens <- density(temp$Year.established,
        bw="SJ",
            n=107, ## number of consecutive establishment years
        from=1897, ## earliest year
        to=2004) ## latest year
  k <- 1000 ## scale factor
  par(mgp=c(3,0.1,0),tcl=0.1)
    plot(k*dens$y~dens$x, ## a visualization
            ylim=c(0,50),
            type="h",ann=F)
    mtext(side=1,line=1,"Establishment year")
    mtext(side=2,line=1,"Count")
    sample.this <- data.frame(y=round(k*dens$y),
            x=round(dens$x)) %>%
    subset(y > 0)
    rep(sample.this$x,sample.this$y)
}
```

The function **sampler()** defined above is applied to each of the height classes to create the “populations” to resample for 1,000 simulations.

The code for two constructions of the sampling distributions is displayed. The remaining three distributions do not echo the code.

```
par(mfrow=c(2,1),mar=c(2,2,0,1))
bigone3_4 <- sampler("(3,4]")
    legend("topleft",
        bty="n",
        legend=paste0("total count = ",length(bigone3_4 )))
    legend("topright",
        bty="n",
        legend=paste0("size class = ","(3,4]","m"))
bigone4_4.5 <- sampler("(4,4.5]")
    legend("topleft",
        bty="n",
        legend=paste0("total count = ",length(bigone4_4.5)))
    legend("topright",
        bty="n",
        legend=paste0("size class = ","(4,4.5]","m"))
```

```
par(mfrow=c(2,2),mar=c(2,2,0,1))
bigone4.5_5 <- sampler("(4.5,5]")
    legend("topleft",
        bty="n",
        legend=paste0("total count = ",length(bigone4.5_5)))
    legend("topright",
```

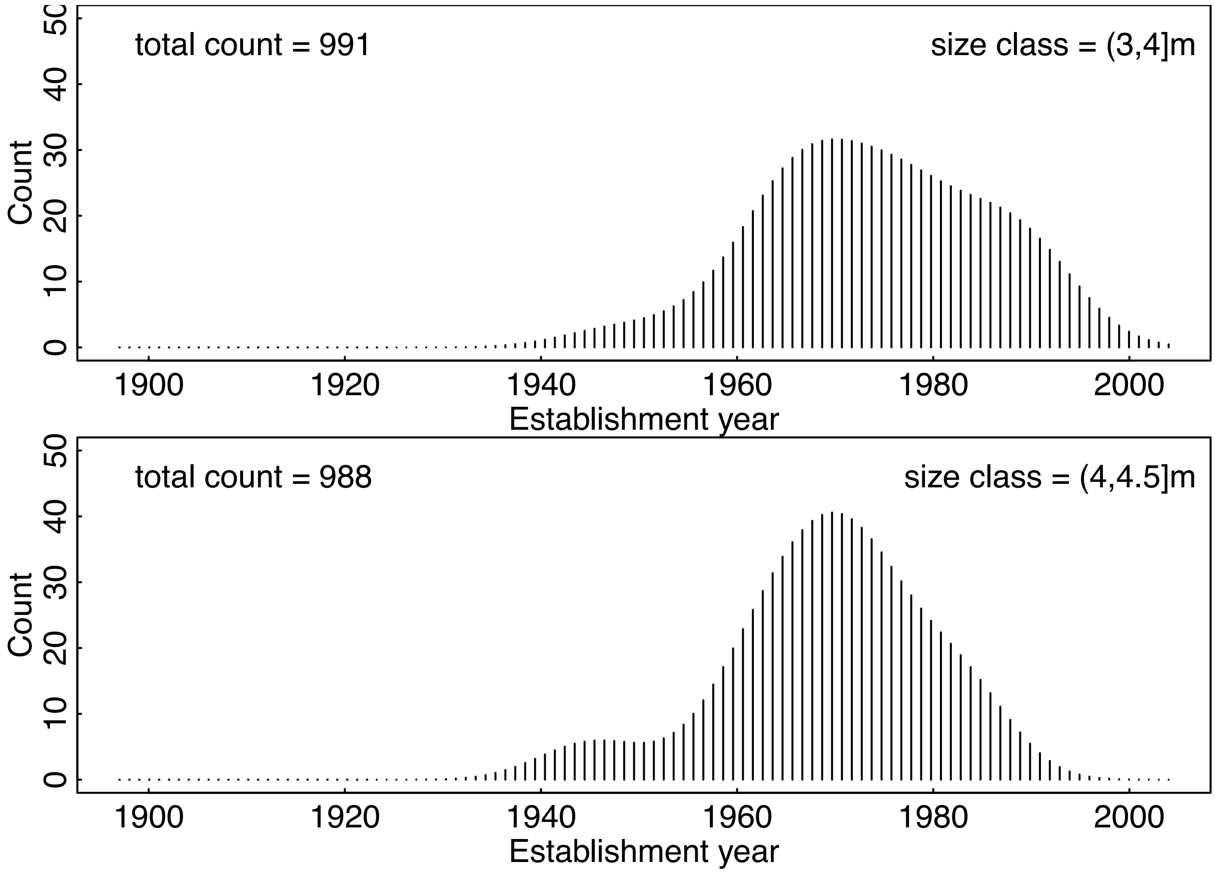

Density estimates of establishment year as discrete samples of trees by tree heights. Total count is a scaling factor. Size class gives min and max height of trees generating the distribution.

```
        bty="n",
        legend=paste0("size class = ","(4.5,5]","m"))
bigone5_5.5 <- sampler("(5,5.5]")
    legend("topleft",
        bty="n",
        legend=paste0("total count = ",length(bigone5_5.5)))
    legend("topright",
        bty="n",
        legend=paste0("size class = ","(5,5.5]","m"))
bigone5.5_7 <- sampler("(5.5,7]")
    legend("topleft",
        bty="n",
        legend=paste0("total count = ",length(bigone5.5_7)))
    legend("topright",
        bty="n",
        legend=paste0("size class = ","(5.5,7]","m"))
```

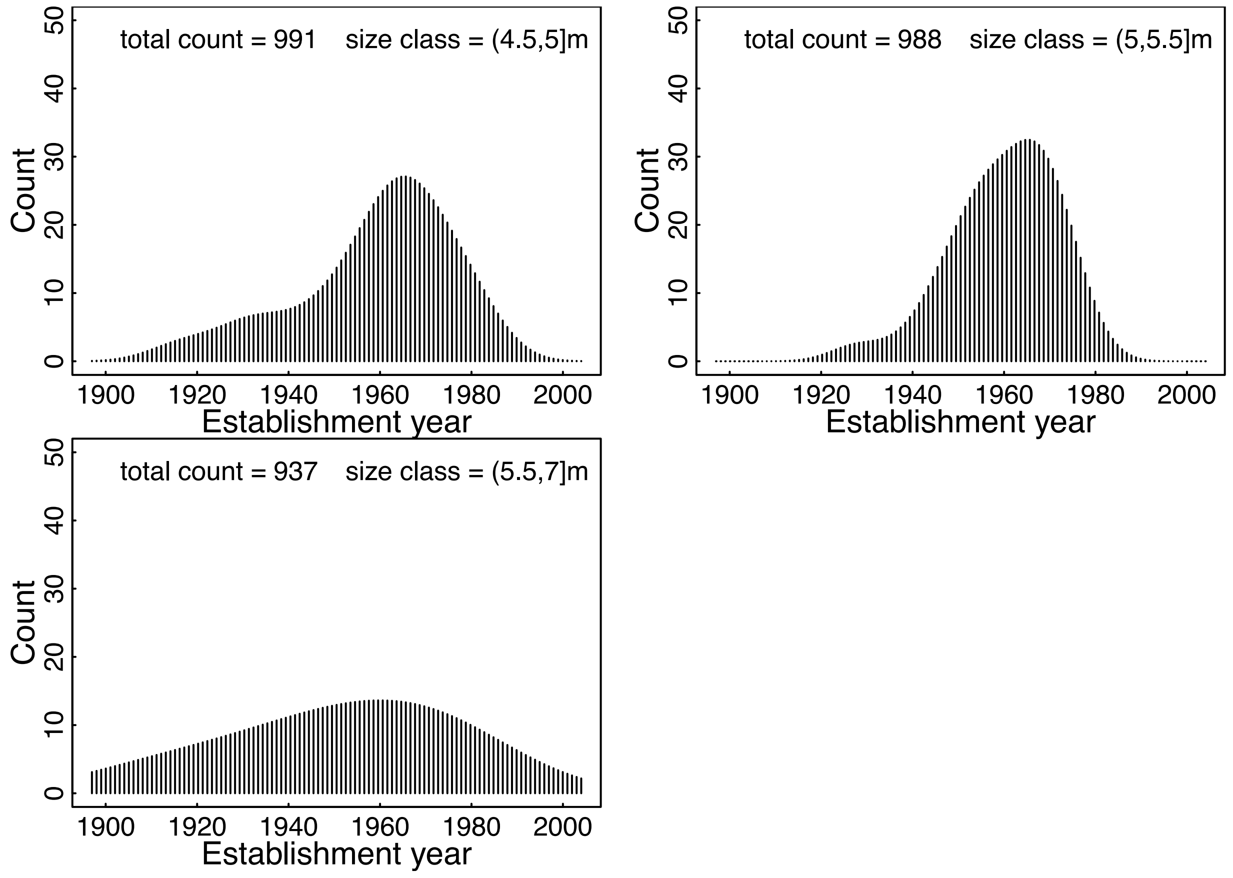

Density estimates of establishment year as discrete samples of trees by tree heights binned into five height classes. Total count is a scaling factor. Size class gives min and max height of trees generating the distribution.

Each plot above displays the discrete distribution of establishment-year as a vector to randomly sample (with replacement) given a height class: “bigone6_7”,“bigone5.5_6”, “bigone5_5.5”,“bigone4.5_5”,“bigone4_4.5”, “bigone3_4”.

4.3.1 Given a tree's height-class estimate establishment-year

The year-of-establishment vectors were sampled with replacement, using the 1,971 estimated heights binned into height-classes. Once all 1,971 individuals were identified with an establishment-year, the counts of trees in each establishment-year were aggregated over each year by summing. This aggregation represented a realization of the number of individuals recruiting for any given year.

The count of trees in each height class are defined as a data frame here (they were displayed above as a table). These counts are the number of samples to draw from the respective height-class based year-of-establishment distributions in the re-sampling below and make up a critical object in the re-sampling that follows.

```
cutpoints <- c(3,seq(4,5.5,0.5),7)

dat <- read.csv("./BestShadowData.csv") %>%
   subset(sun.elevation.deg < 20 &
    Shadow_m <= 14.2 &
    lat > 67.536 &
    long < -157.8) %>%
    dplyr::mutate(height.m=Slope*Shadow_m + Intercept,
        height.class=cut(height.m,cutpoints))

df <- aggregate(height.m~height.class,
                               FUN=length,data=dat)
    names(df)[names(df)=="height.m"] <- "count"
    sum(df$count)
```

```
## [1] 1971
```

```
sampler <- function(class){
    temp <- subset(dat.aged,height.class == class)
  dens <- density(temp$Year.established,
        bw="SJ",
            n=107, ## number of consecutive establishment years
        from=1897, ## earliest year
        to=2004) ## latest year
  k <- 1000 ## scale factor
  par(mgp=c(3,0.1,0),tcl=0.1)
    plot(k*dens$y~dens$x, ## a visualization
            ylim=c(0,50),
            type="h",ann=F)
    mtext(side=1,line=1,"Establishment year")
    mtext(side=2,line=1,"Count")
    sample.this <- data.frame(y=round(k*dens$y),
            x=round(dens$x)) %>%
    subset(y > 0)
    rep(sample.this$x,sample.this$y)
}
```

Here additional objects used in the resampling are defined.

```
n <- 1000 ## the number of resamples

Year <- 1897:2004
time <- length(Year)

rate <- 1:n ## a vector to hold each realizations relative growth rate
intercept <-1:n ## a vector to hold each realizations initial pop size
```

```r
## This data.frame has a row for each year
## and columns for year, population size and relative growth rate
pops <- data.frame(matrix(c(Year,rep(NA,time),rep(NA,time)),
                byrow=F,
                nrow=time,
                ncol=3))
    names(pops) <- c("year","pop.size","simulation")
```

4.4 1,000 simulations

We performed the resampling 1,000 times, each time fitting an exponential growth equation,

$$N(t) = N_o \exp(kt)$$

using **nls()** in R, then averaged the population relative growth rates as $\bar{k}$.

```r
dat <- read.csv("./BestShadowData.csv") %>%
   subset(sun.elevation.deg < 20 &
    Shadow_m <= 14.2 &
    lat > 67.536 &
    long < -157.8)
#sigma <- vcov(mod.mer,full=F) # the fixed effects only version of covariance
sigma <- cov.ran
```

```r
set.seed(20)
## sample for all n resamples
## propogate uncertainty of height on shadow relationship
for(i in 1:n) ## sampling year of establishment vectors by height class
    { MC.coef <- mvrnorm(1,  ## randomly sample intercept and slope
        mu=c(Intercept,Slope), ## from lmer height~shadow fixed effects
        Sigma=sigma)           ## using covariance structure
      snow.depth <- MC.coef[1]
        tangent.sun <- MC.coef[2]
    dat <- dat %>%
    dplyr::mutate(height.m=tangent.sun*Shadow_m + snow.depth,
        height.class=cut(height.m,cutpoints))
    df.1 <- aggregate(height.m~height.class,
                            FUN=length,data=dat)
    names(df.1)[names(df.1)=="height.m"] <- "count"
    df.2 <- data.frame(height.class=
                c("(3,4]","(4,4.5]",
                    "(4.5,5]","(5,5.5]",
                    "(5.5,7]"),
                count=0)
    df <- merge(df.2,df.1,
                        by="height.class",
                        all.x=T) %>%
        dplyr::mutate(height.class,
                    count=count.y,
                    count=ifelse(is.na(count),0,
                                count),
                    .keep="none")
```

```
    year <- c(
    ifelse(df$count[df$height.class=="(5.5,7]"]==0,0,
            sample(x=bigone5.5_7,
            df$count[df$height.class=="(5.5,7]"],
            replace=T)),
    sample(bigone5_5.5,
        df$count[df$height.class=="(5,5.5]"],
        replace=T),
    sample(bigone4.5_5,
        df$count[df$height.class=="(4.5,5]"],
        replace=T),
    sample(bigone4_4.5,
        df$count[df$height.class=="(4,4.5]"],
        replace=T),
    sample(bigone3_4,
        df$count[df$height.class=="(3,4]"],
        replace=T))

    ## counting number of individuals establishing each year
    ## and merging with a dummy data.frame to capture years without any
    ## establishing individuals
establishments <- aggregate(data=
        data.frame(year=year,
            count=1),
        count~year,
        FUN=sum)  %>%
base::merge(data.frame(year=Year,
        count2=0),
        by="year",
        all.y=T) %>%
dplyr::mutate(year=year,
    count = ifelse(is.na(count),
                count2,
                count),
    .keep="none") %>%
        #### cumulative sums give population size by year
dplyr::mutate(pop.size = cumsum(count))
    ## Now calculate the relative growth rate from 1900 to 1980
temp <- data.frame(
    year=establishments$year,
    pop.size=establishments$pop.size,
    simulation=i)

mod <- nls(pop.size~a*exp(r*(year-1900)),
        data=temp,
        subset=year >= 1900 &
        year < 1980 &
        pop.size > 0,
        start=list(a=1,r=0.04))
    ## save the relative growth rate
    rate[i] <- coef(mod)[2]
    intercept[i] <- coef(mod)[1]
    pops <- rbind(pops,temp)
```

```
}
```

Clean up the population data.frame "pops":

```
pops <- pops[-(1:time),] %>%
                subset(pop.size!=0)
row.names(pops) <- 1:nrow(pops)
```

4.4.1 Find the mean value of growth rate across the 1,000 bootstrap estimates

```
mean(intercept)
```

```
## [1] 6.663963
```

```
mean(rate)
```

```
## [1] 0.07268329
```

4.4.2 Expected doubling time for the population

```
paste(round(log(2)/mean(rate),1), "years")
```

```
## [1] "9.5 years"
```

4.4.3 Expected doubling time for the population

```
data.frame(rate=rate,
                     dbl.year=log(2)/rate) %>%
    ggplot(aes(x=dbl.year)) +
geom_density() +
    labs(x="Doubling time (years) from 1,000 simulations",
             title="Five height classes")
```

```
paste(round(log(2)/mean(rate),1), "years")
```

```
## [1] "9.5 years"
```

```
summary(log(2)/rate)
```

```
##    Min. 1st Qu.  Median    Mean 3rd Qu.    Max.
##   7.662   8.707   9.532   9.674  10.571  13.300
```

```
write.csv(pops,row.names=F,
                    "./sim.pops.csv")
```

This is the exponential growth function plotted in Fig. 3 of the text.

```
# define function
growth <- function(x) mean(intercept)*exp(mean(rate)*(x-1900))
```

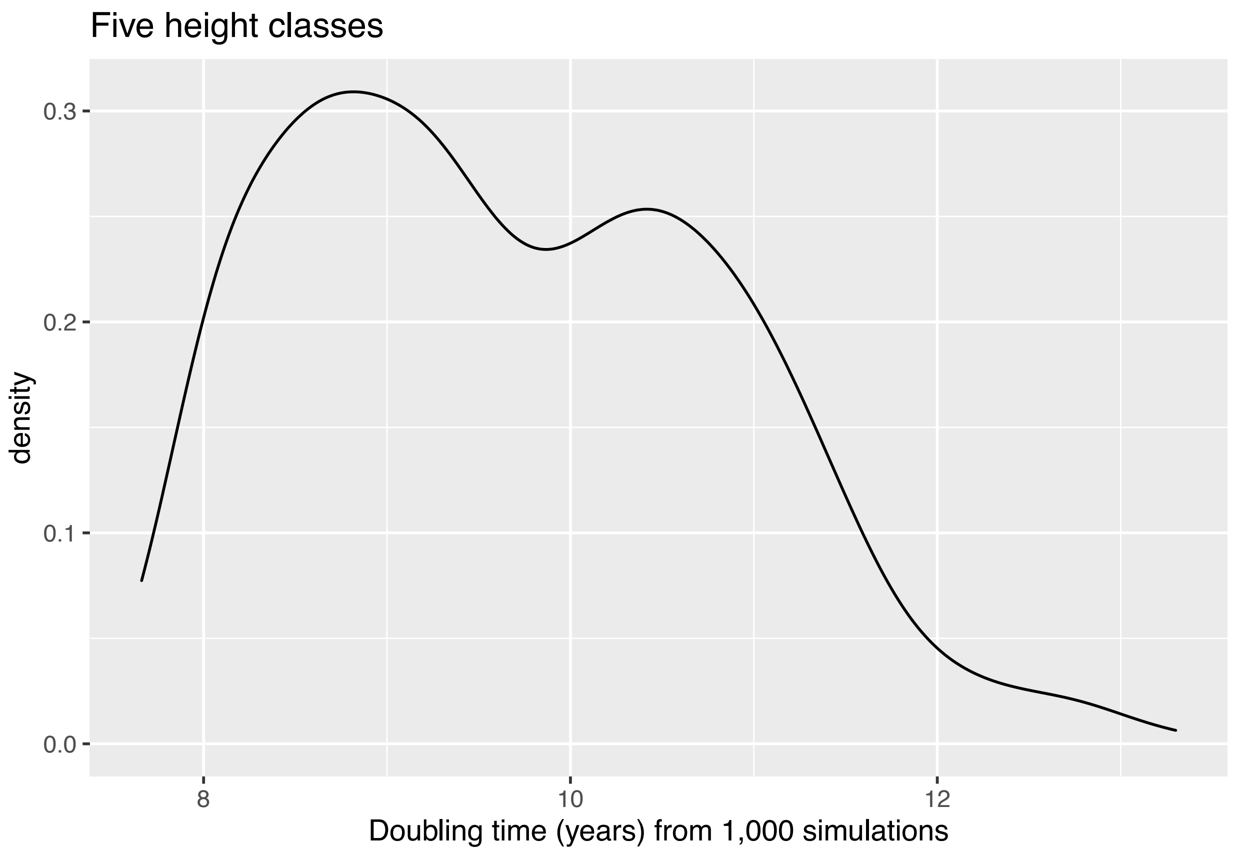

Density plot of 1,000 estimates of doubling time for the expanding population in two sub-watersheds. These simulations used five height-classes to sample establishment year.

4.5 Population simulations displayed

```
#### Plot the population reconstructions with the bootstrapped growth curve:

decades <- seq(1900,1980,10)

pops %>%
    subset(pop.size >= 1) %>%
    ggplot(aes(x=year, y=pop.size,
                         group = simulation)) +
    scale_x_continuous(limits=c(1900,1980),
            "Year",
            breaks=decades,
            labels = decades) +
        scale_y_continuous(limits=c(0,1700),
            name ="Population size",
            breaks=seq(0,2000,500),
            labels=seq(0,2000,500)) +
    geom_line(alpha=0.01,colour="#009E73")
```

```
## Warning: Removed 24013 row(s) containing missing values (geom_path).
```

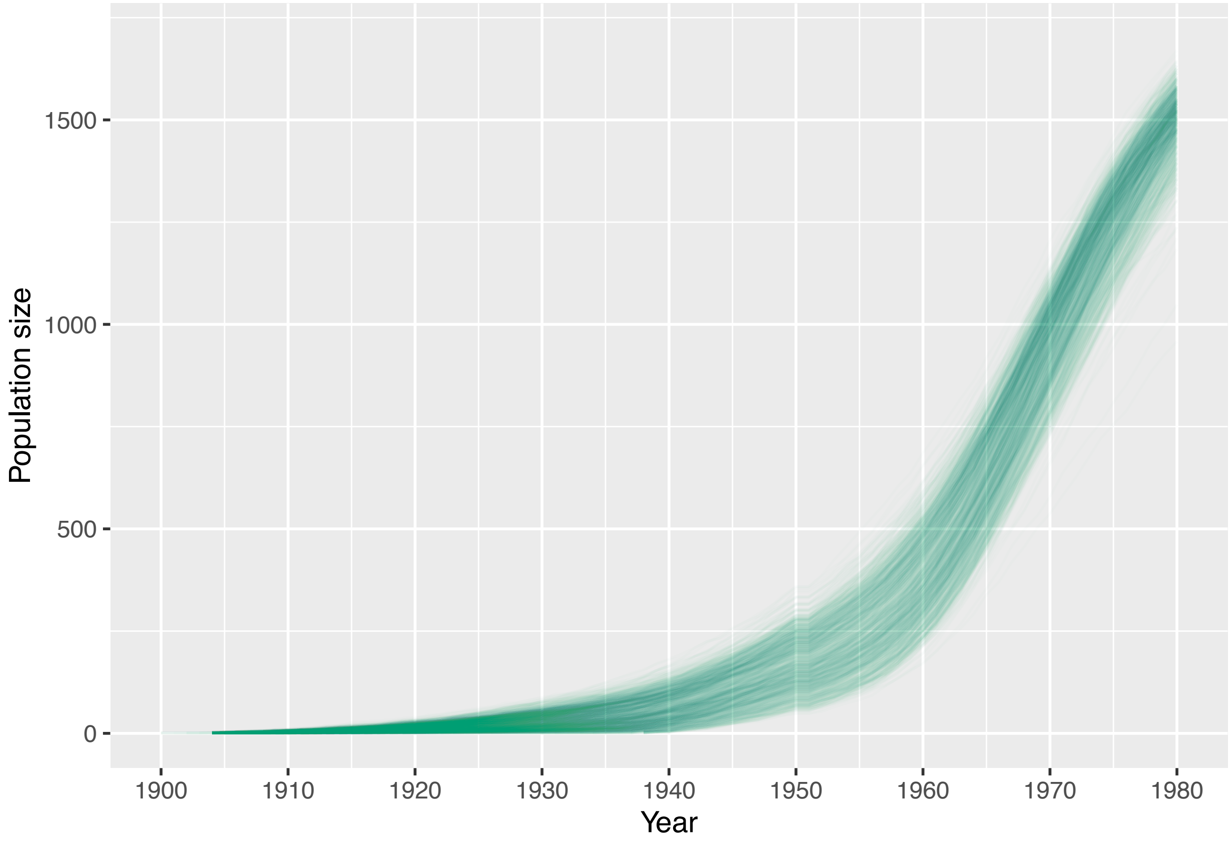

1,000 simulated populations of 1,971 trees in two sub-watersheds from 1900 to 1980 contstructed by resampling five height-class distributions of establsihment year and Monte Carlo sampling of multivariate distribution of height on shadow using a mixed effects linear model.

4.6 Sensitivity Analysis

Here we perform a sensitivity analysis of doubling time by widening each height-class to 1.3 m and so reducing the number of height classes from five to three.

The width of each height-class differs by more than the within-site variability in snow depth. The among-site snow depth is partly accounted for in the Monte Carlo sampling of model coefficients that give tree height as a function of shadow length.

```
cutpoints <- c(3,4.3,5.6,7)

dat <- read.csv("./BestShadowData.csv") %>%
   subset(sun.elevation.deg < 20 &
    Shadow_m <= 14.2 &
    lat > 67.536 &
    long < -157.8) %>%
    dplyr::mutate(height.m=Slope*Shadow_m + Intercept,
        height.class=cut(height.m,cutpoints))
```

These 1,971 trees fall into the three size classes as follows:

```
dat %>% tabyl(height.class) %>%
  adorn_pct_formatting() %>%
  kable(caption="Frequency distribution of 1,971
        shadows into three height-classes predicted to cast the shadows.")
```

Frequency distribution of 1,971 shadows into three height-classes predicted to cast the shadows.

height.class	n	percent
(3,4.3]	1277	64.8%
(4.3,5.6]	663	33.6%
(5.6,7]	31	1.6%

```
year.cuts <- seq(1900,2000,10)
mid.year <- (year.cuts[1:(length(year.cuts)-1)] +
                        year.cuts[2:length(year.cuts)])/2

dat.aged <- dat.r %>%
   dplyr::mutate(height.class=cut(Height_m,cutpoints),
             Age.Class=cut(Establishment_yr,year.cuts),
         Year.established=Establishment_yr,
         prob.no.shadow=inv.logt(fixef(shad.mer)[1],
                        fixef(shad.mer)[2],Height_m),
      .keep="none") %>%
    na.omit()

paste(nrow(dat.aged), "aged trees")
```

```
## [1] "123 aged trees"
```

```
dat.aged %>% tabyl(height.class) %>%
  adorn_pct_formatting() %>%
  kable(caption="Frequency distribution of 123 trees
        of known age and heights into three height-classes.")
```

Frequency distribution of 123 trees of known age and heights into three height-classes.

height.class	n	percent
(3,4.3]	45	36.6%
(4.3,5.6]	65	52.8%
(5.6,7]	13	10.6%

```
par(mfrow=c(2,2),mar=c(2,2,0,1))
bigone3_4.3 <- sampler("(3,4.3]")
    legend("topleft",
        bty="n",
        legend=paste0("total count = ",length(bigone3_4.3 )))
    legend("topright",
        bty="n",
        legend=paste0("size class = ","(3,4.3]","m"))

bigone4.3_5.6 <- sampler("(4.3,5.6]")
    legend("topleft",
        bty="n",
        legend=paste0("total count = ",length(bigone4.3_5.6)))
    legend("topright",
        bty="n",
        legend=paste0("size class = ","(5,5.5]","m"))

bigone5.6_7 <- sampler("(5.6,7]")
    legend("topleft",
        bty="n",
        legend=paste0("total count = ",length(bigone5.6_7)))
    legend("topright",
        bty="n",
        legend=paste0("size class = ","(5.6,7]","m"))
```

```
cutpoints <- c(3,4.3,5.6,7)

dat <- read.csv("./BestShadowData.csv") %>%
   subset(sun.elevation.deg < 20 &
    Shadow_m <= 14.2 &
    lat > 67.536 &
    long < -157.8) %>%
    dplyr::mutate(height.m=Slope*Shadow_m + Intercept,
        height.class=cut(height.m,cutpoints))

df <- aggregate(height.m~height.class,
                                FUN=length,data=dat)
    names(df)[names(df)=="height.m"] <- "count"
    sum(df$count)
```

```
## [1] 1971
```

```
    df %>% kable()
```

height.class	count
(3,4.3]	1277

height.class	count
(4.3,5.6]	663
(5.6,7]	31

```
n <-  1000 ## the number of resamples

Year <- 1897:2004
time <- length(Year)

rate <- 1:n ## a vector to hold each realizations relative growth rate
intercept <-1:n ## a vector to hold each realizations initial pop size

## This data.frame has a row for each year
## and columns for year, population size and relative growth rate
pops <- data.frame(matrix(c(Year,rep(NA,time),rep(NA,time)),
                byrow=F,
                nrow=time,
                ncol=3))
    names(pops) <- c("year","pop.size","simulation")
```

```
dat <- read.csv("./BestShadowData.csv") %>%
   subset(sun.elevation.deg < 20 &
    Shadow_m <= 14.2 &
    lat > 67.536 &
    long < -157.8)
#sigma <- vcov(mod.mer,full=F) # the fixed effects only version of covariance
sigma <- cov.ran
```

```
set.seed(14)
## sample for all n resamples
## propogate uncertainty of height on shadow relationship
for(i in 1:n) ## sampling year of establishment vectors by height class
    { MC.coef <- mvrnorm(1,  ## randomly sample intercept and slope
        mu=c(Intercept,Slope), ## from lmer height~shadow fixed effects
        Sigma=sigma)           ## using covariance structure
      snow.depth <- MC.coef[1]
        tangent.sun <- MC.coef[2]
    dat <- dat %>%
    dplyr::mutate(height.m=tangent.sun*Shadow_m + snow.depth,
        height.class=cut(height.m,cutpoints))
    df.1 <- aggregate(height.m~height.class,
                            FUN=length,data=dat)
    names(df.1)[names(df.1)=="height.m"] <- "count"
    df.2 <- data.frame(height.class=
                c("(3,4.3]",
                    "(4.3,5.6]",
                    "(5.6,7]"),
                count=0)
    df <- merge(df.2,df.1,
                        by="height.class",
                        all.x=T) %>%
        dplyr::mutate(height.class,
                count=count.y,
```

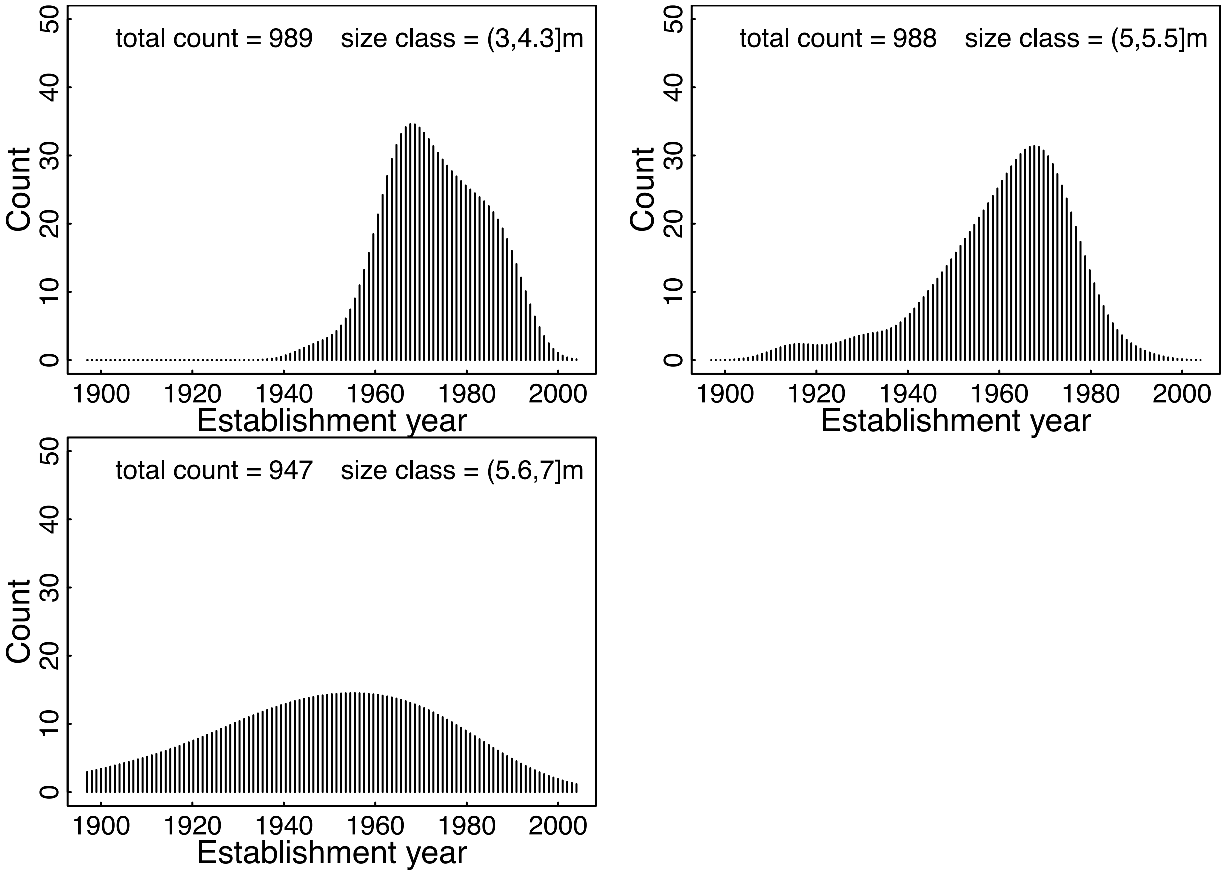

Density estimates of establishment year as discrete samples of trees by tree heights binned into three height classes. Total count is a scaling factor. Size class gives min and max height of trees generating the distribution.

```
                     count=ifelse(is.na(count),0,
                                  count),
                     .keep="none")

    year <- c(
    ifelse(df$count[df$height.class=="(5.6,7]"]==0,0,
            sample(x=bigone5.6_7,
            df$count[df$height.class=="(5.6,7]"],
            replace=T)),
    sample(bigone4.3_5.6,
        df$count[df$height.class=="(4.3,5.6]"],
        replace=T),
    sample(bigone3_4.3,
        df$count[df$height.class=="(3,4.3]"],
        replace=T))

    ## counting number of individuals establishing each year
    ## and merging with a dummy data.frame to capture years without any
    ## establishing individuals
establishments <- aggregate(data=
        data.frame(year=year,
            count=1),
        count~year,
        FUN=sum)  %>%
base::merge(data.frame(year=Year,
        count2=0),
        by="year",
        all.y=T) %>%
dplyr::mutate(year=year,
    count = ifelse(is.na(count),
                count2,
                count),
    .keep="none") %>%
        #### cumulative sums give population size by year
dplyr::mutate(pop.size = cumsum(count))
    ## Now calculate the relative growth rate from 1900 to 1980
temp <- data.frame(
    year=establishments$year,
    pop.size=establishments$pop.size,
    simulation=i)

mod <- nls(pop.size~a*exp(r*(year-1900)),
        data=temp,
        subset=year >= 1900 &
        year < 1980 &
        pop.size > 0,
        start=list(a=1,r=0.04))
    ## save the relative growth rate
    rate[i] <- coef(mod)[2]
    intercept[i] <- coef(mod)[1]
    pops <- rbind(pops,temp)
}
```

Clean up the population data.frame "pops":

```
pops <- pops[-(1:time),] %>%
                subset(pop.size!=0)
row.names(pops) <- 1:nrow(pops)
```

4.6.1 Find the mean value of growth rate across the 1,000 bootstrap estimates

```
mean(intercept)
```

```
## [1] 7.640221
```

```
mean(rate)
```

```
## [1] 0.07147869
```

```
double.time <- data.frame(rate=rate,
                     dbl.year=round(log(2)/rate,1))
```

4.6.2 Expected doubling time for the population

```
data.frame(rate=rate,
                     dbl.year=log(2)/rate) %>%
    ggplot(aes(x=dbl.year)) +
geom_density()+
    labs(x="Doubling time (years) from 1,000 simulations",
            title="Three height classes")
```

```
paste(round(log(2)/mean(rate),1), "years")
```

```
## [1] "9.7 years"
```

```
summary(log(2)/rate)
```

```
##    Min. 1st Qu.  Median    Mean 3rd Qu.    Max.
##   7.447   8.808   9.798   9.865  10.796  13.092
```

```
#### Plot the population reconstructions with the bootstrapped growth curve:

decades <- seq(1900,1980,10)


pops %>%
    subset(pop.size >= 1) %>%
    ggplot(aes(x=year, y=pop.size,
                         group = simulation)) +
    scale_x_continuous(limits=c(1900,1980),
            "Year",
            breaks=decades,
            labels = decades) +
        scale_y_continuous(limits=c(0,1700),
            name ="Population size",
            breaks=seq(0,2000,500),
            labels=seq(0,2000,500)) +
    geom_line(alpha=0.01,colour="#009E73")
```

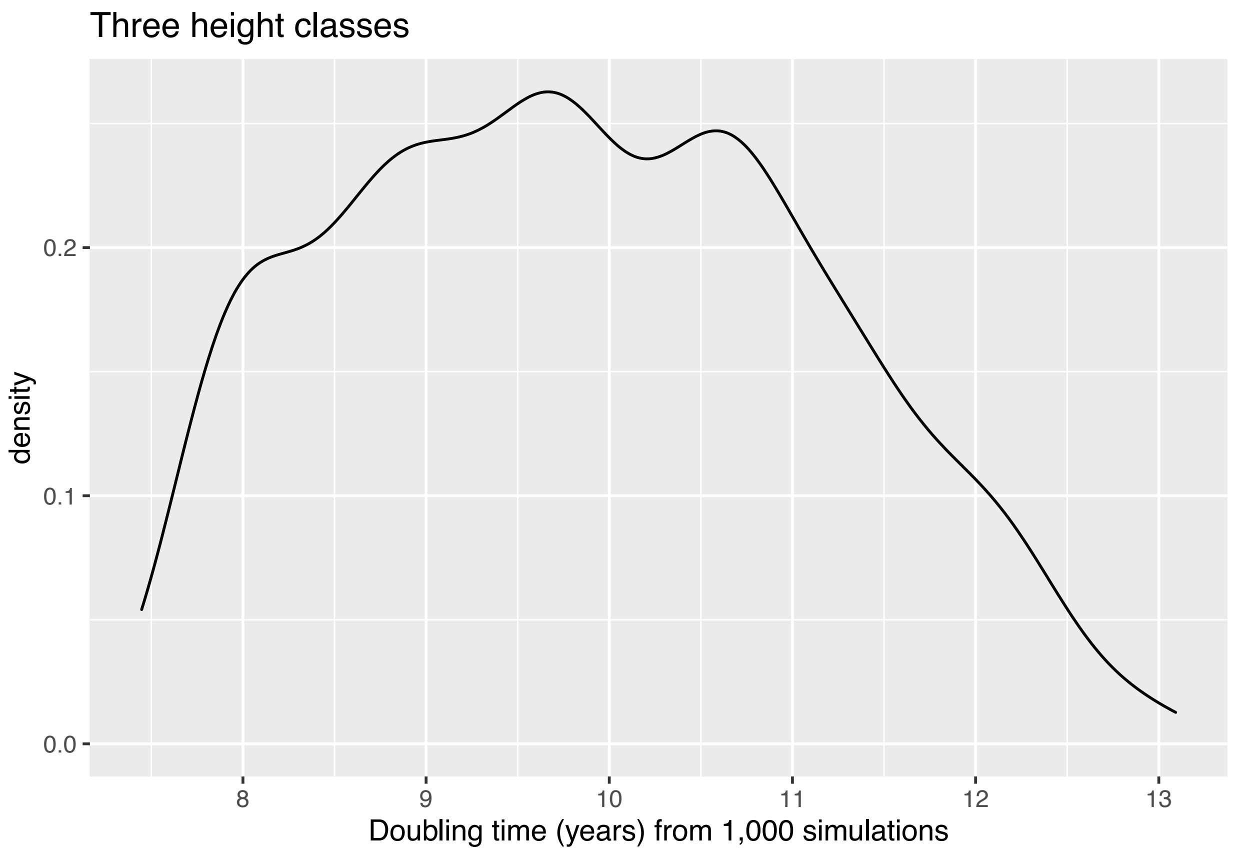

Density plot of 1,000 estimates of doubling time for the expanding population in two sub-watersheds. These simulations used three height-classes to sample establishment year.

```
## Warning: Removed 24016 row(s) containing missing values (geom_path).
```

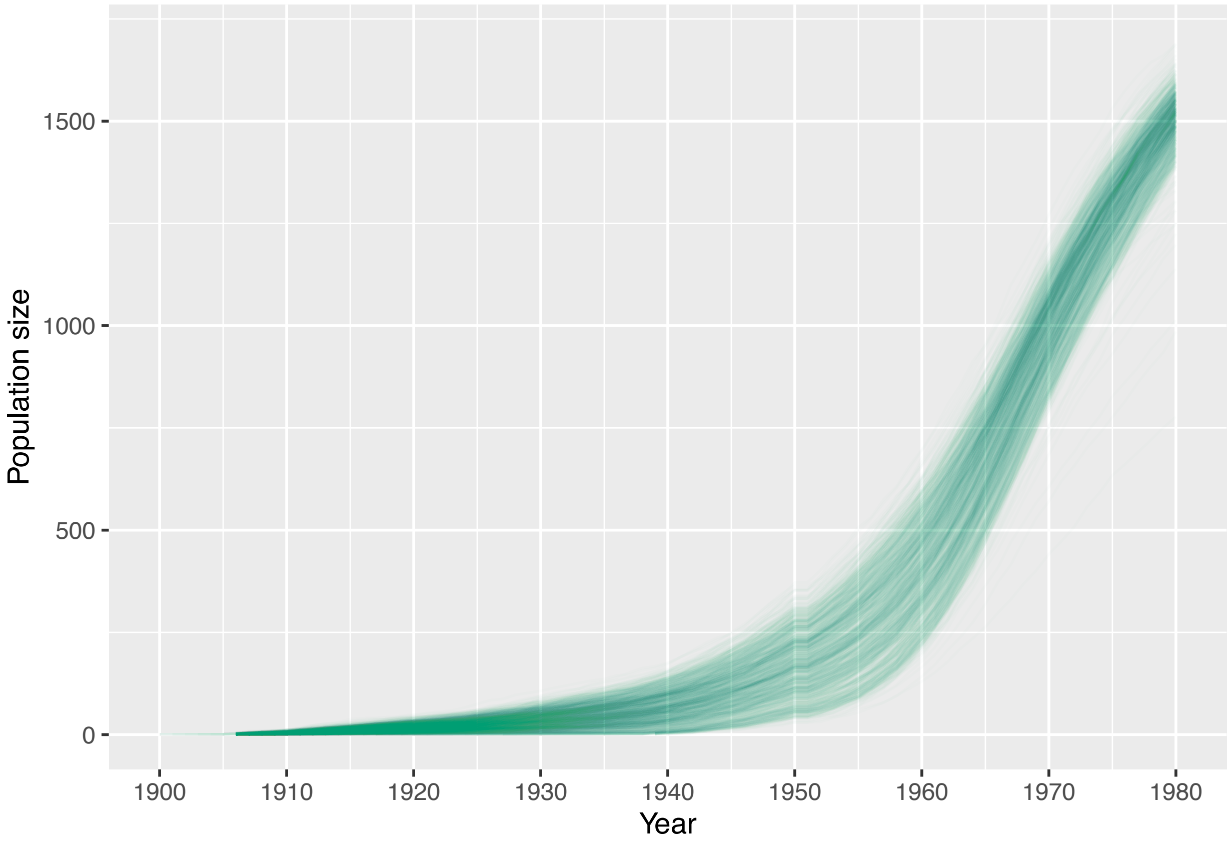

1,000 simulated populations of 1,971 trees in two sub-watersheds from 1900 to 1980 contsructed by resampling three height-class distributions of establishment year and Monte Carlo sampling of multivariate distribution of height on shadow using a mixed effects linear model.

5 Foliar Sample Comparisons

Samples from lateral growth on adults produced in 2019 were used to compare growth, foliar concentrations of nitrogen (N) and phosphorous (P), and the stable isotope δ^{15}N.

5.1 Linear mixed effects model comparing CAG

Current annual growth (CAG) in 2019 of lateral branches in adults was one of three growth metrics used to compare colonists to established treeline individuals.

We used a linear mixed effects model with watershed as a random factor to compare colonist and established treeline populations.

```
colonist.CAG <- read.csv("./CAG2019.branch.extensionCutler.csv") %>%
    dplyr::mutate(Source="colonist",
                  Watershed,
                  Site,
                  Branch.avg.mm)

estab.treeline.CAG <- read.csv("./CAG2019.branch.extensionBRTL.csv") %>%
    dplyr::mutate(Source="established treeline",
                  Watershed,
                  Site,
                  Branch.avg.mm)

CAG <- rbind(colonist.CAG,estab.treeline.CAG) %>%
    dplyr::mutate(Watershed=paste(Source,Watershed))

table(CAG$Source)
```

```
##
##             colonist established treeline
##                   17                  455
```

```
aggregate(Branch.avg.mm~Watershed,
                    data=CAG,
                    FUN=length) %>%
    dplyr::mutate(Watershed,
                  Sample.size=Branch.avg.mm,
                  .keep="none")
```

```
##                    Watershed Sample.size
## 1  colonist Amakomanak Creek           1
## 2            colonist Cutler           5
## 3        colonist Hunt River           5
## 4     colonist Kaluich Creek           1
## 5         colonist WF Cutler           5
## 6    established treeline AG          59
## 7    established treeline AL          60
## 8    established treeline AM          40
## 9    established treeline BO           5
## 10   established treeline CL          10
## 11   established treeline EL          10
## 12   established treeline HF          60
## 13   established treeline KE          60
```

```
## 14   established treeline KU          61
## 15   established treeline OM          60
## 16   established treeline SA          10
## 17   established treeline TA          10
## 18   established treeline TI          10
```

Because there were many more sample trees from BRTL with more watersheds and more trees within watersheds, we used the **lmer()** function in package *lme4* as it handles unbalanced designs well.

We are interested in the t value of the fixed factor "Source." A log-likelihood, drop in deviance test suggests that fixed effect of "Source" as colonist or established treeline is significant.

```
CAG.mod.null <- lmer(log(Branch.avg.mm)~ (1|Watershed/Site),
                                      data=CAG,
                                      REML=F)

CAG.mod <- lmer(log(Branch.avg.mm)~Source+(1|Watershed/Site),
                                      data=CAG,
                                      REML=F)

anova(CAG.mod.null,CAG.mod)
```

```
## Data: CAG
## Models:
## CAG.mod.null: log(Branch.avg.mm) ~ (1 | Watershed/Site)
## CAG.mod: log(Branch.avg.mm) ~ Source + (1 | Watershed/Site)
##              npar    AIC    BIC  logLik deviance Chisq Df Pr(>Chisq)
## CAG.mod.null    4 305.78 322.41 -148.89   297.78
## CAG.mod         5 295.84 316.62 -142.92   285.84 11.94  1  0.0005494 ***
## ---
## Signif. codes:  0 '***' 0.001 '**' 0.01 '*' 0.05 '.' 0.1 ' ' 1
```

5.1.1 Setting REML = T and rerunning we extract the fixed factor coefficients and the $t-$value for reporting.

```
CAG.mod <- lmer(log(Branch.avg.mm)~Source+(1|Watershed),
                                      data=CAG,
                                      REML=T)

summary(CAG.mod)
```

```
## Linear mixed model fit by REML ['lmerMod']
## Formula: log(Branch.avg.mm) ~ Source + (1 | Watershed)
##    Data: CAG
##
## REML criterion at convergence: 351.3
##
## Scaled residuals:
##      Min       1Q   Median       3Q      Max
## -2.66008 -0.66354  0.00384  0.64864  2.92976
##
## Random effects:
##  Groups    Name        Variance Std.Dev.
##  Watershed (Intercept) 0.08361  0.2892
##  Residual              0.11146  0.3339
```

```
## Number of obs: 472, groups:  Watershed, 18
##
## Fixed effects:
##                             Estimate Std. Error t value
## (Intercept)                   3.5985     0.1610  22.351
## Sourceestablished treeline  -0.6788     0.1813  -3.745
##
## Correlation of Fixed Effects:
##             (Intr)
## Srcstblshdt -0.888
```

In Fig. 3d for the comparison of colonist (C) and established treeline (E) CAG we constructed the means as the estimates for "(Intercept)" and "(Intercept)" + "Sourceestablished treeline" for colonist CAG and established treeline CAG respectively. Error bars used the means ±2 × "Std. Error" respectively.

5.2 Linear mixed effects model comparing foliar nutrients

As with CAG, we applied linear mixed effects models with watershed as a random effects to test the null hypothesis that mean concentrations of nitrogen and phosphorous were the same in colonist and established treeline populations. Because of sample pooling before chemical analysis, the design is more balanced than for CAG.

```
Nutrients <- read.csv("./needle.NP.2019.csv") %>%
    dplyr::mutate(Watershed=paste(Source,Watershed))

table(Nutrients$Source)
```

```
##
##             colonist established treeline
##                   20                   51
```

```
aggregate(perN~Watershed,
                    data=Nutrients,
                    FUN=length) %>%
    dplyr::mutate(Watershed,
                 Sample.size=perN,
                 .keep="none")
```

```
##                   Watershed Sample.size
## 1  colonist Akamoakak Creek           3
## 2           colonist Cutler           3
## 3       colonist Hunt River           5
## 4    colonist Kaluich Creek           3
## 5        colonist WF Cutler           6
## 6   established treeline AG           6
## 7   established treeline AL           6
## 8   established treeline AM           4
## 9   established treeline BO           1
## 10  established treeline CL           2
## 11  established treeline EL           2
## 12  established treeline HF           6
## 13  established treeline KE           6
## 14  established treeline KU           6
## 15  established treeline OM           6
## 16  established treeline SA           2
```

```
## 17  established treeline TA           2
## 18  established treeline TI           2
```

5.2.1 Nitrogen concentration

```
N.mod <- lmer(perN~Source+(1|Watershed),
                 data=Nutrients,REML=T)
summary(N.mod)
```

```
## Linear mixed model fit by REML ['lmerMod']
## Formula: perN ~ Source + (1 | Watershed)
##    Data: Nutrients
##
## REML criterion at convergence: -45.4
##
## Scaled residuals:
##     Min      1Q  Median      3Q     Max
## -1.8314 -0.6303  0.1141  0.6657  3.9102
##
## Random effects:
##  Groups    Name        Variance  Std.Dev.
##  Watershed (Intercept) 0.0004468 0.02114
##  Residual              0.0270543 0.16448
## Number of obs: 71, groups:  Watershed, 18
##
## Fixed effects:
##                            Estimate Std. Error t value
## (Intercept)                 1.28245    0.03808  33.674
## Sourceestablished treeline -0.15050    0.04499  -3.345
##
## Correlation of Fixed Effects:
##             (Intr)
## Srcstblshdt -0.847
```

5.2.2 Phosphorous concentration

```
P.mod <- lmer(perP~Source+(1|Watershed),
                    data=Nutrients,REML=T)

summary(P.mod)
```

```
## Linear mixed model fit by REML ['lmerMod']
## Formula: perP ~ Source + (1 | Watershed)
##    Data: Nutrients
##
## REML criterion at convergence: -292.4
##
## Scaled residuals:
##      Min       1Q   Median       3Q      Max
## -2.08348 -0.58125 -0.02304  0.54012  2.41202
##
## Random effects:
```

```
##  Groups    Name        Variance  Std.Dev.
##  Watershed (Intercept) 0.0002056 0.01434
##  Residual              0.0005942 0.02438
## Number of obs: 70, groups:  Watershed, 18
##
## Fixed effects:
##                              Estimate Std. Error t value
## (Intercept)                  0.163338   0.008577   19.05
## Sourceestablished treeline -0.036782   0.010160   -3.62
##
## Correlation of Fixed Effects:
##             (Intr)
## Srcstblshdt -0.844
```

5.2.3 N15:N ratio

```
N15.mod <- lmer(I(d15N/perN)~Source+(1|Watershed),
                        data=Nutrients,REML=T)

summary(N15.mod)
```

```
## Linear mixed model fit by REML ['lmerMod']
## Formula: I(d15N/perN) ~ Source + (1 | Watershed)
##    Data: Nutrients
##
## REML criterion at convergence: 286.7
##
## Scaled residuals:
##     Min      1Q  Median      3Q     Max
## -3.2596 -0.3886  0.0295  0.6167  2.3027
##
## Random effects:
##  Groups    Name        Variance Std.Dev.
##  Watershed (Intercept) 0.7376   0.8589
##  Residual              2.9001   1.7030
## Number of obs: 71, groups:  Watershed, 18
##
## Fixed effects:
##                            Estimate Std. Error t value
## (Intercept)                 -2.8711     0.5473  -5.246
## Sourceestablished treeline  -2.2744     0.6497  -3.500
##
## Correlation of Fixed Effects:
##             (Intr)
## Srcstblshdt -0.842
```

6 Relative Growth Rate (RGR) Comparison Between Colonist and Established Treeline Populations

Juvenile relative growth rate (RGR) from 2015-2020 of the stem leader was calculated for both colonist and established treeline populations as described in **SI Calc. 3.**

6.1 Linear mixed effects models comparing RGR

Here we compare RGR between the two source populations using the linear mixed effects model function **lmer()** with these variable assignments:

1. ln(RGR) as the response variable ("growth.rate").
2. ln(height) in 2015 as a covariate ("log(Initial.cm)").
3. Random intercepts by field site ("Field.Site").
4. Fixed factor of interest: colonist or established treeline juveniles ("Source")).

```
BRTL <- read.csv("./GrowthRates_NoatakBasin.csv")
NoaBasin <- read.csv("./GrowthRates_BRTL.csv")
Big.dat <- rbind(BRTL,NoaBasin)
```

6.1.1 The sample sizes for each population source:

```
table(Big.dat$Source)
```

```
##
##              colonist established treeline
##                   300                  271
```

6.1.2 The sample sizes for each population source by field site:

```
table(Big.dat$Field.Site,
            Big.dat$Source)
```

```
##
##                     colonist established treeline
##    AGL                     0                    6
##    AGR                     0                   10
##    ALL 3                   0                    5
##    AmakCutlerRidge        19                    0
##    AmakE                  79                    0
##    AmakW                 195                    0
##    AML3                    0                   15
##    AMR1                    0                   14
##    CLL                     0                   13
##    CLR                     0                    8
##    Cutler                  7                    0
##    HFL                     0                   17
##    HFR                     0                   12
##    KEL1                    0                   12
##    KER1                    0                   12
##    KUL1                    0                   12
```

```
##    KUR1                 0                   13
##    OML1                 0                    6
##    OMR1                 0                   11
##    PhilipSmith          0                   60
##    TAL1                 0                   12
##    TAR1                 0                   12
##    TIL1                 0                    9
##    TIR1                 0                   12
```

6.1.3 Additive model: set REML = F

First, an additive model assuming the response of RGR to Source of a juvenile is shown only in the intercept. It assumes that the slope of "log(growth.rate)" on "log(Initial.cm)" is the same for both populations.

```
mod.A <- lmer(log(growth.rate)~log(Initial.cm)+Source +
                            (1|Field.Site),
                        data=Big.dat,REML=F,
                        subset=Initial.cm > 0)
```

6.1.4 Interaction model: set REML = F

Second, an interaction model assuming the response of RGR to Source of a juvenile is shown both in the intercept and in the slope of "log(growth.rate)" on "log(Initial.cm)".

```
mod.I <- lmer(log(growth.rate)~log(Initial.cm)*Source +
                            (1|Field.Site),
                        data=Big.dat,REML=F,
                        subset=Initial.cm > 0)
```

6.1.5 Compare the two models

```
AIC(mod.A) %>% round(1)
```

```
## [1] 359.9
```

```
AIC(mod.I) %>% round(1)
```

```
## [1] 342.2
```

$\Delta\texttt{AIC} = \texttt{AIC}(\texttt{mod.A}) - \texttt{AIC}(\texttt{mod.I}) = 17.7$, so interaction model is better.

A Likelihood-ratio test shows the interaction model drops the deviance significantly more than expected:

```
anova(mod.A,mod.I)
```

```
## Data: Big.dat
## Subset: Initial.cm > 0
## Models:
## mod.A: log(growth.rate) ~ log(Initial.cm) + Source + (1 | Field.Site)
## mod.I: log(growth.rate) ~ log(Initial.cm) * Source + (1 | Field.Site)
##       npar    AIC    BIC  logLik deviance  Chisq Df Pr(>Chisq)
## mod.A    5 359.91 381.65 -174.96   349.91
## mod.I    6 342.17 368.25 -165.08   330.17 19.744  1  8.853e-06 ***
## ---
## Signif. codes:  0 '***' 0.001 '**' 0.01 '*' 0.05 '.' 0.1 ' ' 1
```

6.2 Interaction model fixed effects coefficients with REML = T

gives truer, more conservative (i.e. bigger) standard errors of fixed effects coefficients.

```
mod.I.REML <- lmer(log10(growth.rate)~log10(Initial.cm)*Source +
                           (1|Field.Site),
                        data=Big.dat,REML=T,
                        subset=Initial.cm > 0)

(Intercept.C <- fixef(mod.I.REML)[1])
```

```
## (Intercept)
##  -0.6002489
```

```
(Slope.C <- fixef(mod.I.REML)[2])
```

```
## log10(Initial.cm)
##        -0.2000881
```

```
(Intercept.E <- fixef(mod.I.REML)[3])
```

```
## Sourceestablished treeline
##                -0.01894191
```

```
(Slope.E <- fixef(mod.I.REML)[4])
```

```
## log10(Initial.cm):Sourceestablished treeline
##                                   -0.1381284
```

```
summary(mod.I.REML)
```

```
## Linear mixed model fit by REML ['lmerMod']
## Formula: log10(growth.rate) ~ log10(Initial.cm) * Source + (1 | Field.Site)
##    Data: Big.dat
##  Subset: Initial.cm > 0
##
## REML criterion at convergence: -601
##
## Scaled residuals:
##     Min      1Q  Median      3Q     Max
## -6.0711 -0.5448  0.1233  0.6478  2.7658
##
## Random effects:
##  Groups     Name        Variance Std.Dev.
##  Field.Site (Intercept) 0.01116  0.1056
##  Residual               0.01800  0.1342
## Number of obs: 571, groups:  Field.Site, 24
##
## Fixed effects:
##                                               Estimate Std. Error t value
## (Intercept)                                   -0.60025    0.06512  -9.217
## log10(Initial.cm)                             -0.20009    0.02473  -8.091
## Sourceestablished treeline                    -0.01894    0.07455  -0.254
## log10(Initial.cm):Sourceestablished treeline -0.13813    0.03095  -4.463
##
## Correlation of Fixed Effects:
##             (Intr) l10(I. Srcstt
## lg10(Intl.) -0.539
```

```
## Srcstblshdt -0.874  0.471
## lg10(I.):St  0.430 -0.799 -0.586
```

```
Big.dat %>% ggplot(aes(x=log10(Initial.cm),
            y=log10(growth.rate),col=Source)) +
    geom_point() +
    scale_x_continuous("Height in 2015 (cm)",
                       breaks=log10(c(1,3,10,30,100)),
                       labels=c(1,3,10,30,100)) +
    scale_y_continuous("RGR (per year)",
                       breaks=log10(c(0.01,0.03,0.1,0.3)),
                       labels=c(0.01,0.03,0.1,0.3)) +
    geom_smooth(formula = y ~ x, method="loess",
                       linetype="dotted") +
    geom_abline(aes(intercept = Intercept.C, slope = Slope.C),
                       colour="red") +
    geom_abline(aes(intercept = Intercept.C+Intercept.E, slope = Slope.C+Slope.E),
                       colour="blue")
```

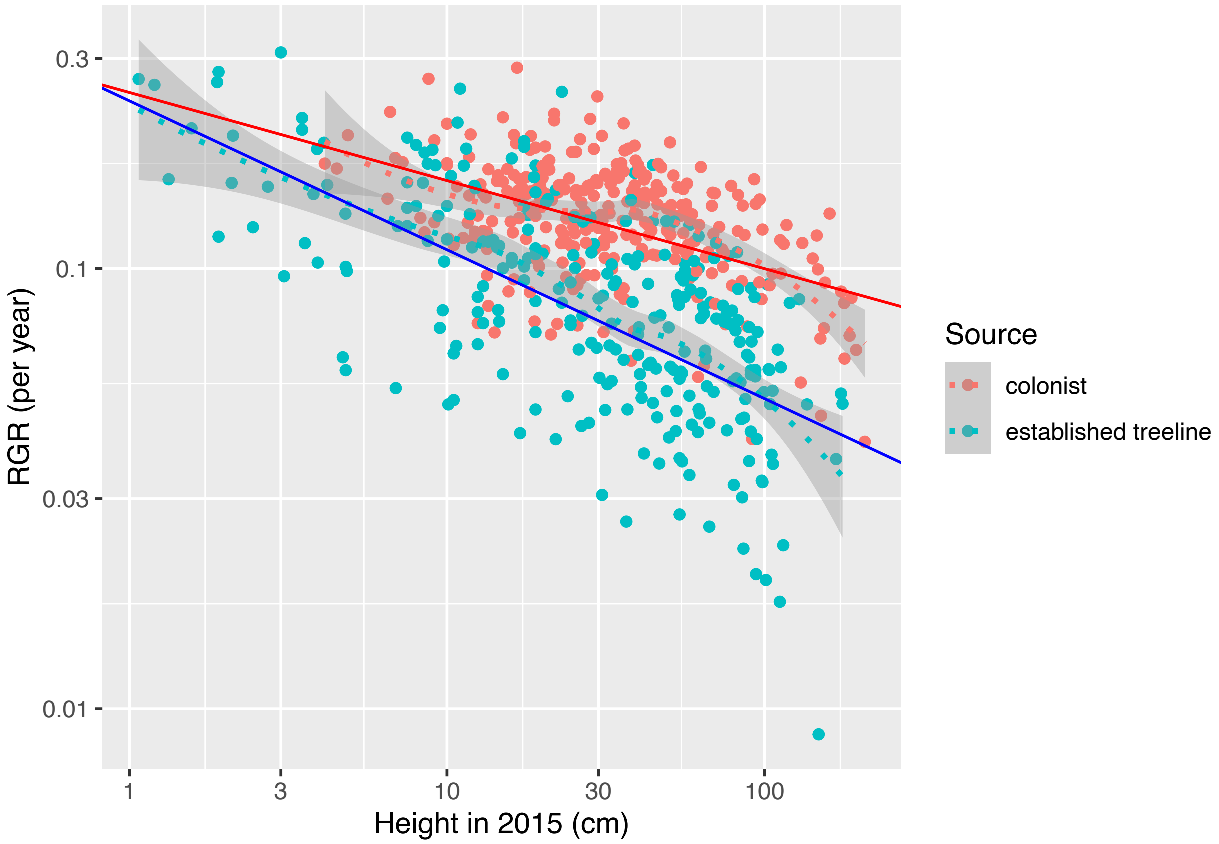

Relative growth rate (RGR) of juveniles from colonist (red) and established treeline (blue) populations by total height (cm) in 2015. Lines give best fit linear mixed effects model of log RGR on log height.

6.2.1 How much greater is RGR for 80-160 cm colonists

than similar sized juveniles at established treelines?

```r
Big.dat %>%
    filter(Initial.cm <= 160 & Initial.cm >= 80) %>%
    dplyr::select(Source,growth.rate) %>%
    group_by(Source) %>%
    summarize(mean.RGL=mean(growth.rate)) %>%
    dplyr::mutate(ratio=mean.RGL/min(mean.RGL))
```

```
## # A tibble: 2 x 3
##   Source               mean.RGL ratio
##   <chr>                   <dbl> <dbl>
## 1 colonist               0.103   1.90
## 2 established treeline   0.0544  1
```

7 Tree Ring Correlation with July Air Temperatures

Divergence–the lack of a positive correlation between tree ring indices and air temperature–is frequently observed[34,35] in treeline populations of Arctic Alaska during the latter half of the 10th century.

Consider two trees, one a "positive responder" to temperature (*i.e.*, positive correlation between tree ring indices and temperature) and the other a "negative responder" to temperature (*i.e.*, negative correlation between tree ring indices and temperature and so showing diveregence). Under similar environmental conditions including warming, the positive responder will grow more rapidly.

We show that nearly all colonists with cross-dated increment cores were positive responders with mean daily July temperatures in Kotzebue.

7.1 Relationship between mean daily July temperatures in the AOI and Kotzebue

Lapse rates are the regression slopes of the relationship between temperature and elevation. Here we use remote automated weather stations (RAWS) from the Baird Mountains and Kotzebue instrumental temperature records and regress them against elevation. The strong consistent linearity in the relationship suggests that temperatures are well correlated among stations and so would be the AOI, since its elevation is within the range of RAWS.

We use Kotzebue because it is longest record near to the AOI. Kotzebue mean daily July temperatures and those from three remote automated weather stations (RAWS) show stable lapse rates over the eight years of records, 2012-2019. The AOI is located geographically between the two highest RAWS, with Imelyak to the east and Kaluich to the west. The oldest trees aged in the AOI are located at about 450 m asl, intermediate in elevation between Salmon (366 m asl) and Kaluich (758 m asl).

```
station.temps <- read.csv("./station_temps.csv") %>% filter(Year < 2020)
colnames(station.temps)[1] <- "Station"
unique(station.temps$Year) %>% sort()
```

```
## [1] 2012 2013 2014 2015 2016 2017 2018 2019
```

Elevations by station:

```
station.temps %>%
    group_by(Station) %>%
    dplyr::summarize(elevation.m.asl=round(unique(Elevation_masl))) %>%
    arrange(elevation.m.asl) %>%
    kable(caption="Elevations in m asl for Kotzebue and remote
          automated weather stations (RAWS)
          in Baird Mountains.")
```

Elevations in m asl for Kotzebue and remote automated weather stations (RAWS) in Baird Mountains.

Station	elevation.m.asl
Kotzebue	9
Salmon	366
Kaluich	758
Imelyak	1088

Over the eight years of recorded temperatures reported here (2012-2019) the mean daily July temperature of Kotzebue was strongly correlated with that of each RAWS.

```
stations.by.year <- station.temps %>%
   dplyr::select(!Elevation_masl) %>%
   pivot_wider(names_from=c(Station), values_from=JulyTemp_C)
```

Correlation between Kotzebue July air temperature and the RAWS nearest to Kotzebue and lowest in elevation, Salmon:

```
cor.test(~Kotzebue+Salmon,data=stations.by.year)
```

```
##
##  Pearson's product-moment correlation
##
## data:  Kotzebue and Salmon
## t = 5.3833, df = 2, p-value = 0.03282
## alternative hypothesis: true correlation is not equal to 0
## 95 percent confidence interval:
##  0.08651334 0.99933817
## sample estimates:
##       cor
## 0.9671823
```

Correlation between Kotzebue July air temperature and the RAWS nearest to the sample of tree cores from the AOI and intermediate in elevation, Kaluich:

```
cor.test(~Kotzebue+Kaluich,data=stations.by.year)
```

```
##
##  Pearson's product-moment correlation
##
## data:  Kotzebue and Kaluich
## t = 7.0366, df = 4, p-value = 0.00215
## alternative hypothesis: true correlation is not equal to 0
## 95 percent confidence interval:
##  0.6853582 0.9959681
## sample estimates:
##       cor
## 0.9619006
```

Correlation between Kotzebue July air temperature and the RAWS farthest from Kotzebue and highest in elevation, Imelyak:

```
cor.test(~Kotzebue+Imelyak,data=stations.by.year)
```

```
##
##  Pearson's product-moment correlation
##
## data:  Kotzebue and Imelyak
## t = 8.4505, df = 4, p-value = 0.001074
```

```
## alternative hypothesis: true correlation is not equal to 0
## 95 percent confidence interval:
##  0.7683777 0.9971696
## sample estimates:
##       cor
## 0.9731172
```

These correlations are best captured by yearly lapse rates, which show strong consistency in their values across years.

Using a mixed model approach with year as random intercept capturing the annual variability in Kotzebue air temperature and considering the fixed effect intercept as the mean temperature in Kotzebue (i.e., at sea level) during the eight years:

```
lapse.lm <- lmer(JulyTemp_C~I(Elevation_masl/1000)+(1|Year),
                 data=station.temps,subset=Year < 2020)
(intercept <- round(fixef(lapse.lm)[1],2))
```

```
## (Intercept)
##        14.7
```

Here is the mean lapse rate in °C km^{-1} of elevation over the eight years,

```
(lapse <- round(fixef(lapse.lm)[2],2))
```

```
## I(Elevation_masl/1000)
##                  -5.41
```

and its standard error:

```
(lapse.se <- round(coef(summary(lapse.lm))[2,2],2))
```

```
## [1] 0.15
```

7.2 Lapse rates in Baird Mountains of Brooks Range

```
station.temps %>% subset(Year < 2020) %>%
  mutate(Year=factor(Year)) %>%
  ggplot(aes(x=Elevation_masl,
      y=JulyTemp_C,
      group=Year,
      col=Year)) +
  scale_y_continuous(expression(Station~mean~daily~July~temperature~(degree*C)),
        sec.axis=dup_axis(name=NULL)) +
  scale_x_continuous("Elevation (m asl)",
       breaks=c(9,366,460,758,1088),
       labels=c(9,366,"AOI",758,1088),
       sec.axis=sec_axis(name="RAWS station",~.,
                         breaks=c(9,366,758,1088),
        labels=c("Kotzebue","Salmon","Kaluich","Imelyak"))) +
```

```r
 geom_point() +
 geom_smooth(formula=y~x,
                      method="lm",
            se=F) +
 theme_gray(base_size = 14) +
   geom_abline(intercept=intercept,
                           slope=lapse/1000,
linetype="dotted") + labs(title=bquote(Dotted~line:~Mean~July~lapse~rate==.(lapse)*degree*C~km^-1)) +
theme(panel.grid.major = element_blank(),
      panel.grid.minor = element_blank(),
       panel.background = element_blank(),
      axis.line = element_line(colour = "black"))
```

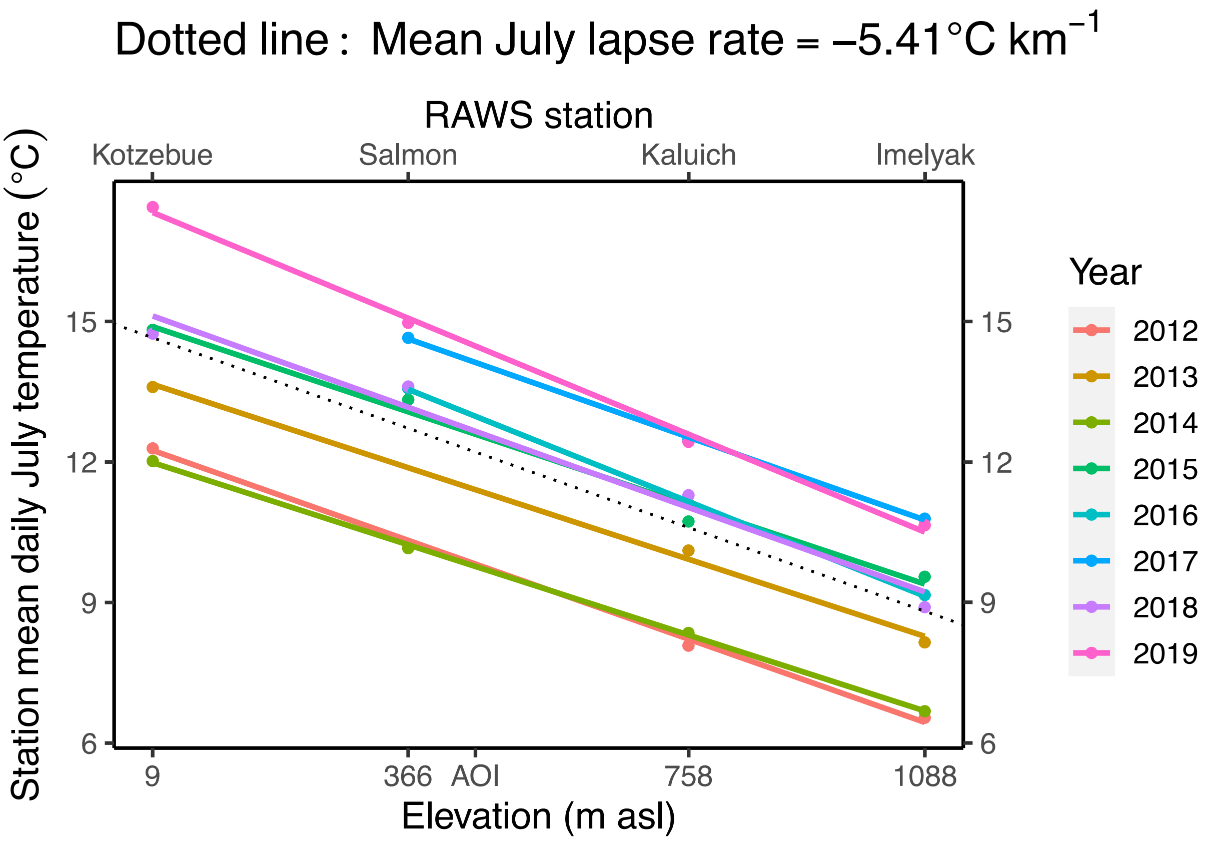

Mean daily July temperature plotted against elevation for remote area weather stations (RAWS) in Baird Mountains and Kotzebue by year. Dotted line plots fixed effects estimate from linear mixed effects model with year as a random factor.

The strong relationship between RAWS temperatures and elevations, with the AOI elevation well within the range of RAWS elevations, implies a very strong correlation between Kotzebue and AOI mean daily July temperatures, similar to those between Kotzebue and the three RAWS ($0.96 \le r \le 0.97$).

7.2.1 Northwest Alaska's warmest recorded decade

We highlight the difference in radial growth during the period 1989-2019 (included in the warmest three consecutive decades in the Kotzebue July temperature record) with the previous time period in the tree ring time series.

```
kotz.july <- read.csv("./Kotzebue_Julytemps.csv")
 colnames(kotz.july)[1] <- "year"

 kotz.july %>%
 dplyr::mutate(decade=cut(year,c(1930,1960,1990,2020), dig.lab = 4)) %>%
aggregate(July_mean_C~decade, FUN=function(x) round(mean(x),1),
            data=.)  %>%
   kable(caption="Mean of July temperature
         in Kotzebue for each of three decades.")
```

Mean of July temperature in Kotzebue for each of three decades.

decade	July_mean_C
(1930,1960]	11.6
(1960,1990]	12.1
(1990,2020]	13.0

7.2.2 Pre-whitening the July temperature series

We also attempt to "pre-whiten" the July temperature series to remove any autocorrelation present in that series. A pre-whitened series is just the residuals from a best-fit autoregressive model. We apply this transformation to the tree ring series below (which typically display persistence or "memory", *i.e.*, the growth that a tree does in a given year is related to growth in the previous year(s)) before running correlations with temperature. For statistical coherence we apply pre-whitening to both the tree-ring and the temperature data, as climate data can also show persistence.

First, construct a "pre-whitened" (AR residual) temperature series, using same method as tree ring series: `detrend.series()` from the `dplR` package. Take a look at the raw series and the "pre-whitened" series.

```
 kotz.july$July_mean_C_AR <- detrend.series(kotz.july$July_mean_C, method = "Ar", make.plot = F)
```

Next, plot the two series and compare in the figure below:

```
raw.temp <- ggplot(kotz.july,
           aes(year, July_mean_C)) + geom_line()
ar.temp <- ggplot(kotz.july,
           aes(year, July_mean_C_AR)) + geom_line()
plot_grid(raw.temp,
          ar.temp,
          align = "hv",
          axis = "tblr",
          nrow = 2)
```

The temperature series and the pre-whitened series look similar, but the AR version is scaled. The perfect correlation confirms this:

```
 cor(kotz.july$July_mean_C, kotz.july$July_mean_C_AR)
```

```
## [1] 1
```

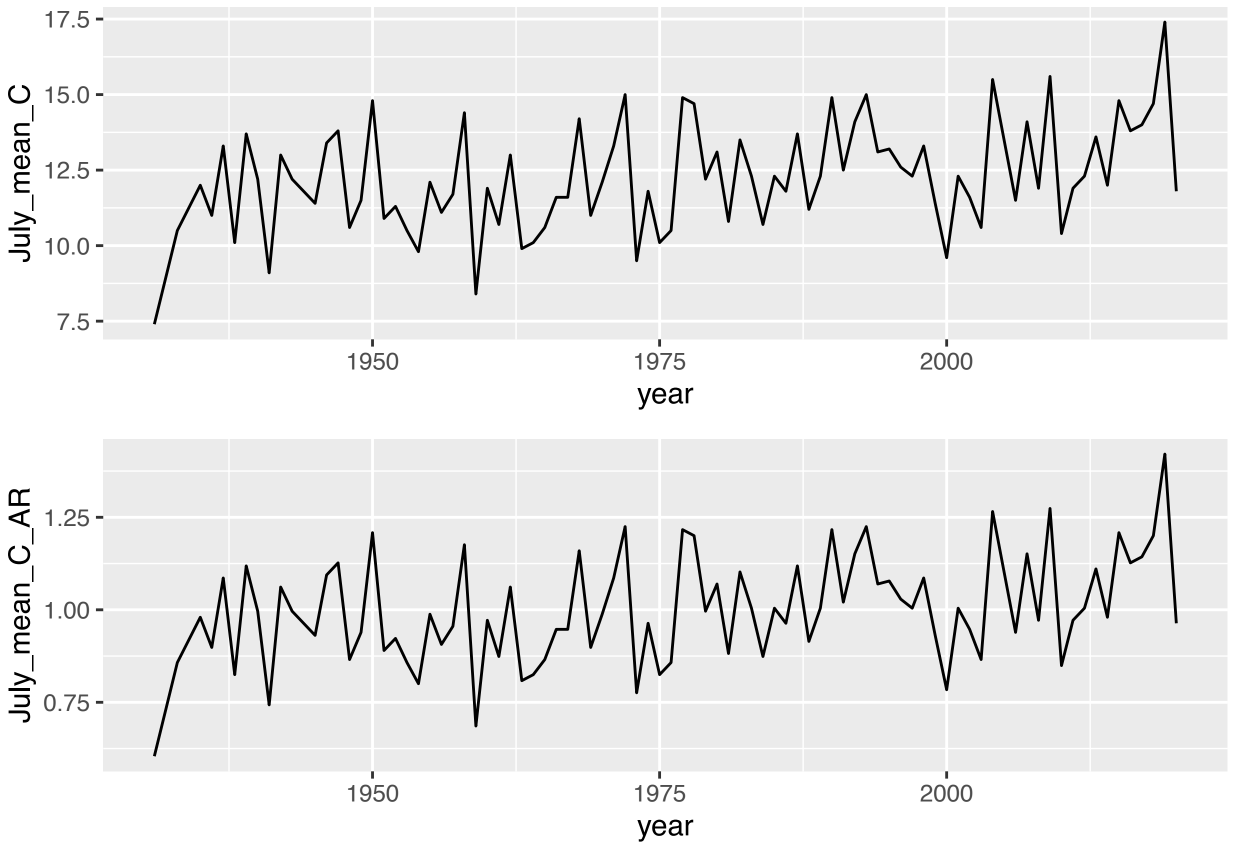

Raw Kotzebue temperature series (top) and residuals from best fit autoregressive model (bottom). The series differ only by a scaling factor because Kotzebue july temperature already approximates white noise.

The function `detrend.series()` from the `dplR` package doesn't provide details about its AR model, but we know that `detrend.series()` uses the defaults of the base R function `ar()`, which applies the "Yule-Walker" method and AR order selection by AIC.

```
(ar.mod1 <- ar(kotz.july$July_mean_C))
```

```
##
## Call:
## ar(x = kotz.july$July_mean_C)
##
##
## Order selected 0  sigma^2 estimated as  3.135
```

This AR order of 0 (zero) suggests the temperature series is approximately white noise: there is no memory in the series.

To confirm this is not a result of the Yule-Walker method, we apply a second method, "mle", as suggested in Cowpertwait, P.S. and Metcalfe, A. V. (2009) Introductory Time Series with R, Springer, NY.

```
(ar.mod2 <- ar(kotz.july$July_mean_C,
               method = "mle"))
```

```
##
## Call:
## ar(x = kotz.july$July_mean_C, method = "mle")
##
##
## Order selected 0  sigma^2 estimated as  3.1
```

This is the same AR order = 0 as found with the "Yule-Walker" method.

A partial correlogram (autocorrelation values with lag after removing the effects of shorter lags) for the July temperature data shows a significant autocorrelation at lag = 10 (the blue dotted line shown in the next figure is significance).

```
pacf(kotz.july$July_mean_C)
```

However, model complexity is too high for a lag = order = 10 as shown when AIC values are plotted against order, showing that the lowest AIC is at lag = order = 0.

```
aic.mod1 <- as.data.frame(ar.mod1$aic)
aic.mod1$order <- as.numeric(rownames(aic.mod1))
colnames(aic.mod1)[1] <- "AIC"

ggplot(aic.mod1, aes(order, AIC)) + geom_line() + theme_bw() +
  xlab("AR order")
```

Because the July temperature series already approximates white noise and does not contain substantial autocorrelation, we retain the "raw" temperature series in subsequent analyses.

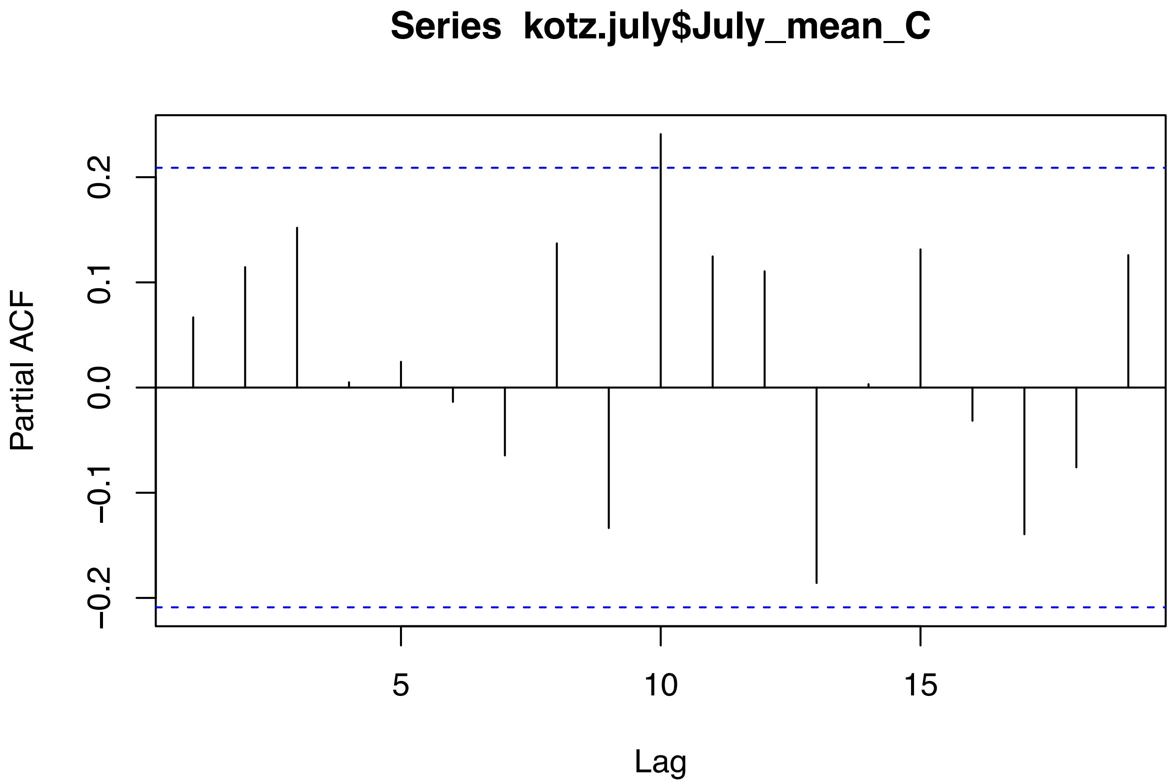

Partial autocorrelation on vertical axis with lag on horizontal axis for the Kotzebue temperature series. The dotted lines give significance at the p = 0.05 level for the correlations.

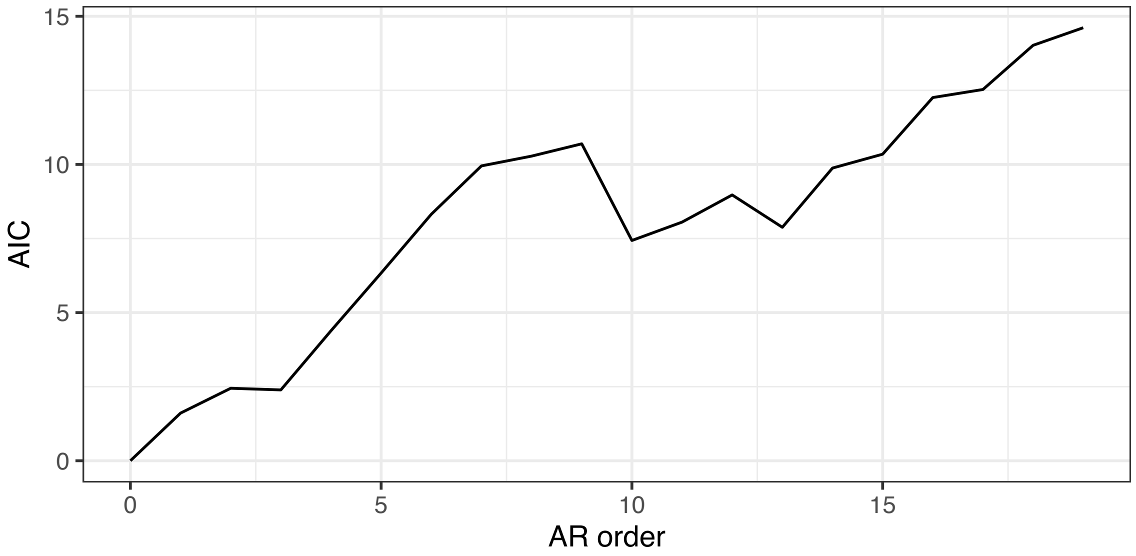

AIC as a function of lag for autoregressive model for Kotzebue time series showing the lowest AIC occurs with no lag.

7.3 Tree ring indices

We begin by reading in tree ring data and ordering the series. A "spaghetti plot" of raw ring widths is shown in ED. Fig 4b.

```
cut <- read.rwl("./cutler.trees.rwl") %>%
    .[,order(colnames(.))]
```

```
## Attempting to automatically detect format.
## Assuming a Tucson format file.

## Warning in readLines(con): incomplete final line found on './cutler.trees.rwl'

## There does not appear to be a header in the rwl file
## There are 150 series
## 1        GAIA        1954    2019    0.01
## 2        Tr02        1977    2019    0.01
## 3        Tr03        1983    2019    0.01
## 4        Tr04        1995    2019    0.01
## 5        Tr05        1980    2019    0.01
## 6        Tr07        1977    2019    0.01
## 7        Tr08        1955    2019    0.01
## 8        Tr09        1983    2019    0.01
## 9        Tr10        1991    2019    0.01
## 10       Tr11        1999    2019    0.01
## 11       Tr12        1977    2019    0.01
## 12       Tr13        1998    2019    0.01
## 13       Tr14        2004    2019    0.01
## 14       Tr15        1987    2019    0.01
## 15       Tr16        1979    2019    0.01
## 16       Tr17        1981    2019    0.01
## 17       Tr18        1986    2019    0.01
## 18       Tr19        1981    2019    0.01
## 19       Tr20        2005    2019    0.01
## 20       Tr21        2008    2019    0.01
## 21       Tr22        2004    2019    0.01
## 22       Tr23        2005    2019    0.01
## 23       Tr24        1997    2019    0.01
## 24       Tr25        2006    2019    0.01
## 25       Tr26        2008    2019    0.01
## 26       Tr27        1992    2019    0.01
## 27       Tr28        2000    2019    0.01
## 28       Tr30        2006    2019    0.01
## 29       Tr31        1998    2019    0.01
## 30       Tr32        2004    2019    0.01
## 31       Tr33        1989    2019    0.01
## 32       Tr34        1955    2019    0.01
## 33       Tr35        1985    2019    0.01
## 34       Tr36        1992    2019    0.01
## 35       Tr37        1994    2019    0.01
## 36       Tr38        1989    2019    0.01
## 37       Tr39        1993    2019    0.01
## 38       Tr40        2000    2019    0.01
```

```
## 39        Tr41          1983    2019   0.01
## 40        Tr42          1996    2019   0.01
## 41        Tr43          1996    2019   0.01
## 42        Tr44          1980    2019   0.01
## 43        Tr45          2005    2019   0.01
## 44        Tr46          1999    2019   0.01
## 45        Tr47          1986    2019   0.01
## 46        Tr48          1998    2019   0.01
## 47        Tr49          1953    2019   0.01
## 48        Tr50          1992    2019   0.01
## 49        Tr51          1962    2019   0.01
## 50        Tr52          1946    2019   0.01
## 51        Tr60          1951    2019   0.01
## 52        T01           1995    2020   0.01
## 53        T02           1989    2020   0.01
## 54        T03           1980    2020   0.01
## 55        T04           1979    2020   0.01
## 56        T05           1977    2020   0.01
## 57        T06           1999    2020   0.01
## 58        T07           1933    2020   0.01
## 59        T08           1964    2020   0.01
## 60        T09           1985    2020   0.01
## 61        T10           1983    2020   0.01
## 62        T100          1971    2017   0.01
## 63        T101          1991    2020   0.01
## 64        T102          1968    2020   0.01
## 65        T11           1976    2020   0.01
## 66        T12           1952    2020   0.01
## 67        T13           1952    2020   0.01
## 68        T14           1967    2020   0.01
## 69        T16           1962    2020   0.01
## 70        T17           1999    2020   0.01
## 71        T18           1976    2020   0.01
## 72        T19           1993    2020   0.01
## 73        T20           1983    2020   0.01
## 74        T21           1954    2020   0.01
## 75        T22           1986    2020   0.01
## 76        T23           1947    2020   0.01
## 77        T25A          1982    2020   0.01
## 78        T25B          1972    2020   0.01
## 79        T26           1966    2020   0.01
## 80        T27           1969    2020   0.01
## 81        T28           1982    2020   0.01
## 82        T29           1988    2020   0.01
## 83        T30           1993    2020   0.01
## 84        T31           1984    2020   0.01
## 85        T32           1988    2020   0.01
## 86        T33           1983    2020   0.01
## 87        T34           1985    2020   0.01
## 88        T35           1981    2020   0.01
## 89        T36           1996    2020   0.01
## 90        T37           1996    2020   0.01
## 91        T38           1979    2020   0.01
## 92        T39           1984    2020   0.01
```

```
## 93        T40          1996    2020   0.01
## 94        T41          1988    2020   0.01
## 95        T42          1952    2020   0.01
## 96        T43          1983    2020   0.01
## 97        T44          1991    2020   0.01
## 98        T45          1995    2020   0.01
## 99        T46          1984    2020   0.01
## 100       T47          1990    2020   0.01
## 101       T48          1989    2020   0.01
## 102       T49          1967    2020   0.01
## 103       T50          1979    2020   0.01
## 104       T51          1979    2020   0.01
## 105       T52          1990    2020   0.01
## 106       T53          1963    2020   0.01
## 107       T54          1978    2020   0.01
## 108       T55          1982    2020   0.01
## 109       T57          1980    2020   0.01
## 110       T58          1984    2020   0.01
## 111       T59          1968    2020   0.01
## 112       T60          1984    2020   0.01
## 113       T61          1965    2020   0.01
## 114       T62          1988    2020   0.01
## 115       T63          1982    2020   0.01
## 116       T64          1975    2020   0.01
## 117       T65          1985    2020   0.01
## 118       T66          1992    2020   0.01
## 119       T67          1963    2020   0.01
## 120       T68          1967    2020   0.01
## 121       T69          1988    2020   0.01
## 122       T70          1980    2020   0.01
## 123       T71          1978    2020   0.01
## 124       T72          1982    2020   0.01
## 125       T73          1991    2020   0.01
## 126       T74          2009    2020   0.01
## 127       T75          2002    2020   0.01
## 128       T76          1991    2020   0.01
## 129       T77          1993    2020   0.01
## 130       T78          1979    2020   0.01
## 131       T79          1980    2020   0.01
## 132       T80          1984    2020   0.01
## 133       T81          1974    2020   0.01
## 134       T82          1979    2020   0.01
## 135       T83          1996    2020   0.01
## 136       T84          1984    2020   0.01
## 137       T85          1989    2020   0.01
## 138       T86          1971    2020   0.01
## 139       T87          1991    2020   0.01
## 140       T88          1990    2020   0.01
## 141       T89          1955    2020   0.01
## 142       T90          1998    2020   0.01
## 143       T91          1995    2020   0.01
## 144       T92          1997    2020   0.01
## 145       T94          1980    2020   0.01
## 146       T95          1973    2020   0.01
```

```
## 147      T96          2004    2020    0.01
## 148      T97          1996    2020    0.01
## 149      T98          1983    2020    0.01
## 150      T99          1988    2020    0.01
```

The main purpose of analysis here is to compare growth rates across years in light of Kotzebue temperature.

The chron() function in the dplR package computes a mean of the series allowing a visualization of average growth (line plot) and a view of sample depth (gray area) through time.

```
chron(cut, prefix = "cut") %>%
  crn.plot(abline.pos   = NULL, ylab = "Ring width (mm)")
```

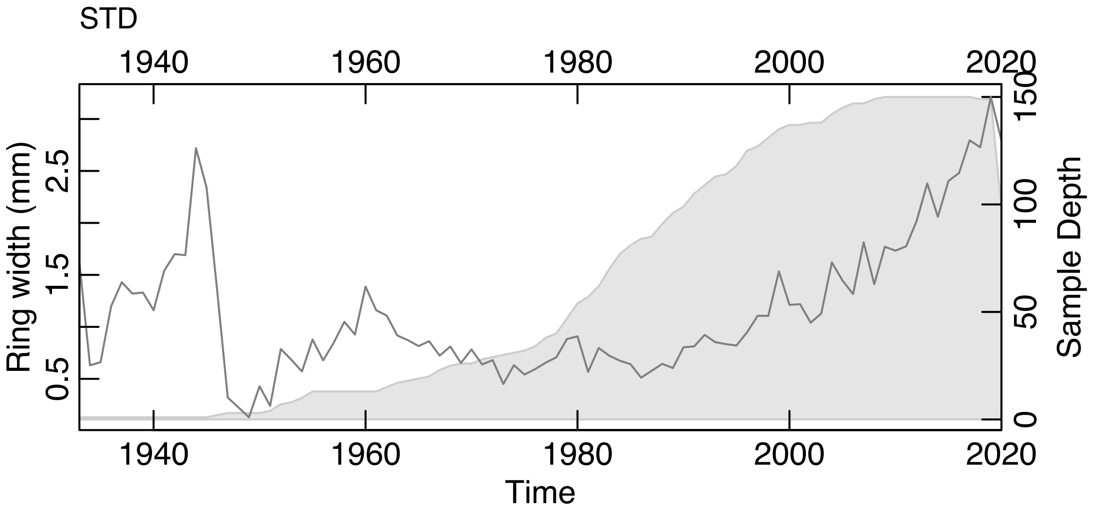

Mean ring width (gray line) and tree-core sample size (gray polygon) by year.

7.4 Ages corrected by core height

We used relative growth rates (**SI Calc. 3**) of small spruce ≥ 20 cm tall to estimate ages at height in (**SI Calc. 2**), then applied the relationship to core height.

```
cut.ages <- read.csv("./Cutler_treeAges and pith info.csv") %>%
    mutate(id=TreeID,
        nRings,
        .keep="none")
ext.ages <- read.csv("./AgingIncrementCoreHeight_V3.csv") %>%
    mutate(id=Core_ID,
                 Height_m,
                 Establishment_yr,
                 .keep="none") %>%
    merge(cut.ages,
                by = "id")
```

7.3.1 Compute tree ring indices

Compute basal area index (BAI) using `bai.in()` where output is in mm^2.

```
cut.dat <- read.csv("./cutler.trees.d2pith_CORRECTED.csv") %>%
.[order(.$series),]
names(cut.dat)[names(cut.dat)=="series"] <- "id"

 cut.bai <- bai.in(cut,
    d2pith = cut.dat)
```

Compute pre-whitened (autoregressive residual) series using `detrend()` in the `dplR` package.

```
suppressWarnings(cut.ar <- detrend(cut, method = "Ar"))
## throws warnings for 5 series that Ar fits are not all positive.
```

Add year for converting to long format.

```
cut.bai$year <- as.numeric(rownames(cut.bai))
cut$year <- as.numeric(rownames(cut))
cut.ar$year <- as.numeric(rownames(cut.ar))
```

Pivot as longer data frames and convert BAI from mm^2 to cm^2

```
cut.bai.df <- pivot_longer(cut.bai,
        cols = -year,
        values_to = "bai.mm2",
        names_to = "id") %>%
    data.frame() %>%
    dplyr::mutate(bai.cm2=bai.mm2/100) %>%
    na.omit()
cut.df <- pivot_longer(cut,
        cols = -year,
        values_to = "rw.mm",
        names_to = "id") %>%
    data.frame() %>%
na.omit()
cut.ar.df <- pivot_longer(cut.ar,
        cols = -year,
        values_to = "rwi.ar",
        names_to = "id") %>%
    data.frame() %>%
na.omit()
```

Combine data frames of basal area index, ring width, and auto-regression residuals as one data frame.

```
cut.df <- mutate(cut.bai.df,
        rw.mm=cut.df$rw.mm) %>%
    merge(cut.ar.df,
        by = c("year","id"),
        all.x = T)
```

Sum ring-widths cumulatively by individual tree id as the tree grows to define radius by year in mm.

```
##  package data.table can make indexed assignments by reference
cut.dT <- data.table(cut.df) %>%
    .[order(year),
radius.mm := cumsum(rw.mm),
by=list(id)]
```

7.5 Data frame of ring indices and Kotzebue July air temperatures

The following code merges several dataframes and adds the distance to pith to each radius converted radius to cm (originally in mm), then merges with tree ages that have been adjusted by the estimated age that the tree reached the height of the increment core hole (median 5 cm above ground).

The dataframe retains only the variables needed for correlations and merges with 1937-2020 July air temperatures from Kotzebue.

```
Cutler.rings.kotz.C <-
    join(as.data.frame(cut.dT),
        cut.dat, by = "id") %>%
    dplyr::mutate(radius.cm=
        (radius.mm + d2pith)/10) %>%
    merge(ext.ages[,c("id",
        "Height_m",
        "Establishment_yr",
        "nRings")], all.x = T) %>%
    dplyr::mutate(id,
        Height_m,
        Establishment_yr,
        nRings,
        year,
        bai.cm2,
        ln_bai.cm2=log(bai.cm2),
        radius.cm,
rwi.ar,
        .keep="none") %>%
    merge(kotz.july,
by="year")
```

Nine trees lack core height above-ground measures, or other data needed for the next steps, and are removed.

```
missing.dat <- Cutler.rings.kotz.C %>%
    filter(is.na(Establishment_yr)) %>%
    summarize(no.core.ht=length(unique(id)))
 missing.dat$no.core.ht
```

```
## [1] 9
```

7.6 Growth trends by cambial age

We also show BAI by cambial age (i.e., years from the pith) for the first 30 years for each age class, juvenile vs. adult.

Thick lines give means by age class. The thin lines show individual series. Both show growth in contemporary juveniles is unique to the last 30 years among all trees.

Older trees tend to lack innermost rings missed during field coring and so are often missing the youngest cambial ages.

```
bai.df <- merge(as.data.frame(cut.dT),
                             cut.dat, by = "id")
# add the ages and establishment year as well
bai.df <- merge(bai.df, cut.ages[,c("id","Age","Est.yr","nRings")])
# Add age classes
bai.df$age.class <- ifelse(bai.df$Age > 30, "adult (>30y)", "juvenile (<30y)") # Get a secondary age cl
bai.df$cambial.age <- bai.df$year - bai.df$Est.yr
bai.df$Cohort <- cut(bai.df$Est.yr, seq(1900,2020,30), dig.lab = 4) # Re-convert bai from mm2 to cm2
bai.df$bai.cm2 <- bai.df$bai.mm2*0.01
# Aggregate to means and se of the two metrics of growth - bai and AR
bai.agg <- aggregate(bai.cm2 ~ year + age.class, data = bai.df, FUN = length)
ar.agg <- aggregate(rwi.ar ~ year + age.class, data = bai.df, FUN = length)
rw.agg <- aggregate(rw.mm ~ year + age.class, data = bai.df, FUN = length)
colnames(bai.agg)[3] <- "samp.depth"
colnames(ar.agg)[3] <- "samp.depth"
colnames(rw.agg)[3] <- "samp.depth"
# Means
bai.agg$bai.cm2 <- aggregate(bai.cm2 ~ year + age.class, data = bai.df, FUN = mean)[,3]
ar.agg$rwi.ar <- aggregate(rwi.ar ~ year + age.class, data = bai.df, FUN = mean)[,3]
rw.agg$rw.mm <- aggregate(rw.mm ~ year + age.class, data = bai.df, FUN = mean)[,3]
# 2 se
bai.agg$bai.cm2.se <- aggregate(bai.cm2 ~ year + age.class, data = bai.df, FUN = function(x)sd(x)/sqrt(l
ar.agg$rwi.ar.se <- aggregate(rwi.ar ~ year + age.class, data = bai.df,
FUN = function(x)sd(x)/sqrt(length(x)))[,3]
rw.agg$rw.mm.se <- aggregate(rw.mm ~ year + age.class, data = bai.df,
FUN = function(x)sd(x)/sqrt(length(x)))[,3]
agg <- merge(bai.agg, ar.agg[,!(colnames(ar.agg) %in% "samp.depth")],
    by = c("year","age.class"), all.x = T)
agg <- merge(agg, rw.agg[,!(colnames(rw.agg) %in% "samp.depth")],
    by = c("year","age.class"), all.x = T)

# Convert to long format
agg.long <- pivot_longer(agg[,!(colnames(agg) %in% c("bai.cm2.se","rwi.ar.se","rw.mm.se"))],
   cols = c("bai.cm2","rwi.ar","rw.mm"),
   names_to = "growth.metric", values_to = "growth.value")

agg.long[,6] <- pivot_longer(agg[,!(colnames(agg) %in% c("bai.cm2","rwi.ar","rw.mm"))],
cols = c("bai.cm2.se","rwi.ar.se","rw.mm.se"),
   names_to = "growth.metric.se", values_to = "growth.value.se")[,5]

agg.long$growth.metric <- factor(agg.long$growth.metric, levels = c("rw.mm","rwi.ar", "bai.cm2"))

# Function to compute mean ± 2 se in stat_summary() in ggplot
mean_2se <- function(x){
y <- mean(x)
se <- sd(x)/sqrt(length(x))
ymin <- y - 2*se
ymax <- y + 2*se
```

```
data.frame(y,ymin,ymax)
}

# All trees
ggplot(bai.df[bai.df$cambial.age <= 30,],
       aes(cambial.age, bai.cm2)) +
  geom_line(aes(cambial.age, bai.cm2, group = id, color = age.class),
            alpha = 0.5) +
  stat_summary(geom = "line", aes(cambial.age, bai.cm2, id = age.class),
               color = "black",
               fun.data = mean_2se, size = 1.5) +
  stat_summary(geom = "line", aes(cambial.age, bai.cm2, color = age.class),
               fun.data = mean_2se, size = 1) +
  scale_color_manual(name = "Contemporary age class",
        values=c(cbPalette[4],cbPalette[5])) +
  theme_bw() +
  scale_x_continuous(breaks = seq(0,30, by = 5)) +
  theme(#axis.text.x = element_text(angle = 90),
        legend.position = c(0.2,0.8),
        panel.grid.major = element_blank(),
        panel.grid.minor = element_blank(),
        panel.background = element_blank(),
        axis.line = element_line(colour = "black")) +
  xlab("Cambial age (years from pith)") +
  ylab(bquote("BAI ("*cm^2*")"))
```

```
## Warning: Ignoring unknown aesthetics: id
```

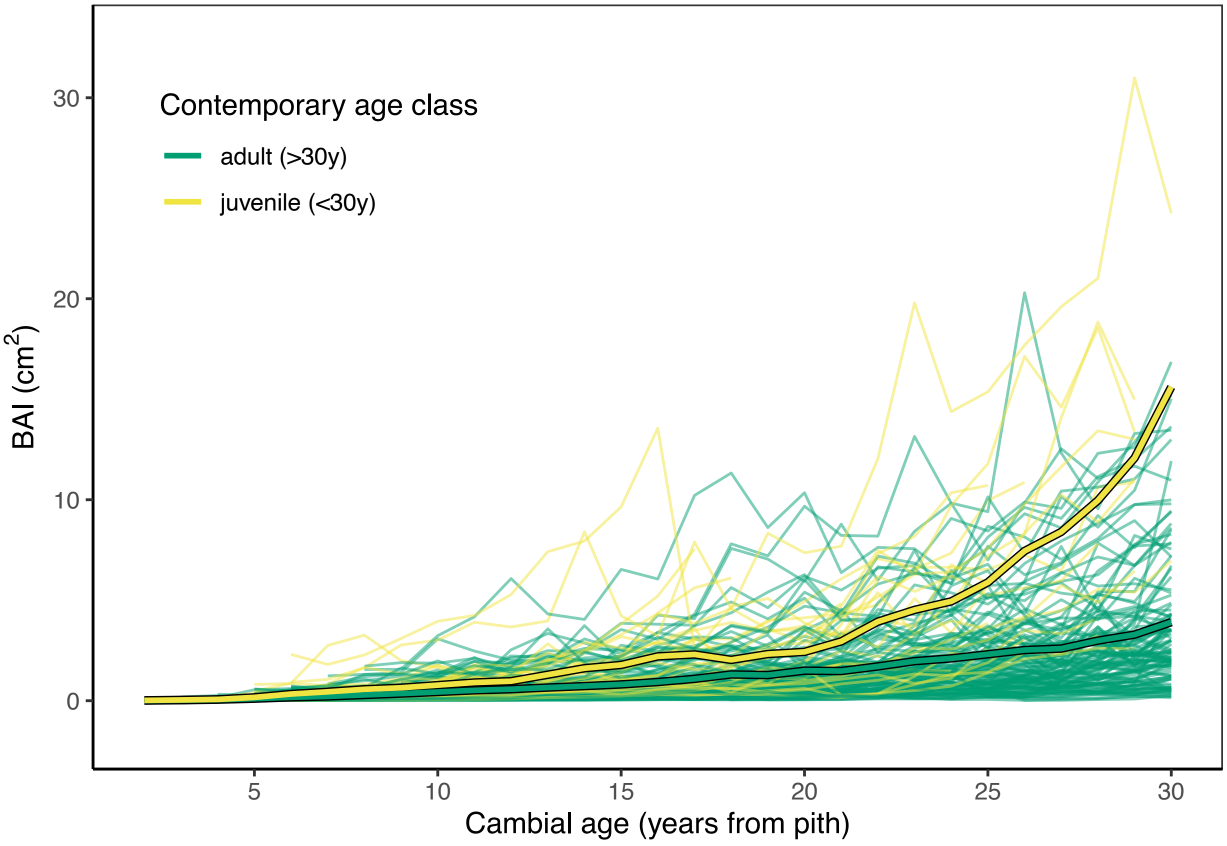

7.7 Cambial age growth across thirty-year cohorts

Here the BAI plotted by age group to more clearly discern the cambial age-related patterns. It appears that over four thirty-year periods, all trees grew at about the same rate for the first 5 years. Then two series of three decades, the early colonial decades and the most recent decades, continued to grow rapidly while the other two slowed somewhat, and finally at about age 20, the most recent cohort of trees diverged as fastest growing juveniles over the record.

```
bai.df.juv <- bai.df %>% filter(cambial.age <= 30) %>% mutate(Cohort=factor(Cohort))

sample.size <- aggregate(id ~ Cohort, data = bai.df.juv, FUN = \(x) length(unique(x)))
colnames(sample.size)[2] <- "n"
sample.size$n <- paste0("n = ", sample.size$n)
sample.size$cambial.age = 15
sample.size$bai.cm2 = 25


ggplot(bai.df.juv, aes(x=cambial.age, y=bai.cm2, group = Cohort)) +
    facet_wrap(~Cohort) +
    geom_smooth() +
    geom_point(size=0.1,alpha=0.5) +
    geom_text(data=sample.size, aes(x=cambial.age, y=bai.cm2, label=n)) +
      theme_bw() +
    theme(#panel.grid.major = element_blank(),
        #panel.grid.minor = element_blank(),
        panel.background = element_blank(),
```

```
        axis.line = element_line(colour = "black")) +
        labs(x="Cambial age (y)",y=expression(BAI~(cm^2)))
```

```
## `geom_smooth()` using method = 'gam' and formula 'y ~ s(x, bs = "cs")'
```

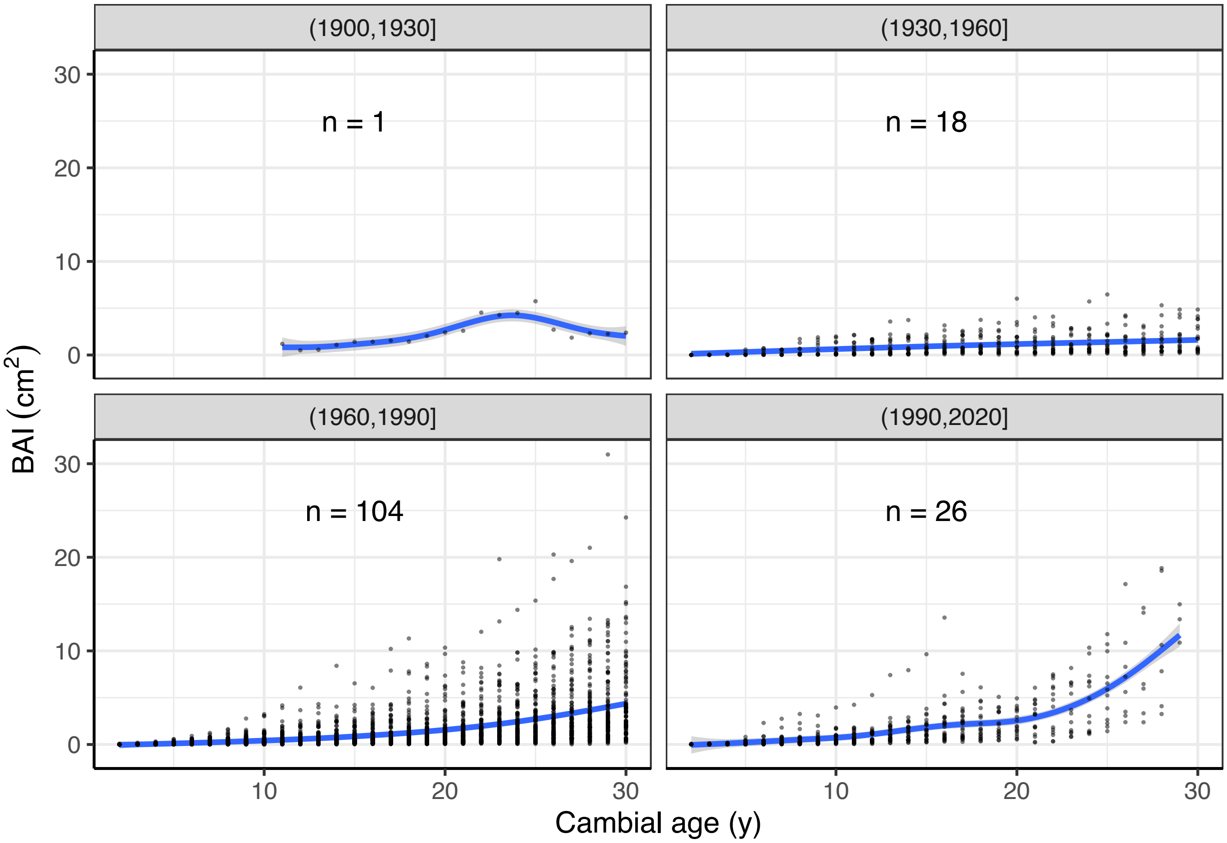


```
#ggsave("./BAI_30_cambial_age.pdf",height=4,width=4)
bai.df %>% filter(cambial.age <= 30) %>% mutate(Cohort=factor(Cohort)) %>%
ggplot(aes(x=cambial.age, y=log10(bai.cm2), colour = Cohort)) +
# geom_line(aes(group = id), alpha = 0.25) +
    geom_point(size=0.1) +
        geom_smooth() +
    scale_x_continuous(breaks = seq(0,30, by = 5),
                                    lim=c(0,30)) +
        scale_y_continuous(breaks = c(-2,-1,0,1),
                                        labels=c(0.01,0.1,1,10)) +
    geom_vline(xintercept=c(5,10,15,20,25),
                        linetype=c("solid","dotted","dotdash","dashed","solid"),
                        colour=c("gray",rep("black",4))) +
    labs(x="Cambial age (y)",y=expression(BAI~(cm^2))) +
  theme_bw() +
    theme(panel.grid.major = element_blank(),
        panel.grid.minor = element_blank(),
        panel.background = element_blank(),
        axis.line = element_line(colour = "black"))
```

```
## `geom_smooth()` using method = 'gam' and formula 'y ~ s(x, bs = "cs")'
```

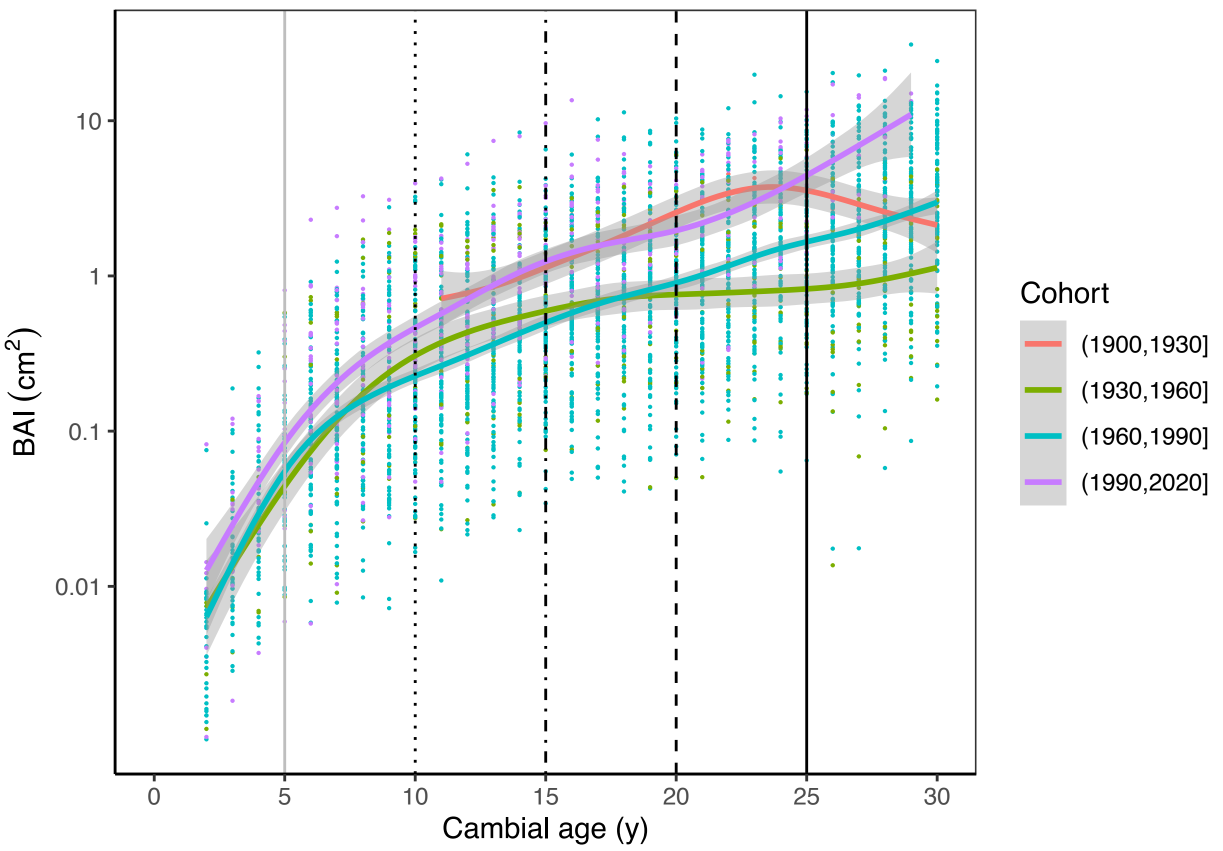

We can also examine each tree's first 30 years of growth by plotting against calendar year instead of cambial age.

```
ggplot(bai.df[bai.df$cambial.age <= 30,],
       aes(year, bai.cm2)) +
  geom_line(aes(year, bai.cm2, group = id, color = age.class),
            alpha = 0.5) +
  stat_summary(geom = "line", aes(year, bai.cm2, id = age.class),
               color = "black",
               fun.data = mean_2se, size = 1.5) +
  stat_summary(geom = "line", aes(year, bai.cm2, color = age.class),
               fun.data = mean_2se, size = 1) +
  scale_color_manual(name = "Contemporary age class", values=c(cbPalette[4],cbPalette[5])) +
  theme_bw() +
  scale_x_continuous(breaks = seq(1930,2020, by = 10), limits = c(1930,2019)) +
  theme(#axis.text.x = element_text(angle = 90),
        legend.position = c(0.2,0.8),
        panel.grid.major = element_blank(),
        panel.grid.minor = element_blank(),
        panel.background = element_blank(),
        axis.line = element_line(colour = "black")) +
  xlab("Year") +
  ylab(bquote("BAI ("*cm^2*")"))
```

```
## Warning: Ignoring unknown aesthetics: id
```

```
## Warning: Removed 8 rows containing non-finite values (stat_summary).
```

```
## Removed 8 rows containing non-finite values (stat_summary).
```

```
## Warning: Removed 8 row(s) containing missing values (geom_path).
```

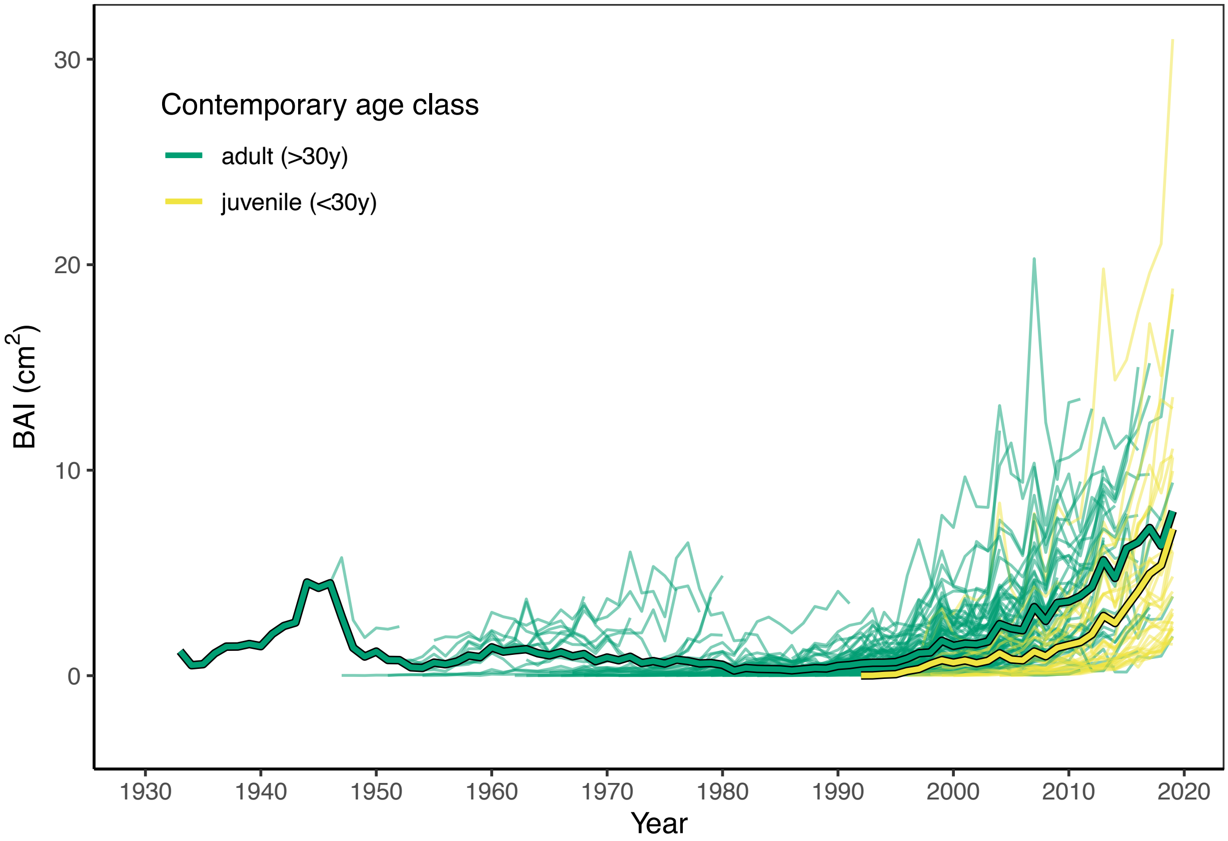

7.8 Correlations of tree ring metrics with July air temperature

We measured 150 individual tree ring series. However, 9 cores were missing core heights so could not be assigned to an establishment year, leaving 141 samples that could be confidently assigned to adult or juvenile age classes.

The Kotzebue air temperatures are likely the best available proxy for the Cutler River basin because the lapse rates appear quite stable, at least over the last decade (as shown above).

For each individual tree ring series, this function calculates the correlations between the two ring indices (BAI and AR) and Kotzebue July temperature (both raw data and pre-whitened - same as AR - data).

```
Pearson.r.stats <- function(only.these) {
    IDs <- unique(only.these$id)
how.many <- length(IDs)
## Vectors to fill
Establishment_yr <- rep(NA,how.many)
    r.BAI <- r.AR <- rep(NA,how.many)
    p.BAI <- p.AR <- rep(NA,how.many)
    series.len <- nRings <-rep(NA,how.many)
## A loop to fill all the vectors
for(i in 1:how.many) {
```

```
    temp <- subset(only.these,
        id == IDs[i])
    Establishment_yr[i] <-
        max(temp$Establishment_yr)
  test.BAI <-
    cor.test(~ln_bai.cm2+July_mean_C_AR,data=temp)
    test.AR <-
        cor.test(~rwi.ar+July_mean_C_AR,data=temp)
    r.BAI[i] <- test.BAI$estimate
    p.BAI[i] <- test.BAI$p.value
    r.AR[i] <- test.AR$estimate
    p.AR[i] <- test.AR$p.value
    nRings[i] <- max(temp$nRings)
    series.len[i] <-
        sum(!is.na(temp$bai.cm2))
    }
data.frame(id=IDs,
    Establishment_yr,
    series.len,
    nRings,
    r.BAI,
    p.BAI,
    r.AR,
p.AR) }
```

7.8.1 Ring-width indices of juveniles (estimated age < 30 y) and correlations with temperature since 1989

Rather than separate juveniles by height, here they are separated by age: establishment year after 1989.

```
Juvees.stats <- subset(Cutler.rings.kotz.C,
        Establishment_yr >= 1989) %>%
    Pearson.r.stats() %>%
    dplyr::mutate(age.class="juvenile (<30 y)")
```

There are n = 15 individuals and include over 2/3 are above 2.5 tall: 1.68, 1.93, 2.09, 2.14, 2.37, 2.6, 2.6, 2.6, 2.7, 2.9, 3.3, 3.5, 3.6, 3.9, 5.53.

7.9 Juveniles and temperature

The correlations between July Kotzebue air temperature and ring indices for juveniles are generally positive and relatively strong.

```
Juvees.stats  %>%
    summarize(n=nrow(.),
        posBAI=sum(r.BAI > 0),
        r.BAI.min=round(min(r.BAI),2),
        r.BAI.mean=round(mean(r.BAI),2),
        r.BAI.max=round(max(r.BAI),2),
        sigBAI=sum(p.BAI <= 0.05),
posAR=sum(r.AR > 0),
```

```
r.AR.min=round(min(r.AR),2),
r.AR.mean=round(mean(r.AR),2),
r.AR.max=round(max(r.AR),2),
        sigAR=sum(p.AR <= 0.05)) %>%
  kable(caption="Descriptive statistics for correlations, r,
        between juvenile tree-core indices (BAI nad AR) and
        Kotzebue July air temperature.")
```

Descriptive statistics for correlations, r, between juvenile tree-core indices (BAI nad AR) and Kotzebue July air temperature.

n	posBAI	r.BAI.min	r.BAI.mean	r.BAI.max	sigBAI	posAR	r.AR.min	r.AR.mean	r.AR.max	sigAR
15	15	0.22	0.46	0.64	9	15	0.31	0.53	0.81	10

All 15 juveniles show positive correlations between ring indices and July air temperature, with 9 BAI series and 10 AR series significant at the p <= 0.05 level.

Besides showing more series with significant correlations, the AR index had a higher mean correlation with July temperatures than BAI.

7.9.1 Ring-width indices of adults (estimated age > 30 y) and correlations with temperature since 1989

Here adults are defined in terms of establishment age being prior to 1989. In addition to selecting only adults, the correlations between ring width statistics and Kotzebue July air temperature are calculated only for the 30 years 1989-2019.

```
Adults.stats <- subset(Cutler.rings.kotz.C,
        Establishment_yr < 1989) %>%
    filter(year >= 1989) %>%
    Pearson.r.stats() %>%
    dplyr::mutate(age.class="adult (>30y)")
```

There were n = 125 individuals of those measured for height and with known establishment years, 119 were 2.5 m tall or taller.

7.10 Adults and temperature

Correlations between July Kotzebue air temperature and ring indices for adults were mostly positive but tended to be weaker than for juveniles.

```
Adults.stats  %>%
    summarize(n=nrow(.),
        posBAI=sum(r.BAI > 0),
                r.BAI.25=sum(r.BAI > 0.25),
        r.BAI.min=round(min(r.BAI),2),
        r.BAI.mean=round(mean(r.BAI),2),
        r.BAI.max=round(max(r.BAI),2),
                sigBAI=sum(p.BAI <= 0.05),
        posAR=sum(r.AR > 0),
```

```
                r.AR.25=sum(r.AR > 0.25),
        r.AR.min=round(min(r.AR),2),
        r.AR.mean=round(mean(r.AR),2),
        r.AR.max=round(max(r.AR),2),
                sigAR=sum(p.AR <= 0.05)) %>%
  kable(caption="Descriptive statistics for correlations, r,
        between adult tree-core indices (BAI nad AR) and
        Kotzebue July air temperature.")
```

Descriptive statistics for correlations, r, between adult tree-core indices (BAI nad AR) and Kotzebue July air temperature.

n	posBAI	r.BAI.25	r.BAI.min	r.BAI.mean	r.BAI.max	sigBAI	posAR	r.AR.25	r.AR.min	r.AR.mean	r.AR.max	sigAR
126	124	34	-0.03	0.23	0.71	16	124	99	-0.17	0.36	0.8	64

Of 125 adults, 123 show positive correlations between BAI and July air temperature and 123 positive correlations between AR and July air temperature, with 16 BAI series and 64 AR series significant at the p <= 0.05 level. Besides showing more series with significant correlations, the AR index had a higher mean correlation with July temperatures than the BAI index.

7.10.1 Graphical comparisons of adults, juveniles, BAI, and AR

Bind together and widen all the data

```
dat <- bind_rows(Adults.stats, Juvees.stats)
wide.dat <- data.frame(
    r =
        c(dat$r.BAI, dat$r.AR),
    index.type =
        c(rep("ln(BAI)", length(dat$r.BAI)),
        rep("AR", length(dat$r.AR))),
    age.class = c(dat$age.class, dat$age.class))
```

The adult time series from 1989 to 2019:

```
adults <- aggregate(
    cbind(bai.cm2, rwi.ar) ~ year,
data = Cutler.rings.kotz.C, subset = Establishment_yr < 1989,
FUN = function(x){c(m = mean(x), s = 2*sd(x)/sqrt(sum(!is.na(x))))}
)

means <- c(adults$bai.cm2[,1],adults$rwi.ar[,1])
variability <- c(adults$bai.cm2[,2],adults$rwi.ar[,2])
cutler.time.adult <-
    data.frame(age.class=rep("adult (>30y)",nrow(adults)),
        year=rep(adults$year,nrow(adults)),
        mean.index=means,
        min.index=means-variability,
        max.index=means+variability,
        index.type=c(rep("BAI",nrow(adults)),
```

```
            rep("AR",nrow(adults))))  %>%
    subset(year >= 1989 & year < 2019)
```

And the juveniles:

```
juveniles <- aggregate(
    cbind(bai.cm2, rwi.ar) ~ year,
        data = Cutler.rings.kotz.C,
        subset = Establishment_yr >= 1989,
    FUN=function(x){c(m = mean(x), s = 2*sd(x)/sqrt(sum(!is.na(x))))}
    )
means <- c(juveniles$bai.cm2[,1], juveniles$rwi.ar[,1])
variability <- c(juveniles$bai.cm2[,2], juveniles$rwi.ar[,2])
cutler.time.juveniles <-
    data.frame(age.class = rep("juvenile (<30y)", nrow(juveniles)),
        year = rep(juveniles$year, nrow(juveniles)),
        mean.index = means,
        min.index = means - variability,
        max.index = means + variability,
        index.type = c(rep("BAI", nrow(juveniles)),
            rep("AR", nrow(juveniles))))  %>%
    subset(year >= 1989 & year < 2019)
time.series.dat <- rbind(cutler.time.adult,cutler.time.juveniles)
```

7.11 Plotting distributions of correlations of BAI with July Kotzebue temperature

```
 wide.dat[wide.dat$index.type == "ln(BAI)",] %>%
     mutate(age.class=factor(age.class,
levels=c("juvenile (<30 y)","adult (>30y)"))) %>%
ggplot(aes(x=ring.index, y=age.class)) + labs(fill="Age class") + #facet_grid(row=var
scale_x_continuous(lim=c(-0.25,1), expand = c(0.01, 0)) +
 geom_density_ridges(
   aes(x = r,
           fill=age.class),
   alpha = .5,
 color = "black",
 from = -0.25, to = 1) +
 scale_fill_manual(values=c(cbPalette[5], cbPalette[4])) +
        #geom_vline(xintercept=0, linetype="dotted") +
     coord_cartesian(clip = "on") +
  scale_y_discrete(labels = NULL, breaks = NULL,
                                      expand = c(0.01, 0)) +
     labs(y = element_blank(),
             x="log(BAI) Correlation with July °C") +
     theme_bw() +
   theme_ridges(grid = FALSE) +
     theme(text = element_text(size = 19),
                legend.position="right") +
   theme(strip.background = element_blank()) +
    geom_vline(xintercept=0)
```

```
## Picking joint bandwidth of 0.0516
```

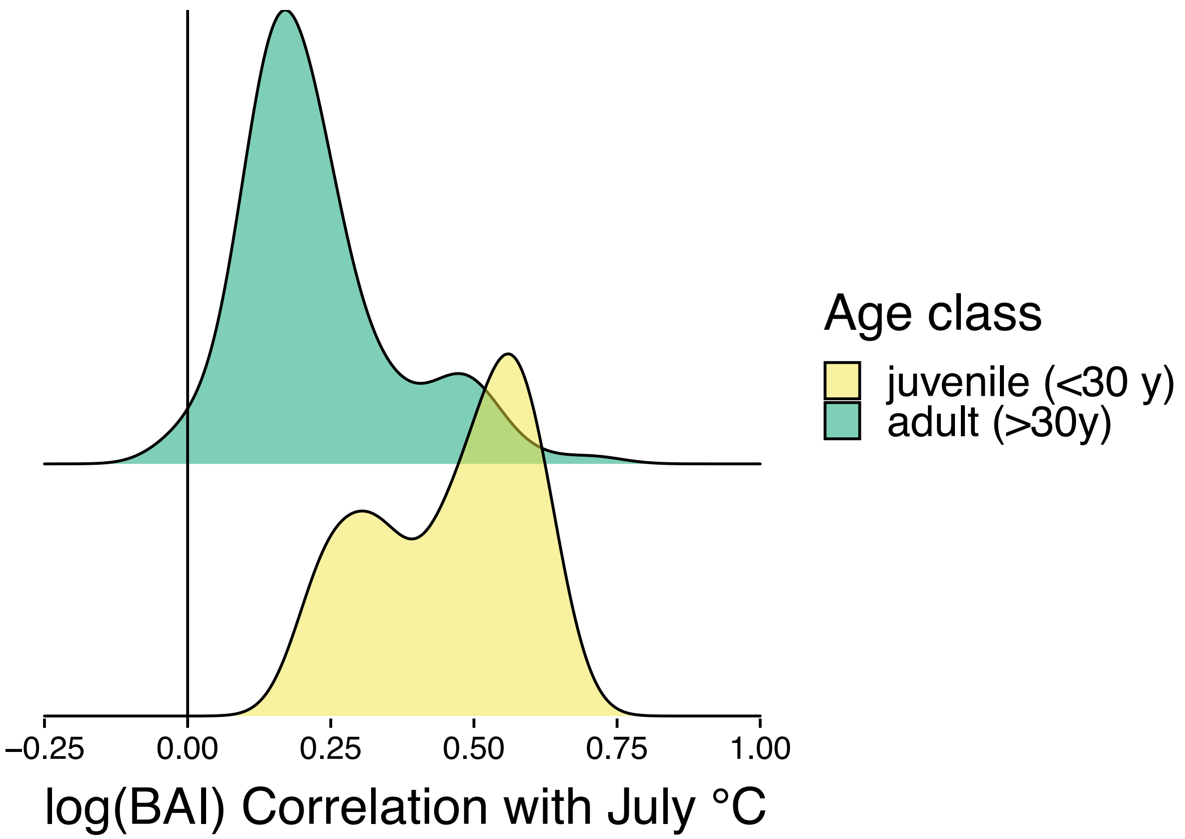

Distribution of correlation coefficient between ln(BAI) and temperature for juveniles (yellow) and adults (green).

8 Climate

8.1 PRISM

The Parameter-elevation Regressions on Independent Slopes Model (PRISM) group at Oregon State University produced a set of gridded climate products representing 30-year mean temperatures (1981-2010) and precipitation for Alaska[69]. We extracted July mean daily temperatures and the sum of November through March precipitation from the four Noatak watersheds of the AOI and the adjacent four Kobuk watersheds where the established treelines are located. In addition, we extracted the gridded values for any pixel containing colonists and any pixel containing BRTL.

First, a function for reading and reprojecting PRISM rasters:

```
prism.project <- function(fil)
{r <- raster(fil)
    extent(r)[1]  <- extent(r)[1] - 360
    extent(r)[2] <- extent(r)[2] - 360
    crs(r) <- CRS("+init=epsg:4269")
    crop(r,br.extent)
}
```

Some details:

```
load("br.extent.RData")
pal <- carto_pal(7,"Teal") ## color for maps
safe_colorblind_palette <- c("#88CCEE","#CC6677","#DDCC77",
        "#117733","#332288","#AA4499",
    "#44AA99","#999933","#882255",
        "#661100","#6699CC","#888888")
geog <- "+proj=longlat +ellps=WGS84 +datum=WGS84 +no_defs"
nad83 <- "+init=epsg:4269"
```

The Noatak watersheds (AOI) and the adjacent Kobuk watersheds:

```
HUC10.AOI <- c("Kaluich Creek","Cutler River",
                "Imelyak River","Amakomanak Creek")
HUC10.kobuk <- c("Akillik River","Hunt River",
                "Redstone River","Miluet Creek")

AOI.83.kob.noa <- readOGR("./AOI.shp",
            verbose=F) %>%
        spTransform(CRS(nad83)) %>%
    .[.@data$name %in% c(HUC10.AOI,HUC10.kobuk),]

noatak.watersheds <- AOI.83.kob.noa[AOI.83.kob.noa$Basin == "Noatak",]

kobuk.watersheds <- AOI.83.kob.noa[AOI.83.kob.noa$Basin == "Kobuk",]
```

The Brooks Range established treelines within the four Kobuk watersheds:

```
BRTL.points <- readOGR("./BRTL.AOI.shp",
            verbose=F) %>%
    spTransform(CRS(nad83))
```

The digitized shadow locations:

```
adult.colonists <- read.csv("./BestShadowData.csv") %>%
    mutate(Long=long,
        Lat=lat)

adult.colonists.nad83 <- SpatialPointsDataFrame(coords=
     adult.colonists[,c("Long","Lat")],
     data=adult.colonists[,c("Long","Lat")],
            proj4string=CRS(geog)) %>%
    spTransform(CRS(nad83))
```

The PRISM rasters

```
temp <- prism.project("./ak_tmean_1981_2010.07.txt")
    names(temp) <- "layer"


precip.11 <- prism.project("./ak_ppt_1981_2010.11.txt")
precip.12 <- prism.project("./ak_ppt_1981_2010.12.txt")
precip.1 <- prism.project("./ak_ppt_1981_2010.01.txt")
precip.2 <- prism.project("./ak_ppt_1981_2010.02.txt")
precip.3 <- prism.project("./ak_ppt_1981_2010.03.txt")
```

Summing the winter precipitation:

```
precip <- precip.3 + precip.2 + precip.1 +
                    precip.12 + precip.11
```

Stacking July temperature and November-March precip.

```
var.rs <- stack(c(temp,
        precip))
```

Extracting climate variables for envelope plots

First, AOI adult colonists, recording cell numbers so as to reduce redundancy.

```
vars.noa <- var.rs %>%
    crop(noatak.watersheds) %>%
  mask(noatak.watersheds) %>%
  raster::extract(adult.colonists.nad83, ## colonist locations
        cellnumbers=T)
```

Combine these PRISM pixel values with the data frame of colonists:

```
adult.colonists.nad83@data  <- cbind(adult.colonists.nad83@data,
        vars.noa)
```

There are

```
nrow(adult.colonists.nad83@data) %>%
    format(big.mark=",") %>%
        paste("adults")
```

```
## [1] "5,988 adults"
```

The climate envelope uses landscape pixels, not individual trees which would inflate the importance of some pixels:

```
adult.colonists <- aggregate(cbind(layer.1,
        layer.2)~cells,
```

```
        data=adult.colonists.nad83@data,
        FUN=mean) %>%
    mutate(layer.1, ## July C
        layer.2, ## winter snow
        Basin="Colonists",
        .keep="none")
nrow(adult.colonists) %>%
    format(big.mark=",") %>%
        paste("colonist pixels")
```

```
## [1] "708 colonist pixels"
```

In the data frame "adult.colonists" each row is a pixel with at least one adult colonist. Each pixel has a winter precipitation sum and a July mean temperature.

Extract pixels for Noatak AOI watersheds

```
vars.noa.H2Osheds.df <- var.rs %>%
    crop(noatak.watersheds) %>%
    mask(noatak.watersheds) %>%
    values() %>%
    data.frame %>%
    mutate(Basin="Noatak watersheds")
```

And for Kobuk watersheds

```
vars.kob.H2Osheds.df <- var.rs %>%
    crop(kobuk.watersheds) %>%
    mask(kobuk.watersheds) %>%
    values() %>%
    data.frame %>%
    mutate(Basin="Kobuk watersheds")
```

Repeat for BRTL

Kobuk BRTL:

```
###  Kobuk BRTL
vars.kob.treelines <- var.rs %>%
    crop(kobuk.watersheds) %>%
    mask(kobuk.watersheds) %>%
    raster::extract(BRTL.points,
        cellnumbers=T)

BRTL.points@data  <- cbind(BRTL.points@data,
        vars.kob.treelines)

BRTL <- aggregate(cbind(layer.1,
        layer.2)~cells,
        data=BRTL.points@data ,
        FUN=mean) %>%
    mutate(layer.1,
                 layer.2,
                 Basin="Established treelines",
                 .keep="none")
```

Note that BRTL occupies more pixels than the colonist population.

```
nrow(BRTL) %>%
    format(big.mark=",") %>%
        paste("treeline pixels")
```

```
## [1] "1,213 treeline pixels"
```

Because there are fewer colonist (1213) pixels and we wish to compare the climate envelopes among watersheds and sources of adult trees, we randomly select the same number of pixels from BRTL and watersheds as pixels among colonists.

```
seed <- 140
sample.pix <- function(df,seed=140){
    set.seed(seed)
    sample(nrow(df),nrow(adult.colonists))
}
```

```
dat.r <- rbind(BRTL[sample.pix(BRTL),],
        adult.colonists,
    vars.noa.H2Osheds.df[sample.pix(vars.noa.H2Osheds.df),],
        vars.kob.H2Osheds.df[sample.pix(vars.kob.H2Osheds.df),]) %>%
    mutate(Basin=factor(Basin,
        levels=c("Established treelines",
        "Colonists",
        "Kobuk watersheds",
        "Noatak watersheds")))
```

8.2 Constructing climate envelopes

```
# Not run
# dat.r %>% ggplot(aes(x=layer.2,
#                                      y=layer.1,
#                                      col=Basin)) +
# geom_density_2d_filled(contour_var = "ndensity") +
#   facet_wrap(vars(Basin)) +
#   guides(fill = "none") +
#   guides(col = "none")    +
#   ylim(8,16) +
#   xlim(110,170)+
#   geom_hline(yintercept=10,col="yellow")+
#   labs(x = "1980-2010 mean winter precipitation (mm)",
#       y = "1980-2010 mean July temperature (C)")
# ggsave('../../Figures/ComparingKobukNoatak4.pdf',
#             width = 4,
#             height = 4, units = 'in')
```

8.3 Kotzebue and the Chukchi Sea

8.3.1 Kotzebue mean daily July temperature

The Kotzebue, Alaska (162.5966° W, 66.8983° N) July temperature record is the most complete in the region and most complete of the summer months in Kotzebue, with 84 annual monthly records from 1937 to 2021.

Here we display the 1937-2020 record downloaded from NOAA (see Methods).

```
July <- read.csv("./KotzebueTemps Upto 2020.csv") %>%
  subset(Month ==7 ) %>%
  mutate(Year=Year,
      July.C=(TAVG-32)*5/9,
      .keep="none")
```

Are all the years between 1937 and 2020 in the record? Compare the "Year" values with the sequence from 1937 to 2020.

```
identical(seq(1937,2020),
                    July$Year)
```

```
## [1] TRUE
```

Are any July mean daily temperatures missing? No, all 84 are non-NA.

```
sum(!is.na(July$July.C))
```

```
## [1] 84
```

**What is the warming trend from 1937 to 2020?"

```
mod <- lm(July.C~Year,
    data=July,
    subset= Year >= 1937)

warming <- round(100*coef(mod)[2],2)

warming %>%
    paste("degrees C per century")
```

```
## [1] "2.28 degrees C per century"
```

8.4 1937-2020 Kotzebue July Air Temperatures

```
July %>%
  ggplot(aes(x=Year,
           y=July.C)) +
  geom_line() +
  xlim(1920,2020) +
  scale_x_continuous(breaks=seq(1920,2020,10)) +
  scale_y_continuous("Kotzebue July Temperature (C)",
                     sec.axis=sec_axis(~.-5.36*0.45,
                    "450 m asl July Temperature(C)")) +
  geom_smooth(formula=y~x,method="loess") +
  theme_gray(base_size = 14) +
  theme(axis.text.x =
                element_text(angle = 90,
                vjust = 0.5, hjust=1))+
  labs(title=bquote(Warming~trend==.(warming)*degree*C~century^-1))
```

```
## Scale for 'x' is already present. Adding another scale for 'x', which will
## replace the existing scale.
```

```
#ggsave("../../Figures/KotzebueTemps2020.pdf")
```

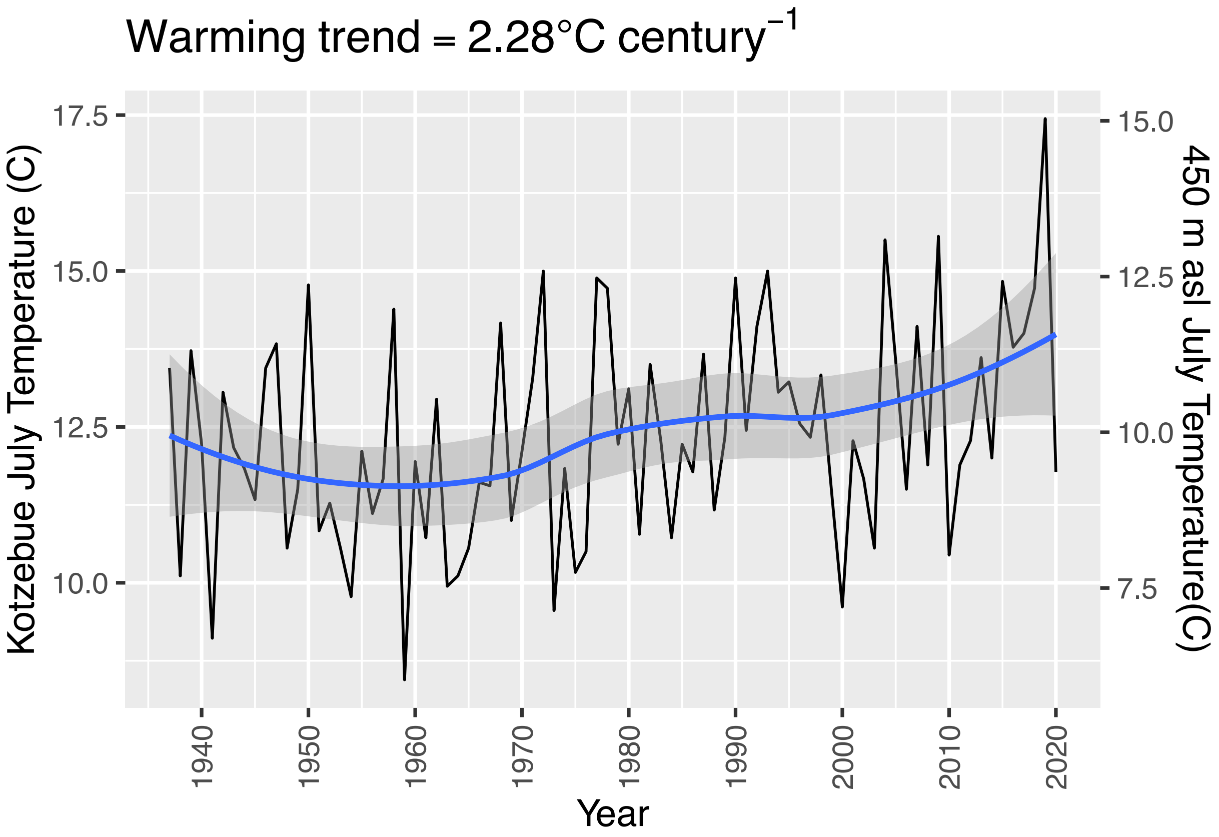

1937-2020 Kotzebue July air temperatures on the shore of the Chukchi Sea of the Arctic Ocean. Blue line is lowess fit through annual July mean daily values.

8.5 Chukchi Sea Ice

The Chukchi Sea is directly north of the Bering Strait, and represents that part of the Arctic Ocean west of the Beaufort Sea and east of the East Siberian Sea where sea ice is declining most rapidly[32].

Here we display the sea ice record for October downloaded from NSIDC (see Methods). We chose October because it is the month where temperatures generally fall below freezing for the duration of the winter in northwest Alaska. It is the month of below freezing air temperatures with minimum sea ice cover, thus the maximum open water available for evaporation and deposition as snow.

```
chukchi.precip <- read.csv("./ChukchiIceAreaFall.csv")
```

The 41 years of coverage are

```
paste(min(chukchi.precip$YEAR),"to",max(chukchi.precip$YEAR))
```

```
## [1] "1979 to 2019"
```

with no missing years,

```
identical(chukchi.precip$YEAR,
                    seq(1979,2019))
```

```
## [1] TRUE
```

and no missing months of October sea ice estimates:

```
sum(!is.na(chukchi.precip$OCT))
```

```
## [1] 41
```

Sea ice extent has been declining over this time period

```
chukchi.precip %>% ggplot(aes(x=YEAR,y=OCT)) +
    geom_point() +
    geom_line() +
    scale_y_continuous(lim=c(0,600000),
                                     breaks=seq(0,600000,100000),
                                     labels=seq(0,0.6,0.1))+
    labs(y=expression(Sea~ice~area~(10^6*km^2))) +
    labs(title="October Sea Ice Cover") +
    geom_smooth(formula=y~x,method="loess")
```

Because it is open water that leads to precipitation, we re-plotted this data set as millions of km^2 of open water Chukchi Sea open water during October by subtracting ice cover from the maximum ice extent in the record which occurred in April

```
chuk <- read.csv("./ChukchiArea.csv")
apply(chuk[,-1],2,
            function(x) round(max(x/10^6,na.rm=T),4)) %>%
    sort(decreasing=T)
```

```
##    APR    JAN    MAR    FEB    DEC    MAY    JUN    NOV    JUL    OCT    SEP
## 0.8232 0.8226 0.8219 0.8183 0.8180 0.8088 0.7622 0.7439 0.6025 0.5847 0.4696
##    AUG
## 0.4325
```

of

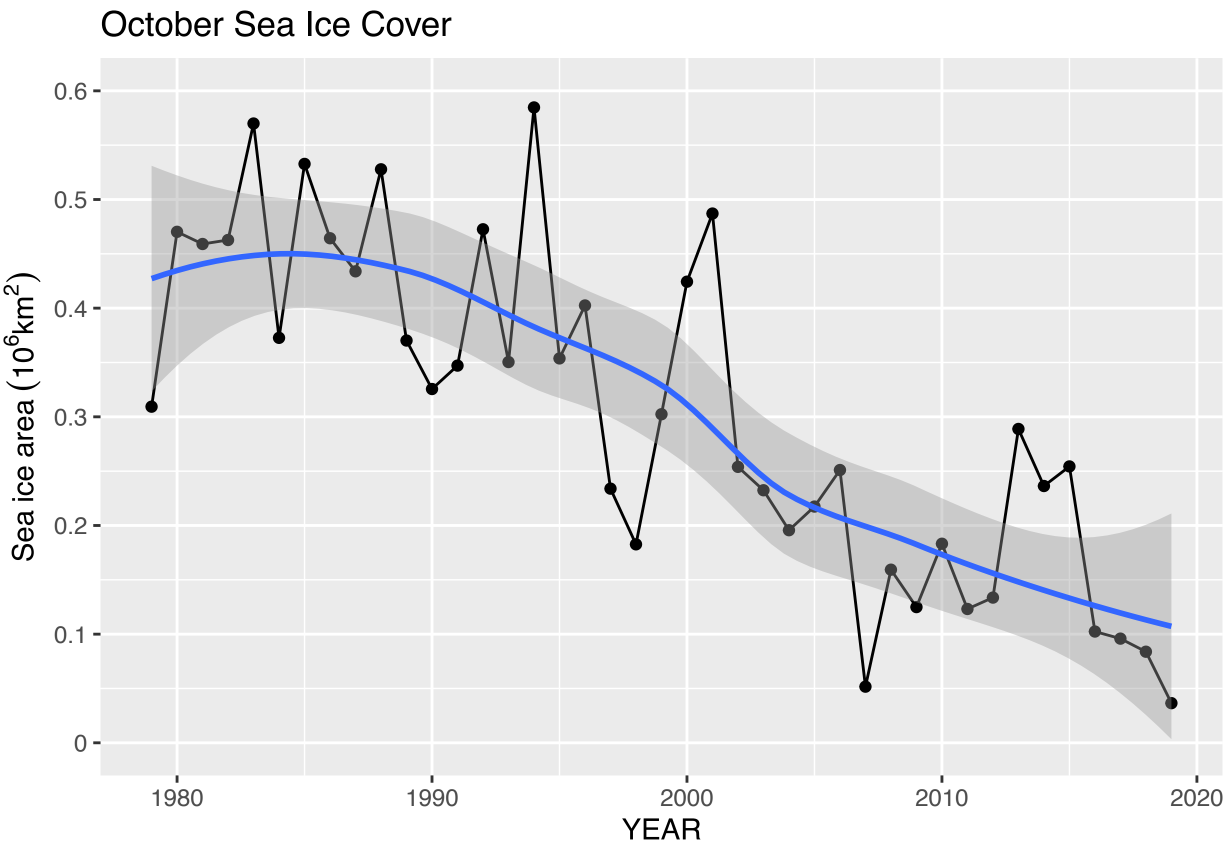

1979-2019 October sea ice cover in the Chukchi Sea of the Arctic Ocean. Blue line is lowess fit through annual area estimates.

```r
chuk.tot <- max(chuk,na.rm=T)/10^6

chuk$YEAR[which.max(chuk$APR)]
```

```
## [1] 2006
```

We also downloaded Kotzebue precipitation for winter months during the same hydrological years at the NOAA data portal and combined them with the Chukchi ice data converted to open water.

```r
chuk.kotz <-    chukchi.precip %>%
    mutate(Year=YEAR,
        H2O.SEP.milkm2=chuk.tot-SEP/10^6,
        H2O.OCT.milkm2=chuk.tot-OCT/10^6,
        H2O.NOV.milkm2=chuk.tot-NOV/10^6,
        H2O.DEC.milkm2=chuk.tot-DEC/10^6,
        Dec.cm=Fol.DEC.in*2.54,
        Jan.cm=Fol.JAN.in*2.54,
        Feb.cm=Fol.FEB.in*2.54,
        Mar.cm=Fol.MAR.in*2.54,
        TOT.cm=sqrt(Dec.cm+Jan.cm+Feb.cm+Mar.cm),
        Tot.cm=sqrt(Jan.cm+Feb.cm+Mar.cm),
        tot.cm=sqrt(Feb.cm+Mar.cm)) %>%
    na.omit()
```

The strongest correlations between precipitation and open water was found

```r
round(cor(chuk.kotz),3) ## max r = 0.438 for OCT open water vs Feb+Mar precip
```

```
##                   YEAR    SEP    OCT    NOV    DEC Fol.DEC.in Fol.JAN.in
## YEAR             1.000 -0.851 -0.799 -0.695 -0.579      0.167     -0.071
## SEP             -0.851  1.000  0.918  0.687  0.481     -0.172     -0.053
## OCT             -0.799  0.918  1.000  0.834  0.593     -0.187      0.026
## NOV             -0.695  0.687  0.834  1.000  0.761     -0.115      0.206
## DEC             -0.579  0.481  0.593  0.761  1.000      0.023      0.033
## Fol.DEC.in       0.167 -0.172 -0.187 -0.115  0.023      1.000     -0.258
## Fol.JAN.in      -0.071 -0.053  0.026  0.206  0.033     -0.258      1.000
## Fol.FEB.in       0.357 -0.390 -0.372 -0.200 -0.124      0.207      0.225
## Fol.MAR.in       0.192 -0.269 -0.343 -0.297 -0.273      0.104      0.027
## Year             1.000 -0.851 -0.799 -0.695 -0.579      0.167     -0.071
## H2O.SEP.milkm2   0.851 -1.000 -0.918 -0.687 -0.481      0.172      0.053
## H2O.OCT.milkm2   0.799 -0.918 -1.000 -0.834 -0.593      0.187     -0.026
## H2O.NOV.milkm2   0.695 -0.687 -0.834 -1.000 -0.761      0.115     -0.206
## H2O.DEC.milkm2   0.579 -0.481 -0.593 -0.761 -1.000     -0.023     -0.033
## Dec.cm           0.167 -0.172 -0.187 -0.115  0.023      1.000     -0.258
## Jan.cm          -0.071 -0.053  0.026  0.206  0.033     -0.258      1.000
## Feb.cm           0.357 -0.390 -0.372 -0.200 -0.124      0.207      0.225
## Mar.cm           0.192 -0.269 -0.343 -0.297 -0.273      0.104      0.027
## TOT.cm           0.308 -0.395 -0.406 -0.219 -0.159      0.459      0.349
## Tot.cm           0.275 -0.368 -0.377 -0.211 -0.208      0.115      0.507
## tot.cm           0.325 -0.402 -0.438 -0.307 -0.198      0.265      0.079
##                 Fol.FEB.in Fol.MAR.in   Year H2O.SEP.milkm2 H2O.OCT.milkm2
## YEAR                 0.357      0.192  1.000          0.851          0.799
## SEP                 -0.390     -0.269 -0.851         -1.000         -0.918
## OCT                 -0.372     -0.343 -0.799         -0.918         -1.000
## NOV                 -0.200     -0.297 -0.695         -0.687         -0.834
## DEC                 -0.124     -0.273 -0.579         -0.481         -0.593
```

```
## Fol.DEC.in          0.207      0.104  0.167          0.172          0.187
## Fol.JAN.in          0.225      0.027 -0.071          0.053         -0.026
## Fol.FEB.in          1.000      0.499  0.357          0.390          0.372
## Fol.MAR.in          0.499      1.000  0.192          0.269          0.343
## Year                0.357      0.192  1.000          0.851          0.799
## H2O.SEP.milkm2      0.390      0.269  0.851          1.000          0.918
## H2O.OCT.milkm2      0.372      0.343  0.799          0.918          1.000
## H2O.NOV.milkm2      0.200      0.297  0.695          0.687          0.834
## H2O.DEC.milkm2      0.124      0.273  0.579          0.481          0.593
## Dec.cm              0.207      0.104  0.167          0.172          0.187
## Jan.cm              0.225      0.027 -0.071          0.053         -0.026
## Feb.cm              1.000      0.499  0.357          0.390          0.372
## Mar.cm              0.499      1.000  0.192          0.269          0.343
## TOT.cm              0.852      0.673  0.308          0.395          0.406
## Tot.cm              0.850      0.709  0.275          0.368          0.377
## tot.cm              0.873      0.781  0.325          0.402          0.438
##                H2O.NOV.milkm2 H2O.DEC.milkm2 Dec.cm Jan.cm Feb.cm Mar.cm TOT.cm
## YEAR                    0.695          0.579  0.167 -0.071  0.357  0.192  0.308
## SEP                    -0.687         -0.481 -0.172 -0.053 -0.390 -0.269 -0.395
## OCT                    -0.834         -0.593 -0.187  0.026 -0.372 -0.343 -0.406
## NOV                    -1.000         -0.761 -0.115  0.206 -0.200 -0.297 -0.219
## DEC                    -0.761         -1.000  0.023  0.033 -0.124 -0.273 -0.159
## Fol.DEC.in              0.115         -0.023  1.000 -0.258  0.207  0.104  0.459
## Fol.JAN.in             -0.206         -0.033 -0.258  1.000  0.225  0.027  0.349
## Fol.FEB.in              0.200          0.124  0.207  0.225  1.000  0.499  0.852
## Fol.MAR.in              0.297          0.273  0.104  0.027  0.499  1.000  0.673
## Year                    0.695          0.579  0.167 -0.071  0.357  0.192  0.308
## H2O.SEP.milkm2          0.687          0.481  0.172  0.053  0.390  0.269  0.395
## H2O.OCT.milkm2          0.834          0.593  0.187 -0.026  0.372  0.343  0.406
## H2O.NOV.milkm2          1.000          0.761  0.115 -0.206  0.200  0.297  0.219
## H2O.DEC.milkm2          0.761          1.000 -0.023 -0.033  0.124  0.273  0.159
## Dec.cm                  0.115         -0.023  1.000 -0.258  0.207  0.104  0.459
## Jan.cm                 -0.206         -0.033 -0.258  1.000  0.225  0.027  0.349
## Feb.cm                  0.200          0.124  0.207  0.225  1.000  0.499  0.852
## Mar.cm                  0.297          0.273  0.104  0.027  0.499  1.000  0.673
## TOT.cm                  0.219          0.159  0.459  0.349  0.852  0.673  1.000
## Tot.cm                  0.211          0.208  0.115  0.507  0.850  0.709  0.933
## tot.cm                  0.307          0.198  0.265  0.079  0.873  0.781  0.895
##                Tot.cm tot.cm
## YEAR            0.275  0.325
## SEP            -0.368 -0.402
## OCT            -0.377 -0.438
## NOV            -0.211 -0.307
## DEC            -0.208 -0.198
## Fol.DEC.in      0.115  0.265
## Fol.JAN.in      0.507  0.079
## Fol.FEB.in      0.850  0.873
## Fol.MAR.in      0.709  0.781
## Year            0.275  0.325
## H2O.SEP.milkm2  0.368  0.402
## H2O.OCT.milkm2  0.377  0.438
## H2O.NOV.milkm2  0.211  0.307
## H2O.DEC.milkm2  0.208  0.198
## Dec.cm          0.115  0.265
```

```
## Jan.cm          0.507  0.079
## Feb.cm          0.850  0.873
## Mar.cm          0.709  0.781
## TOT.cm          0.933  0.895
## Tot.cm          1.000  0.889
## tot.cm          0.889  1.000
```

as between October ("H2O.OCT.milkm2") and the square root of Feb + March precip ("tot.cm") with a one-tailed test of the correlation between the two as

```
cor.test(~H2O.OCT.milkm2+tot.cm,
                 alternative="greater",
                 data=chuk.kotz)
```

```
##
##  Pearson's product-moment correlation
##
## data:  H2O.OCT.milkm2 and tot.cm
## t = 3.0039, df = 38, p-value = 0.002349
## alternative hypothesis: true correlation is greater than 0
## 95 percent confidence interval:
##  0.1968116 1.0000000
## sample estimates:
##       cor
## 0.4380585
```

The only other correlation > 0.40 was square root of Dec through March precipitation with October open water,with a one-tailed test of the correlation given as

```
cor.test(~H2O.OCT.milkm2+TOT.cm,
                 data=chuk.kotz)
```

```
##
##  Pearson's product-moment correlation
##
## data:  H2O.OCT.milkm2 and TOT.cm
## t = 2.7399, df = 38, p-value = 0.00931
## alternative hypothesis: true correlation is not equal to 0
## 95 percent confidence interval:
##  0.1083525 0.6370611
## sample estimates:
##       cor
## 0.4061531
```

and correlations of the same precipitation sums with the sum of October and September open water.

```
chuk.kotz %>% ggplot(aes(x=YEAR,y=TOT.cm)) +
    geom_point() +
    geom_line() +
    scale_y_continuous(breaks=c(2,3,4),
                labels=c(2,3,4)^2)+
    labs(y="Precipitation (cm)") +
    labs(title="Kotzebue December to March") +
    geom_smooth(formula=y~x,method="loess")
```

Taken together, increasing temperatures, October open water, and precipitation all show increasing trends over the same time period (ED Fig. 6):

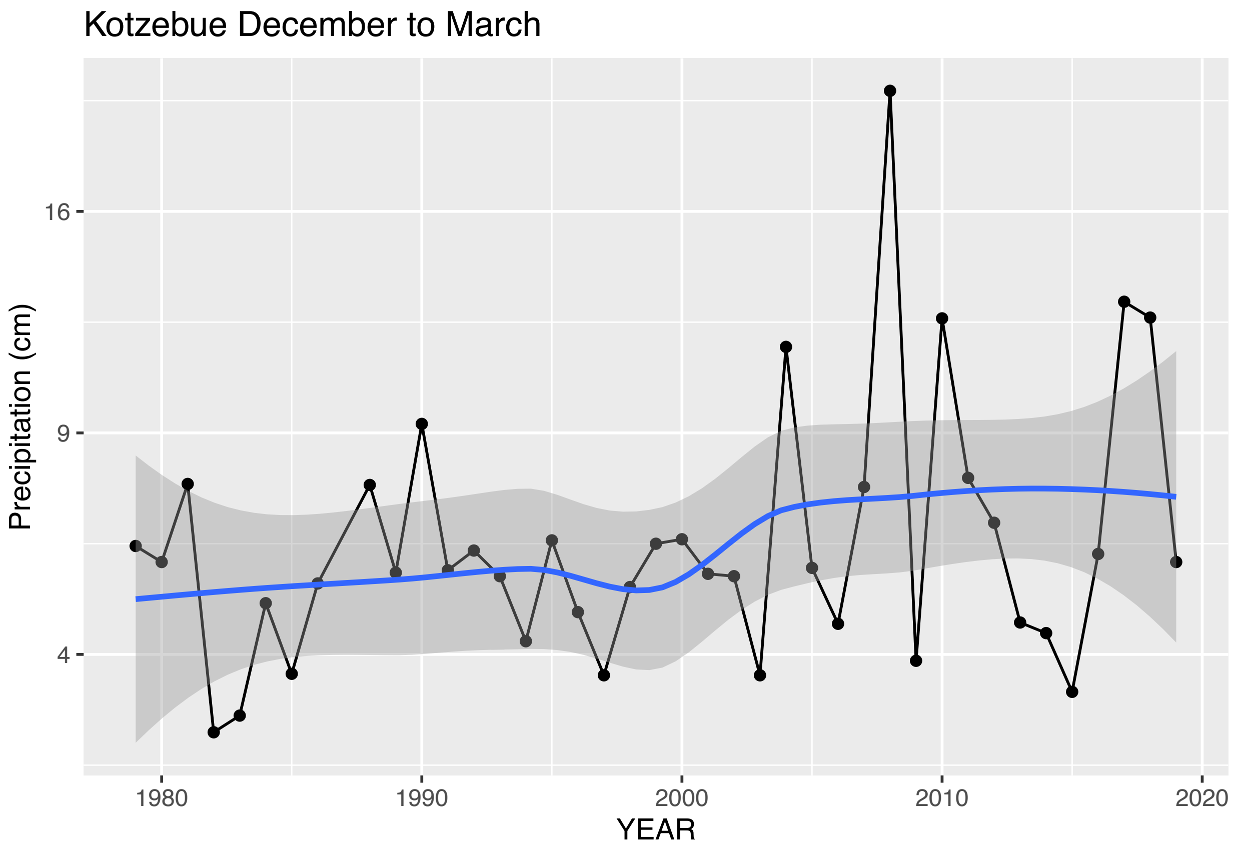

1979-2019 Kotzebue December-Mrch precipitation. Blue line is lowess fit through annual values.

```r
sea.ice <- chuk.kotz %>%
    mutate(EnvVar=H2O.OCT.milkm2,
                 type="Open_water_E.6km2",
                 Year=YEAR,
                 .keep="none")

winter.precip <- chuk.kotz %>%
    mutate(EnvVar=TOT.cm,
                 type="Dec_Mar_precip.cm",
                 Year=YEAR,
                 .keep="none")

July.T <- July %>%
    mutate(EnvVar=July.C,
                 type="temp_July_C",
                 Year=Year,
                 .keep="none")
```

```r
## Not Run
## ED Figure 5.a-c Time series of instrumental records from Kotzebue for
## mean July air temperature and total Dec-March precipitation;
## and satellite-sensed October open water in the Chukchi Sea
# index.names <- c(
#   `Dec_Mar_precip.cm` = 'Winter~precip~(cm)',
#   `Open_water_E.6km2` = 'Open~water~(km^2)',
#   `temp_July_C` = 'July~(degree*C)')
#
# scales_y <- list(
#   `Dec_Mar_precip.cm` = scale_y_continuous(limits = c(1.5, 4.5),
#       breaks=c(2,3,4),labels=c(2,3,4)^2),
#   `Open_water_E.6km2` = scale_y_continuous(limits = c(0.1, 0.8),
#       breaks = seq(0.1, 0.8, 0.2)),
#   `temp_July_C` = scale_y_continuous(limits = c(9, 18),
#       breaks = seq(0, 18, 3)))
#
# rbind(sea.ice,
#   winter.precip,
#       July.T) %>%
#   filter(Year <= 2019) %>%
#   ggplot(aes(x=Year,y=EnvVar,colour=type)) +
#   geom_point() +
#   geom_line() +
#   theme_void() +
#   facet_grid_sc(rows = vars(type),
#           scales = list(y = scales_y),
#           labeller = labeller(type =
#           as_labeller(index.names, label_parsed))) +
#   geom_smooth(formula=y~x,method="loess") +
#   labs(y=NULL) +
#   guides(colour="none") +
#   theme_bw() +
#       theme(axis.text.x=(element_text(angle = 90))) +
#   theme(panel.grid.major = element_blank(),
#               panel.grid.minor = element_blank(),
```

```
#                panel.background = element_blank(),
#                axis.line = element_line(colour = "black")) +
#   theme(text = element_text(size = 12)) +
#   theme(plot.title = element_text(size=12))
#
```

8.5.1 Correlations among Kotzebue July temperature, Chukchi open water, and Kotzebue winter precipitation

First make a dataframe holding all three:

```
climate.vars <- July %>%
    inner_join(chuk.kotz[,c("H2O.OCT.milkm2","TOT.cm","YEAR")],
                                   by=c("Year"="YEAR"))
```

The correlation between Kotzebue July temperature, Chukchi open water is one-tailed because we expect that warmer temperatures should lead to more open water:

```
cor.test(data=climate.vars,
                 alternative="greater",
                 ~July.C+H2O.OCT.milkm2)
```

```
##
##  Pearson's product-moment correlation
##
## data:  July.C and H2O.OCT.milkm2
## t = 2.4659, df = 38, p-value = 0.009147
## alternative hypothesis: true correlation is greater than 0
## 95 percent confidence interval:
##  0.1190756 1.0000000
## sample estimates:
##       cor
## 0.3714081
```

```
cor.test(data=climate.vars,
                 alternative="greater",
                 ~TOT.cm+H2O.OCT.milkm2)
```

```
##
##  Pearson's product-moment correlation
##
## data:  TOT.cm and H2O.OCT.milkm2
## t = 2.7399, df = 38, p-value = 0.004655
## alternative hypothesis: true correlation is greater than 0
## 95 percent confidence interval:
##  0.1592171 1.0000000
## sample estimates:
##       cor
## 0.4061531
```
