# Supplementary material for: Sufficient conditions for rapid range expansion of a boreal conifer
Source: Nature. 2022 Aug 10;608(7923):546–51. doi: 10.1038/s41586-022-05093-2 (PMC9385489; doi:10.1038/s41586-022-05093-2)
Supplement: Supplementary file 2 — Reporting Summary [file 41586_2022_5093_MOESM2_ESM.pdf]

nature portfolio

Corresponding author(s): Roman Dial

Last updated by author(s): 2022_06_09

Reporting Summary

Nature Portfolio wishes to improve the reproducibility of the work that we publish. This form provides structure for consistency and transparency in reporting. For further information on Nature Portfolio policies, see our Editorial Policies and the Editorial Policy Checklist.

Statistics

For all statistical analyses, confirm that the following items are present in the figure legend, table legend, main text, or Methods section.

n/a	Confirmed	
☐	☒	The exact sample size (*n*) for each experimental group/condition, given as a discrete number and unit of measurement
☐	☒	A statement on whether measurements were taken from distinct samples or whether the same sample was measured repeatedly
☐	☒	The statistical test(s) used AND whether they are one- or two-sided<br>*Only common tests should be described solely by name; describe more complex techniques in the Methods section.*
☐	☒	A description of all covariates tested
☐	☒	A description of any assumptions or corrections, such as tests of normality and adjustment for multiple comparisons
☐	☒	A full description of the statistical parameters including central tendency (e.g. means) or other basic estimates (e.g. regression coefficient) AND variation (e.g. standard deviation) or associated estimates of uncertainty (e.g. confidence intervals)
☐	☒	For null hypothesis testing, the test statistic (e.g. F, t, r) with confidence intervals, effect sizes, degrees of freedom and P value noted<br>*Give P values as exact values whenever suitable.*
☒	☐	For Bayesian analysis, information on the choice of priors and Markov chain Monte Carlo settings
☐	☒	For hierarchical and complex designs, identification of the appropriate level for tests and full reporting of outcomes
☐	☒	Estimates of effect sizes (e.g. Cohen's *d*, Pearson's *r*), indicating how they were calculated

Our web collection on statistics for biologists contains articles on many of the points above.

Software and code

Policy information about availability of computer code

Data collection

Data collected in the field and recorded on smart phone apps for recording data on iPhone 12 Pro and Pro Max: We used Gaia GPS (https://www.gaiagps.com/) for locations which uses the phone's GPS; the native camera app for taking photos; we measured tree heights with an LTI laser range finder (https://lasertech.com/trupulse-series/) and with the Arboreal app (https://www.arboreal.se/en/) which uses the structure from motion from the phone camera and the iPhone 12 Pro's lidar; we entered data into the native iPhone app Numbers and a third party app Form Connect Pro (https://www.formconnections.com/formconnect-pro-form-designer/). The laboratory data we collected and the on-line sources of the climate, digital elevation model, sea ice, and imagery data are described in the methods. For example we used CooRecorder (https://www.cybis.se/forfun/dendro/index.htm) to scan increment cores and we imported satellite images into Google Earth Pro and digitized the shadows of trees there.

Data analysis

We used the open source statistical software R in its latest version, R 4.1.2 "Bird Hippie" released on 2021/11/01. Our Supplementary Information provides the key code annotated and run as a pdf Markdown from RStudio. We tried to provide a readable walkthrough of the analysis for reviewers and interested readers, when/if the paper is published. All the R packages are listed below.

Other software is described in the methods in the appropriate locations: like Google Earth Pro, and two dendro-packages CooRecorder (https://www.cybis.se/forfun/dendro/index.htm) and CDendro (https://www.cybis.se/forfun/dendro/index.htm) software.

R packages (latest versions as of submission: January 18 2022) used:
bookdown
broom
Cairo
cowplot
data.table
doBy
dplR

emmeans
fields
ggridges
github("zeehio/facetscales")
Hmisc
lme4
MASS
nlme
plyr
raster
rcartocolor
reshape2
rgdal
rgeos
scales
sf
sp
stars
stringr
terra
tidyverse
viridis

For manuscripts utilizing custom algorithms or software that are central to the research but not yet described in published literature, software must be made available to editors and reviewers. We strongly encourage code deposition in a community repository (e.g. GitHub). See the Nature Portfolio guidelines for submitting code & software for further information.

Data

Policy information about availability of data

All manuscripts must include a data availability statement. This statement should provide the following information, where applicable:

- Accession codes, unique identifiers, or web links for publicly available datasets
- A description of any restrictions on data availability
- For clinical datasets or third party data, please ensure that the statement adheres to our policy

Data and code used for analysis presented here are archived at the National Science Foundation's Arctic Data Center as doi:10.18739/A2X63B650.

Field-specific reporting

Please select the one below that is the best fit for your research. If you are not sure, read the appropriate sections before making your selection.

☐ Life sciences ☐ Behavioural & social sciences ☒ Ecological, evolutionary & environmental sciences

For a reference copy of the document with all sections, see nature.com/documents/nr-reporting-summary-flat.pdf

Ecological, evolutionary & environmental sciences study design

All studies must disclose on these points even when the disclosure is negative.

Study description

This study compares attributes of a recently discovered disjunct population of a boreal conifer that colonized in the 20th century to attributes of established treelines, thereby describing for the first time sufficient conditions leading to boreal forest advance into Arctic tundra. These conditions are linked to a warming climate, increasingly open Arctic Ocean waters, and increased winter precipitation.

Essentially all of the data presented is quantitative and covers five topics that tell the story:
1) The distance and rate of range expansion comparing adult to juvenile colonists that suggests acceleration of the range expansion.
2) The population growth of colonists from 1900-1980 equals a ten-year doubling time.
3) A comparison of individual growth metrics in colonists to individuals at established treelines (IET) shows that colonist growth is nearly double that of individuals in established populations.
4) A comparison of the environmental conditions between colonists and established treelines suggests that colonists occupy snowier, more nutrient-rich environments.
5) Regional climate variables over the historical period of instrumental recording show a century of average warming at over 2 degreeC/century since 1937 and near doubling of open water extent in the October Chukchi Sea and in winter precipitation over the last four decades

For 1) the dependent numeric variables include distances northward from established treelines (defined by 3,366 vertices of digitized lines) and elevations above (or below in the case of colonists beyond the mountain barrier) established treelines. Using the maximum age of trees sampled with increment cores another dependent variable is migration rate as distance divided by maximum age. The independent factor variable is age-class, with adults as trees over 2.5 m (n = 5,986), and juveniles (n = 770) as under 2.5 m. These data are presented within the framework of invasion theory and dispersal kernels and the context of documented recent spruce migration rates vs paleo-migration rates.

For 2) we measured 5,986 tree shadow lengths on satellite imagery and collected tree heights (n = 340 trees) and increment cores (n = 140 trees) from hundreds of trees in the field. We constructed Gaussian kernel density distributions by tree height class of n = 123 aged trees in five size classes: 3-4m (n = 31 aged trees with heights), 4-4.5 (n = 25), 4.5-5 (n = 21), 5-5.5 (n = 29), and 5.5-7m (n = 17)). We used linear mixed models with height as dependent variable, shadow length as independent variable and sample area as a random factor (random slopes and random intercepts) to construct a model to predict tree height based on shadow length for 1,971 (all shadows in two subwatersheds) of the 5,986 shadows repeatedly in a Monte Carlo method with 5,000 runs. For each run, we drew parameters from a multivariate normal distribution to estimate 1,972 tree heights based on shadow length. For each of the 1,971 trees we then drew an age from the appropriate kernel density of ages given the tree's height class. We aggregated by year and cumulatively summed to get population size by year from 1900 to 1980. For each of these 5,000 runs we fit an exponential model using a non-linear least squares algorithm. Averaging the exponential growth rate gave our best estimate of population growth of the 1,971 trees that cast the shadows in the two subwatersheds.

For 3) we used three growth metrics as dependent variables: (i) Current annual growth (CAG) in lateral branches of adults (n = 17 colonists from m = 6 watersheds and n = 457 IET from m = 12 watersheds) at 1.4 m above ground. (ii) Relative growth rate (RGR) in height during the last five years (2015-2020) in juveniles (n = 300 colonists from m = 4 field sites and n = 271 IET from m = 20 field sites)). And (iii) Radial stem growth in juvenile (n = 15 trees < 30 years old) and adult (n = 125 trees ≥ 30 years old) colonists as measured using increment cores and two standard ring indices, basal area index (BAI) and autoregressive residuals (AR). Each individual tree ring chronology was correlated with Pearson's correlation with July temperature from Kotzebue; no -values were calculated. For comparisons between colonist and established treeline populations, measured adults and juveniles were replicated within random factor "areas" as watersheds (CAG) or field sites (RGR). These areas served as random factors in linear mixed effects models. The independent fixed factor variable for (i) and (ii) was source population of the individual as "colonist" or "established treeline" populations. For RGR an included covariate was juvenile height, because RGR decreases with height. The linear mixed effects model included random intercepts; the interaction of the covariate juvenile height with source population provided the fixed effects. For CAG, there was no covariate, only the fixed factor of source population and the random intercept of watershed.

For 4) we compared two types of environmental variables: nutrient and climatic. We used three dependent nutrient variables: percent foliar phosphorous (n = 19 colonists at m = 6 watersheds; n = 51 pooled colonist needle samples from m = 14 watersheds), percent foliar nitrogen (n = 20 colonists at m = 6 watersheds; n = 51 pooled colonist needle samples from m = 14 watersheds), and the ratio of delta N15 to N. Each nutrient variable was treated as described above in 3) (i) for CAG. That is, we employed a watershed as a random factor for intercept and population ("colonist" or "established treeline") as fixed factor. We employed two gridded climate variables each with a biological basis: mean July temperature and winter precipitation. We performed no inferential statistics with these variables. Instead we graphically compare the bivariate kernel density distributions of the two variables for (i) the watersheds supporting the colonist populations, (ii) the nearby watersheds supporting established treelines, (iii) the gridded climate pixels enclosing the locations of the colonists (n = 708 pixels; if multiple individuals were found in one pixel the pixel is reported only once) and (iv)) the gridded climate pixels enclosing the locations of established treeline vertices (n = 1,213 pixels; if multiple vertices were contained in one pixel the pixel is reported only once).

For 5) we report the instrumental records of July temperature and winter precipitation as available from the US Government's NOAA web site and use the sea ice extent in October to estimate the amount of open water in October over the period of reporting on the National Snow and Ice Data Center. Here we perform simple one-tailed Pearson correlation tests.

Research sample

We sampled white spruce, Picea glauca, growing at and beyond treeline at the northwest extreme of their geographic range in Arctic Alaska, USA. The sample is meant to represent the population of white spruce that has recently expanded beyond the known range of the species.

Sampling strategy

We collected as many colonist samples as we could given the time and weather constraints of remote fly-in/walk and float out self-contained expeditionary field science. At the established treelines our samples were pre-determined by experimental design of 10 trees per study plot with one to three study plots per study site and two to six study sites per remote wilderness access site by fixed wing, single engine airplane. In addition, we searched for and recorded locations of colonists found beyond treeline in 37 other watersheds.

Data collection

RJD led four field campaigns to document, map, measure, and collect foliar and wood samples of white spruce colonists to the area of interest (AOI) and seven field campaigns outside the AOI to search for spruce colonists. He was accompanied by one to six field assistants. Field assistants Logan Berner and Patrick Burns of Northern Arizona University and Ray Koleser, formerly of the United States Forest Service Forest Inventory and Analysis program cored the trees. RJD, Julia Ditto, Ray Koleser, Brad Meiklejohn, Patrick Burns, Ben Weissenbach, Russell Wong, and Madeline Zietlow participated in the following: measuring heights above ground of bud scars of juvenile colonists with a tape measure and adults with a laser range finder and/or phone app; measuring DBH; mapping colonists with a GPS; photographing individual trees; recording ecologically relevant information such as cone abundance, growth form, damage, abundance of shrubs and juveniles within 5m of measured adults. Annie Brownlee and Jocyln Kramer assisted in laboratory processing and dendrochronology supervised by CTM and PFS. CTM measured rings and cross-dated increment cores. CTM, PFS, Julia Ditto, Ben Weissenbach, Madeline Zietlow, Russell Wong, measured bud scar heights of juveniles at established treelines. RJD and Sylvia Taylor digitized shadows on satellite imagery. RJD, CTM, PFS, Russell Wong and Scout Donahue collected foliar samples. REH and PFS supervised foliar nutrient data collection. RJD downloaded on-line data including Digital Globe World-View imagery, PRISM gridded climatology, IFSAR Digital Elevation models, Kotzebue and RAWS climate data, and Chukchi Sea Ice area.

Timing and spatial scale

We collected data from the colonist population within the AOI in August and September of 2019; September 2020, and July 2021. We searched for and found spruce colonists outside the AOU in June and August 2018, June 2019, July and August 2020, and June-August 2021. We collected data from established treelines in August-September 2019, and June-September 2021. The spatial extent of the data collection covers 1000 km east-west and 200 km north-south approximately. Most of the colonist AOI data were collected in an area 90 km east-west and 55 km north-south.

Data exclusions

We exclude data only from the population simulation. In that case we excluded trees less than 3 m tall because they were unlikely visible in the remote sensing imagery, buried by snow and casting short shadows. We also exclude two locations of spruce beyond treeline. These were the only two krummholz tree islands that we encountered distant from treeline and we did not consider them

to be colonists. We also discarded 10 of 150 tree cores because they lacked height above ground measurements where increment cores were taken.

Reproducibility

We collected data during 12 field campaigns, where a field campaign is defined by a discrete field expedition to the Brooks Range led by one of the co-authors. Because the study areas are distant and expensive to access, our goal was to visit as broad an area as possible. We did not repeat any measurements in the AOI but we used the same sampling methods we used on previous trips plus generally new ones as well as we learned more about the area and new questions arose. This study was more akin to scientific field exploration than a replicated experiment. However we have ongoing experiments at established treelines that are included in this study for comparison, but we include current annual growth measurements (CAG) from only 2019 (since we only measured CAG from the AOI that year) and relative growth rates (RGR) are from 2021 at our experimental study sites because we only measured RGR in the AOI in 2021.

Randomization

Field sites among the established treelines were selected randomly conditional on being at treeline and within 4 km of the airstrip access. For the colonist populations or transects were not random, but chosen based on remote sensing imagery and the ability to travel on the terrain. Four field campaigns (2019-2021) focused on three objectives in watersheds that were within or adjacent to the Noatak River basin but without established treelines visible on WV growing-season scenes: (1) locate and document colonists at the geographic range boundary of white spruce, (2) verify locations of a sample of trees suggested by imagery in the AOI, and (3) collect ecological measurements germane to white spruce range expansion. We selected representative (i.e. stratified random sampling by height class) and extreme individual adults (i.e., we sought out the highest, tallest, farthest north, and biggest and oldest looking individuals). An additional five field campaigns (June and August 2018, June 2019, July-August 2020, and June-August 2021) were undertaken to locate and record whiet spruce beyond established treelines during very long sample transects that were not random, but instead selected to explore treeline conditional on ability to travel and be resupplied by small fixed-wing, single-engine aircraft.

Blinding

For field data collection blinding is impractical.

Did the study involve field work? ☒ Yes ☐ No

Field work, collection and transport

Field conditions

The Brooks Range extends from the Canadian Border to the Chukchi Sea, roughly 1000 km east-west. In the east it is dry and very cold in winter. In the center it receives more precipitation. In the west it is warmest and wettest. Vegetaion along treeline is mostly dominated by white spruce with an understory of willow, birch, and occasionally alder. The ground cover included ericaceous and other dwarf shrubs, especially Dryas, growing among gramminoids, mosses, and lichens.

Only one road crosses the Range roughly one-third of the distance from Canada to Kotzebue. Within the Range itself there are only three communities, two on the road. Nearly all of the Brooks Range is protected in Federally designated conservation areas that protect the Range from extractive activities including mining and logging. At treeline forest fires are uncommon although thawing permafrost is leading to increased disturbance by frozen debris lobes.

Northwest Alaska’s Noatak and Kobuk Rivers are located above the Arctic Circle and drain into the Chukchi Sea of the Arctic Ocean near Kotzebue (162.604°W, 66.891°N). The Baird Mountains of the southwestern Brooks Range separate the Kobuk from the Noatak basins and the De Long Mountains of the northwestern Brooks Range separate the Noatak from North Slope river basins and from the Wulik basin. The lower basins of the Noatak and Kobuk Rivers are included in Alaska’s West Coast climate division, characterized by greater precipitation, warmer winters, and cooler summers than the Central Interior climate, with greater precipitation and warmer temperatures than the North Slope climate. The upper basin of the 700 km Noatak lies at the intersection of all three climate divisions, which warmed 1949-2012. December-January precipitation increased 1949-2012 in the West Coast climate division, as did North Slope winter precipitation 1980-2012.

Vegetation includes dwarf, low, or tall shrub tundras that cover about 60% of the 33,000 km2 Noatak River basin (entirely protected within federal conservation units), with tussock sedge tundra covering another 30% and wetlands and barrens most of the remainder The main valley and tributaries along the lowest 200 km of the Noatak River support stands of white spruce, typically associated with greater depth or absence of permafrost. The treelines bounding these forests have long been identified as the northwest range extent of white spruce.

The upper Noatak basin, a 500 km reach, is underlain by extensive continuous permafrost. It has been considered empty of spruce since USGS geologist Philip Smith explored the Kobuk, Alatna, and Noatak Rivers by canoe in 1911. The adjacent Kobuk and Alatna River basins support boreal forests of black and white spruce, paper birch, and aspen along much of their lengths. By surveying transects along and beyond hydrological divides separating the Noatak, Wulik, Kobuk, and Alatna River basins, informed by very-high resolution satellite scenes, we documented the locations of over 7,000 individual spruce colonists.

Location

The location of the AOI includes four watersheds of the Cutler River a tributary of the upper Noatak River in northwest Alaska's Brooks Range and four adjacent watersheds in the upper Kobuk River basin. The center of the eight watersheds is roughly located at 157.7 W 67.5 N. The watersheds include the Kaluich, Cutler, Amakomanak, and Imelyak Rivers in the Noatak Basin and the Akillik, Redstone, and Redstone, Miluet, Akillik, and Hunt watersheds (HUC 10) of the Middle Kobuk subbasin as defined by the US Governments USGS "Hydrologic Unit Classification" system. In addition, we cite data from 37 other watersheds that are spread across the Range. Here are there locations: Philip Smith North Fork East Fork Chandalar River 147.424851 -147.424851
Philip Smith Sheenjek River 143.612821 -143.612821
Endicott Clear River 150.69185 -150.69185
Endicott North Fork Koyukuk River 150.69901 -150.69901
Endicott Iniakuk River 153.04305 -153.04305
Endicott Wolverine Creek 152.90086 -152.90086
Endicott Kutuk River 153.706 -153.706
Endicott Lucky Six 154.859 -154.859
Schwatka Ipnelivik 156.294 -156.294

Schwatka Reed 155.061 -155.061
Baird Timber Creek 160.420162 -160.420162
Philip Smith Wind River 147.148 -147.148
Endicott Publituk Creek 151.90186 -151.90186
Endicott Wild River 151.67801 -151.67801
Endicott Agiak Creek 152.921 -152.921
Baird Omar River 160.518988 -160.518988
Baird Squirrel 161.456217 -161.456217
De Long Wulik 163.22 -163.22
Philip Smith Middle Fork Chandalar River 147.523 -147.523
Endicott Tinayguk River 151.48682 -151.48682
Endicott Pingaluk River 153.46783 -153.46783
Schwatka Kugrak 155.723 -155.723
Baird North Fork Squirrel 161.241982 -161.241982
Endicott Glacier River 150.40739 -150.40739
Endicott Nahtuk River 153.17322 -153.17322
Endicott Akabluak east 154.461 -154.461
Endicott Awlinyak 154.342 -154.342
De Long Upper Wrench 162.624 -162.624
Endicott Kevuk Creek 152.68016 -152.68016
Schwatka Imelyak 157.004 -157.004
Endicott Wolf Creek 151.29355 -151.29355
Endicott Akabluak west 154.777 -154.777
Endicott Arrigetch Valley 154.185 -154.185
De Long Wrench Ck south 162.544 -162.544
Philip Smith W. Sheenjek River 147.424851 -147.424851
De Long Wrench Ck woods 162.596537 -162.596537
Philip Smith North Fork Chandalar River 149.406395 -149.406395
Chukchi Sea Cape Krusenstern 67.040703 -163.11433
Noatak Noatak. 158.34332 -158.34332
Noatak Noatak.158.22879 -158.22879

Access & import/export

We applied for and received the following United States National Park Service Permits to conduct research in the Gates of the Arctic National Park and Noatak National Preserve conservation units: # NOAT-2021-SCI-0002, # GAAR-2021-SCI-0004, # GAAR-2019-SCI-0002. In order to reduce our environmental impact we used aircraft only to fly-in to the colonist population. from there we walked and rafted out to small villages.

Disturbance

We camped following "leave no trace principals" and made every effort not to disturb the wilderness environment or its wild inhabitants.

Reporting for specific materials, systems and methods

We require information from authors about some types of materials, experimental systems and methods used in many studies. Here, indicate whether each material, system or method listed is relevant to your study. If you are not sure if a list item applies to your research, read the appropriate section before selecting a response.

Materials & experimental systems

n/a	Involved in the study
☒	☐ Antibodies
☒	☐ Eukaryotic cell lines
☒	☐ Palaeontology and archaeology
☒	☐ Animals and other organisms
☒	☐ Human research participants
☒	☐ Clinical data
☒	☐ Dual use research of concern

Methods

n/a	Involved in the study
☒	☐ ChIP-seq
☒	☐ Flow cytometry
☒	☐ MRI-based neuroimaging
