# Supplementary material for: Sufficient conditions for rapid range expansion of a boreal conifer
Source: Nature. 2022 Aug 10;608(7923):546–51. doi: 10.1038/s41586-022-05093-2 (PMC9385489; doi:10.1038/s41586-022-05093-2)
Supplement: Supplementary file 3 — This file contains Supplementary Figures 1–14. [file 41586_2022_5093_MOESM3_ESM.pdf]

# Supplementary Information Figures for

## Sufficient conditions for rapid range expansion of a boreal conifer

R. J. Dial, C. T. Maher, R. E. Hewitt, and P. F. Sullivan

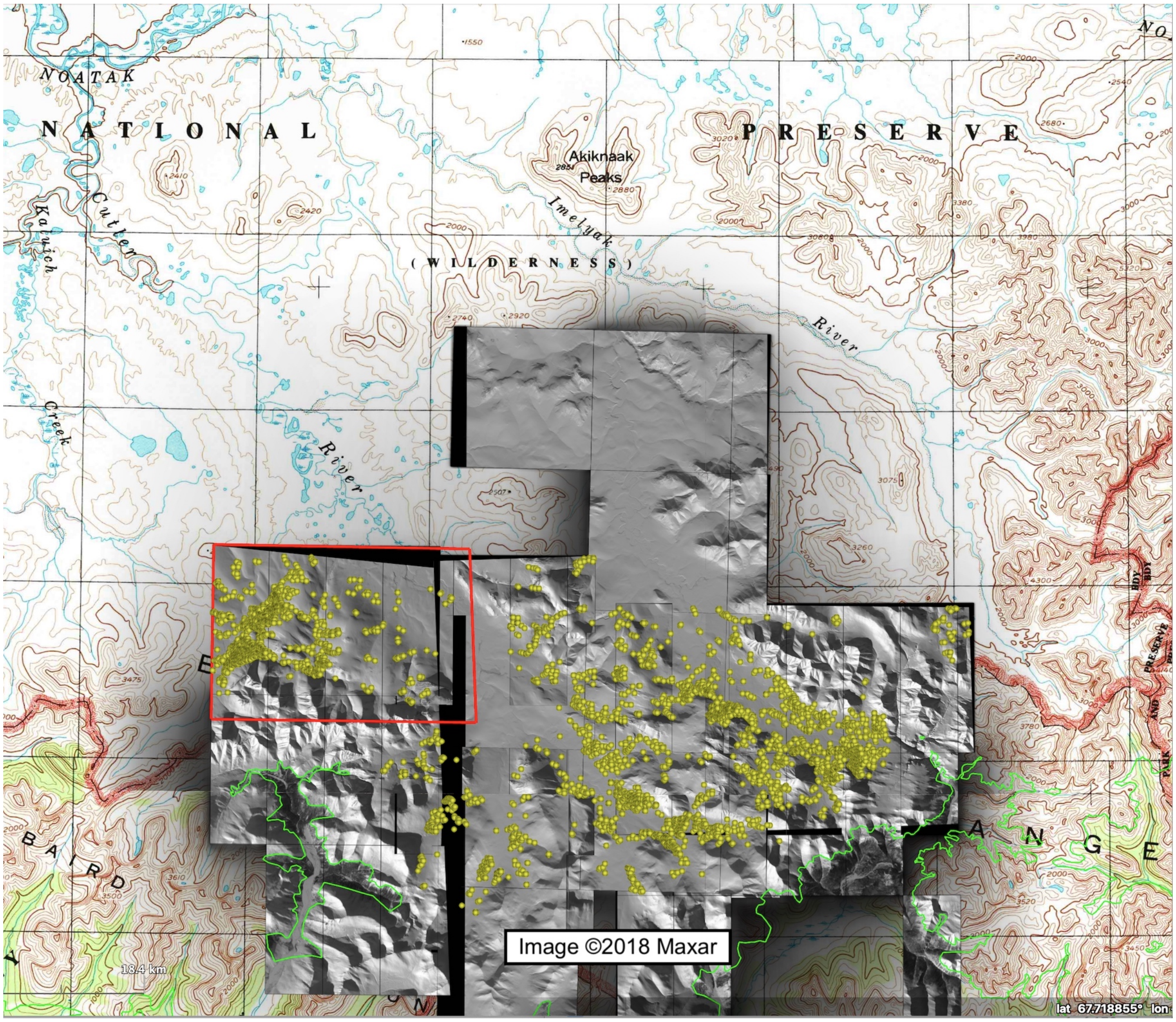


**SI Figure 1.** Snow-covered scenes of World View (WV) imagery captured in late March and early April 2018 overlayed on USGS 1:250,000 scale Ambler River topographic quadrangle. Yellow dots locate base of spruce shadows digitized wall-to-wall over available imagery of WV winter scenes within Noatak basin watersheds shown here as super-overlays on Google Earth Pro. Each yellow dot is located at the base of a shadow digitized in GEP on a super-overlay. Green lines in Kobuk basin watersheds indicate established treelines, defined as those white spruce treelines visible on summer scenes of WV imagery. The red rectangle encloses an area of 136 km$^2$ surrounding the population simulated in SI 4. Uniform grid of squares on topographic map are 10 km on a side. Contour interval is 250 feet. North is toward top of page.

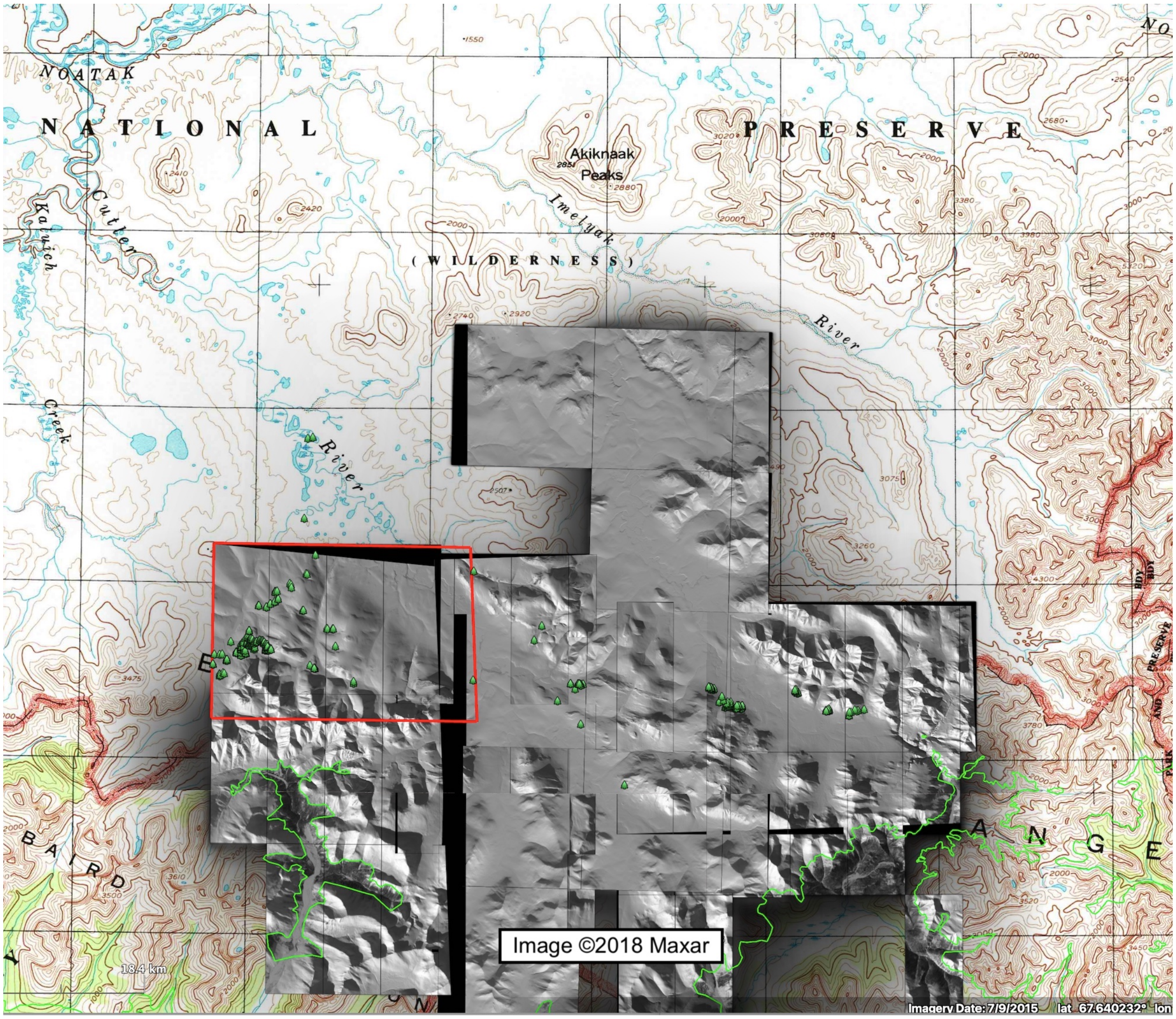


**SI Figure 2.** Same thematic features, WV imagery, and base USGS map as SI Figure 1. Green tree icons indicate GNSS locations where white spruce adults (height >2.5 m) were measured in the field. Note three icons representing three adults outside WV imagery extent near the word "River" along Cutler River less than 10 km north of red rectangle.

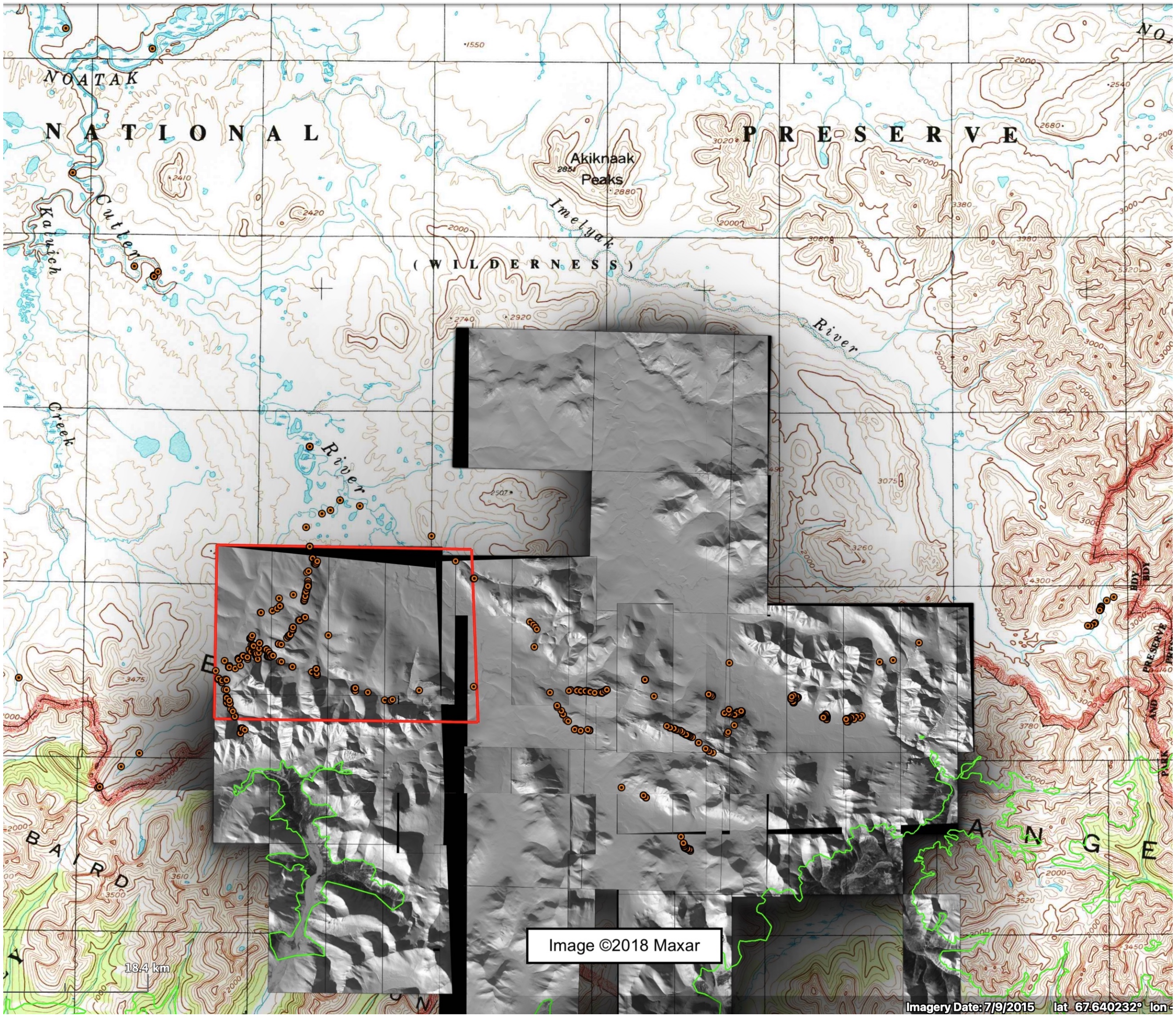


**SI Figure 3.** Same thematic features, WV imagery, and base USGS map as SI Figures 1 and 2.Orange bullets indicate GNSS locations where white spruce juveniles (height <2.5 m) were measured in the field. Note six orange bullets representing fourteen juveniles beyond known adults shown in SI Figures 1 and 2. These juveniles are located downstream of the Cutler River's northernmost tributaries, Imelyak River and Kaluich Creek, and on the north side of the Noatak River.

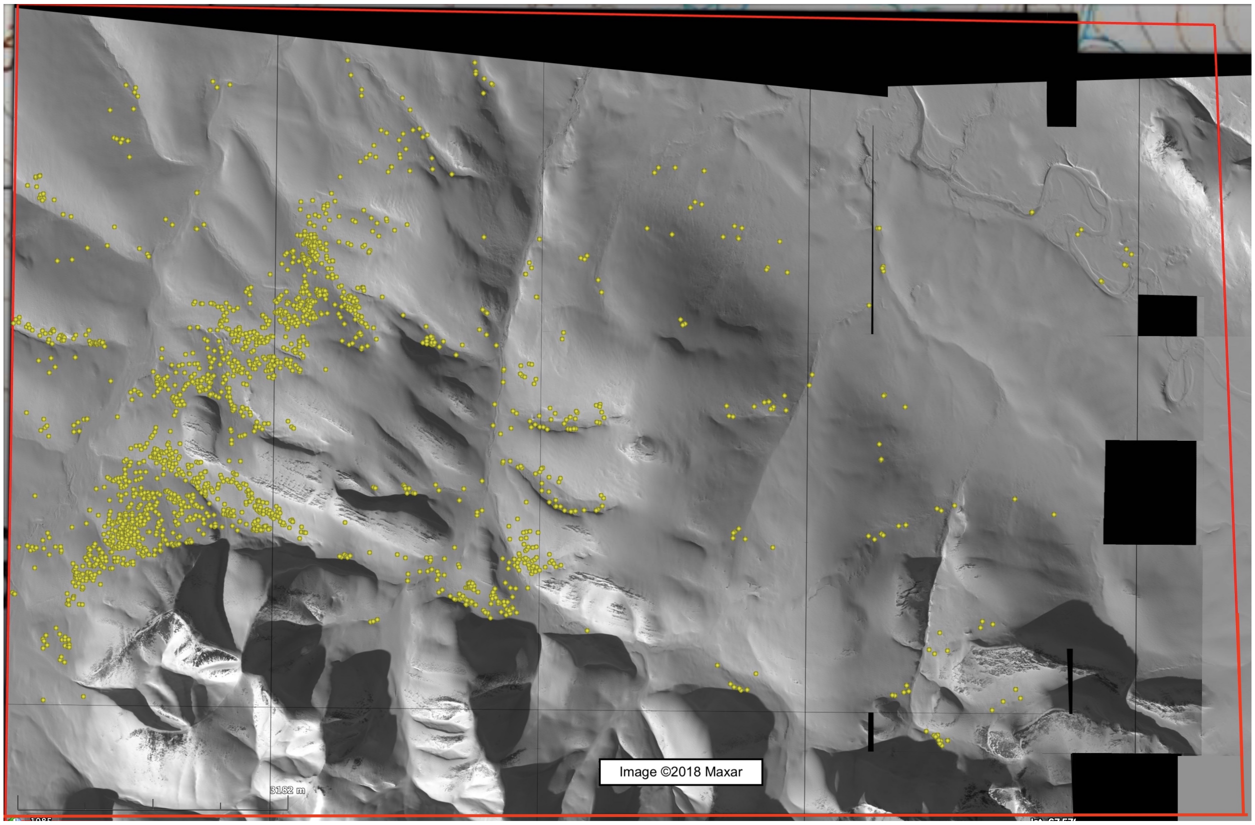


**SI Figure 4.** Area of population reconstruction from 1900 to 1980 using relationships betwen cross-dated ages, field-measured heights, and shadow lengths of 1,971 spruce digitized on WV imagery. Yellow bullets are locations of digitized shadow lengths. Red border encloses reconstructed population and is shown in SI Figs. 1-3 as red rectangle in each and in ED Fig. 1a as central black rectangle labeled "Simulated population area." Black areas to the east are artifacts of using multiple and overlapping SuperOverlays with Google Earth Pro (GEP) for this figure and did not obscure areas when used seprately duriing digitizing. Black areas to the north are the edge of available imagery on 26 March 2018. North is toward the top of the page. The dimensions of the 136 km$^2$ area are 14 km $\times$ 9.7 km.

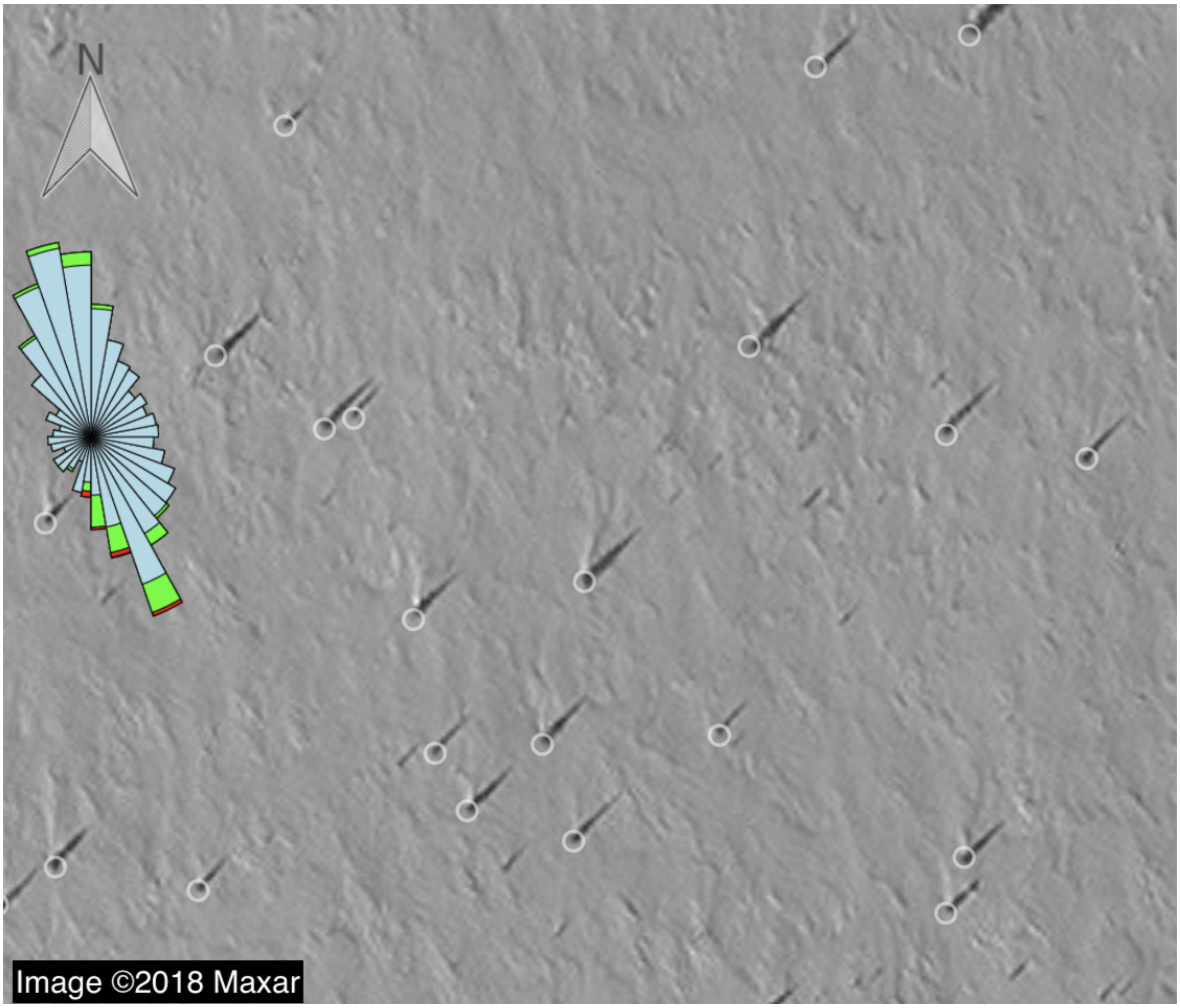


**SI Figure 5.** Image from WV-1 scene displayed using R package raster and similar to main text Figure 1b. Note that some short shadows are not marked with small white circles that indicate shadows digitized by technician.

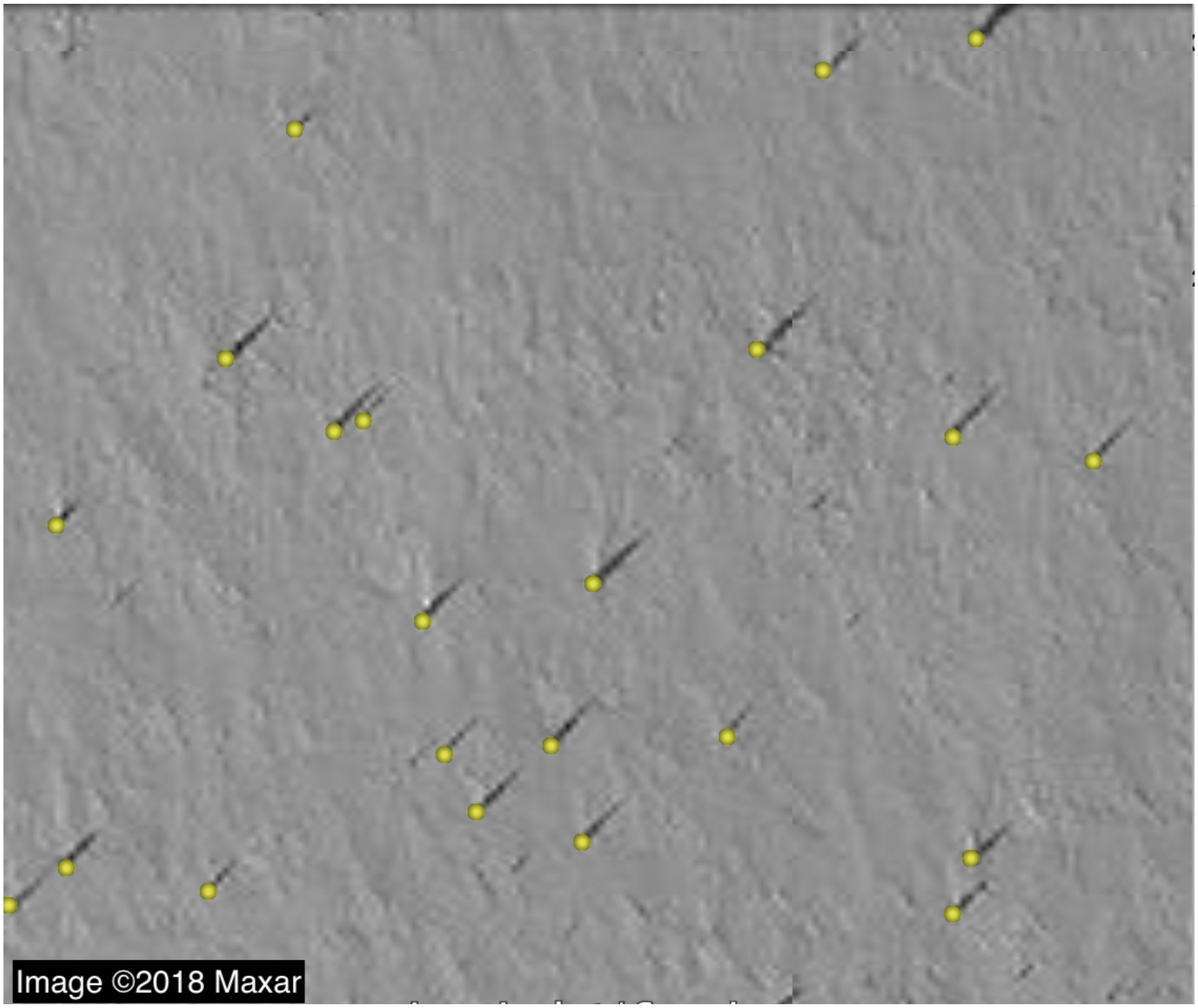


**SI Figure 6.** Image of super-overlay of WV-1 scene as displayed using GEP. Note ambiguity in short shadows unmarked with small yellow bullets that indicate shadows digitized by technician.

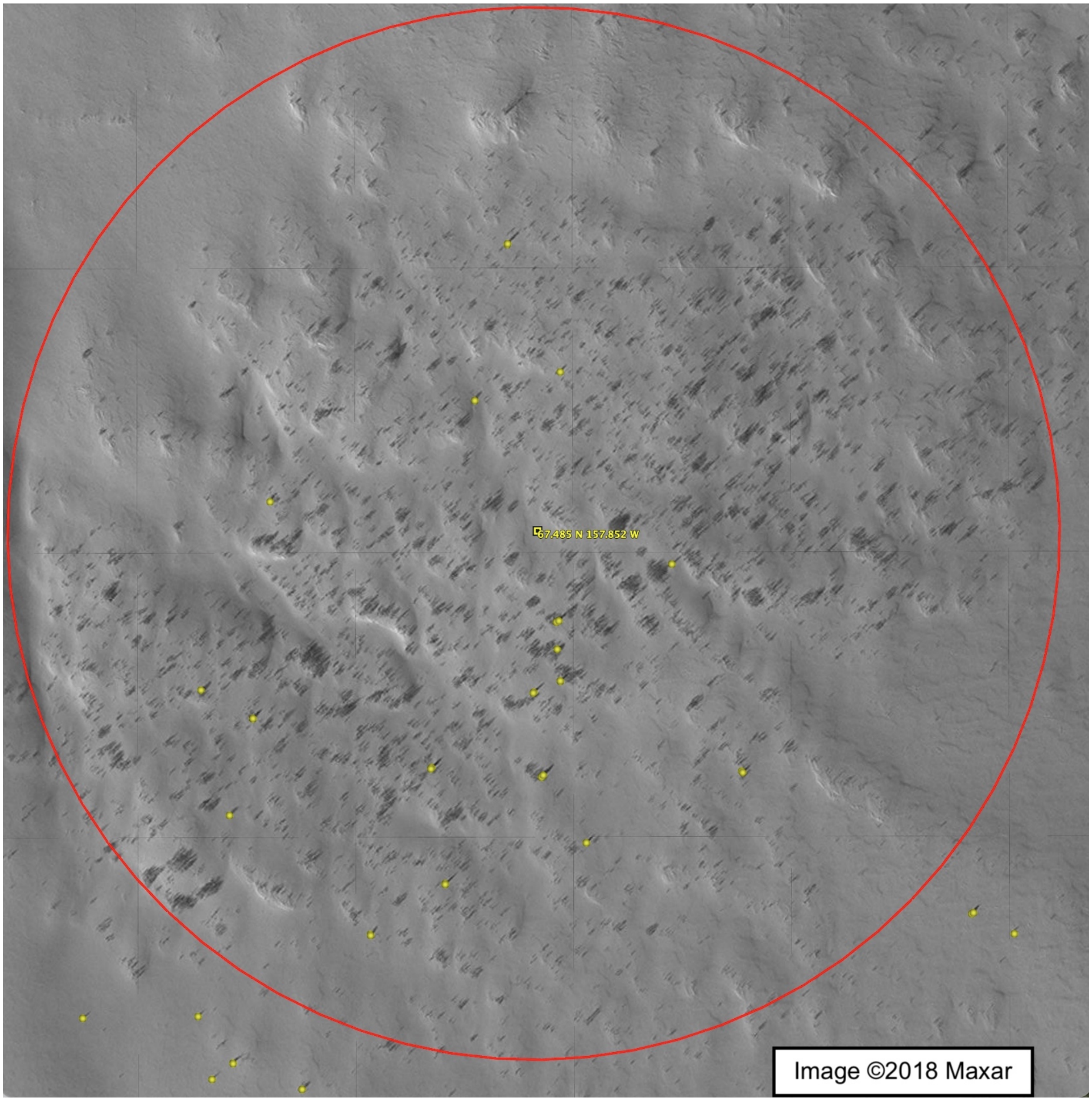


**SI Figure 7.** A challenging satellite scene for shadow classification. The red circle encloses a 25 ha area with both willow and spruce growth on a hillside, shown here to compare spruce with very tall shrubs. We did not ground-truth this location, but from the imagery it appears to have the most shrubs protruding from snow in March-April 2018 of all satellite scenes interpreted within the AOI as shown in SI Fig. 1. Yellow bullets indicate shadows interpreted as spruce.

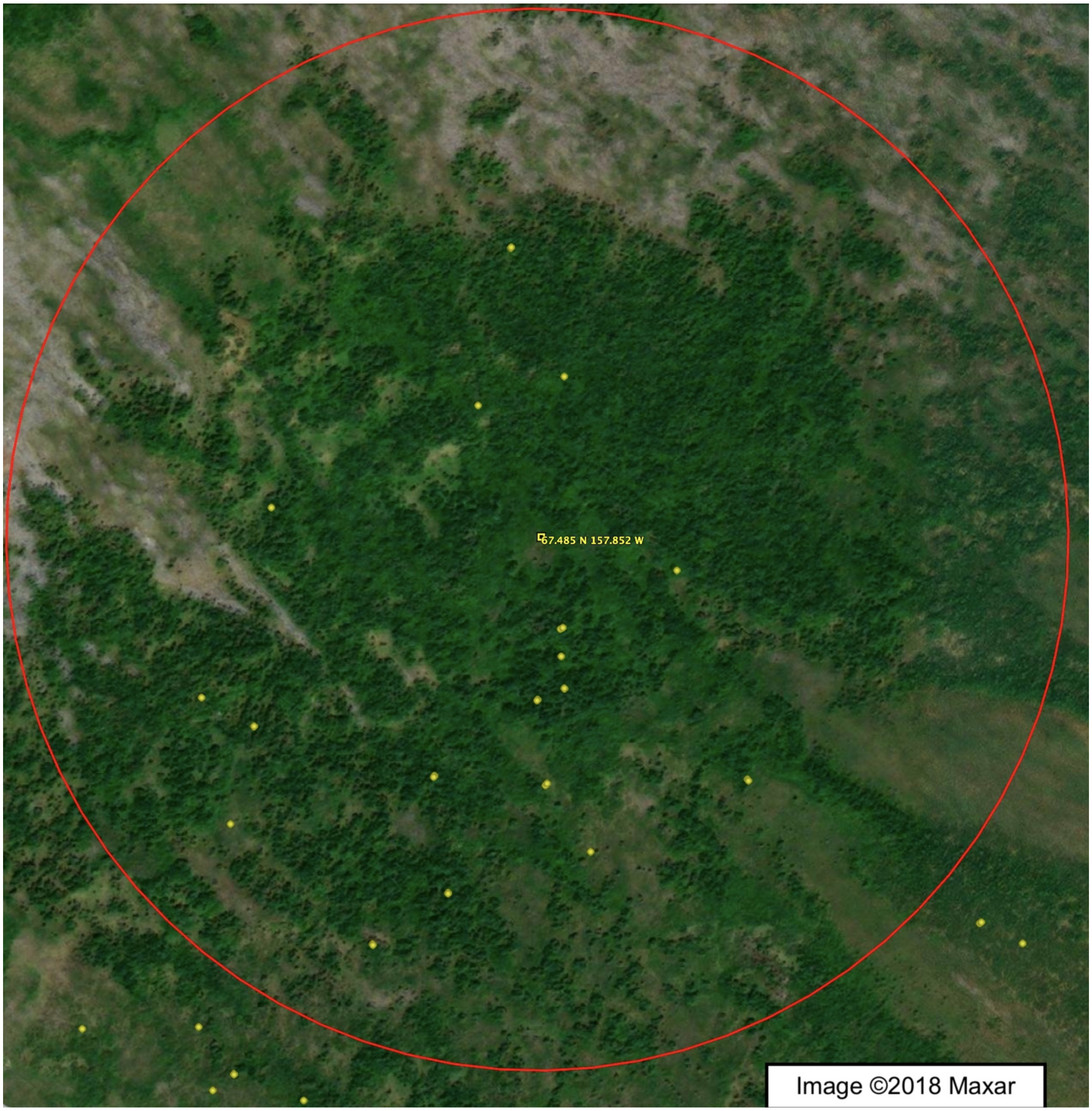


**SI Figure 8.** Same location as above (SI Fig. 7), but as a summer scene when spruce appear absent.

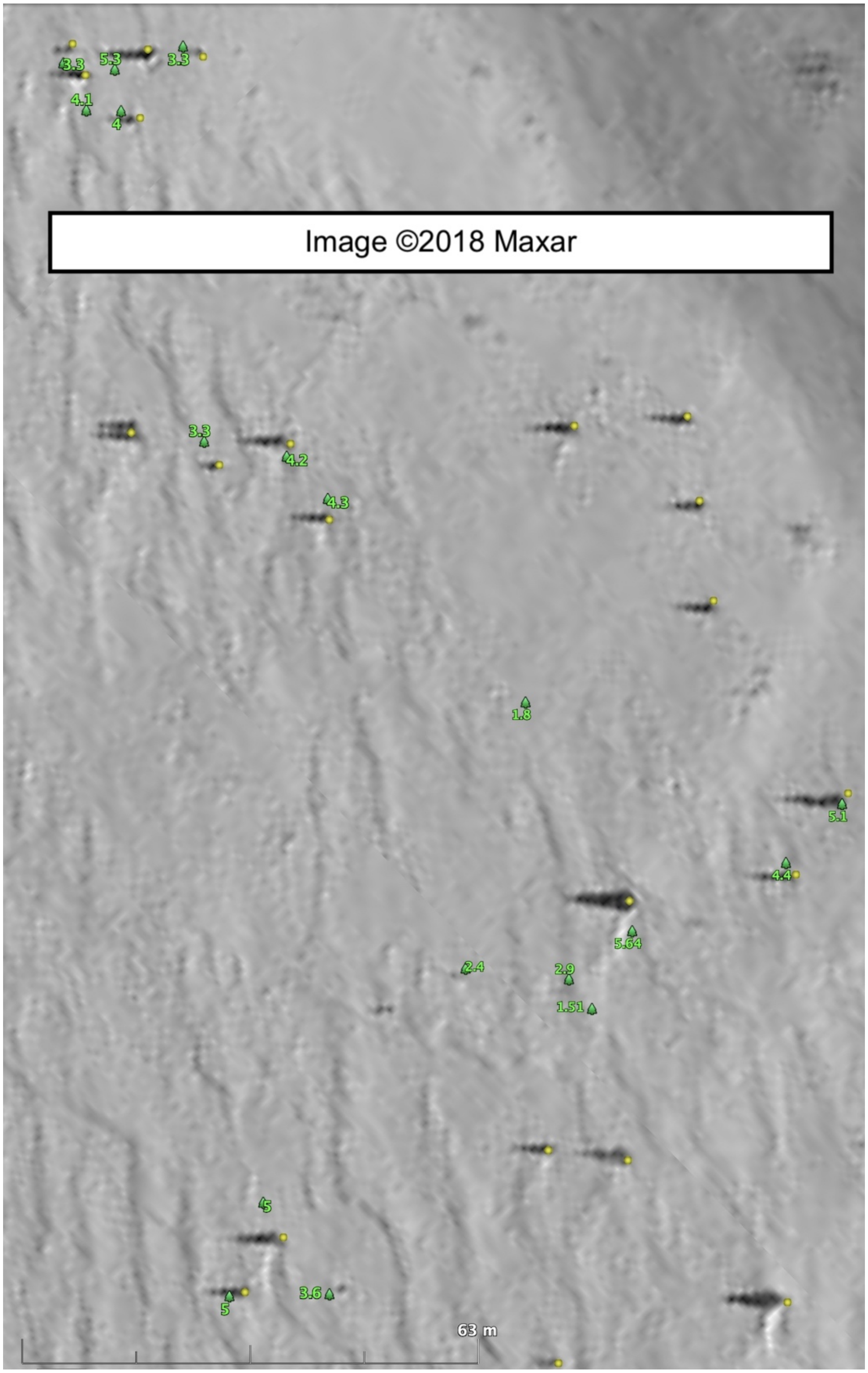


**SI Figure 9.** Spruce shadows as yellow bullets and GNSS locations as small green icons. Numbers give height above ground (m) measured in the field with laser range finder (LTI Tru-Pulse 200). Notice that field measured heights less than 3 m do not generally match with any shadows.

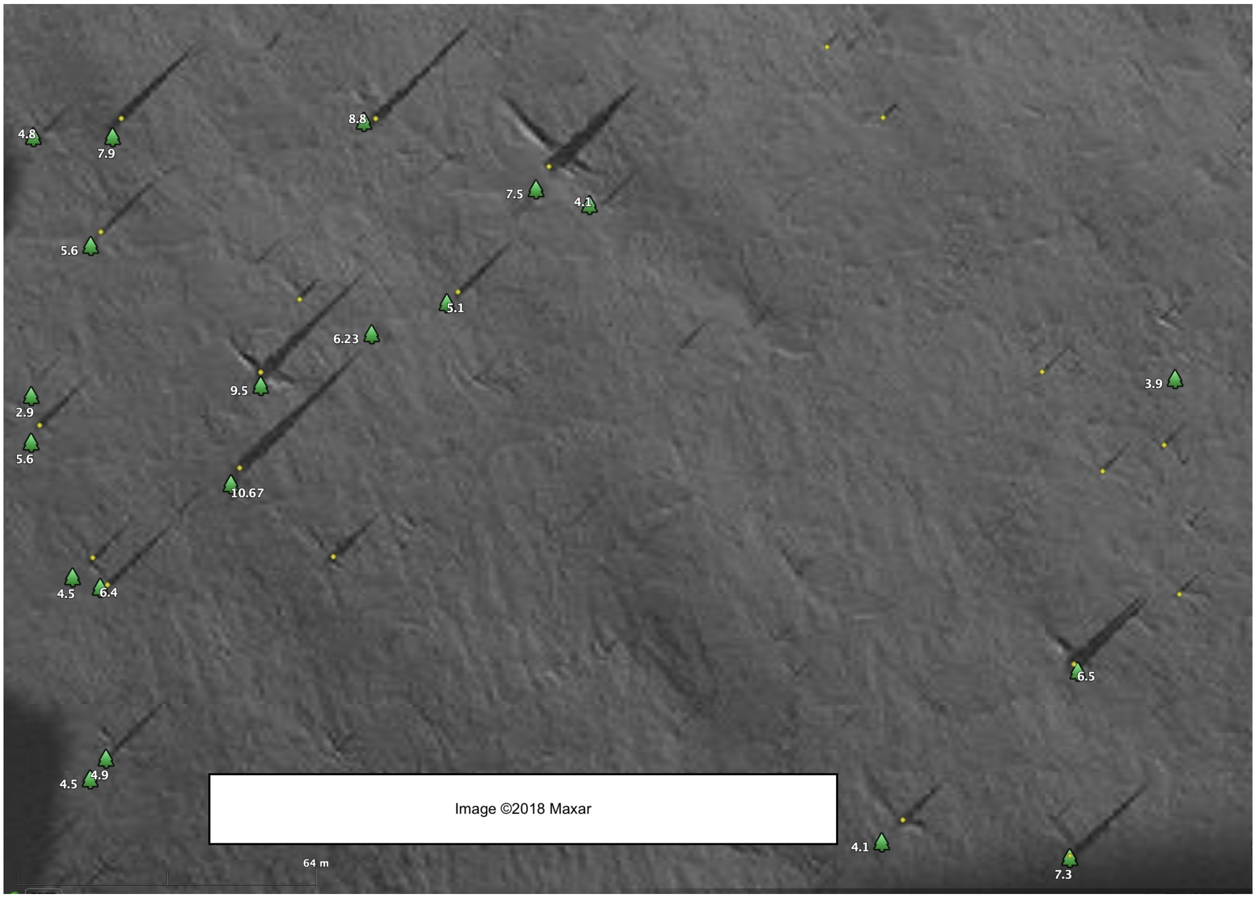


**SI Figure 10.** Spruce shadows with bases of digitized shadows as yellow bullets, GNSS locations as green tree icons, and field measured tree heights as white numbers measured at field site AmokWest.

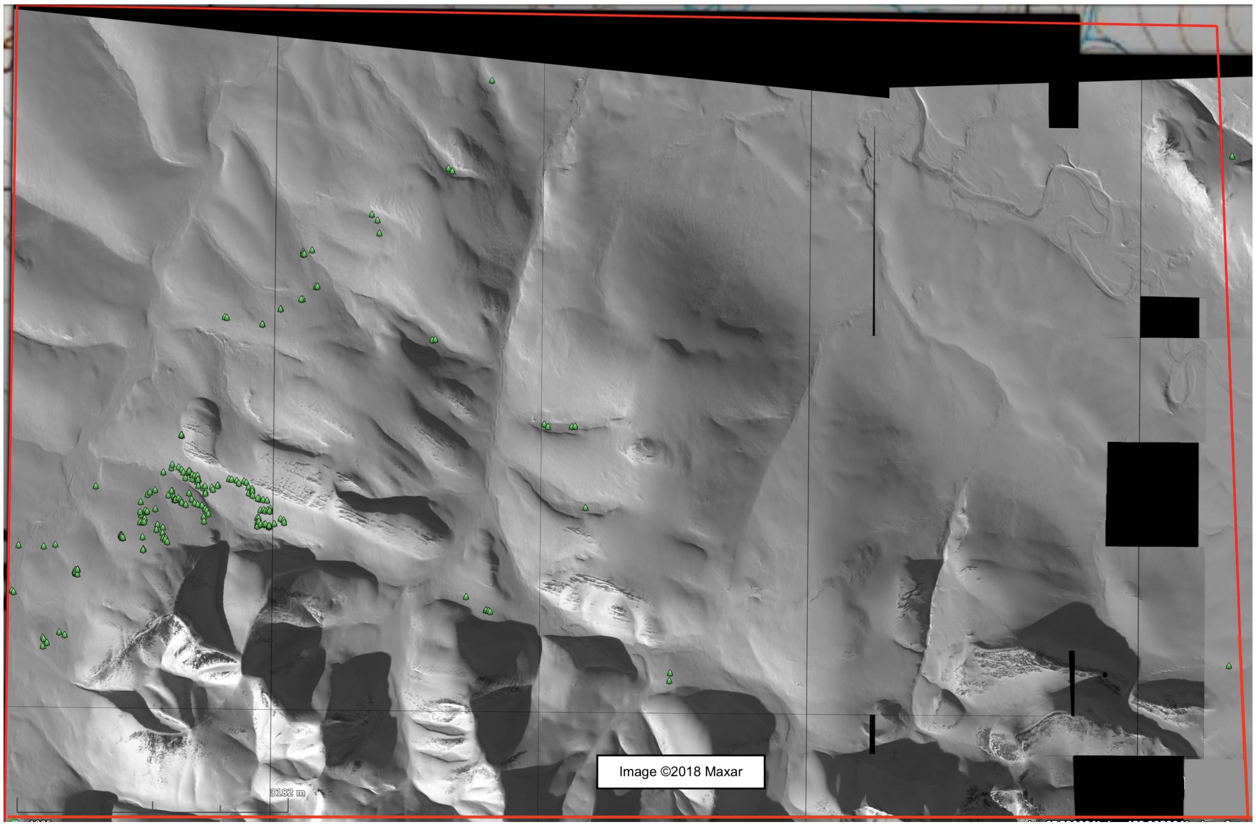


**SI Figure 11.** Green tree icons indicate locations of field-sampled adults. Lower left (SW) corner is the GaiaHill area. Along the obvious E-W trending fault line is BobWoods and north of the fault line is Buffalo Drifts. The predominant winter wind direction carries snow from the south over the smooth but corrugated topography north of the E-W trending fault line.

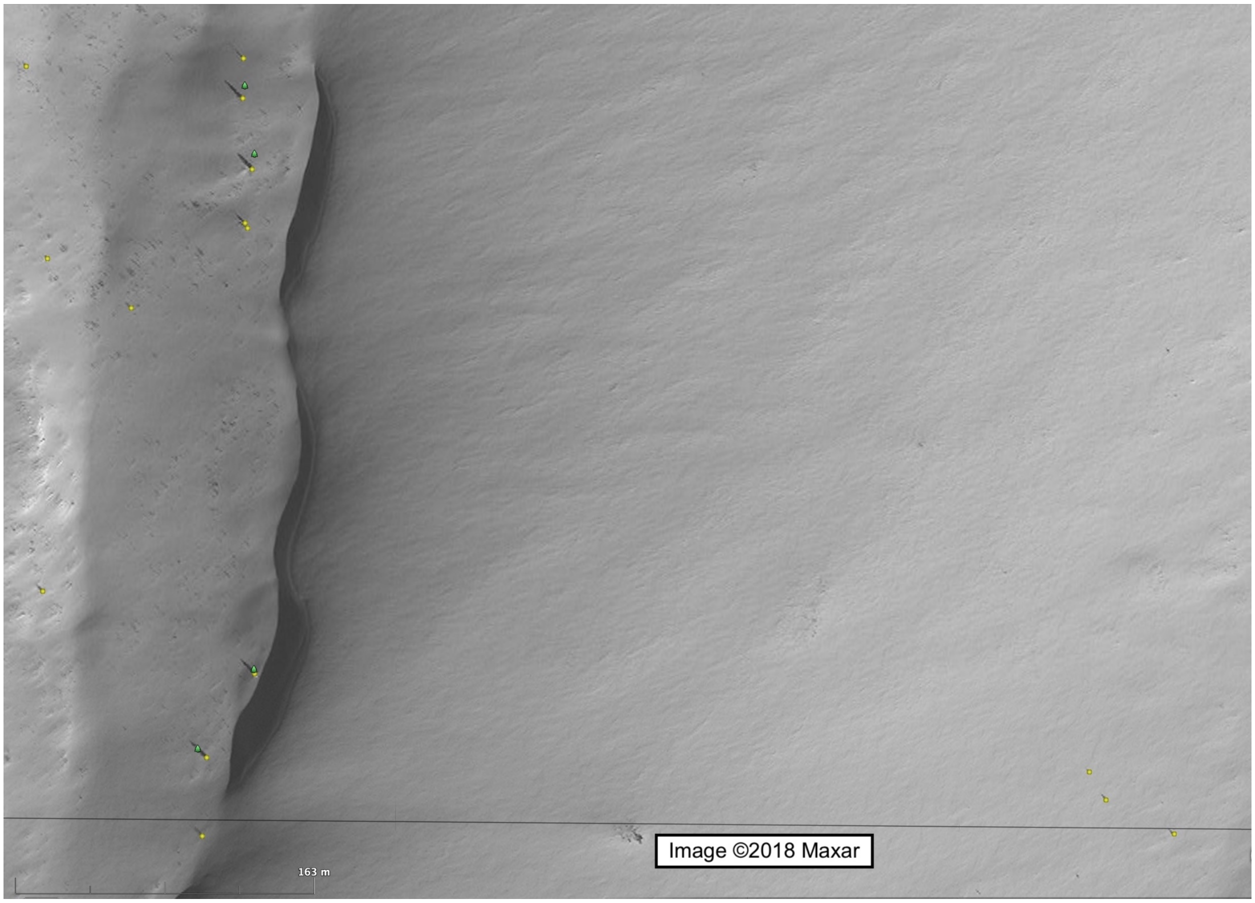


**SI Figure 12.** Snow drift and variable snow depths in "BuffaloDrift" study area. Green tree icons indicate locations of field-sampled adults. Yellow bullets indicate digitized shadows.

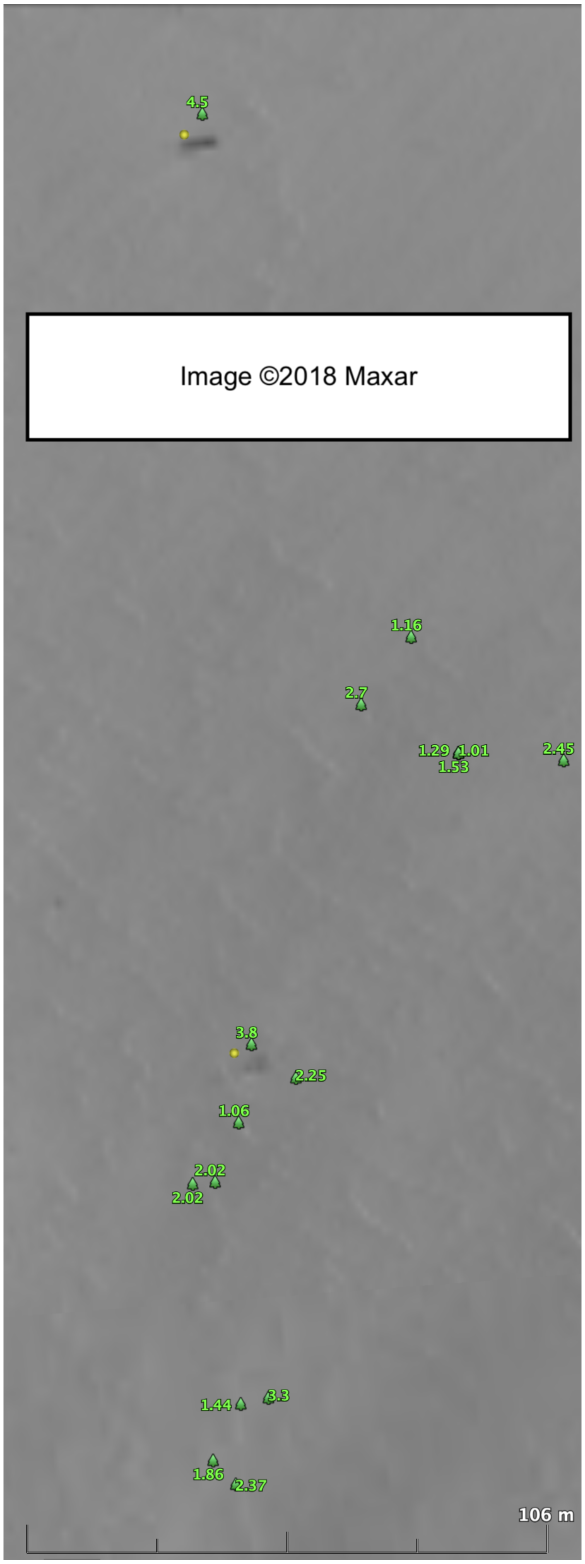


**SI Figure 13.** Tree heights (green numbers) and field locations (green icons) with visible shadows associated only with a 4.5 m and a 3.8 m tree. All other heights less than 3.8 m in the image appear to cast no shadow. This image is from the main Cutler River valley. Compare with "Cutler Main" in the violin plot.

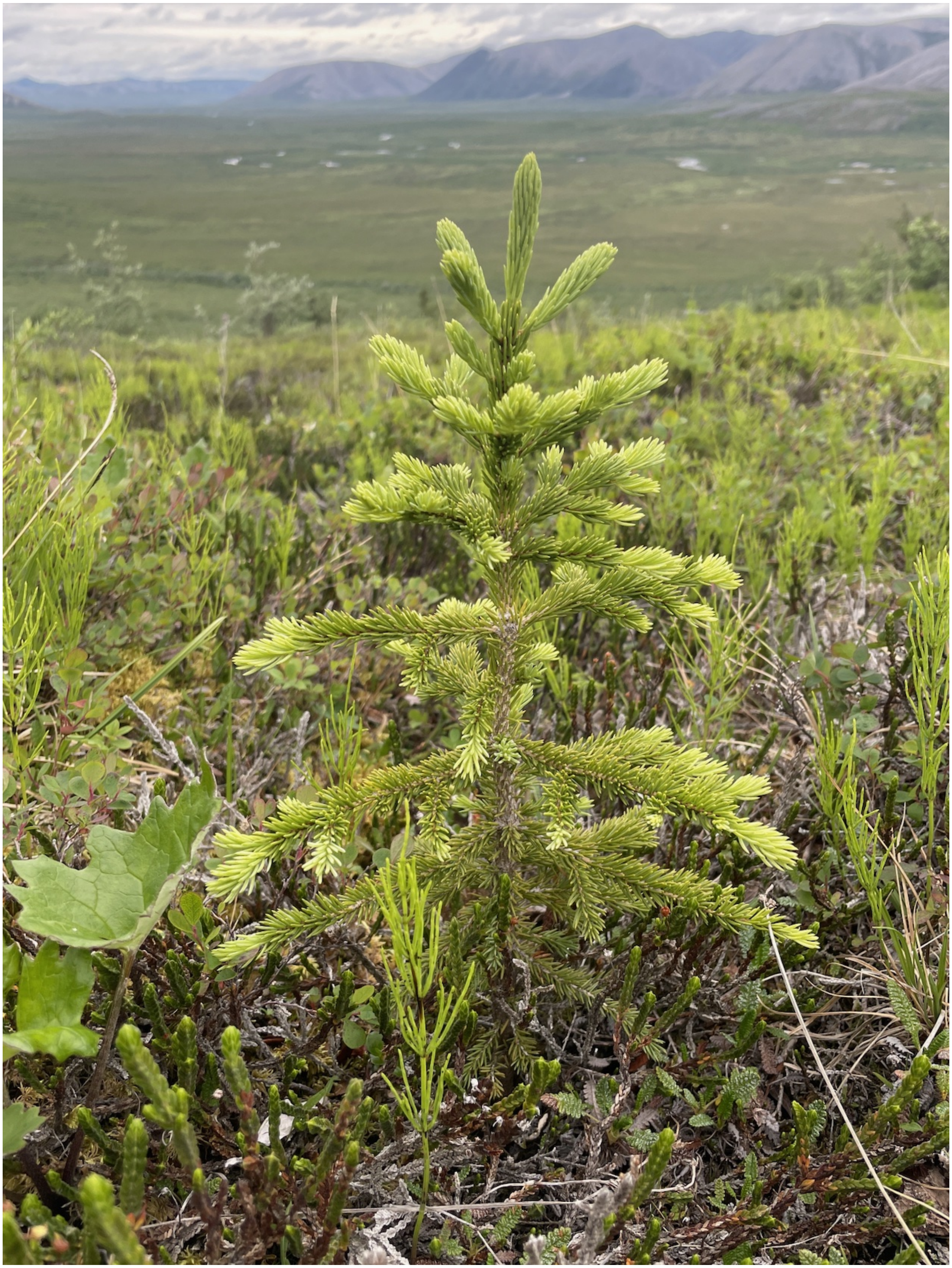

**SI Figure 14.** Fifteen year-old, 30 cm tall juvenile white spruce in Amakomanak Valley, Noatak Basin, Alaska. Photo by M. Zietlow, used with permission.
