# Supplementary figures and images for: Sufficient conditions for rapid range expansion of a boreal conifer
Source: Nature. 2022 Aug 10;608(7923):546–51. doi: 10.1038/s41586-022-05093-2 (PMC9385489; doi:10.1038/s41586-022-05093-2)

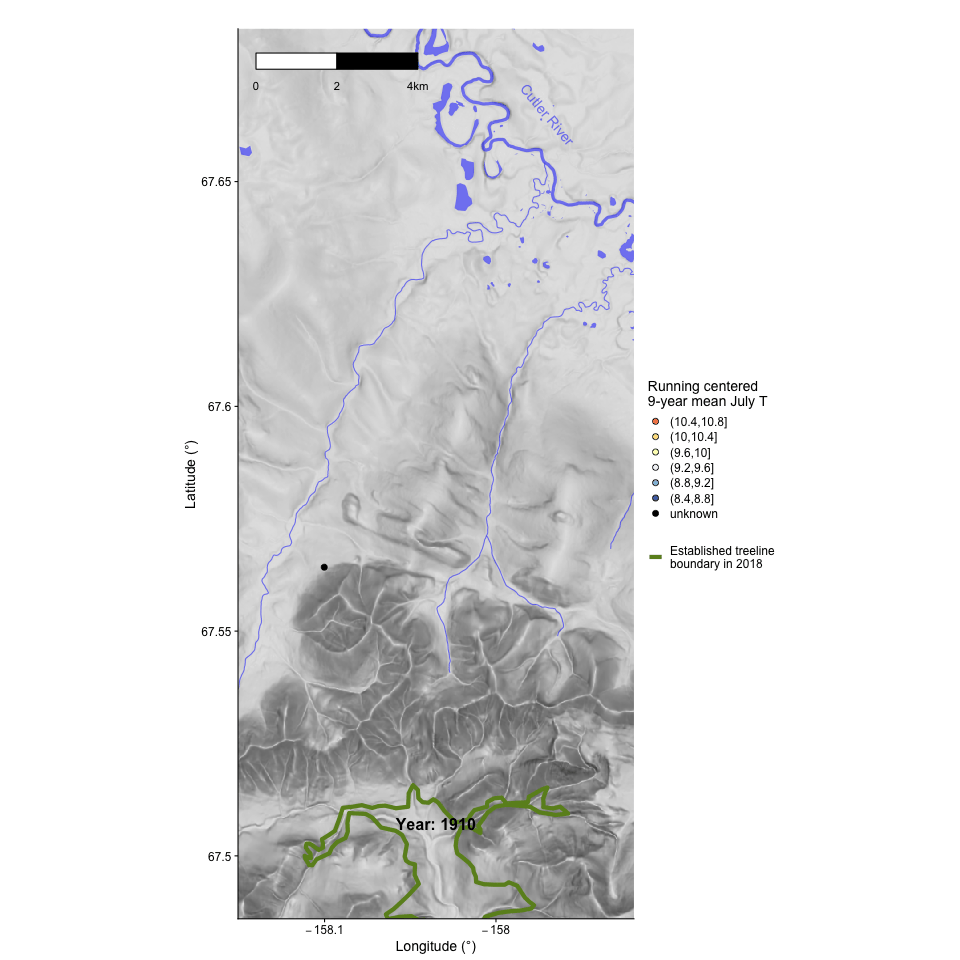

Supplement: Supplementary file 5 — A spatially explicit simulation from the western portion of “Simulated population area” shown in Extended Data Fig. 1a during1910 to 1980. Study area temperatures found by applying lapse rate (Extended Data Fig. 3) to Kotzebue July air temperaturerecord. [file 41586_2022_5093_MOESM5_ESM.gif]
